# On the Valorisation of Chitin‐Derived Furans by Milling

**DOI:** 10.1002/cssc.202401584

**Published:** 2024-10-30

**Authors:** Renan Rodini Mattioli, Camila Souza Santos, Bruna Butke de Souza, Pedro Dominguez Branco, Robert R. A. Bolt, Sarah E. Raby‐Buck, Tadeu Luiz Gomes Cabral, Claudio F. Tormena, Duncan L. Browne, Julio C. Pastre

**Affiliations:** ^1^ Institute of Chemistry State University of Campinas (UNICAMP) Campinas, SP 13083-970 Brazil; ^2^ Department of Pharmaceutical and Biological Chemistry School of Pharmacy University College London (UCL) 29-39 Brunswick Square London WC1N 1AX UK

**Keywords:** Diels–Alder, Hydrazone, Aromatics, Chitin, Mechanochemistry

## Abstract

Chitin‐derived furans offer a sustainable alternative feedstock for nitrogen appended aromatic compounds. Herein, we address the challenge of using chitin‐derived furans, 3‐acetamido‐5‐acetylfuran (**3A5AF**) and 3‐acetamido‐5‐furfural aldehyde (**3A5F**), to favour the formation of *exo* Diels–Alder adducts and 4‐acetylaminophthalimides respectively, using a mechanochemical ball‐milling technique. Mechanochemical activation is explored through the synthesis of 7‐*oxa*‐norbornene backbones with novel substitution pattern from **3A5AF** in yields up to 77 % and improved *exo*:*endo* selectivity compared to solution‐phase reactions. The synthesis of 4‐acetylaminophthalimides from **3A5F** in yields up to 79 % is also showcased from hydrazone derivatives.

## Graphical abstract

This work showcases the selective synthesis of *exo* Diels–Alder adducts with novel substitution pattern from **3A5AF** under heated milling in up to 77 % yield. It is also demonstrated the synthesis of nitrogen appended phthalimides from **3A5F**‐derived hydrazone under both heated and room temperature milling in up to 79 % yield.

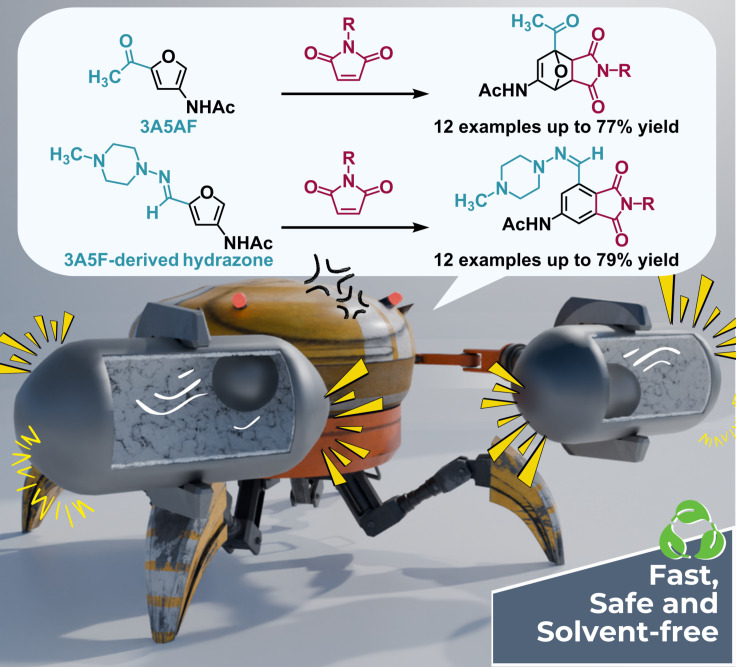

## Introduction

Bio‐based platform chemicals have recently emerged as a sustainable alternative to commonly used fossil‐fuel derivatives in synthetic organic chemistry.^[1]^ These platforms are most often obtained from lignocellulosic biomass and more recently from chitin.^[2]^ Chitin is the second most abundant bio‐polymer after cellulose and a major component of fungi cell walls and the exoskeletons of insects and crustaceans.^[3]^ This bio‐polymer is composed of *N*‐acetyl‐glucosamine (NAG) units that can be readily acquired via acidic, enzymatic or thermal depolymerisation of chitin. Bio‐based nitrogenated furans represent a route to high added value products such as substituted 4‐acetylaminophthalimides, anilides[^4^, ^5^] and benzene derivatives[^6^, ^7^, ^8^, ^9^, ^10^] for the synthesis of polymers. Functionalisation of lignocellulosic‐derived furans has been extensively explored through highly atom efficient Diels–Alder (DA) reactions to produce aromatics via subsequent aromatisation.[^11^, ^12^, ^13^, ^14^, ^15^, ^16^, ^17^, ^18^]

**Scheme 1:**
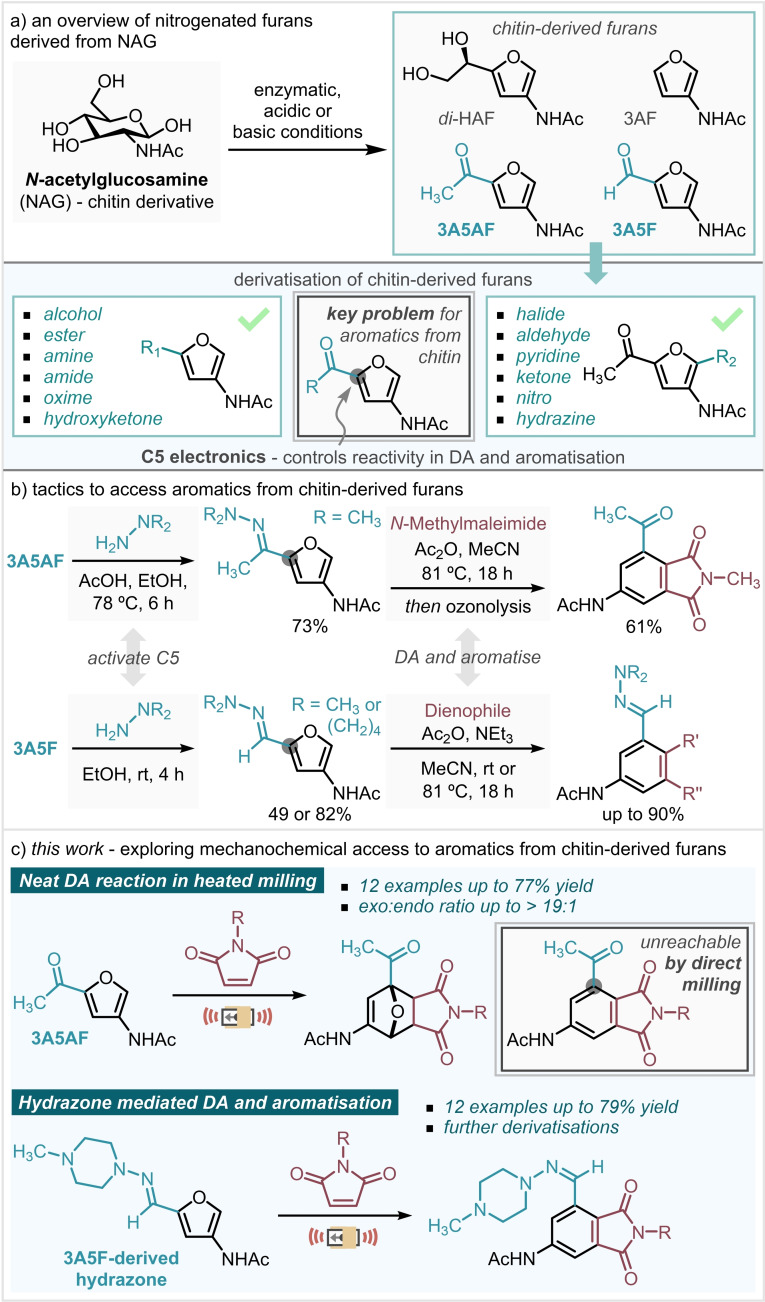
Nitrogenated furans derived from chitin as valuable compounds in synthesis and its use to afford aromatics via hydrazone intermediates. Mechanochemistry can enhance both the selectivity and yield of the DA reaction.

Recently, chemical valorisation has been widely applied to chitin derived furans.[^19^, ^20^, ^21^, ^22^, ^23^, ^24^] Several methodologies have been described for the direct transformation of both NAG and chitin into furans (Scheme 1a): 3‐acetamido‐5‐acetylfuran (**3A5AF**),[^25^, ^26^, ^27^] dihydroxyethyl acetamidofuran (**Di‐HAF**),^[22]^ 3‐acetamidofuran (**3AF**)^[23]^ and 3‐acetamido‐5‐furfural aldehyde (**3 A5F**).^[24]^

The valorisation of **3A5AF** was recently investigated by Gomes *et al* ^[28]^ and Pastre *et al* ^[4]^ using DA reaction. Notably, the DA reaction of **3A5AF** is limited to maleimides due to an unfavourable HOMO‐LUMO gap with other dienophiles. In methanol, the experimental *exo*:*endo* selectivity of the DA adduct is low (5 : 1) with a combined isomeric yield of 45 % after 16 hours. This yield was increased to 73 % with exclusive formation of the *exo* product using buffered deuterated dimethyl sulfoxide over 24 hours.[^4^, ^28^] DA adducts derived from **3A5AF** did not proceed under aromatisation conditions, likely due to destabilising effect of the electron‐withdrawing carbonyl group.^[4]^ However, further strategic advancements have shown that DA reactivity can be achieved by prior chemical derivatisation of electron‐poor furans by reduction, hydration or hydrazone formation reactions.[^5^, ^10^, ^12^, ^29^] Formyl derivatisation to a hydrazone increases the orbital coefficient at the C5 position allowing spontaneous aromatisation at temperatures below 50 °C (Scheme 1b).[^12^, ^29^, ^30^, ^31^, ^32^] Minnaard and co‐workers used this approach to circumvent the unfavourable thermodynamics of **3A5AF**‐derived adducts towards aromatisation (Scheme 1b).^[5]^ Formation of a hydrazone with *N*,*N*‐dimethylhydrazine allowed a one‐pot DA/aromatisation with *N*‐methylmaleimide, followed by ozonolysis to restore the ketone.^[5]^ Although this approach allows access to high added value aromatics, the reaction is limited by the use of *N,N*‐dimethylhydrazine and 1‐aminopyrrolidine.^[5]^ These hydrazines are supposed to be toxic, flammable, explosive and, in view of their nature, they are not commercialised in several countries.

Mechanochemistry is an emerging technology that provides an alternative to solution‐based synthesis. It has been shown to help reduce waste by removing the need for bulk solvent,[^33^, ^34^, ^35^, ^36^] thus improving sustainability parameters compared to traditional solution procedures.^[37]^ Ball mills have been used for a wide range of synthetic organic transformations with recent findings suggesting mechanochemistry can offer improvements in reaction rates, selectivity and alternative reactivity.[^38^, ^39^, ^40^, ^41^] Remarkable works on mechanochemical valorisation has demonstrated the activation of cellulose to obtain high‐yielding oligosaccharides,^[42]^ sugar alcohols^[43]^ and furfurals[^44^, ^45^, ^46^] under mild conditions. To the best of our knowledge, mechanochemistry has been not yet applied to the valorisation of chitin derivatives towards to high added value nitrogenated aromatics.

In this work, we showcase a mechanochemical DA reaction to afford 7‐*oxa*‐norbornene backbones derived from **3A5AF**, with improved yield and diastereoselectivity compared to solution‐phase alternatives (Scheme 1c). In addition, we demonstrate the use of mechanochemistry in the synthesis of substituted 4‐acetylaminophthalimides from **3A5F** using a hydrazone mediated approach.

## Results and Discussion

We commenced our study starting from our previous results, in which **3A5AF** reacted with maleimide in methanol at 50 °C for 16 h to afford the DA adduct **1 a** in 45 % and *exo*:*endo* ratio=5 : 1.^[4]^ Unsatisfied with both the yield and selectivity of the solution‐phase protocol, we decided to investigate **3A5AF**’s reactivity through mechanochemical methods.

Using **3A5AF** and maleimide as the model substrates with sodium chloride as a grinding auxiliary, optimisation of DA reaction by milling was covered by the screening of ball diameters, maleimide equivalents, solid and liquid grinding agents, Lewis acids, temperature and reaction time (see Tables S2–S9 for full range of conditions tested). Afterwards, some additional experiments were performed with the optimised conditions (Table 1, entry 1). An increased ball diameter (10 mm) yielded 67 %, with further diameter increase (12 mm) yielding only 48 % (Table 1, entries 2–3). Reducing the equivalents of maleimide to 1.0 led to a drop in yield to 56 %, while increasing to 3.0 equivalents resulted in negligible yield change (Table 1, entries 4–5). The optimal amount of sodium chloride was 2.5 mass equivalents, with 2.0 and 3.0 equivalents providing similar yields, however the latter rendered a reduction in diastereoselectivity to 5 : 1 (Table 1, entries 6–7). Probably, there must be higher energy input and therefore less control over the selectivity when increasing the amount of auxiliary. Notably, no enhancements were observed for yield or diastereoisomeric ratio with the use of Lewis acids (Table 1, entries 8–9).

**Table 1:** Optimisation experiments of **3A5AF** in DA reaction by milling.

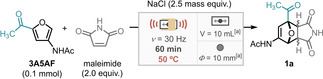			
Entry	Variation from standard conditions	Yield of **1 a** (%)^[b]^	*Exo*:*endo* ratio of **1 a**
1	None	67 (62^[c]^)	9 : 1
2	Ball size=8.0 mm	64	4 : 1
3	Ball size=12.0 mm	48	6 : 1
4	Maleimide (1.0 equiv.)	56	4 : 1
5	Maleimide (3.0 equiv.)	64	4 : 1
6	NaCl (2.0 mass equiv.)	61	9 : 1
7	NaCl (3.0 mass equiv.)	60	5 : 1
8	LiCl as catalyst	67	3 : 1
9	MgOTf_2_ as catalyst	42	2 : 1
10	Temperature=40 °C	62	7 : 1
11	Temperature=60 °C	57	>19 : 1
12	Time=15 min	49	3 : 1
13	Time=90 min	62	15 : 1

[a] Stainless steel materials. [b] Determined by ^1^H NMR analysis using mesitylene as internal standard. [c] Isolated yield.

**Scheme 2:**
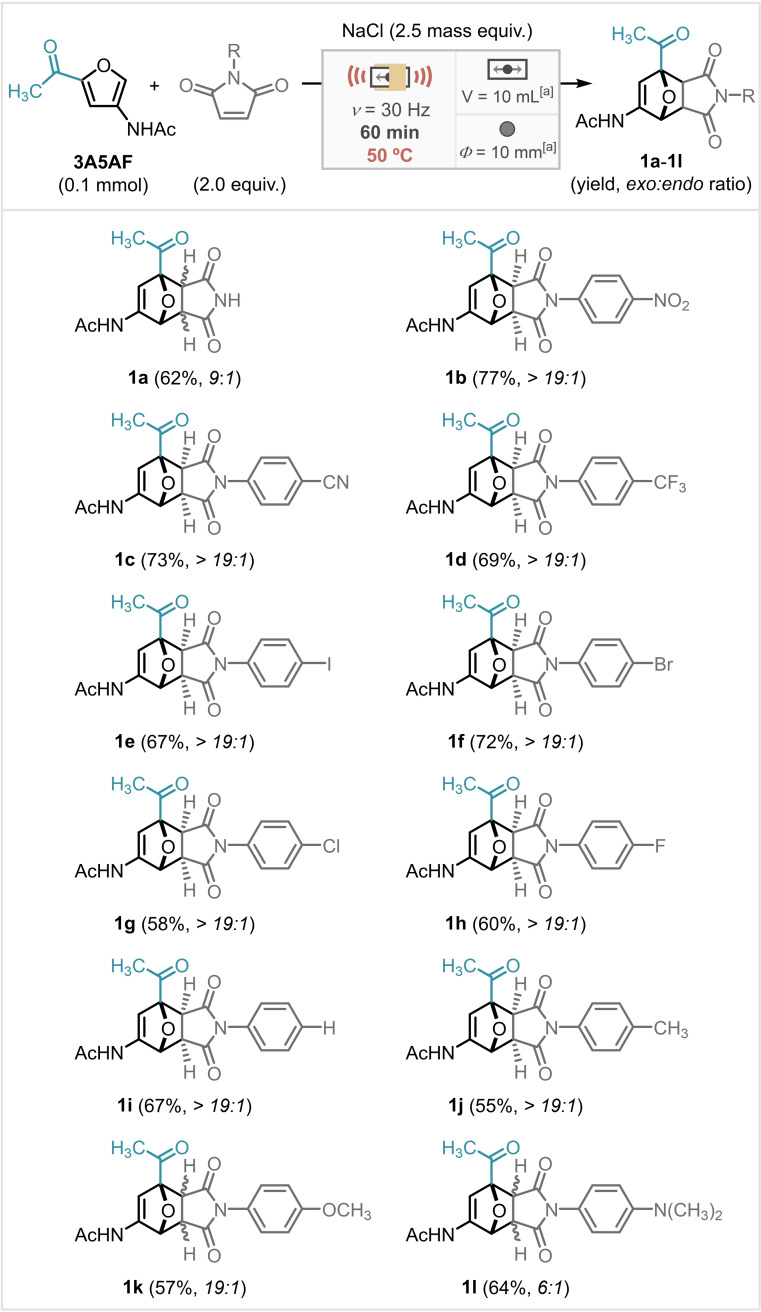
Scope of *N*‐(4‐phenyl)substituted maleimides in DA reaction with **3A5AF** with optimal conditions. [a] Stainless steel materials. Isolated yields.

Examining reaction temperature and time (Table 1, entries 10–13), at 60 °C for 60 minutes the *exo* isomer is formed exclusively, with a reduced yield. The optimal conditions for the formation of **1 a**, was deemed to be 50 °C for 60 minutes. With these optimal conditions in hand, DA adduct **1 a** was isolated in 62 % yield (Table 1, entry 1). These conditions were then applied to a scope of eleven *N*‐(4‐phenyl)substituted maleimides (Scheme 2). We observed that the average yield was around 65 %, in agreement with the model substrate **1 a** after optimisation.

Varying the electronics of the *para* position of the phenyl ring demonstrated that nitro and cyano substituents outperformed the model reaction, giving adducts **1 b** and **1 c** in 77 % and 73 % respectively. This can be rationalised by the mesomeric effect of these substituents, lowering the energy of the maleimide LUMO and, thus, reducing the HOMO‐LUMO gap. Theoretical calculations of LUMO energy for nitro (major yield) and methyl (minor yield) substituted maleimides exhibited HOMO‐LUMO energy gaps of −3.81 eV and −3.93 eV, respectively, which differ roughly 0.10 eV and corroborate to the tendence of experimental yields and increased reactivity by mesomeric effect (see Tables S15 and S16 for more details). An improvement in *exo*:*endo* selectivity was observed in all cases (≥19 : 1), except for product **1 l** (6 : 1). Notably, the strong electron donating group in **1 l** lowers the reaction rate and reduces the selectivity for the *exo* product.

Unfortunately, DA adducts **1 a**–**1 l** did not directly undergo aromatisation in the mill. The employment of acids was explored, however the prolongated use of strong acids with stainless steel materials is avoided due to corrosion. On the other hand, supported acids, such as *p*‐toluenesulfonic acid (Amberlyst‐15), are an adequate alternative to liquid acids, however the aromatisation of DA adducts was unsuccessful. Notably no starting DA adduct was recovered likely due to decomposition.

**Scheme 3:**
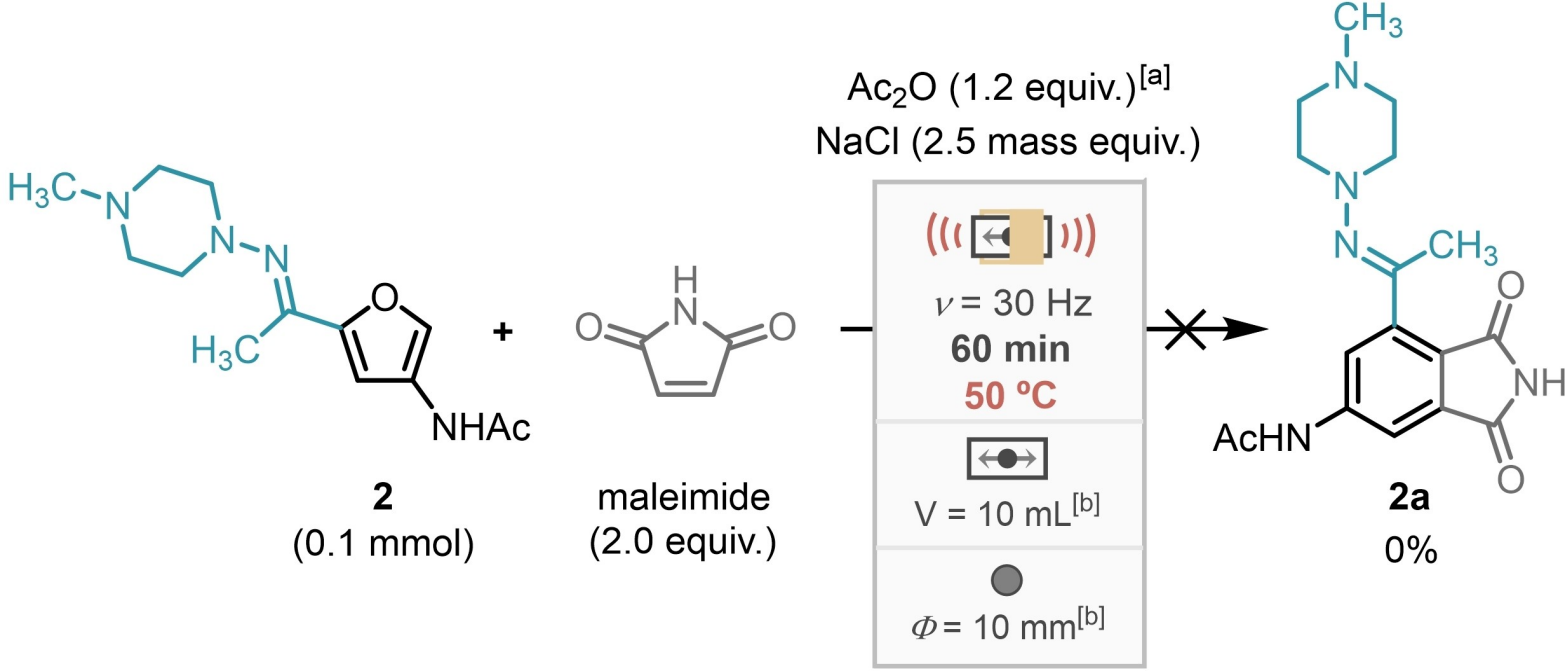
Unsuccessful DA/aromatisation performed with hydrazone **2** in milling. [a] With or without Ac_2_O. [b] Stainless steel materials.

Attention was then turned to an alternative strategy to achieve aromatisation by attenuation of the electronics at C5, through the installation of a hydrazone moiety (to **3A5AF**) and subsequent DA/aromatisation steps. 1‐Amino‐4‐methylpiperazine was selected as the model hydrazine due to its relatively low harmfulness, toxicity, and volatility compared to other hydrazine candidates as well as the presence of piperazine ring notably observed in several molecules with biological properties.^[47]^ Unfortunately, the initial preparation of the **3A5AF**‐derived (hydrazone **2**) and submission to DA/aromatisation conditions in the mill were unsuccessful (Scheme 3).

**Scheme 4:**
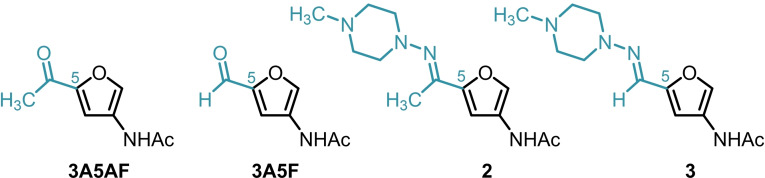
Furanic structures used in computational studies for HOMO‐LUMO energy gap determination.

Computational studies were carried out using the Density Functional Theory (DFT) to better understand these results and inform a revision of the strategy. The theoretical reactivity (HOMO‐LUMO energy gap) of dienes **3A5AF** (−3.94 eV) and **3A5F** (−4.02 eV), and the respective hydrazones derived from **3A5AF** (hydrazone **2**, −3.06 eV) and **3A5F** (hydrazone **3**, −2.91 eV), with maleimide as dienophile were compared (Scheme 4). **3A5F** and its corresponding hydrazone **3** were included to give us a bigger picture of the methyl effect on furan **3A5AF** and hydrazone **2** reactivities. The calculated HOMO‐LUMO gap suggests that hydrazone **3** is the most reactive, then **2** and, as expected, **3A5F** and **3A5AF** are the least reactive. Indeed, the DA reaction with **3A5F** in solution‐phase gives the adduct in only 7–21 % yield (see Table S10 for more details).

The improved reactivity of hydrazone **3** can be rationalised by its higher HOMO energy, reducing the energy gap with LUMO from maleimide. Hydrazone **3** also has a considerable increase in orbital coefficient on carbon C5 (see Tables S15 and S17 for more details). Furthermore, hydrazone **3** has favourable kinetics due to the absent steric repulsion caused by the methyl group of hydrazone **2** during 7‐*oxa*‐norbornene ring opening and aromatisation steps.^[5]^ These results redirected our investigations to hydrazone **3** for further mechanochemical DA reaction followed by a spontaneous aromatisation process.

*N*‐(4‐Nitrophenyl)maleimide was chosen as model dienophile to optimise the aromatisation reaction with hydrazone **3**. Reactions were performed at room temperature and 50 °C using initially 2.0 equivalents of *N*‐(4‐nitrophenyl)maleimide, yielding 56 % and 65 % respectively (Table 2, entry 2). Surprisingly, deacetylated product **3 a’** was formed in 8 % at both temperatures. To prevent the undesired deacetylation, acetic anhydride was added resulting in the exclusive formation of **3 a** in 58 % and 67 % yield respectively (Table 2, entry 3). Triethylamine was also added, turning the reaction mixture slightly basic, however no significant benefit was observed (Table 2, entry 4).

**Table 2:** Optimisation experiments of hydrazone **3** aromatisation in milling.

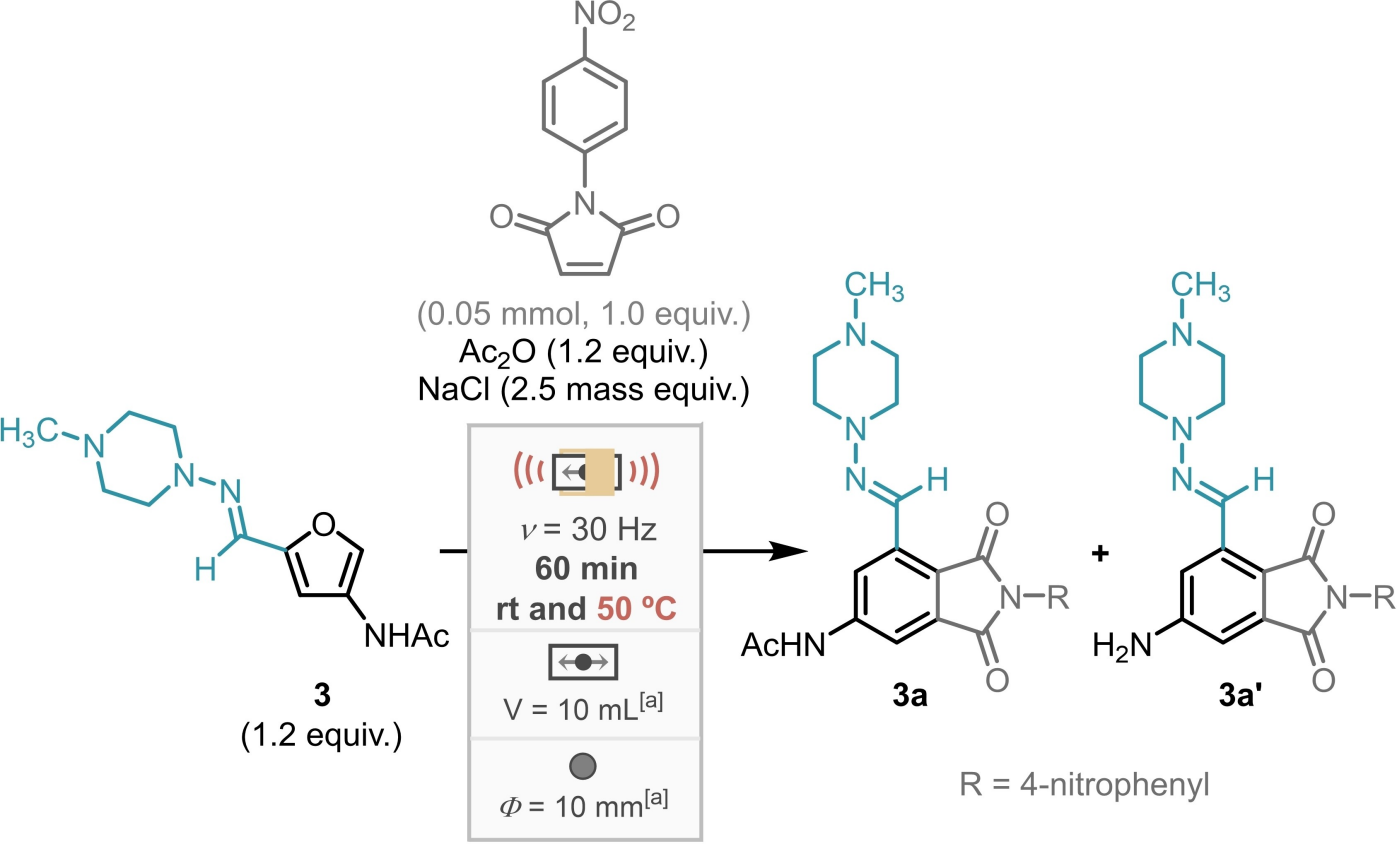					
Entry	Variations from optimised conditions (equiv.)	Room temperature		50 °C	
		**3 a %**	**3 a’ %**	**3 a %**	**3 a’ %**
1	None	65	0	69	0
2	*N*‐(4‐Nitrophenyl)maleimide (2.0), **3** (1.0), without Ac_2_O	56	8	65	8
3	*N*‐(4‐Nitrophenyl)maleimide (2.0), **3** (1.0)	58	0	67	0
4	*N*‐(4‐Nitrophenyl)maleimide (2.0), **3** (1.0), with NEt_3_ (2.5)	58	0	66	0
5	**3** (1.0)	62	0	64	0

[a] Stainless steel materials. [b] Determined by ^1^H NMR analysis using mesitylene as internal standard.

Acetic anhydride reduced the formation of **3 a’** without the requirement of added external base. Notably it was observed that the hydrazone incorporates 1.0 equivalent of an organic tertiary base. Additionally, the stoichiometry of hydrazone was reduced to 1.0 equivalent with slightly decrease in yield (Table 2, entry 5). The optimal conditions were achieved by keeping the amount of hydrazone to 1.2 equiv. (Table 2, entry 1), with **3 a** yielded in 65 % and 69 % at room temperature and 50 °C respectively (see Table S13 for full range of conditions tested). The stability of hydrazone **3** was examined under mechanochemical conditions in the absence of the maleimide (see Table S14 for more details). Milling at 50 °C the hydrazone **3** degraded with only 35 % recovery, whereas at room temperature 90 % of starting material was recovered. Therefore, an excess of hydrazone **3** was elected pivotal for optimal conditions. More details of varied amounts of acetic anhydride and grinding agents can be found in *Supporting Information* along with solution‐phase comparisons (see Tables S11–S13 for more details).

**Scheme 5:**
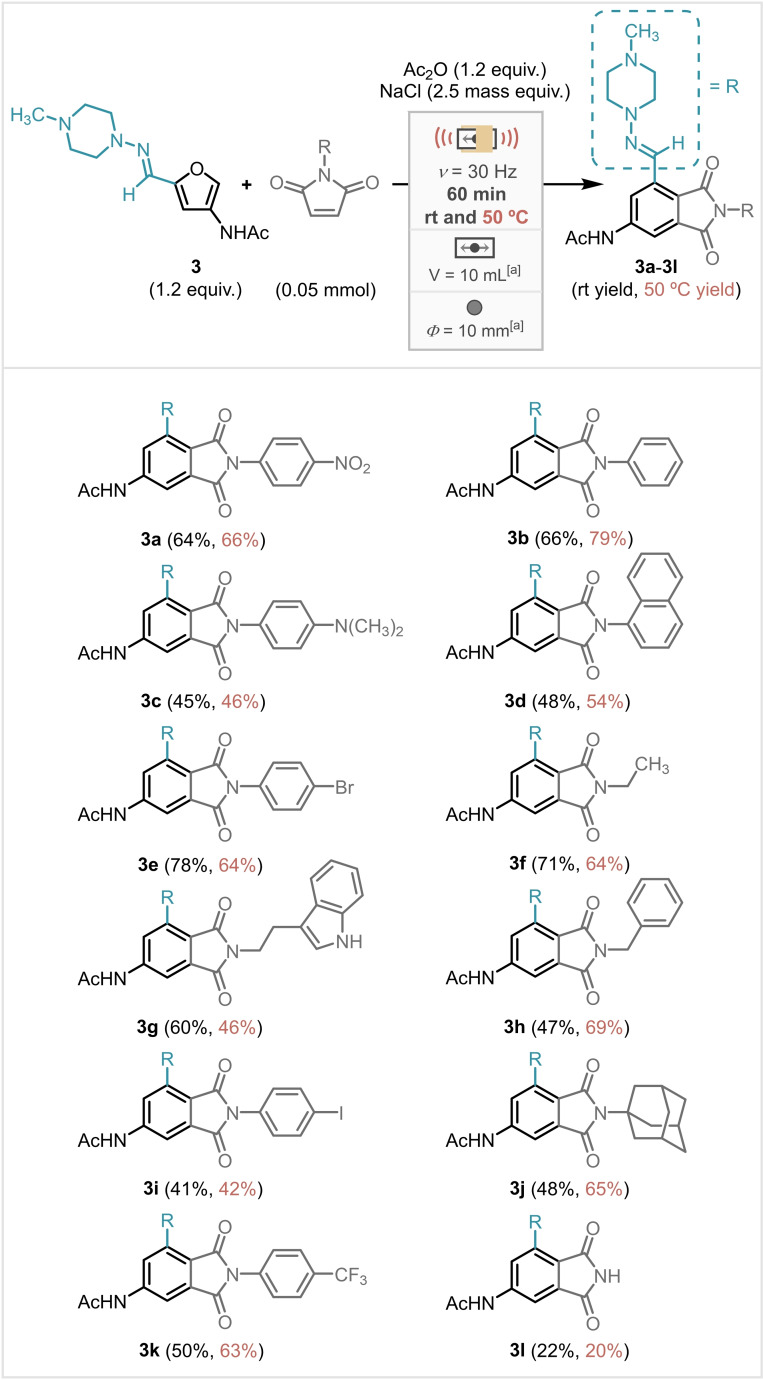
Scope of maleimides in cascade protocol with hydrazone **3** in milling at room temperature and 50 °C with isolated yields.

This novel cascade DA/aromatisation protocol by milling was tested on a range of *N*‐substituted maleimides with hydrazone **3** yielding 4‐acetylaminophthalimides **3 a**–**3 l** in 20–79 % (Scheme 5).

It was observed in some cases that there was a significant variation in yield between room temperature and 50 °C, with **3 h** and **3 j** yielding around 20 % higher at 50 °C. Generally, greater yields of the aromatics were obtained at 50 °C, however the opposite trend was also observed for **3 e**–**3 g**. This is likely attributable to a difference in rates between the degradation of the hydrazone starting material and the aromatisation reaction. Synthesis of compound **3 b** was also evaluated in a 10‐fold scale‐up experiment (0.5 mmol) affording similar yields, purified by either chromatography (72 %) or recrystallisation (76 %).

**Scheme 6:**
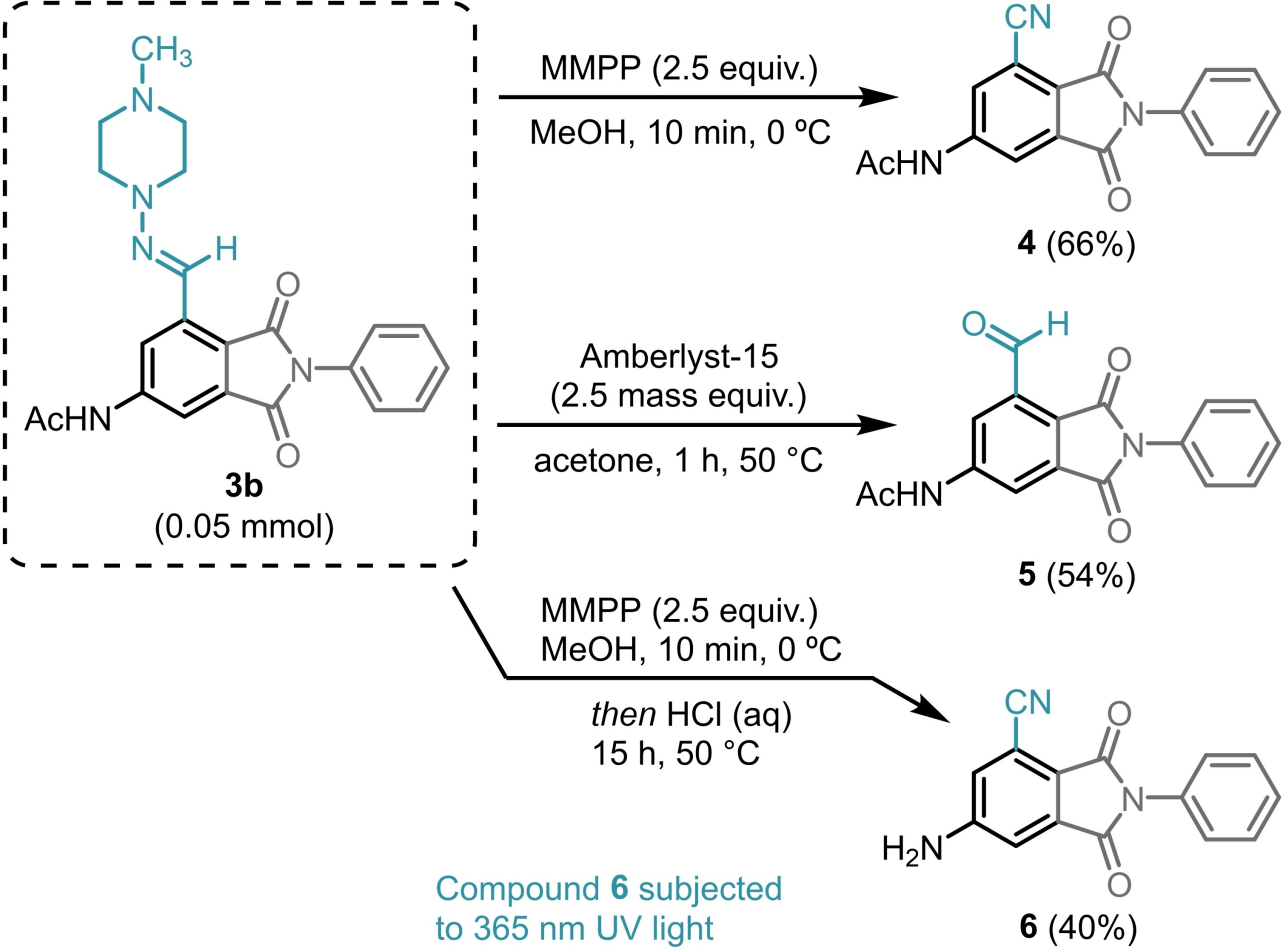
Follow‐up chemistry at solution‐phase. Functionalisation of 4‐acetylaminophthalimide **3 b** into three higher added‐value derivatives **4**–**6**.

Hydrazones are privileged structures in medicinal chemistry due to their biological activity for the potential treatment of several diseases, including Alzheimer′s, cancer, inflammation, and leishmaniasis.[^48^, ^49^, ^50^, ^51^, ^52^] Modifications of 4‐acetylaminophthalimide **3 b** were investigated in solution‐phase to demonstrate the versatility of this aromatic product (Scheme 6).

Oxidative cleavage of the hydrazone was performed using magnesium monoperoxyphthalate (MMPP) providing the nitrile derivative **4** in 66 % yield. The hydrazone moiety in **3 b** was also hydrolysed using Amberlyst‐15, affording aldehyde **5** in 54 % yield. Compound **6** was synthesised sequentially *via* oxidation with MMPP, followed by hydrolysis with aqueous hydrochloric acid solution to give a 40 % yield over the two steps. Interestingly, compound **6** exhibited fluorescence when subjected to 365 nm UV light source, likely due to the removal of the acetyl protecting group.


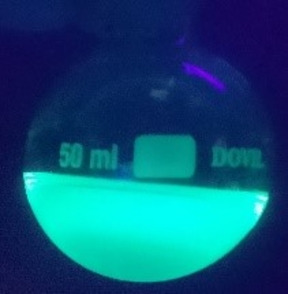


## Conclusions

A new method for the mechanochemical valorisation of chitin biomass derived furans has been developed in the synthesis of novel high added value 4‐acetylaminophthalimides. Encompassing many of the principles of Green Chemistry, this methodology takes advantage of mechanochemical techniques. It was demonstrated that activation of **3A5AF** and *N*‐(4‐phenyl)substituted maleimides by mechanochemistry favours the formation of the *exo* DA product within one hour, without the use of solvents or additives at mild reaction temperatures (50 °C). Electron withdrawing groups in *N*‐(4‐phenyl)substituted maleimides, *e. g*. nitro (77 %) and cyano (73 %), benefited both yield and *exo* isomer selectivity (>19 : 1). In comparison to solution‐phase, the DA reaction of **3A5AF** had improved specificity. These 7‐*oxa*‐norbornenes with high diastereoisomeric purity can be useful intermediates for the synthesis of novel potentially bioactive compounds.

Moreover, we developed a non‐petrochemical route to functionalised pharmaceutically relevant nitrogenated aromatics using renewable chemical feedstocks and solvent minimal conditions. The formation of a hydrazone derivative of **3A5F** proved effective for producing *N*‐containing aromatic compounds in yields ranging from 20 to 79 %. This was not feasible for **3A5AF**‐derived hydrazones as the additional methyl group causes a steric repulsion during the ring opening, inhibiting the aromatisation step under our experimental conditions. Further solution‐based synthetic derivatisations of 4‐acetylaminophthalimides were also showcased towards the versatility of the molecules produced.

## Author Contributions

Conceptualisation, RRM, CSS and JCP; Methodology, RRM, CSS, BBS, PDB, RRAB and SERB; Formal Analysis, TLGC and CFT; Investigation, RRM, CSS, BBS, PDB, RRAB and SERB; Resources, RRM, CSS, DLB and JCP; Writing–Original Draft, RRM, CSS, BBS, PDB and JCP; Writing–Review & Editing, RRM, CSS, BBS, PDB, RRAB, SERB, TLGC, CFT, DLB and JCP; Visualisation, RRM, CSS, BBS, PDB, RRAB and SERB; Supervision, DLB and JCP; Funding Acquisition,DLB and JCP.

## Conflict of Interests

There are no conflicts to declare.

1

## Supporting information

As a service to our authors and readers, this journal provides supporting information supplied by the authors. Such materials are peer reviewed and may be re‐organized for online delivery, but are not copy‐edited or typeset. Technical support issues arising from supporting information (other than missing files) should be addressed to the authors.

Supporting Information

## Data Availability

The data that support the findings of this study are available in the supplementary material of this article.
