# Supplementary material for: On the Valorisation of Chitin‐Derived Furans by Milling
Source: ChemSusChem. 2024 Oct 30;18(3):e202401584. doi: 10.1002/cssc.202401584 (PMC11790004; doi:10.1002/cssc.202401584)
Supplement: Supplementary file 1 — Supporting Information [file CSSC-18-e202401584-s001.pdf]

ChemSusChem

Supporting Information

On the Valorisation of Chitin-Derived Furans by Milling

Renan Rodini Mattioli, Camila Souza Santos, Bruna Butke de Souza,
Pedro Dominguez Branco, Robert R. A. Bolt, Sarah E. Raby-Buck, Tadeu Luiz Gomes Cabral,
Claudio F. Tormena, Duncan L. Browne,* and Julio C. Pastre*

Supporting Information for

On the Valorisation of Chitin-Derived Furans by Milling

Renan Rodini Mattioli[a,b], Camila Souza Santos[a], Bruna Butke de Souza[a], Pedro Dominguez Branco[a], Robert R. A. Bolt[b], Sarah E. Raby-Buck[b], Tadeu Luiz Gomes Cabral[a], Claudio F. Tormena[a], Duncan L. Browne*[,b], Julio C. Pastre*[,a]

[a] Dr. R. R. Mattioli, Dr. C. S. Santos, B. B. de Souza, P. D. Branco, T. L. G. Cabral, Prof. Dr. C. F. Tormena, Prof. Dr. J. C. Pastre
Institute of Chemistry
State University of Campinas (UNICAMP)
13083-970, Campinas, SP, Brazil
E-mail: jpastre@unicamp.br

[b] Dr. R. R. Mattioli, Dr. R. R. A. Bolt, S. E. Raby-Buck, Prof. Dr. D. L. Browne
Department of Pharmaceutical and Biological Chemistry, School of Pharmacy
University College London (UCL)
29-39 Brunswick Square, WC1N 1AX London, UK
E-mail: duncan.browne@ucl.ac.uk

Table of Contents

General Information **S4**
Optimisation of Diels–Alder reaction of **3A5AF** in milling **S5**
Study of Diels–Alder reaction of **3A5F** in solution **S10**
Study of aromatisation of hydrazone **3** in solution **S11**
Study of aromatisation of hydrazone **3** under microwave heating **S12**
Optimisation of aromatisation of hydrazone **3** in milling **S13**
Stability test of hydrazone **3** at anhydride conditions in milling **S14**
Procedure A: Synthesis of 3-acetamido-5-acetylfuran (**3A5AF**) **S15**
Procedure B: Synthesis of Diels–Alder adducts (**1a**–**1l**) **S15**
Procedure C: Synthesis of 3-acetamido-5-furfural (**3A5F**) **S16**
Procedure D: Synthesis of hydrazone **2** **S16**
Procedure E: Synthesis of hydrazone **3** **S16**
Procedure F: Synthesis of phthalimides at 50 °C (**3a**–**3l**) **S17**
Procedure G: Synthesis of phthalimides at room temperature (**3a**–**3l**) **S17**
Procedure H: Synthesis of phthalimide **4** **S18**
Procedure I: Synthesis of phthalimide **5** **S18**
Procedure J: Synthesis of phthalimide **6** **S18**
Procedure K: Synthesis of *N*-substituted maleimides (**7b**–**7p**) **S19**
Computational Studies **S20**
Structural Characterisation **S37**
NMR Spectra **S63**
References **S164**

List of Figures

Figure S1. The optimised molecular geometries of the dienes **A**, **B**, **C**, **D** and **E** employed in the Diels–Alder reaction **S26**
Figure S2. The optimised molecular geometry of the dienophiles **F**, **G** and **H** employed in the Diels–Alder reaction **S27**
Figure S3. Assigned numerical values to each carbon within the principal structure of dienes and maleimide **S28**
Figure S4. ^{1}H NMR Spectrum (400 MHz, DMSO-*d*$_6$) for 3-acetamido-5-acetylfuran (**3A5AF**) **S63**
Figure S5. ^{13}C NMR Spectrum (100 MHz, DMSO-*d*$_6$) for 3-acetamido-5-acetylfuran (**3A5AF**) **S64**
Figure S6. ^{1}H NMR Spectrum (400 MHz, DMSO-*d*$_6$) for Diels–Alder adduct **1a** **S65**
Figure S7. ^{13}C NMR Spectrum (100 MHz, DMSO-*d*$_6$) for Diels–Alder adduct **1a** **S66**
Figure S8. ^{1}H NMR Spectrum (400 MHz, DMSO-*d*$_6$) for Diels–Alder adduct **1b** **S67**
Figure S9. ^{13}C NMR Spectrum (100 MHz, DMSO-*d*$_6$) for Diels–Alder adduct **1b** **S68**
Figure S10. ^{1}H NMR Spectrum (400 MHz, DMSO-*d*$_6$) for Diels–Alder adduct **1c** **S69**
Figure S11. ^{13}C NMR Spectrum (100 MHz, DMSO-*d*$_6$) for Diels–Alder adduct **1c** **S70**
Figure S12. ^{1}H NMR Spectrum (400 MHz, DMSO-*d*$_6$) for Diels–Alder adduct **1d** **S71**
Figure S13. ^{19}F NMR Spectrum (376 MHz, DMSO-*d*$_6$) for Diels–Alder adduct **1d** **S72**
Figure S14. ^{13}C NMR Spectrum (100 MHz, DMSO-*d*$_6$) for Diels–Alder adduct **1d** **S73**
Figure S15. ^{1}H NMR Spectrum (400 MHz, DMSO-*d*$_6$) for Diels–Alder adduct **1e** **S74**
Figure S16. ^{13}C NMR Spectrum (100 MHz, DMSO-*d*$_6$) for Diels–Alder adduct **1e** **S75**
Figure S17. ^{1}H NMR Spectrum (400 MHz, DMSO-*d*$_6$) for Diels–Alder adduct **1f** **S76**
Figure S18. ^{13}C NMR Spectrum (100 MHz, DMSO-*d*$_6$) for Diels–Alder adduct **1f** **S77**
Figure S19. ^{1}H NMR Spectrum (400 MHz, DMSO-*d*$_6$) for Diels–Alder adduct **1g** **S78**
Figure S20. ^{13}C NMR Spectrum (100 MHz, DMSO-*d*$_6$) for Diels–Alder adduct **1g** **S79**
Figure S21. ^{1}H NMR Spectrum (400 MHz, DMSO-*d*$_6$) for Diels–Alder adduct **1h** **S80**
Figure S22. ^{19}F NMR Spectrum (376 MHz, DMSO-*d*$_6$) for Diels–Alder adduct **1h** **S81**
Figure S23. ^{13}C NMR Spectrum (100 MHz, DMSO-*d*$_6$) for Diels–Alder adduct **1h** **S82**
Figure S24. ^{1}H NMR Spectrum (400 MHz, DMSO-*d*$_6$) for Diels–Alder adduct **1i** **S83**
Figure S25. ^{13}C NMR Spectrum (100 MHz, DMSO-*d*$_6$) for Diels–Alder adduct **1i** **S84**
Figure S26. ^{1}H NMR Spectrum (400 MHz, DMSO-*d*$_6$) for Diels–Alder adduct **1j** **S85**
Figure S27. ^{13}C NMR Spectrum (100 MHz, DMSO-*d*$_6$) for Diels–Alder adduct **1j** **S86**
Figure S28. ^{1}H NMR Spectrum (400 MHz, DMSO-*d*$_6$) for Diels–Alder adduct **1k** **S87**
Figure S29. ^{13}C NMR Spectrum (100 MHz, DMSO-*d*$_6$) for Diels–Alder adduct **1k** **S88**
Figure S30. ^{1}H NMR Spectrum (400 MHz, DMSO-*d*$_6$) for Diels–Alder adduct **1l** **S89**
Figure S31. ^{13}C NMR Spectrum (100 MHz, DMSO-*d*$_6$) for Diels–Alder adduct **1l** **S90**
Figure S32. ^{1}H NMR Spectrum (400 MHz, DMSO-*d*$_6$) for 3-acetamido-5-furfural (**3A5F**) **S91**
Figure S33. ^{13}C NMR Spectrum (100 MHz, DMSO-*d*$_6$) for 3-acetamido-5-furfural (**3A5F**) **S92**
Figure S34. ^{1}H NMR Spectrum (400 MHz, DMSO-*d*$_6$) for hydrazone **2** **S93**
Figure S35. ^{13}C NMR Spectrum (100 MHz, DMSO-*d*$_6$) for hydrazone **2** **S94**
Figure S36. DEPT-135 NMR Spectrum (400 MHz, DMSO-*d*$_6$) for hydrazone **2** **S95**
Figure S37. HSQC NMR Spectrum (400 MHz, DMSO-*d*$_6$) for hydrazone **2** **S96**
Figure S38. HMBC NMR Spectrum (400 MHz, DMSO-*d*$_6$) for hydrazone **2** **S97**
Figure S39. NOESY NMR Spectrum (400 MHz, DMSO-*d*$_6$) for hydrazone **2** **S98**
Figure S40. ^{1}H NMR Spectrum (400 MHz, DMSO-*d*$_6$) for hydrazone **3** **S99**
Figure S41. ^{13}C NMR Spectrum (100 MHz, DMSO-*d*$_6$) for hydrazone **3** **S100**
Figure S42. ^{1}H NMR Spectrum (400 MHz, DMSO-*d*$_6$) for phthalimide **3a** **S101**
Figure S43. ^{13}C NMR Spectrum (100 MHz, DMSO-*d*$_6$) for phthalimide **3a** **S102**
Figure S44. ^{1}H NMR Spectrum (400 MHz, DMSO-*d*$_6$) for phthalimide **3b** **S103**

Figure S45. ^{13}C NMR Spectrum (100 MHz, DMSO-d_6) for phthalimide **3b** **S104**
Figure S46. ^{1}H NMR Spectrum (400 MHz, DMSO-d_6) for phthalimide **3c** **S105**
Figure S47. ^{13}C NMR Spectrum (100 MHz, DMSO-d_6) for phthalimide **3c** **S106**
Figure S48. ^{1}H NMR Spectrum (400 MHz, DMSO-d_6) for phthalimide **3d** **S107**
Figure S49. ^{13}C NMR Spectrum (100 MHz, DMSO-d_6) for phthalimide **3d** **S108**
Figure S50. ^{1}H NMR Spectrum (400 MHz, DMSO-d_6) for phthalimide **3e** **S109**
Figure S51. ^{13}C NMR Spectrum (100 MHz, DMSO-d_6) for phthalimide **3e** **S110**
Figure S52. ^{1}H NMR Spectrum (400 MHz, DMSO-d_6) for phthalimide **3f** **S111**
Figure S53. ^{13}C NMR Spectrum (100 MHz, DMSO-d_6) for phthalimide **3f** **S112**
Figure S54. ^{1}H NMR Spectrum (400 MHz, DMSO-d_6) for phthalimide **3g** **S113**
Figure S55. ^{13}C NMR Spectrum (100 MHz, DMSO-d_6) for phthalimide **3g** **S114**
Figure S56. ^{1}H NMR Spectrum (400 MHz, DMSO-d_6) for phthalimide **3h** **S115**
Figure S57. ^{13}C NMR Spectrum (100 MHz, DMSO-d_6) for phthalimide **3h** **S116**
Figure S58. ^{1}H NMR Spectrum (400 MHz, DMSO-d_6) for phthalimide **3i** **S117**
Figure S59. ^{13}C NMR Spectrum (100 MHz, DMSO-d_6) for phthalimide **3i** **S118**
Figure S60. ^{1}H NMR Spectrum (400 MHz, DMSO-d_6) for phthalimide **3j** **S119**
Figure S61. ^{13}C NMR Spectrum (100 MHz, DMSO-d_6) for phthalimide **3j** **S120**
Figure S62. ^{1}H NMR Spectrum (400 MHz, DMSO-d_6) for phthalimide **3k** **S121**
Figure S63. ^{19}F NMR Spectrum (376 MHz, DMSO-d_6) for phthalimide **3k** **S122**
Figure S64. ^{13}C NMR Spectrum (100 MHz, DMSO-d_6) for phthalimide **3k** **S123**
Figure S65. ^{1}H NMR Spectrum (400 MHz, DMSO-d_6) for phthalimide **3l** **S124**
Figure S66. ^{13}C NMR Spectrum (100 MHz, DMSO-d_6) for phthalimide **3l** **S125**
Figure S67. ^{1}H NMR Spectrum (400 MHz, DMSO-d_6) for phthalimide **4** **S126**
Figure S68. ^{13}C NMR Spectrum (100 MHz, DMSO-d_6) for phthalimide **4** **S127**
Figure S69. ^{1}H NMR Spectrum (400 MHz, DMSO-d_6) for phthalimide **5** **S128**
Figure S70. ^{13}C NMR Spectrum (100 MHz, DMSO-d_6) for phthalimide **5** **S129**
Figure S71. ^{1}H NMR Spectrum (400 MHz, DMSO-d_6) for phthalimide **6** **S130**
Figure S72. ^{13}C NMR Spectrum (100 MHz, DMSO-d_6) for phthalimide **6** **S131**
Figure S73. ^{1}H NMR Spectrum (400 MHz, $CDCl_3$) for *N*-(4-nitrophenyl)maleimide (**7b**) **S132**
Figure S74. ^{13}C NMR Spectrum (100 MHz, $CDCl_3$) for *N*-(4-nitrophenyl)maleimide (**7b**) **S133**
Figure S75. ^{1}H NMR Spectrum (400 MHz, $CDCl_3$) for *N*-(4-cyanophenyl)maleimide (**7c**) **S134**
Figure S76. ^{13}C NMR Spectrum (100 MHz, $CDCl_3$) for *N*-(4-cyanophenyl)maleimide (**7c**) **S135**
Figure S77. ^{1}H NMR Spectrum (400 MHz, $CDCl_3$) for *N*-(4-(trifluoromethyl)phenyl)maleimide (**7d**) **S136**
Figure S78. ^{19}F NMR Spectrum (376 MHz, $CDCl_3$) for *N*-(4-(trifluoromethyl)phenyl)maleimide (**7d**) **S137**
Figure S79. ^{13}C NMR Spectrum (100 MHz, $CDCl_3$) for *N*-(4-(trifluoromethyl)phenyl)maleimide (**7d**) **S138**
Figure S80. ^{1}H NMR Spectrum (400 MHz, $CDCl_3$) for *N*-(4-iodophenyl)maleimide (**7e**) **S139**
Figure S81. ^{13}C NMR Spectrum (100 MHz, $CDCl_3$) for *N*-(4-iodophenyl)maleimide (**7e**) **S140**
Figure S82. ^{1}H NMR Spectrum (400 MHz, $CDCl_3$) for *N*-(4-bromophenyl)maleimide (**7f**) **S141**
Figure S83. ^{13}C NMR Spectrum (100 MHz, $CDCl_3$) for *N*-(4-bromophenyl)maleimide (**7f**) **S142**
Figure S84. ^{1}H NMR Spectrum (400 MHz, $CDCl_3$) for *N*-(4-chlorophenyl)maleimide (**7g**) **S143**
Figure S85. ^{13}C NMR Spectrum (100 MHz, $CDCl_3$) for *N*-(4-chlorophenyl)maleimide (**7g**) **S144**
Figure S86. ^{1}H NMR Spectrum (400 MHz, $CDCl_3$) for *N*-(4-fluorophenyl)maleimide (**7h**) **S145**
Figure S87. ^{19}F NMR Spectrum (376 MHz, $CDCl_3$) for *N*-(4-fluorophenyl)maleimide (**7h**) **S146**
Figure S88. ^{13}C NMR Spectrum (100 MHz, $CDCl_3$) for *N*-(4-fluorophenyl)maleimide (**7h**) **S147**
Figure S89. ^{1}H NMR Spectrum (400 MHz, $CDCl_3$) for *N*-phenylmaleimide (**7i**) **S148**
Figure S90. ^{13}C NMR Spectrum (100 MHz, $CDCl_3$) for *N*-phenylmaleimide (**7i**) **S149**
Figure S91. ^{1}H NMR Spectrum (400 MHz, $CDCl_3$) for *N*-(4-methylphenyl)maleimide (**7j**) **S150**
Figure S92. ^{13}C NMR Spectrum (100 MHz, $CDCl_3$) for *N*-(4-methylphenyl)maleimide (**7j**) **S151**
Figure S93. ^{1}H NMR Spectrum (400 MHz, $CDCl_3$) for *N*-(4-methoxyphenyl)maleimide (**7k**) **S152**
Figure S94. ^{13}C NMR Spectrum (100 MHz, $CDCl_3$) for *N*-(4-methoxyphenyl)maleimide (**7k**) **S153**

Figure S95. ^{1}H NMR Spectrum (400 MHz, $CDCl_3$) for *N*-(4-(dimethylamino)phenyl)maleimide (**7l**) **S154**
Figure S96. ^{13}C NMR Spectrum (100 MHz, $CDCl_3$) for *N*-(4-(dimethylamino)phenyl)maleimide (**7l**)...... **S155**
Figure S97. ^{1}H NMR Spectrum (400 MHz, $CDCl_3$) for *N*-(naphthalene-1-yl)maleimide (**7m**)................. **S156**
Figure S98. ^{13}C NMR Spectrum (100 MHz, $CDCl_3$) for *N*-(naphthalene-1-yl)maleimide (**7m**)................ **S157**
Figure S99. ^{1}H NMR Spectrum (400 MHz, $CDCl_3$) for *N*-benzylmaleimide (**7n**) **S158**
Figure S100. ^{13}C NMR Spectrum (100 MHz, $CDCl_3$) for *N*-benzylmaleimide (**7n**) **S159**
Figure S101. ^{1}H NMR Spectrum (400 MHz, $CDCl_3$) for *N*-(2-(1*H*-indol-3-yl)ethyl)maleimide (**7o**).......... **S160**
Figure S102. ^{13}C NMR Spectrum (100 MHz, $CDCl_3$) for *N*-(2-(1*H*-indol-3-yl)ethyl)maleimide (**7o**)......... **S161**
Figure S103. ^{1}H NMR Spectrum (400 MHz, $CDCl_3$) for *N*-(1-adamantyl)maleimide (**7p**) **S162**
Figure S104. ^{13}C NMR Spectrum (100 MHz, $CDCl_3$) for *N*-(1-adamantyl)maleimide (**7p**) **S163**

List of Tables

Table S1. Screening of commercial dienophiles on Diels–Alder reaction in milling.................................. **S5**
Table S2. Screening of ball diameters on Diels–Alder reaction in milling **S6**
Table S3. Screening of maleimide equivalents on Diels–Alder reaction in milling.................................... **S6**
Table S4. Screening of grinding agents on Diels–Alder reaction in milling **S7**
Table S5. Screening of sodium chloride mass equivalents on Diels–Alder reaction in milling.................. **S7**
Table S6. Screening of liquid-assisted grinding agents on Diels–Alder reaction in milling **S8**
Table S7. Screening of Lewis acids on Diels–Alder reaction in milling **S8**
Table S8. Screening of temperature on Diels–Alder reaction in milling... **S9**
Table S9. Screening of time on Diels–Alder reaction in milling.. **S9**
Table S10. Study of Diels–Alder reaction in solution.. **S10**
Table S11. Screening of aromatisation of hydrazone **3** in solution **S11**
Table S12. Screening of aromatisation of hydrazone **3** under microwave heating................................. **S12**
Table S13. Screening of different conditions for aromatisation of hydrazone **3** in milling **S13**
Table S14. Stability test of hydrazone **3** at anhydride conditions **S14**
Table S15. Energy, HOMO energy and Energy Gap for the DFT-optimised structures of dienes **A**–**E**...... **S27**
Table S16. Energy and LUMO energy for the DFT-optimised structure of dienophiles **F**–**H** **S28**
Table S17. Molecular Orbital Coefficients (MOC) for the HOMO of each carbon in dienes **S28**
Table S18. Molecular Orbital Coefficients (MOC) for the LUMO of each carbon in maleimide **S33**

General Information

All commercial chemicals and solvents were purchased and used as received without further purification unless otherwise stated. The synthesis of 3-acetamido-5-acetylfuran (**3A5AF**) and 3-acetamido-5-furfural (**3A5F**) were performed by *N*-acetylglucosamine (NAG) dehydration on solution-phase. Diels–Alder adducts **1a**–**1l** and phthalimides **3a**–**3l** were synthesised on Retsch MM 400 mixer mill equipped with a homemade heating band system. The heating band system was fabricated as previously reported.[1] Except for procured *N*-ethylmaleimide, the *N*-substituted maleimides **7b**–**7p** were prepared from maleic anhydride and corresponding amines on solution-phase. All spectral data obtained for known compounds were in agreement with the ones reported in the literature. Thin layer chromatography (TLC) was carried out in aluminium sheets with silica gel 60 matrix and fluorescent indicator at 254 nm. Visualisation of spots were performed with UV irradiation at 254 nm and staining with potassium permanganate basic aqueous solution (substituted maleimides and phthalimides), vanillin ethanolic solution (bioderived furans) or ninhydrin ethanolic solution (Diels–Alder adducts). Purifications of compounds were performed with silica gel at 60 Å pore size and 40−63 µm particle size in Biotage Selekt flash chromatography system. Melting point (mp) was determined on OptiMelt MPA100 automated melting point system. Melting point values are reported as Celsius temperature values (°C) and only for *N*-substituted maleimides. Infrared (FTIR) was recorded on Agilent Cary 630 spectrometer using a diamond ATR sampling accessory. Wavenumbers of infrared bands are reported as $\tilde{\nu}$ values (cm^{-1}). Nuclear magnetic resonance (NMR) was recorded on Bruker Avance 400 MHz spectrometer operating at 400 MHz (^{1}H nuclei), 376 MHz (^{19}F nuclei) and 100 MHz (^{13}C nuclei) frequencies at 293 K. Chemical shifts are reported as δ values (ppm) referenced to the signal of chloroform (^{1}H: 7.26 ppm, ^{13}C: 77.2 ppm) or dimethylsulfoxide (^{1}H: 2.50 ppm, ^{13}C: 39.5 ppm). Multiplicities were given as "s" (singlet), "br s" (broad singlet), "d" (doublet), "dd" (doublet of doublets), "ddd" (doublet of doublets of doublets), "t" (triplet), "q" (quartet) and "m" (multiplet). Coupling constants (J) are reported in Hertz (Hz). Samples were prepared in deuterated solvent immediately before spectral acquisition. High-resolution mass spectrometry (HRMS) was carried out on Agilent Q-TOF 6545 mass spectrometer equipped with an electrospray source at 298 K for novel compounds. Molecular ions are reported as m/z values. Average mass errors are reported as $|\Delta m/z|$ values (ppm) related to the mass difference between calculated and experimental protonated molecular ion $[M+H]^{+}$ masses. Samples were prepared in HPLC gradient grade methanol immediately before experimental acquisition.

Optimisation of Diels–Alder reaction of 3A5AF in milling

To a 10 mL stainless steel milling jar were added a stainless-steel ball, 3-acetamido-5-acetylfuran (**3A5AF**, 16.7 mg, 0.10 mmol), maleimide, catalyst (when applicable), grinding agent and liquid-assisted grinding agent (when applicable). A band heater encased the jar and milled at 30 Hz. Different temperatures and times were performed. After this time the milling was stopped and the jar was cooled down to room temperature before any further manipulation when needed. The reaction mixture was removed from the jar into a conical flask with ethyl acetate (15 mL) and water (15 mL). The mixture was sonicated for 5 minutes, the organic layer was separated and the aqueous layer was extracted five times with ethyl acetate (15 mL). The organic layers were combined, dried over magnesium sulfate (10 g) and evaporated in rotary evaporator under reduced pressure to give the crude residue. To the resulting crude were added DMSO-d_6 (600 µL) and mesitylene (4.64 µL). The yield of the reaction and *exo*:*endo* ratio of reactions were determined by ^{1}H NMR analysis.

Table S1. Screening of commercial dienophiles on Diels–Alder reaction in milling

Entry	Dienophile	3A5AF[a]	Yield[b]	*Exo*:*endo*[c]	Balance[d]
**1**	Maleic anhydride	20	**-**	-	20
**2**	Maleimide	24	**46**	80:20 (4:1)	70
**3**	Acrylonitrile	63	**-**	-	63
**4**	Dimethyl but-2-yne dioate	33	**-**	-	33
**5**	Diphenylacetylene	53	**-**	-	53
**6**	*p*-Benzoquinone	58	**-**	-	58
**7**	Tetrachloro *p*-benzoquinone	63	**-**	-	63

Conditions: 3-Acetamido-5-acetylfuran (**3A5AF**, 16.7 mg, 0.10 mmol), dienophile (0.10 mmol), NaCl (2.0 mass equiv.), using 10 mm diameter milling ball at room temperature for 90 minutes. [a]NMR residual **3A5AF** measured in percentage (%). [b]NMR yield of sum of *exo* and *endo* products measured in percentage (%). [c]*Exo*:*endo* ratio of individual NMR yields. [d]Sum of individual NMR yields from *exo*/*endo* products and residual **3A5AF** measured in percentage (%).

Table S2. Screening of ball diameters on Diels–Alder reaction in milling

Entry	Ball diameter (mm)	3A5AF[a]	Yield[b]	*Exo*:*endo*[c]	Balance[d]
**1**	4.0	38	**32**	61:39 (2:1)	70
**2**	5.0	28	**40**	78:22 (3:1)	68
**3**	6.0	20	**40**	75:25 (3:1)	60
**4**	8.0	22	**43**	81:19 (4:1)	65
**5**	9.0	27	**42**	81:19 (4:1)	69
**6**	10.0	24	**46**	80:20 (4:1)	70
**7**	12.0	18	**27**	85:15 (6:1)	45
**8**	13.0	21	**21**	81:19 (4:1)	42
**9**	14.0	16	**25**	80:20 (4:1)	41
**10**	15.0	21	**20**	80:20 (4:1)	41
**11**	16.0	19	**34**	85:15 (6:1)	53

Conditions: 3-Acetamido-5-acetylfuran (**3A5AF**, 16.7 mg, 0.10 mmol), maleimide (**7a**, 9.7 mg, 0.10 mmol), NaCl (52.8 mg, 2.0 mass equiv.), at room temperature for 90 minutes. [a]NMR residual **3A5AF** measured in percentage (%). [b]NMR yield of sum of *exo* and *endo* products **1a** measured in percentage (%). [c]*Exo*:*endo* ratio of individual NMR yields. [d]Sum of individual NMR yields from *exo*/*endo* products **1a** and residual **3A5AF** measured in percentage (%).

Table S3. Screening of maleimide equivalents on Diels–Alder reaction in milling

Entry	Maleimide (equiv.)	3A5AF[a]	Yield[b]	*Exo*:*endo*[c]	Balance[d]
**1**	1.0	24	**46**	80:20 (4:1)	70
**2**	2.0	3	**57**	89:11 (9:1)	60
**3**	3.0	3	**54**	81:19 (4:1)	57
**4**	4.0	2	**52**	83:17 (5:1)	54

Conditions: 3-Acetamido-5-acetylfuran (**3A5AF**, 16.7 mg, 0.10 mmol), maleimide (**7a**), NaCl (2.0 mass equiv.), using 10 mm diameter milling ball at room temperature for 90 minutes. [a]NMR residual **3A5AF** measured in percentage (%). [b]NMR yield of sum of *exo* and *endo* products **1a** measured in percentage (%). [c]*Exo*:*endo* ratio of individual NMR yields. [d]Sum of individual NMR yields from *exo*/*endo* products **1a** and residual **3A5AF** measured in percentage (%).

Table S4. Screening of grinding agents on Diels–Alder reaction in milling

Entry	Grinding agent	3A5AF[a]	Yield[b]	*Exo*:*endo*[c]	Balance[d]
**1**	NaCl	3	**57**	89:11 (9:1)	60
**2**	LiBr	15	**50**	72:28 (3:1)	65
**3**	Na_2SO_4	5	**50**	80:20 (4:1)	55
**4**	Sand	24	**21**	81:19 (4:1)	45
**5**	Celite	3	**44**	84:16 (5:1)	47
**6**	Silica gel	10	**18**	72:28 (3:1)	28
**7**	-	43	**33**	70:30 (2:1)	76

Conditions: 3-Acetamido-5-acetylfuran (**3A5AF**, 16.7 mg, 0.10 mmol), maleimide (**7a**, 19.4 mg, 0.20 mmol), grinding agent (72.2 mg, 2.0 mass equiv.), using 10 mm diameter milling ball at room temperature for 90 minutes. [a]NMR residual **3A5AF** measured in percentage (%). [b]NMR yield of sum of *exo* and *endo* products **1a** measured in percentage (%). [c]*Exo*:*endo* ratio of individual NMR yields. [d]Sum of individual NMR yields from *exo*/*endo* products **1a** and residual **3A5AF** measured in percentage (%).

Table S5. Screening of sodium chloride mass equivalents on Diels–Alder reaction in milling

Entry	NaCl (mass equiv.)	3A5AF[a]	Yield[b]	*Exo*:*endo*[c]	Balance[d]
**1**	0.5	25	**45**	69:31 (2:1)	70
**2**	1.0	6	**60**	77:23 (3:1)	66
**3**	1.5	25	**40**	73:27 (3:1)	65
**4**	2.0	3	**57**	89:11 (9:1)	60
**5**	2.5	4	**63**	86:14 (5:1)	67
**6**	3.0	5	**56**	86:14 (5:1)	61
**7**	-	43	**33**	70:30 (2:1)	76

Conditions: 3-Acetamido-5-acetylfuran (**3A5AF**, 16.7 mg, 0.10 mmol), maleimide (**7a**, 19.4 mg, 0.20 mmol), NaCl, using 10 mm diameter milling ball at room temperature for 90 minutes. [a]NMR residual **3A5AF** measured in percentage (%). [b]NMR yield of sum of *exo* and *endo* products **1a** measured in percentage (%). [c]*Exo*:*endo* ratio of individual NMR yields. [d]Sum of individual NMR yields from *exo*/*endo* products **1a** and residual **3A5AF** measured in percentage (%).

Table S6. Screening of liquid-assisted grinding agents on Diels–Alder reaction in milling

Entry	Liquid-assisted grinding agent	3A5AF[a]	Yield[b]	*Exo*:*endo*[c]	Balance[d]
**1**	Toluene	10	**57**	77:23 (3:1)	67
**2**	Diethyl Carbonate	19	**48**	71:29 (2:1)	67
**3**	Ethanol	29	**43**	67:33 (2:1)	72
**4**	2,2,2-Trifluoroethanol	21	**46**	70:30 (2:1)	67
**5**	Water	38	**32**	69:31 (2:1)	70
**6**	*N*,*N*-Dimethylformamide	25	**38**	74:26 (3:1)	63
**7**	-	4	**63**	84:16 (5:1)	67

Conditions: 3-Acetamido-5-acetylfuran (**3A5AF**, 16.7 mg, 0.10 mmol), maleimide (**7a**, 19.4 mg, 0.20 mmol), NaCl (90.3 mg, 2.5 mass equiv.), liquid-assisted grinding agent (18.1 µL, 0.500 µL/mg), using 10 mm diameter milling ball at room temperature for 90 minutes. [a]NMR residual **3A5AF** measured in percentage (%). [b]NMR yield of sum of *exo* and *endo* products **1a** measured in percentage (%). [c]*Exo*:*endo* ratio of individual NMR yields. [d]Sum of individual NMR yields from *exo*/*endo* products **1a** and residual **3A5AF** measured in percentage (%).

Table S7. Screening of Lewis acids on Diels–Alder reaction in milling

Entry	Lewis acid	3A5AF[a]	Yield[b]	*Exo*:*endo*[c]	Balance[d]
**1**	KOTf	6	**64**	91:9 (10:1)	70
**2**	AgOTf	5	**70**	85:15 (6:1)	75
**3**	LiCl	13	**63**	73:27 (3:1)	76
**4**	$MgOTf_2$	29	**38**	71:29 (2:1)	67
**5**	$CuOTf_2$	17	**29**	55:45 (1:1)	46
**6**	$ZnOTf_2$	36	**42**	69:31 (2:1)	78
**7**	$LaOTf_3$	4	**66**	85:15 (6:1)	70
**8**	$GdOTf_3$	12	**57**	81:19 (4:1)	69
**9**	$BiOTf_3$	7	**33**	70:30 (2:1)	40
**10**	$AlCl_3$	41	**25**	60:40 (2:1)	66
**11**	$FeCl_3$	10	**36**	78:22 (4:1)	46
**12**	$ZrCl_4$	9	**24**	63:37 (2:1)	33
**13**	-	4	**63**	84:16 (5:1)	67

Conditions: 3-Acetamido-5-acetylfuran (**3A5AF**, 16.7 mg, 0.10 mmol), maleimide (**7a**, 19.4 mg, 0.20 mmol), NaCl (2.5 mass equiv.), Lewis acid (10 mol%), using 10 mm diameter milling ball at room temperature for 90 minutes. [a]NMR residual **3A5AF** measured in percentage (%). [b]NMR yield of sum of *exo* and *endo* products **1a** measured in percentage (%). [c]*Exo*:*endo* ratio of individual NMR yields. [d]Sum of individual NMR yields from *exo*/*endo* products **1a** and residual **3A5AF** measured in percentage (%).

Table S8. Screening of temperature on Diels–Alder reaction in milling

Entry	Temperature (°C)	3A5AF[a]	Yield[b]	*Exo*:*endo*[c]	Balance[d]
**1**	rt	4	**63**	84:16 (5:1)	67
**2**	40	4	**57**	88:12 (7:1)	61
**3**[e]	40	8	**58**	78:22 (3:1)	66
**4**	50	2	**62**	94:6 (15:1)	64
**5**[e]	50	2	**60**	92:8 (11:1)	62
**6**	60	1	**52**	> 95:5 (> 19:1)	53
**7**	70	3	**52**	> 95:5 (> 19:1)	55

Conditions: 3-Acetamido-5-acetylfuran (**3A5AF**, 16.7 mg, 0.10 mmol), maleimide (**7a**, 19.4 mg, 0.20 mmol), NaCl (2.5 mass equiv.), using 10 mm diameter milling ball at indicated temperature for 90 minutes. [a]NMR residual **3A5AF** measured in percentage (%). [b]NMR yield of sum of *exo* and *endo* products **1a** measured in percentage (%). [c]*Exo*:*endo* ratio of individual NMR yields. [d]Sum of individual NMR yields from *exo*/*endo* products **1a** and residual **3A5AF** measured in percentage (%). [e]Addition of AgOTf (10 mol%) as Lewis acid.

Table S9. Screening of time on Diels–Alder reaction in milling

Entry	Time (min)	3A5AF[a]	Yield[b]	*Exo*:*endo*[c]	Balance[d]
**1**	15	26	**49**	71:29 (3:1)	75
**2**	30	8	**68**	79:21 (4:1)	76
**3**	60	2	**67 (62[e])**	90:10 (9:1)	69
**4**	90	2	**62**	94:6 (15:1)	64
**5**	120	3	**60**	83:17 (5:1)	63
**6**	150	4	**54**	82:18 (5:1)	58
**7**	180	5	**49**	80:20 (4:1)	54

Conditions: 3-Acetamido-5-acetylfuran (**3A5AF**, 16.7 mg, 0.10 mmol), maleimide (**7a**, 19.4 mg, 0.20 mmol), NaCl (2.5 mass equiv.), using 10 mm diameter milling ball at 50 °C for indicated time. [a]NMR residual **3A5AF** measured in percentage (%). [b]NMR yield of sum of *exo* and *endo* products **1a** measured in percentage (%). [c]*Exo*:*endo* ratio of individual NMR yields. [d]Sum of individual NMR yields from *exo*/*endo* products **1a** and residual **3A5AF** measured in percentage (%). [e]Isolated yield (see *Procedure B* section for more purification details and scope).

Study of Diels–Alder reaction of 3A5F in solution

To a 5 mL vial were added 3-acetamido-5-furfural (**3A5F**, 15.3 mg, 0.10 mmol, 1.0 equiv.), maleimide (**7a**, 10.7 mg, 0.11 mmol, 1.1 equiv.), indicated solvent (500 µL) and an appropriate magnetic stir bar. The vial was sealed and different temperatures were applied at 72 h. After this time the reaction was stopped and the vial was cooled down to room temperature before any further manipulation when needed. Afterwards, the reaction was evaporated in rotary evaporator under reduced pressure to give the crude residue. To the resulting crude were added DMSO-d_6 (600 µL) and mesitylene (4.64 µL). The reaction yields were determined by ^{1}H NMR analysis.

Table S10. Study of Diels–Alder reaction in solution

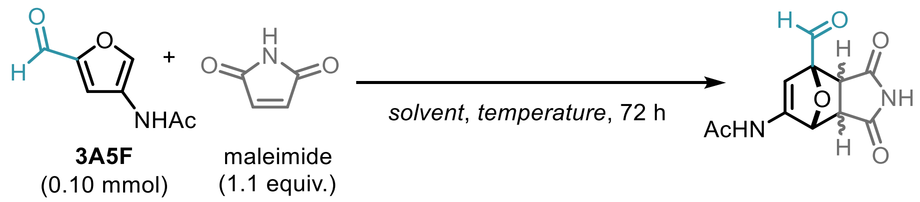

Entry	Solvent	Temperature (°C)	Yield[a]
**1**	MeCN	50	21
**2**	MeCN	rt	13
**3**	H_2O	50	20
**4**	H_2O	rt	7

Conditions: 3-Acetamido-5-furfural (**3A5F**, 15.3 mg, 0.10 mmol), maleimide (**7a**, 10.7 mg, 0.11 mmol) and solvent (500 µL, 0.2 M) at indicated temperature for 72 h. [a]NMR yield of sum of *exo* and *endo* products measured in percentage (%).

Study of aromatisation of hydrazone 3 in solution

To a 5 mL vial were added hydrazone **3** (25.0 mg, 0.10 mmol, 1.0 equiv.), maleimide (**7a**, 9.7 mg, 0.10 mmol, 1.0 equiv.), additive (10 mol%), indicated solvent (500 μL) and an appropriate magnetic stir bar. The vial was sealed and different temperatures and times were performed. After the indicated time the reaction was stopped and the vial was cooled down to room temperature before any further manipulation when needed. Afterwards, the reaction was evaporated in rotary evaporator under reduced pressure to give the crude residue. To the resulting crude were added DMSO-d_6 (600 μL) and mesitylene (4.64 μL). The reaction yields were determined by ^{1}H NMR analysis.

Table S11. Screening of aromatisation of hydrazone **3** in solution

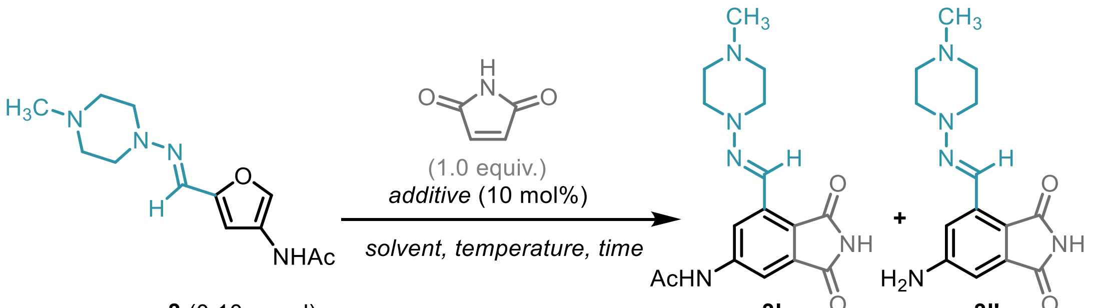

Entry	Solvent	Additive	Temperature (°C)	Time (h)	Yield of 3l/3l'[a]
1	EtOH	-	50	15	46/10
2	MeCN	-	50	15	45/9
3	DMSO	-	50	15	48/7
4	H_2O	-	50	15	24/11
5	AcOEt	-	50	15	25/9
6	Buffer pH 7	-	50	15	24/35
7	EtOH	-	rt	15	45/8
8	EtOH	-	70	1	48/9
9	EtOH	-	70	15	45/7
10	EtOH	MsOH	50	3	48/9
11	EtOH	IL[b]	50	3	47/8
12	EtOH	IL[b]	rt	4	49/7
13	EtOH	*p*-TsOH	50	3	42/12
14	EtOH	TfOH	50	3	34/8
15	EtOH	TFA	50	3	51/6
16	THF	Ac_2O (2.0 equiv.) and H_2SO_4	50	1	40/0
17	EtOH	AcOH	50	15	17/6
18	H_2O	TFA	50	15	20/43
19	EtOH	$MgSO_4$ (2.0 equiv)	50	15	45/10
20	EtOH	Amberlyst-15	50	15	22/8
21	MeCN	Ac_2O (2.0 equiv.)	50	2	50/0
22[c]	MeCN	Ac_2O (2.0 equiv.)	50	2	60/0

[a]NMR yield of **3l**/**3l'** products measured in percentage (%). [b]IL: *N*-methylimidazole·H_2SO_4. [c]Conditions: hydrazone **3** (30.0 mg, 0.12 mmol), maleimide (**7a**, 9.7 mg, 0.10 mmol), Ac_2O (18.9 μL, 0.20 mmol) and MeCN (500 μL).

Study of aromatisation of hydrazone 3 under microwave heating

To a 5 mL microwave vial were added hydrazone **3** (25.0 mg, 0.10 mmol, 1.0 equiv.), maleimide (**7a**, 9.7 mg, 0.10 mmol, 1.0 equiv.), additive (10 mol%), indicated solvent (500 µL) and an appropriate magnetic stir bar. The vial was sealed and different temperatures and times were performed. After the indicated time the reaction was stopped, the vial was cooled down to room temperature and the pressure was carefully released before any further manipulation. Afterwards, the reaction was evaporated in rotary evaporator under reduced pressure to give the crude residue. To the resulting crude were added DMSO-d_6 (600 µL) and mesitylene (4.64 µL). The reaction yields were determined by ^{1}H NMR analysis.

Table S12. Screening of aromatisation of hydrazone **3** under microwave heating

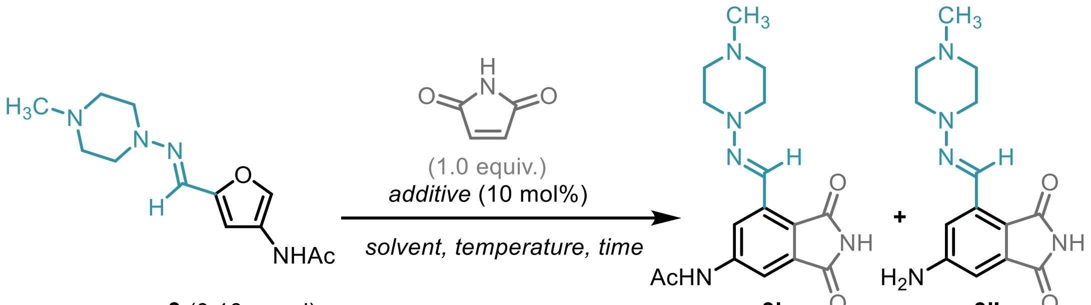

3 (0.10 mmol) **3l** **3l'**

Entry	Solvent	Additive	Temperature (°C)	Time (min)	Yield of 3l/3l'[a]
**1**	EtOH	-	120	15	50/5
**2**	EtOH	-	120	30	39/15
**3**	EtOH	-	100	15	16/15
**4**	EtOH	-	180	15	41/17
**5**	EtOH	-	180	5	42/17
**6**	EtOH	AcOH	120	15	55/8
**7**	EtOH	TFA	120	15	51/9
**8**	EtOH	$AlCl_3$	120	15	55/8
**9**	EtOH	Triethylamine	120	15	56/7
**10**	EtOH	$La(OTf)_3$	120	15	43/10
**11**	EtOH	MsOH	120	15	40/11
**12**	EtOH	*p*-TsOH	120	15	50/8
**13**	EtOH	H_3PO_4	120	15	37/9
**14**	EtOH	NaOH	120	15	0/0
**15**	EtOH	HCl	120	15	42/12
**16**	EtOH	$ZnCl_2$	120	15	38/8
**17**	EtOH	IL[b]	120	15	20/8
**18**	AcOEt	Ac_2O (2.0 equiv.)	120	15	23/0
**19**	THF	Ac_2O (2.0 equiv.)	120	15	46/0
**20**	MeCN	Ac_2O (2.0 equiv.)	120	15	55/0

[a]NMR yield of **3l**/**3l'** products measured in percentage (%). [b]IL: *N*-methylimidazole·H_2SO_4.

Optimisation of aromatisation of hydrazone 3 in milling

Optimised condition: To a 10 mL stainless steel milling jar were added a 10 mm diameter stainless steel ball, hydrazone **3** (15.0 mg, 0.06 mmol, 1.2 equiv.), *N*-(4-nitrophenyl)maleimide (**7b**, 10.9 mg, 0.05 mmol, 1.0 equiv.), sodium chloride (80.1 mg, 2.5 mass equiv.) and acetic anhydride (5.7 µL, 0.06 mmol, 1.2 equiv.). A band heater encased the jar and milled at 30 Hz for 60 min at 50 °C and room temperature. After this time the milling was stopped and the jar was cooled down to room temperature before any further manipulation when needed. The reaction mixture was removed from the jar into a conical flask with ethyl acetate (15 mL) and water (15 mL). The mixture was sonicated for 5 minutes, the organic layer was separated and the aqueous layer was extracted five times with ethyl acetate (15 mL). The organic layers were combined, dried over magnesium sulfate (10 g) and evaporated in rotary evaporator under reduced pressure to give the crude residue. To the resulting crude were added DMSO-d_6 (600 µL) and mesitylene (4.64 µL). The yield was determined by ^{1}H NMR analysis.

Table S13. Screening of different conditions for aromatisation of hydrazone **3** in milling

Entry	Variation from optimised conditions (equivalents)	Temperature (°C)	Yield of 3a/3a'[a]
**1**	*N*-(4-Nitrophenyl)maleimide (2.0), **3** (1.0), without Ac_2O	50	65/8
**2**	*N*-(4-Nitrophenyl)maleimide (2.0), **3** (1.0), without Ac_2O	rt	56/8
**3**	*N*-(4-Nitrophenyl)maleimide (2.0), **3** (1.0)	50	67/0
**4**	*N*-(4-Nitrophenyl)maleimide (2.0), **3** (1.0)	rt	58/0
**5**	*N*-(4-Nitrophenyl)maleimide (2.0), **3** (1.0), with NEt_3 (2.5)	50	66/0
**6**	*N*-(4-Nitrophenyl)maleimide (2.0), **3** (1.0), with NEt_3 (2.5)	rt	58/0
**7**	**3** (1.0)	50	64/0
**8**	**3** (1.0)	rt	62/0
**9**	$MgSO_4$ (2.5 mass equiv.) instead NaCl	50	58/9
**10**	$MgSO_4$ (2.5 mass equiv.) instead NaCl	rt	45/8
**11**	4 Å sieves (2.5 mass equiv.) instead NaCl	50	30/5
**12**	4 Å sieves (2.5 mass equiv.) instead NaCl	rt	35/5
**13**	None	50	69/0
**14**	None	rt	65/0
**15**	Ac_2O (0.5)	50	20/20
**16**	Ac_2O (0.5)	rt	43/6

[a]NMR yield of **3a/3a'** products measured in percentage (%).

Stability test of hydrazone 3 at anhydride conditions in milling

To a 10 mL stainless steel milling jar were added a 10 mm diameter stainless-steel ball, hydrazone **3** (12.5 mg, 0.05 mmol, 1.0 equiv.), acetic anhydride (5.7 µL, 0.06 mmol, 1.2 equiv.) and sodium chloride (46.6 mg, 2.5 mass equiv.). A band heater encased the jar and milled at 30 Hz for 60 min at 50 °C and room temperature. After this time the milling was stopped and the jar was cooled down to room temperature before any further manipulation when needed. The reaction mixture was removed from the jar into a conical flask with ethyl acetate (15 mL) and water (15 mL). The mixture was sonicated for 5 minutes, the organic layer was separated and the aqueous layer was extracted five times with ethyl acetate (15 mL). The organic layers were combined, dried over magnesium sulfate (10 g) and evaporated in rotary evaporator under reduced pressure to give the crude residue. To the resulting crude were added DMSO-d_6 (600 µL) and mesitylene (4.64 µL). The recovery yield was determined by ^{1}H NMR analysis.

Table S14. Stability test of hydrazone **3** at anhydride conditions

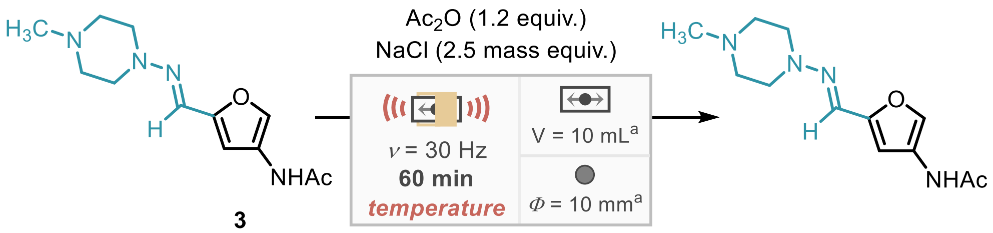

Entry	Temperature (°C)	Recovery yield of 3[b]
**1**	rt	90
**2**	50	35

Conditions: Hydrazone **3** (12.5 mg, 0.05 mmol), Ac_2O (5.7 µL, 0.06 mmol), NaCl (46.6 mg, 2.5 mass equiv.), using 10 mm diameter milling ball at 30 Hz and indicated temperature for 60 minutes. [a]Stainless steel materials. [b]NMR residual hydrazone **3** measured in percentage (%) after reaction.

Procedure A: Synthesis of 3-acetamido-5-acetylfuran (3A5AF)[2]

To a 20 mL microwave vial were added an appropriate magnetic stir bar, *N*-acetylglucosamine (1.0 g, 4.5 mmol, 1.0 equiv.), boric acid (280 mg, 4.5 mmol, 1.0 equiv.) and sodium chloride (1.0 g, 18 mmol, 4.0 equiv.). The flask was purged with nitrogen and then dimethylformamide (10 mL) was added. The flask was inserted in the microwave cavity and was heated at 220 °C for 15 minutes at 900 rpm stirring speed. The internal pressure reached 10 bar along the reaction. Upon completion of the reaction, the heating was stopped, the flask was cooled down to room temperature and the pressure was relieved before opening the microwave vial. After checking the formation of the product by TLC analysis using hexane/isopropanol 20% mixture as mobile phase (R_f = 0.20), the crude residue was transferred to a rounded-bottom flask and the residual solvent was removed in rotary evaporator under 10 mbar at 60 °C. The resulting dark brown oil was purified by flash chromatography using hexane/isopropanol 5–40% mixture (1.5 L) and silica gel (50 g). The fractions were combined, evaporated in rotary evaporator under reduced pressure, dried in a high vacuum system and stored at –5 °C. The product (dark yellow solid) was obtained in 29% yield (216 mg, 1.3 mmol).

Procedure B: Synthesis of Diels–Alder adducts (1a–1l)[3]–[6]

To a 10 mL stainless steel milling jar were added a 10 mm diameter stainless steel ball, 3-acetamido-5-acetylfuran (**3A5AF**, 33.4 mg, 0.20 mmol, 1.0 equiv.), maleimide or *N*-(4-phenyl)substituted maleimide (**7a**–**7l**, 0.40 mmol, 2.0 equiv.) and sodium chloride (2.5 mass equiv.). A band heater encased the jar and milled at 30 Hz and 50 °C for 60 minutes. After this time the milling was stopped and the jar was cooled down to room temperature before any further manipulation. The reaction mixture was removed from the jar into a conical flask with ethyl acetate (15 mL) and water (15 mL). The mixture was sonicated for 5 minutes, the organic layer was separated and the aqueous layer was extracted five times with ethyl acetate (15 mL). The organic layers were combined, dried over magnesium sulfate (10 g) and evaporated in rotary evaporator under reduced pressure to give the crude residue. Upon completion of the extraction the formation of the product was observed by TLC analysis using ethyl acetate as mobile phase. The crude residue was purified by flash chromatography using hexane/ethyl acetate and ethyl acetate/methanol mixtures (1.0 L) and silica gel (25 g). The fractions were combined, evaporated in rotary evaporator under reduced pressure, dried in a high vacuum system and stored at –5 °C. Hexane/dichloromethane 10% mixture (20 mL) was used to remove residual solvent before final dry in high vacuum system and storage. See *Structural Characterisation* section for more experimental details.

Procedure C: Synthesis of 3-acetamido-5-furfural (3A5F)[7]

To a stirred suspension of *N*-acetylglucosamine (4.0 g, 18 mmol, 1.0 equiv.) in pyridine (90 mL) with an appropriate magnetic stir bar, boric acid (1.7 g, 27 mmol, 1.5 equiv.), 4 Å molecular sieves (20 g, 5.0 mass equiv.) and triflic acid (1.6 mL, 18 mmol, 1.0 equiv.) were added and the reaction mixture was stirred at reflux under nitrogen atmosphere for 30 min. After this time, the reaction mixture was allowed to cool down to room temperature. Then, water (21 mL) and $NaIO_4$ (5.8 g, 27 mmol, 1.5 equiv.) were added and the reaction mixture was stirred at room temperature for 90 min. After checking the formation of the product by TLC analysis using hexane/ethyl acetate 50% mixture as mobile phase (R_f = 0.10), the reaction mixture was filtered and washed with ethyl acetate (100 mL) and the residual solvent was removed in rotary evaporator under reduced pressure. The resulting crude was purified by flash chromatography using hexane/ethyl acetate 0–80% mixture (2.0 L) and silica gel (100 g). The fractions were combined, evaporated in rotary evaporator under reduced pressure, dried in a high vacuum system and stored at –5 °C. The product (beige solid) was obtained in 36% yield (1.0 g, 6.5 mmol).

Procedure D: Synthesis of hydrazone 2

To a 10 mL vial were added 3-acetamido-5-acetylfuran (**3A5AF**, 167 mg, 1.0 mmol, 1.0 equiv.), 1-amino-4-methylpiperazine (364 µL, 3.0 mmol, 3.0 equiv.), trifluoroacetic acid (77 µL, 1.0 mmol, 1.0 equiv.), 4 Å molecular sieves (835 mg, 5.0 mass equiv.), toluene (4 mL) and an appropriate magnetic stirrer. The reaction was sealed and heated at 120 °C for 3 h. After checking the formation of the product by TLC analysis using dichloromethane/methanol 10% mixture as mobile phase (R_f = 0.20), the residual solvent was removed in rotary evaporator under reduced pressure and the resulting crude residue was purified by flash column chromatography using dichloromethane/methanol 0–20% mixture (1.0 L) and silica gel (25 g). The fractions were combined, evaporated in rotary evaporator under reduced pressure, dried in a high vacuum system and stored at –5 °C. The product (yellow solid) was obtained in 64% yield (168 mg, 0.64 mmol).

Procedure E: Synthesis of hydrazone 3

To a 25 mL rounded-bottom flask were added 3-acetamido-5-furfural (**3A5F**, 153 mg, 1.0 mmol, 1.0 equiv.), 1-amino-4-methylpiperazine (132 µL, 1.1 mmol, 1.1 equiv.), ethanol (5 mL) and an appropriate magnetic stirrer. The reaction was heated at 50 °C for 1 h. After checking the formation of the product by TLC analysis using dichloromethane/methanol 10% mixture as mobile phase (R_f = 0.25), the residual solvent was removed in rotary evaporator under reduced pressure and the resulting crude residue was purified by flash column chromatography using dichloromethane/methanol 0–15% mixture (1.0 L) and silica gel (25 g). The fractions

were combined, evaporated in rotary evaporator under reduced pressure, dried in a high vacuum system and stored at –5 °C. The product (yellow solid) was obtained in 81% yield (202 mg, 0.81 mmol). Alternatively, the reaction crude can also be purified by precipitation using a mixture of hexane (8 mL) and chloroform (2 mL) in 73% yield (182 mg, 0.73 mmol).

Procedure F: Synthesis of phthalimides at 50 °C (3a–3l)

To a 10 mL stainless steel milling jar were added a 10 mm diameter stainless steel ball, hydrazone **3** (15.0 mg, 0.06 mmol, 1.2 equiv.), maleimide or *N*-substituted maleimide (0.05 mmol, 1.0 equiv.), acetic anhydride (5.7 µL, 0.06 mmol, 1.2 equiv.) and sodium chloride (2.5 mass equiv.). A band heater encased on the jar at 30 Hz and 50 °C for 60 minutes. After this time the milling was stopped and the jar was cooled down to room temperature before any further manipulation. The reaction mixture was removed from the jar into a conical flask with ethyl acetate (15 mL) and water (15 mL). The mixture was sonicated for 5 minutes, the organic layer was separated and the aqueous layer was extracted five times with ethyl acetate (15 mL). The organic layers were combined, dried over magnesium sulfate (10 g) and evaporated in rotary evaporator under reduced pressure to give the crude residue. Alternatively, the reaction mixture can be removed from the jar with dichloromethane/methanol 20% mixture (50 mL), filtrated through a plug of celite (10 g) and the residual solvent removed in rotary evaporator under reduced pressure to give the crude residue. Upon completion of the extraction the formation of the product was observed by TLC analysis. The crude residue was purified by flash chromatography using dichloromethane/methanol 0–15% mixture (0.5 L) and silica gel (10 g). The fractions were combined, evaporated in rotary evaporator under reduced pressure, dried in a high vacuum system and stored at –5 °C. See *Structural Characterisation* section for scale-up and more experimental details.

Procedure G: Synthesis of phthalimides at room temperature (3a–3l)

To a 10 mL stainless steel milling jar were added a 10 mm diameter stainless steel ball, hydrazone **3** (15.0 mg, 0.06 mmol, 1.2 equiv.), maleimide or *N*-substituted maleimide (0.05 mmol, 1.0 equiv.), acetic anhydride (5.7 µL, 0.06 mmol, 1.2 equiv.) and sodium chloride (2.5 mass equiv.). A band heater encased on the jar at 30 Hz and room temperature for 60 minutes. After this time the milling was stopped. The reaction mixture was removed from the jar into a conical flask with ethyl acetate (15 mL) and water (15 mL). The mixture was sonicated for 5 minutes, the organic layer was separated and the aqueous layer was extracted five times with ethyl acetate (15 mL). The organic layers were combined, dried over magnesium sulfate (10 g) and evaporated in rotary evaporator under reduced pressure to give the crude residue. Alternatively, the reaction mixture can be removed from the jar with dichloromethane/methanol 20% mixture (50 mL), filtrated through a plug of celite (10 g) and the residual solvent removed in rotary evaporator under reduced pressure to

give the crude residue. Upon completion of the extraction the formation of the product was observed by TLC analysis. The crude residue was purified by flash chromatography using dichloromethane/methanol 0–15% mixture (0.5 L) and silica gel (10 g). The fractions were combined, evaporated in rotary evaporator under reduced pressure, dried in a high vacuum system and stored at –5 °C. See *Structural Characterisation* section for more experimental details.

Procedure H: Synthesis of phthalimide 4

To a solution of phthalimide **3b** (20.3 mg, 0.05 mmol, 1.0 equiv.) in methanol (500 µL) on ice bath, magnesium monoperoxyphthalate hexahydrate (61.5 mg, 0.13 mmol, 2.5 equiv.) was added and the reaction was stirred for 10 min. To the reaction mixture was added water (5 mL), the product was extracted four times with ethyl acetate (10 mL), dried over magnesium sulfate (10 g) and filtrated. The residual solvent was removed in rotary evaporator under reduced pressure and the resulting crude residue was purified by flash column chromatography using dichloromethane/methanol 0–15% mixture (0.5 L) and silica gel (10 g). The fractions were combined, evaporated in rotary evaporator under reduced pressure, dried in a high vacuum system and stored at –5 °C. The product (beige solid) was obtained in 66% yield (10.1 mg, 0.03 mmol).

Procedure I: Synthesis of phthalimide 5

To a solution of phthalimide **3b** (20.3 mg, 0.05 mmol, 1.0 equiv.) in acetone (1 mL), Amberlyst-15 (50.8 mg, 2.5 mass equiv.) was added and the reaction was stirred at 50 °C for 1 h. The Amberlyst-15 was removed by filtration, the residual solvent was removed in rotary evaporator under reduced pressure and the resulting crude residue was purified by flash column chromatography using dichloromethane/methanol 0–15% mixture (0.5 L) and silica gel (10 g). The fractions were combined, evaporated in rotary evaporator under reduced pressure, dried in a high vacuum system and stored at –5 °C. The product (beige solid) was obtained in 54% yield (8.3 mg, 0.03 mmol).

Procedure J: Synthesis of phthalimide 6

To a solution of phthalimide **3b** (20.3 mg, 0.05 mmol, 1.0 equiv.) in methanol (500 µL) on ice bath, magnesium monoperoxyphthalate hexahydrate (61.5 mg, 0.13 mmol, 2.5 mass equiv.) was added and the reaction was stirred for 10 min. Then, concentrated chloridric acid (50 µL) was added and the solution was heated at 50 °C for 15 h. Saturated sodium bicarbonate solution (5 mL) was added to the reaction mixture, extracted four times with dichloromethane (10 mL), dried over magnesium sulfate (10 g) and filtrated. The residual solvent was removed in rotary evaporator under reduced pressure and the resulting crude residue was purified by flash column chromatography using dichloromethane/methanol 0–15% mixture (0.5 L) and silica gel (10 g). The fractions were combined, evaporated in rotary evaporator under reduced pressure,

dried in a high vacuum system and stored at –5 °C. The product (yellow solid) was obtained in 40% yield (5.3 mg, 0.02 mmol).

Procedure K: Synthesis of *N*-substituted maleimides (7b–7p)[8]–[10]

To a 25 mL round bottom flask containing an appropriate magnetic stirrer, maleic anhydride (588 mg, 6.0 mmol, 1.2 equiv.) was solubilised in tetrahydrofuran (4 mL). Then, respective amine (5.0 mmol, 1.0 equiv.) was solubilised in tetrahydrofuran (8 mL) and added to reaction flask under stirring. The mixture was kept at room temperature for 16 h or until complete precipitation of the maleamic acid. Afterwards, the residual solvent was evaporated in rotary evaporator under reduced pressure. To the flask were added sodium acetate (164 mg, 2.0 mmol, 0.4 equiv.) and acetic anhydride (6 mL). A reflux condenser was coupled to the rounded bottom flask and the mixture was stirred under reflux for 2 hours. After this time, the formation of the product was observed by TLC analysis using hexane/ethyl acetate mixture as mobile phase, the reaction was cooled down to room temperature, water (30 mL) was added and the product was extracted three times with diethyl ether (30 mL). The organic layers were combined, dried over magnesium sulfate (20 g), filtered and evaporated in rotary evaporator under reduced pressure to give the crude residue. The crude residue was purified by flash chromatography using hexane/ethyl acetate mixture (1.0 L) and silica gel (50 g). The fractions were combined, evaporated in rotary evaporator under reduced pressure, dried in a high vacuum system and stored at room temperature. See *Structural Characterisation* section for more experimental details.

Computational Studies[11]–[18]

All molecular structures were generated using the GaussView 06[11] program and subsequently subjected to conformational sampling within the CREST package[12] at an extended tight-binding level implemented in the xTB program[13]. The generated structures were subjected to pre-optimisation using the Density Functional Theory (DFT) methods offered by the Gaussian 16 software package[14]. Various theoretical levels, employing the B3LYP method[15] and 6-31G, 6-311G, and 6-311++G(d,p) basis sets, were applied to pre-optimise the structures and eliminate conformations with identical contributions to the Boltzmann distribution. The final geometrical optimisation and frequency calculations were carried out using the PBE0 functional[16] with a 6-311++G(d,p) basis set, incorporating additional diffuse functions for 5d and 7f orbitals. Grimme's DFT dispersion correction and Becke–Johnson damping (DFT-D3(BJ))[17] were applied in all pre-optimisations and final optimisations. The final geometry optimisations and frequency calculations were performed in an ethanol solution applying the solvation model density (SMD) of Cramer-Truhlar and co-workers for the Self-Consistent Reaction Field (SCRF) method[18], as implemented by default in Gaussian 16. The energy gaps and molecular orbital coefficients were computed based on the most stable structures (characterised by lower free energy) for each compound at the same theoretical level employed during the final geometric optimisation. Moreover, energy gaps were also obtained at 323.15 and 393.15 K to mimic the experimental conditions.

1) Coordinates for the optimised structure of **A**:

C	–0.98588	–0.83562	–0.00000
C	0.23563	–0.11763	–0.00000
C	–0.09677	1.21659	0.00000
O	–1.43544	1.35343	0.00000
C	–1.97922	0.10496	–0.00000
N	1.48955	–0.71196	–0.00000
C	–3.41692	0.02400	–0.00000
C	2.67194	–0.04524	–0.00000
C	3.90228	–0.89859	0.00001
O	2.71900	1.18195	0.00001
O	–4.03856	–1.02674	–0.00001
H	–1.11705	–1.90826	–0.00001
H	0.48338	2.12251	0.00001
H	1.51055	–1.72215	–0.00001
H	–3.93430	0.99980	0.00000
H	4.49807	–0.65003	0.88252
H	3.68797	–1.96818	–0.00002
H	4.49810	–0.65000	–0.88248

2) Coordinates for the optimised structure of **B**:

C	–1.85017	–0.52555	0.00139
C	–3.20956	–0.09744	0.00223
C	–3.19527	1.26015	–0.15857
O	–1.90800	1.68352	–0.26087
C	–1.09570	0.59849	–0.16913
N	–4.28862	–0.96163	0.15080
C	0.33936	0.83043	–0.25755
C	–5.59251	–0.59318	0.16244
C	–6.58386	–1.70260	0.33734
O	–5.93398	0.58128	0.03727
C	0.82144	2.24225	–0.33702
N	1.06823	–0.22955	–0.29252
N	2.45051	–0.05907	–0.33550
C	3.01400	0.40660	0.93472
C	4.51488	0.55759	0.80820
N	5.14053	–0.69711	0.42439
C	4.56370	–1.16332	–0.82652
C	3.06528	–1.33082	–0.70095

C	6.57650	–0.54509	0.30957
H	–1.48677	–1.53536	0.12054
H	–3.95601	2.01770	–0.21547
H	–4.07040	–1.94284	0.25424
H	–7.25658	–1.70937	–0.52468
H	–6.11879	–2.68425	0.43864
H	–7.18631	–1.49516	1.22594
H	0.13403	2.84192	–0.93696
H	0.87223	2.70198	0.65595
H	1.81708	2.26985	–0.78340
H	2.58149	1.37040	1.20291
H	2.77305	–0.31146	1.73585
H	4.73774	1.35218	0.07074
H	4.92124	0.87894	1.77208
H	5.01146	–2.12665	–1.08858
H	4.78533	–0.45791	–1.65004
H	2.83403	–2.10260	0.05159
H	2.65020	–1.65212	–1.65883
H	7.03278	–1.50366	0.04620
H	6.99463	–0.21784	1.26590
H	6.86238	0.19418	–0.46082

3) Coordinates for the optimised structure of **C**:

C	–2.12024	–1.05729	0.00336
C	–3.28102	–0.22572	–0.01261
C	–2.83955	1.06233	–0.10657
O	–1.47556	1.06884	–0.14880
C	–1.04779	–0.21641	–0.08144
N	–4.57978	–0.71769	0.06107
C	0.36313	–0.50117	–0.09456
C	–5.70599	0.03478	0.05840
C	–6.99558	–0.72132	0.15906
O	–5.66526	1.26104	–0.02370
N	1.21974	0.45555	–0.16816
N	2.53953	0.20812	–0.24928
C	3.32588	1.30042	0.30715
C	4.78532	1.14699	–0.05380
N	5.28993	–0.14096	0.39420
C	4.53333	–1.19484	–0.25922
C	3.05496	–1.11288	0.06578

C	6.70485	–0.27139	0.10769
H	–2.09193	–2.13554	0.07046
H	–3.32416	2.02081	–0.15272
H	–4.67943	–1.72057	0.13142
H	0.62004	–1.55823	–0.04847
H	–7.55518	–0.34527	1.01960
H	–7.58997	–0.52008	–0.73666
H	–6.85485	–1.79819	0.26281
H	2.93049	2.23646	–0.09232
H	3.21771	1.32079	1.40305
H	4.91064	1.25888	–1.14761
H	5.35577	1.94518	0.42963
H	4.90944	–2.16694	0.07209
H	4.66313	–1.14451	–1.35700
H	2.87384	–1.34816	1.12797
H	2.53539	–1.85934	–0.54001
H	7.07108	–1.23613	0.46999
H	7.26135	0.52030	0.61705
H	6.92354	–0.20472	–0.97345

4) Coordinates for the optimised structure of **D**:

C	0.55424	1.08082	–0.01366
C	1.69424	0.22021	–0.01412
C	1.22115	–1.05844	–0.07168
O	–0.14368	–1.03172	–0.10613
C	–0.53969	0.26503	–0.07020
N	3.00548	0.68149	0.04016
C	–1.94292	0.58227	–0.08575
C	4.11267	–0.09865	0.04139
C	5.42162	0.62725	0.10880
O	4.04098	–1.32503	–0.01431
N	–2.82501	–0.35640	–0.12885
N	–4.12900	–0.06688	–0.21241
C	–4.97956	–1.13522	0.27122
C	–4.58615	1.27980	0.04581
H	0.55348	2.16086	0.02489
H	1.68079	–2.02991	–0.09481
H	3.13115	1.68297	0.08473
H	–2.18026	1.64486	–0.07294
H	5.97946	0.42829	–0.81092

H	5.30772	1.70517	0.23285
H	6.00282	0.22555	0.94269
H	–5.04239	–1.14392	1.36960
H	–5.98485	–1.01223	–0.13677
H	–4.57274	–2.08989	–0.06488
H	–4.18061	1.97648	–0.69339
H	–5.67331	1.29601	–0.03124
H	–4.29361	1.62385	1.05065

5) Coordinates for the optimised structure of **E**:

C	0.53218	–0.98291	0.00000
C	–0.63813	–0.17921	0.00000
C	–0.21392	1.12560	–0.00001
O	1.13545	1.16429	0.00000
C	1.58878	–0.11767	0.00000
N	–1.92972	–0.68886	0.00000
C	3.02742	–0.34770	0.00000
C	–3.06498	0.05362	0.00000
C	–4.34785	–0.71916	0.00000
O	–3.03239	1.28176	0.00000
O	3.45795	–1.49503	0.00000
C	3.92094	0.85032	0.00000
H	0.58375	–2.06214	0.00000
H	–0.72429	2.07242	–0.00001
H	–2.01828	–1.69534	0.00000
H	–4.92693	–0.43439	0.88267
H	–4.92692	–0.43442	–0.88269
H	–4.19852	–1.79989	0.00002
H	3.72073	1.46964	0.88025
H	4.96303	0.53109	0.00001
H	3.72073	1.46963	–0.88025

6) Coordinates for the optimised structure of **F**:

C	–0.66575	–1.26168	0.00004
C	0.66575	–1.26168	0.00006
C	1.13696	0.15454	–0.00002
N	0.00000	0.93739	–0.00003
C	–1.13696	0.15454	–0.00002
O	–2.27717	0.56071	–0.00004

O	2.27717	0.56071	0.00001
H	–1.35161	–2.09826	0.00007
H	1.35161	–2.09826	0.00011
H	0.00000	1.94908	–0.00006

7) Coordinates for the optimised structure of **G**:

C	0.49162	0.44809	–4.00713
C	–0.49162	–0.44809	–4.00713
C	0.84804	0.76748	–2.59978
N	–0.00000	–0.00000	–1.79177
C	–0.84804	–0.76748	–2.59978
O	–1.70273	–1.51847	–2.19937
O	1.70273	1.51847	–2.19937
C	–0.00000	–0.00000	–0.38286
C	0.00000	1.21325	0.30442
C	0.00849	1.21623	1.68775
C	0.00000	0.00000	2.35781
C	–0.00849	–1.21623	1.68775
C	–0.00000	–1.21325	0.30442
N	0.00000	0.00000	3.81404
O	0.05544	1.07217	4.39046
O	–0.05544	–1.07217	4.39046
H	1.00173	0.90732	–4.84299
H	–1.00173	–0.90732	–4.84299
H	–0.01762	2.14914	–0.23992
H	0.00640	2.14736	2.23979
H	–0.00640	–2.14736	2.23979
H	0.01762	–2.14914	–0.23992

8) Coordinates for the optimised structure of **H**:

C	3.39974	–0.64376	0.16788
C	3.39960	0.64531	–0.16073
C	1.98779	–1.10250	0.28763
N	1.18628	–0.00070	–0.00115
C	1.98759	1.10214	–0.28637
O	1.58516	2.20231	–0.58397
O	1.58576	–2.20324	0.58351
C	–0.23309	–0.00105	–0.00303
C	–0.92269	–0.83127	–0.87699

C	–2.31263	–0.83123	–0.86687
C	–3.02923	–0.00032	–0.00490
C	–2.31194	0.83297	0.85884
C	–0.92498	0.83165	0.87105
C	–4.52797	–0.00150	0.00759
H	4.23540	–1.30813	0.34189
H	4.23512	1.31077	–0.33127
H	–0.37535	–1.47001	–1.56224
H	–2.84911	–1.48395	–1.54929
H	–2.84888	1.48830	1.53891
H	–0.37730	1.47147	1.55507
H	–4.92372	1.01217	–0.10936
H	–4.93128	–0.62244	–0.79538
H	–4.90931	–0.38862	0.95879

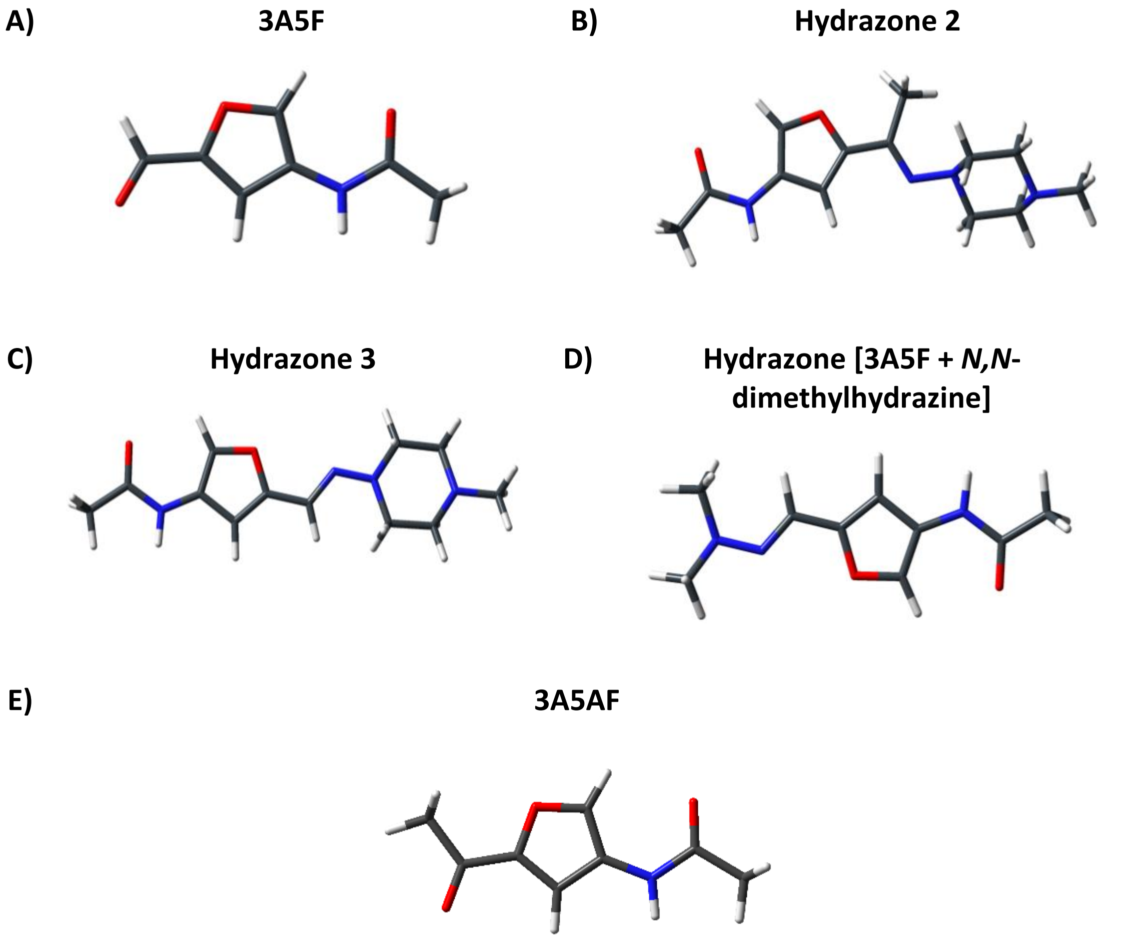

Figure S1. The optimised molecular geometries of the dienes **A**, **B**, **C**, **D** and **E** employed in the Diels–Alder reaction.

F) Maleimide **G) *N*-(4-Nitrophenyl)maleimide** **H) *N*-(4-Methylphenyl)maleimide**

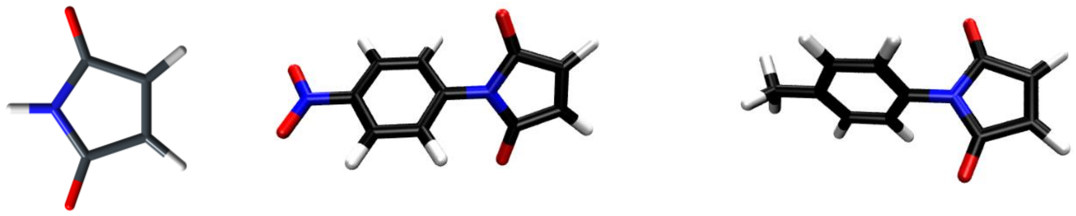

Figure S2. The optimised molecular geometry of the dienophiles **F**, **G** and **H** employed in the Diels–Alder reaction.

The energy gap was computed by subtracting the energy level of the Highest Occupied Molecular Orbital (HOMO) of the diene from that of the Lowest Unoccupied Molecular Orbital (LUMO) of the dienophile.

Table S15. Energy, HOMO energy and Energy Gap for the DFT-optimised structures of dienes **A**–**E**

Compound	Temperature (K)	Energy[a] (Hartree)	HOMO (Hartree)	Gap[b] (eV)
	298.15	−550.841544	−0.24762	−4.02
**A**	323.15	−550.845644	−0.24762	−4.02
	393.15	−550.857794	−0.24762	−4.02
	298.15	−875.838887	−0.21242	−3.06
**B**	323.15	−875.844706	−0.21242	−3.06
	393.15	−875.862274	−0.21242	−3.06
	298.15	−836.588249	−0.20678	−2.90
**C**	323.15	−836.593809	−0.20679	−2.91
	393.15	−836.610560	−0.20679	−2.91
	298.15	−664.727269	−0.20315	−2.81
**D**	323.15	−664.732219	−0.20315	−2.81
	393.15	−664.747138	−0.20248	−2.79
	298.15	−590.108119	−0.24479	−3.94
**E**	323.15	−590.112621	−0.24479	−3.94
	393.15	−590.125997	−0.24479	−3.94

**E**	323.15	−590.112621	−0.24479	−3.81[c]
	323.15	−590.112621	−0.24479	−3.93[d]

[a]The energy expressed is the sum of electronic and thermal free energies. [b]Energy gap = HOMO (dienes **A**–**E**) – LUMO (dienophile **F**). [c]Energy gap = HOMO (diene **E**) – LUMO (dienophile **G**). [d]Energy gap = HOMO (diene **E**) – LUMO (dienophile **H**).

Table S16. Energy and LUMO energy for the DFT-optimised structure of dienophiles **F**–**H**

Compound	Temperature (K)	Energy[a] (Hartree)	LUMO (Hartree)
**F**	298.15	−359.121742	−0.10003
	323.15	−359.124770	−0.10003
	393.15	−359.133624	−0.10003
**G**	323.15	−794.255733	−0.10485
**H**	323.15	−629.150607	−0.10052

[a]The energy expressed is the sum of electronic and thermal free energies.

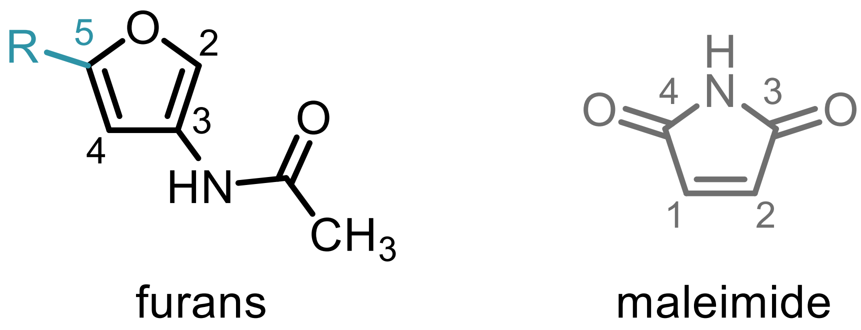

Figure S3. Assigned numerical values to each carbon within the principal structure of dienes and maleimide.

Table S17. Molecular Orbital Coefficients (MOC) for the HOMO of each carbon in dienes*

Carbon	Orbital	MOC of A	MOC of B	MOC of C	MOC of D	MOC of E
**C5**	1S	0.00000	0.00407	0.00037	0.00044	0.00000
	2S	0.00000	0.00663	0.00059	0.00071	0.00000
	2PX	0.00000	−0.01326	−0.00443	0.00400	0.00000
	2PY	0.00000	−0.00953	−0.00519	0.00267	0.00000

	2PZ	**–0.11242**	–0.07764	–0.08721	–0.08340	**–0.11552**
	3S	0.00000	–0.02107	–0.00146	–0.00187	0.00000
	3PX	0.00000	–0.02230	–0.00653	0.00588	0.00000
	3PY	0.00000	–0.01594	–0.00857	0.00444	0.00000
	3PZ	**–0.18813**	**–0.12815**	**–0.14362**	**–0.13714**	**–0.19272**
	4S	0.00000	–0.03936	–0.01676	–0.01198	0.00000
	4PX	0.00000	–0.01574	–0.02153	0.01591	0.00000
	4PY	0.00000	–0.01152	–0.00598	0.00092	0.00000
	4PZ	**–0.17561**	**–0.13089**	**–0.14762**	**–0.14316**	**–0.18180**
**C5**	5S	–0.00001	–0.08858	0.01280	0.02286	–0.00001
	5PX	–0.00001	**–0.18417**	0.00685	0.04116	0.00001
	5PY	0.00000	**–0.11636**	0.04615	–0.01034	0.00001
	5PZ	–0.01714	0.00924	–0.03255	–0.02453	–0.01184
	6D 0	0.00000	–0.00319	–0.00054	–0.00054	0.00000
	6D+1	0.00505	0.01432	0.01965	–0.02091	0.00429
	6D–1	–0.01097	0.00088	–0.00174	0.00215	–0.00989
	6D+2	0.00000	–0.00045	0.00083	0.00068	0.00000
	6D–2	0.00000	0.00211	0.00179	0.00111	0.00000
	1S	0.00000	0.00137	–0.00029	–0.00024	0.00000
	2S	0.00000	0.00230	–0.00047	–0.00039	0.00000
	2PX	0.00000	–0.00485	–0.00161	0.00138	0.00000
	2PY	0.00000	–0.00259	–0.00463	0.00261	0.00000
**C4**	2PZ	0.00107	–0.06493	–0.07936	–0.08218	–0.00605
	3S	0.00000	–0.00738	0.00138	0.00117	0.00000
	3PX	0.00000	–0.01005	–0.00258	0.00211	0.00000
	3PY	0.00000	–0.00641	–0.00757	0.00425	0.00000

	3PZ	0.00109	**–0.10419**	**–0.12612**	**–0.13060**	–0.01022
	4S	0.00000	–0.02344	–0.00134	0.00025	0.00000
	4PX	0.00000	0.02913	–0.00294	0.00319	0.00000
	4PY	0.00000	–0.00626	–0.01122	0.00688	0.00000
	4PZ	0.00210	**–0.11152**	**–0.14211**	**–0.14797**	–0.00942
	5S	0.00005	**–0.56680**	**0.10628**	–0.07551	0.00003
**C4**	5PX	0.00000	**0.21168**	**–0.01694**	**–0.01968**	0.00000
	5PY	0.00001	–0.01646	0.00918	0.01640	0.00000
	5PZ	–0.00327	–0.03391	–0.04073	–0.03993	–0.00452
	6D 0	0.00000	0.00161	0.00103	0.00074	0.00000
	6D+1	0.01995	–0.00963	–0.01298	0.01236	0.02009
	6D–1	0.00059	–0.00562	–0.00251	0.00233	0.00153
	6D+2	0.00000	0.00126	–0.00038	–0.00036	0.00000
	6D–2	0.00000	–0.00314	–0.00074	–0.00036	0.00000
	1S	0.00000	–0.00027	0.00009	0.00009	0.00000
	2S	0.00000	–0.00039	0.00015	0.00014	0.00000
	2PX	0.00000	0.00494	0.00166	–0.00132	0.00000
	2PY	0.00000	0.00396	0.00459	–0.00267	0.00000
	2PZ	**0.10986**	0.05423	0.06405	0.06108	**0.11093**
	3S	0.00000	0.00016	–0.00037	–0.00029	0.00000
**C3**	3PX	0.00000	0.00642	0.00280	–0.00220	0.00000
	3PY	0.00000	0.00733	0.00723	–0.00410	0.00000
	3PZ	**0.17932**	0.08821	**0.10364**	0.09879	**0.18081**
	4S	0.00000	0.02677	0.00527	0.00193	0.00000
	4PX	0.00000	0.02997	0.00161	–0.00223	0.00000
	4PY	0.00000	–0.00519	0.00927	–0.00619	0.00000

	4PZ	**0.18914**	0.09430	**0.11584**	**0.11058**	**0.19114**
	5S	–0.00002	**0.13080**	–0.02103	–0.01560	0.00002
	5PX	0.00001	0.07440	–0.00310	–0.01100	0.00001
	5PY	0.00001	**–0.20340**	0.03499	0.03135	0.00002
	5PZ	0.02452	0.03459	0.03116	0.03066	0.02035
**C3**	6D 0	0.00000	–0.00357	–0.00242	–0.00133	0.00000
	6D+1	–0.01663	–0.00624	–0.00076	0.00100	–0.01714
	6D–1	0.02530	0.01804	0.02275	–0.02270	0.02481
	6D+2	0.00000	–0.00194	–0.00136	–0.00077	0.00000
	6D–2	0.00000	0.00075	0.00060	0.00051	0.00000
	1S	0.00000	0.00041	0.00012	0.00013	0.00000
	2S	0.00000	0.00069	0.00020	0.00022	0.00000
	2PX	0.00000	0.00372	0.00320	–0.00260	0.00000
	2PY	0.00000	0.01458	0.00776	–0.00427	0.00000
	2PZ	**0.15669**	**0.10793**	**0.12504**	**0.12255**	**0.15993**
	3S	0.00000	–0.00290	–0.00068	–0.00085	0.00000
	3PX	0.00000	0.00606	0.00520	–0.00409	0.00000
	3PY	0.00000	0.02359	0.01259	–0.00694	0.00000
**C2**	3PZ	**0.25555**	**0.17457**	**0.20283**	**0.19846**	**0.26038**
	4S	0.00000	0.00912	0.00098	0.00145	0.00000
	4PX	0.00000	0.00382	0.00497	–0.00467	0.00000
	4PY	0.00000	0.01943	0.01730	–0.01026	0.00000
	4PZ	**0.24596**	**0.18323**	**0.20594**	**0.20429**	**0.25134**
	5S	0.00000	**0.28469**	–0.07015	0.03999	0.00004
	5PX	0.00000	–0.00123	–0.00457	0.00074	0.00000
	5PY	0.00000	–0.05342	0.02052	0.00515	0.00001

	5PZ	0.03403	0.04583	0.03855	0.04174	0.03768
	6D 0	0.00000	0.00095	0.00102	0.00061	0.00000
	6D+1	0.00248	–0.00018	–0.00424	0.00454	0.00367
**C2**	6D–1	–0.01502	–0.00620	–0.00684	0.00633	–0.01470
	6D+2	0.00000	0.00129	0.00020	0.00001	0.00000
	6D–2	0.00000	–0.00168	–0.00028	–0.00020	0.00000

*MOC values greater than 0.10000 are highlighted.

Table S18. Molecular Orbital Coefficients (MOC) for the LUMO of each carbon in maleimide*

Carbon	Orbital	MOC of maleimide
**C1**	1S	0.00000
	2S	0.00000
	2PX	0.00000
	2PY	0.00001
	2PZ	**0.12049**
	3S	0.00000
	3PX	0.00000
	3PY	0.00001
	3PZ	**0.19767**
	4S	0.00000
	4PX	0.00000
	4PY	0.00001
	4PZ	**0.26862**
	5S	0.00003
	5PX	0.00000
	5PY	0.00001
	5PZ	**0.14744**
	6D 0	0.00000
	6D+1	**–0.02196**
	6D–1	0.01489
	6D+2	0.00000
	6D–2	0.00000
**C2**	1S	0.00000

	2S	0.00000
	2PX	0.00000
	2PY	–0.00001
	2PZ	**–0.12049**
	3S	–0.00001
	3PX	0.00000
	3PY	–0.00002
	3PZ	**–0.19767**
	4S	–0.00001
	4PX	0.00000
**C2**	4PY	–0.00002
	4PZ	**–0.26862**
	5S	–0.00004
	5PX	0.00000
	5PY	–0.00001
	5PZ	**–0.14744**
	6D 0	0.00000
	6D+1	–0.02196
	6D–1	–0.01489
	6D+2	0.00000
	6D–2	0.00000
	1S	0.00000
	2S	0.00000
**C3**	2PX	0.00000
	2PY	0.00000
	2PZ	**–0.11794**

	3S	0.00001
	3PX	0.00000
	3PY	–0.00001
	3PZ	**–0.20144**
	4S	0.00002
	4PX	0.00000
	4PY	–0.00001
	4PZ	**–0.22899**
**C3**	5S	0.00004
	5PX	–0.00001
	5PY	–0.00001
	5PZ	0.00538
	6D 0	0.00000
	6D+1	0.02510
	6D–1	0.02127
	6D+2	0.00000
	6D–2	0.00000
	1S	0.00000
	2S	0.00000
	2PX	0.00000
	2PY	0.00000
**C4**	2PZ	**0.11794**
	3S	0.00000
	3PX	0.00000
	3PY	0.00001
	3PZ	**0.20144**

	4S	0.00000
	4PX	0.00000
	4PY	0.00001
	4PZ	**0.22899**
	5S	–0.00002
	5PX	0.00000
**C4**	5PY	0.00000
	5PZ	–0.00538
	6D 0	0.00000
	6D+1	0.02510
	6D–1	–0.02127
	6D+2	0.00000
	6D–2	0.00000

*MOC values greater than 0.10000 are highlighted.

Structural Characterisation

3-Acetamido-5-acetylfuran (**3A5AF**)

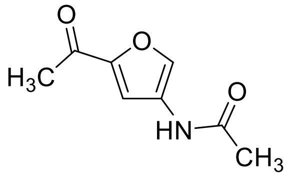

Prepared according to *Procedure A*, starting with *N*-acetylglucosamine (NAG, 1.0 g, 4.5 mmol). Purified by flash chromatography (1.5 L hexane/isopropanol 5–40%); R_f = 0.20 (hexane/isopropanol 20%); Yield = 29% (216 mg, 1.3 mmol); Dark yellow solid; FTIR (ATR) $\tilde{\nu}$ 3452, 3340, 3239, 3165, 3120, 3094, 3053, 1867, 1655, 1610, 1551, 1502, 1431, 1372, 1349, 1327, 1264, 1226, 1185, 1118, 1081, 1040, 1021, 991, 954, 924, 854, 768, 686, 663 cm^{-1}; ^{1}H NMR (DMSO-d_6, 400 MHz) δ 10.20 (s, 1H), δ 8.17 (s, 1H), δ 7.18 (s, 1H), δ 2.40 (s, 3H), δ 2.02 (s, 3H); ^{13}C NMR (DMSO-d_6, 100 MHz) δ 186.0, 167.7, 149.7, 135.2, 127.0, 111.0, 25.8, 22.8 ppm.

Diels–Alder adduct **1a**

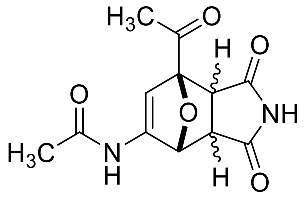

Prepared according to *Procedure B*, starting with 3-acetamido-5-acetylfuran (**3A5AF**, 33.4 mg, 0.20 mmol) and maleimide (**7a**, 38.8 mg, 0.40 mmol). Purified by flash chromatography (800 mL hexane/ethyl acetate 0–100%, then 200 mL ethyl acetate/methanol 0–10%); *Exo*:*endo* ratio = 9:1; R_f = 0.20 (ethyl acetate); Yield = 62% (32.7 mg, 0.12 mmol); Yellow solid; FTIR (ATR) $\tilde{\nu}$ 3526, 3243, 3176, 3060, 3004, 2930, 2777, 1774, 1703, 1633, 1536, 1420, 1353, 1305, 1245, 1185, 1155, 1085, 1040, 1021, 1006, 984, 956, 917, 861, 827, 772, 712, 656 cm^{-1}; ^{1}H NMR (DMSO-d_6, 400 MHz) δ 11.33 (br s, 0.90H), δ 11.02 (br s, 0.10H), δ 10.27 (s, 0.90H), δ 10.21 (s, 0.10H), δ 6.15 (s, 0.10H), δ 6.11 (s, 0.90H), δ 5.29 (d, J = 5.5 Hz, 0.10H), δ 5.17 (s, 0.90H), δ 3.77 (dd, J = 7.7, 5.5 Hz, 0.10H), δ 3.60 (d, J = 7.7 Hz, 0.10H), δ 3.38 (d, J = 6.5 Hz, 0.90H), δ 3.11 (d, J = 6.5 Hz, 0.90H), δ 2.33 (s, 0.30H), δ 2.25 (s, 2.70H), δ 1.98 (s, 2.70H), δ 1.92 (s, 0.30H); ^{13}C NMR (DMSO-d_6, 100 MHz) δ 202.5 (*endo*), 202.4 (*exo*), 176.8 (*exo*), 175.8 (*exo*), 175.1 (*endo*), 174.8 (*endo*), 170.4 (*endo*), 168.6 (*exo*), 144.7 (*exo*), 137.8 (*endo*), 110.1 (*exo*), 105.2 (*endo*), 94.5 (*exo*), 93.1 (*endo*), 79.9 (*exo*), 78.5 (*endo*), 53.7 (*exo*), 52.3 (*endo*), 50.0 (*exo*), 47.8 (*endo*), 26.9 (*exo*), 26.1 (*endo*), 23.2 (*exo*), 23.1 (*endo*) ppm.

Diels–Alder adduct **1b**

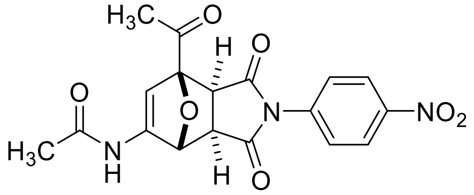

Prepared according to *Procedure B*, starting with 3-acetamido-5-acetylfuran (**3A5AF**, 33.4 mg, 0.20 mmol) and *N*-(4-nitrophenyl)maleimide (**7b**, 87.3 mg, 0.40 mmol). Purified by flash chromatography (800 mL hexane/ethyl acetate 0–100%, then 200 mL ethyl acetate/methanol 0–10%); *Exo*:*endo* ratio > 19:1; R_f = 0.20 (ethyl acetate); Yield = 77% (59.3 mg, 0.15 mmol); Pale yellow solid; FTIR (ATR) $\tilde{\nu}$ 3358, 3120, 3071, 2989, 1774, 1726, 1703, 1625, 1610, 1595, 1524, 1495, 1390, 1368, 1346, 1305, 1260, 1237, 1208, 1185, 1152, 1096, 1073, 1055, 1021, 1003, 956, 943, 921, 883, 854, 831, 798, 745, 719, 708, 686, 667 cm^{-1}; ^{1}H NMR (DMSO-*d*$_6$, 400 MHz) δ 10.38 (s, 1H), δ 8.37 (d, *J* = 8.9 Hz, 2H), δ 7.55 (d, *J* = 8.9 Hz, 2H), δ 6.22 (s, 1H), δ 5.34 (s, 1H), δ 3.68 (d, *J* = 6.6 Hz, 1H), δ 3.39 (d, *J* = 6.6 Hz, 1H), δ 2.32 (s, 3H), δ 2.01 (s, 3H); ^{13}C NMR (DMSO-*d*$_6$, 100 MHz) δ 202.3, 174.2, 173.1, 168.7, 146.8, 144.8, 137.2, 127.6, 124.5, 110.1, 94.9, 80.4, 52.8, 49.3, 27.1, 23.3 ppm; HRMS (ESI-TOF) *m/z* [M+H]$^+$ Calculated for $C_{18}H_{16}N_3O_7^+$ 386.0983, found 386.0984 (|Δ*m/z*|= 0.3 ppm).

Diels–Alder adduct **1c**

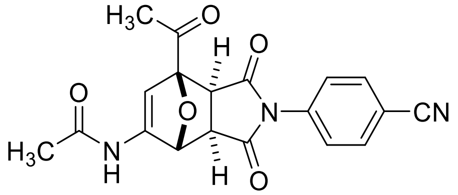

Prepared according to *Procedure B*, starting with 3-acetamido-5-acetylfuran (**3A5AF**, 33.4 mg, 0.20 mmol) and *N*-(4-cyanophenyl)maleimide (**7c**, 79.3 mg, 0.40 mmol). Purified by flash chromatography (800 mL hexane/ethyl acetate 0–100%, then 200 mL ethyl acetate/methanol 0–10%); *Exo*:*endo* ratio > 19:1; R_f = 0.20 (ethyl acetate); Yield = 73% (53.3 mg, 0.15 mmol); Pale yellow solid; FTIR (ATR) $\tilde{\nu}$ 3358, 3127, 3049, 2993, 2930, 2233, 1774, 1703, 1629, 1606, 1528, 1506, 1390, 1364, 1308, 1286, 1260, 1241, 1196, 1152, 1092, 1070, 1055, 1021, 1006, 958, 939, 924, 883, 850, 827, 794, 727, 704, 671 cm^{-1}; ^{1}H NMR (DMSO-*d*$_6$, 400 MHz) δ 10.38 (s, 1H), δ 8.00 (d, *J* = 8.5 Hz, 2H), δ 7.46 (d, *J* = 8.5 Hz, 2H), δ 6.21 (s, 1H), δ 5.32 (s, 1H), δ 3.66 (d, *J* = 6.6 Hz, 1H), δ 3.38 (d, *J* = 6.6 Hz, 1H), δ 2.31 (s, 3H), δ 2.01 (s, 3H); ^{13}C NMR (DMSO-*d*$_6$, 100 MHz) δ 202.3, 174.2, 173.1, 168.7, 144.8, 135.7, 133.3, 127.5, 118.3, 111.2, 110.1, 94.9, 80.4, 52.8, 49.2, 27.1, 23.3 ppm; HRMS (ESI-TOF) *m/z* [M+H]$^+$ Calculated for $C_{19}H_{16}N_3O_5^+$ 366.1084, found 366.1084 (|Δ*m/z*|= 0).

Diels–Alder adduct **1d**

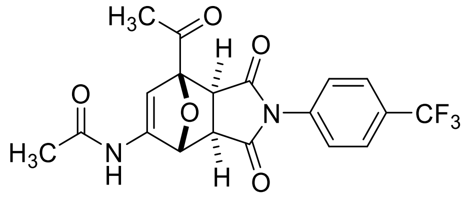

Prepared according to *Procedure B*, starting with 3-acetamido-5-acetylfuran (**3A5AF**, 33.4 mg, 0.20 mmol) and *N*-(4-(trifluoromethyl)phenyl)maleimide (**7d**, 96.5 mg, 0.40 mmol). Purified by flash chromatography (800 mL hexane/ethyl acetate 0–100%, then 200 mL ethyl acetate/methanol 0–10%); *Exo*:*endo* ratio > 19:1; R_f = 0.20 (ethyl acetate); Yield = 69% (56.4 mg, 0.14 mmol); Pale yellow solid; FTIR (ATR) $\tilde{\nu}$ 3343, 3060, 3004, 2960, 2930, 1782, 1711, 1633, 1618, 1532, 1521, 1416, 1375, 1323, 1260, 1170, 1126, 1066, 1021, 936, 883, 842, 827, 805, 783, 738, 727, 697, 671 cm^{-1}; ^{1}H NMR (DMSO-d_6, 400 MHz) δ 10.38 (s, 1H), δ 7.90 (d, J = 8.5 Hz, 2H), δ 7.48 (d, J = 8.5 Hz, 2H), δ 6.21 (s, 1H), δ 5.33 (s, 1H), δ 3.66 (d, J = 6.6 Hz, 1H), δ 3.38 (d, J = 6.6 Hz, 1H), δ 2.31 (s, 3H), δ 2.01 (s, 3H); ^{19}F NMR (DMSO-d_6, 376 MHz) δ –61.1 ppm; ^{13}C NMR (DMSO-d_6, 100 MHz) δ 202.3, 174.4, 173.3, 168.7, 144.8, 135.3, 129.3, 129.0, 128.7, 128.3, 127.9, 127.5, 126.3(4), 126.3(0), 126.2(7), 126.2(3), 125.2, 122.5, 119.8, 110.1, 94.9, 80.4, 52.8, 49.2, 27.0, 23.3 ppm; HRMS (ESI-TOF) *m/z* [M+H]$^+$ Calculated for $C_{19}H_{16}F_3N_2O_5^+$ 409.1006, found 409.1010 (|Δ*m/z*|= 1.0 ppm).

Diels–Alder adduct **1e**

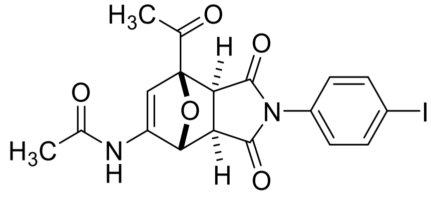

Prepared according to *Procedure B*, starting with 3-acetamido-5-acetylfuran (**3A5AF**, 33.4 mg, 0.20 mmol) and *N*-(4-iodophenyl)maleimide (**7e**, 119.6 mg, 0.40 mmol). Purified by flash chromatography (800 mL hexane/ethyl acetate 0–100%, then 200 mL ethyl acetate/methanol 0–10%); *Exo*:*endo* ratio > 19:1; R_f = 0.25 (ethyl acetate); Yield = 67% (62.8 mg, 0.13 mmol); Pale yellow solid; FTIR (ATR) $\tilde{\nu}$ 3362, 3232, 3150, 3056, 3008, 2967, 2926, 1778, 1696, 1636, 1524, 1483, 1390, 1364, 1331, 1301, 1271, 1237, 1204, 1182, 1144, 1070, 1055, 1029, 1010, 988, 962, 936, 917, 880, 854, 827, 798, 775, 734, 719, 708, 671 cm^{-1}; ^{1}H NMR (DMSO-d_6, 400 MHz) δ 10.35 (s, 1H), δ 7.87 (d, J = 8.3 Hz, 2H), δ 7.03 (d, J = 8.3 Hz, 2H), δ 6.20 (s, 1H), δ 5.31 (s, 1H), δ 3.60 (d, J = 6.5 Hz, 1H), δ 3.34 (d, J = 6.5 Hz, 1H), δ 2.29 (s, 3H), δ 2.01 (s, 3H); ^{13}C NMR (DMSO-d_6, 100 MHz) δ 202.3, 174.4, 173.2, 168.6, 144.7, 137.9, 131.4, 128.7, 110.1, 94.8, 94.6, 80.3, 52.7, 49.0, 26.9, 23.2 ppm; HRMS (ESI-TOF) *m/z* [M+H]$^+$ Calculated for $C_{18}H_{16}IN_2O_5^+$ 467.0098, found 467.0102 (|Δ*m/z*|= 0.9 ppm).

Diels–Alder adduct **1f**

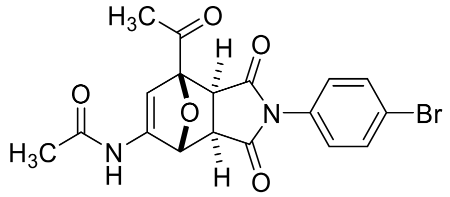

Prepared according to *Procedure B*, starting with 3-acetamido-5-acetylfuran (**3A5AF**, 33.4 mg, 0.20 mmol) and *N*-(4-bromophenyl)maleimide (**7f**, 100.8 mg, 0.40 mmol). Purified by flash chromatography (800 mL hexane/ethyl acetate 0–100%, then 200 mL ethyl acetate/methanol 0–10%); *Exo*:*endo* ratio > 19:1; R_f = 0.20 (ethyl acetate); Yield = 72% (60.2 mg, 0.14 mmol); Pale yellow solid; FTIR (ATR) $\tilde{\nu}$ 3343, 3131, 3056, 3004, 2960, 2926, 2855, 1778, 1703, 1633, 1528, 1487, 1368, 1305, 1260, 1245, 1182, 1152, 1066, 1032, 1014, 936, 880, 857, 827, 801, 783, 734, 712, 667 cm^{-1}; ^{1}H NMR (DMSO-*d$_6$*, 400 MHz) δ 10.34 (s, 1H), δ 7.71 (d, *J* = 8.6 Hz, 2H), δ 7.18 (d, *J* = 8.6 Hz, 2H), δ 6.20 (s, 1H), δ 5.31 (s, 1H), δ 3.61 (d, *J* = 6.6 Hz, 1H), δ 3.34 (d, *J* = 6.6 Hz, 1H), δ 2.29 (s, 3H), δ 2.01 (s, 3H); ^{13}C NMR (DMSO-*d$_6$*, 100 MHz) δ 202.3, 174.4, 173.3, 168.6, 144.7, 132.1, 131.0, 128.7, 121.5, 110.1, 94.8, 80.3, 52.7, 49.0, 26.9, 23.2 ppm; HRMS (ESI-TOF) *m/z* [M+H]$^+$ Calculated for $C_{18}H_{16}BrN_2O_5^+$ 419.0237, found 419.0240 (|Δ*m/z*|= 0.7 ppm).

Diels–Alder adduct **1g**

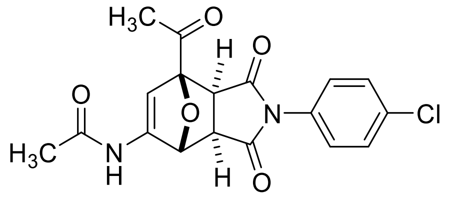

Prepared according to *Procedure B*, starting with 3-acetamido-5-acetylfuran (**3A5AF**, 33.4 mg, 0.20 mmol) and *N*-(4-chlorophenyl)maleimide (**7g**, 83.0 mg, 0.40 mmol). Purified by flash chromatography (800 mL hexane/ethyl acetate 0–100%, then 200 mL ethyl acetate/methanol 0–10%); *Exo*:*endo* ratio > 19:1; R_f = 0.30 (ethyl acetate); Yield = 58% (43.6 mg, 0.12 mmol); Pale yellow solid; FTIR (ATR) $\tilde{\nu}$ 3351, 3239, 3127, 3056, 2993, 1774, 1726, 1700, 1625, 1528, 1495, 1446, 1398, 1364, 1327, 1305, 1278, 1256, 1237, 1208, 1196, 1152, 1088, 1070, 1055, 1018, 958, 939, 921, 883, 861, 839, 801, 779, 727, 716, 671 cm^{-1}; ^{1}H NMR (DMSO-*d$_6$*, 400 MHz) δ 10.37 (s, 1H), δ 7.58 (d, *J* = 8.2 Hz, 2H), δ 7.25 (d, *J* = 8.2 Hz, 2H), δ 6.21 (s, 1H), δ 5.31 (s, 1H), δ 3.62 (d, *J* = 6.3 Hz, 1H), δ 3.35 (d, *J* = 6.3 Hz, 1H), δ 2.30 (s, 3H), δ 2.01 (s, 3H); ^{13}C NMR (DMSO-*d$_6$*, 100 MHz) δ 202.4, 174.5, 173.4, 168.7, 144.8, 133.1, 130.6, 129.2, 128.5, 110.1, 94.8, 80.4, 52.7, 49.1, 27.0, 23.3 ppm; HRMS (ESI-TOF) *m/z* [M+H]$^+$ Calculated for $C_{18}H_{16}ClN_2O_5^+$ 375.0742, found 375.0746 (|Δ*m/z*|= 1.1 ppm).

Diels–Alder adduct **1h**

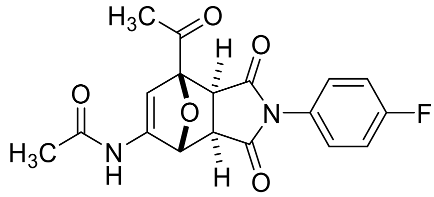

Prepared according to *Procedure B*, starting with 3-acetamido-5-acetylfuran (**3A5AF**, 33.4 mg, 0.20 mmol) and *N*-(4-fluorophenyl)maleimide (**7h**, 76.5 mg, 0.40 mmol). Purified by flash chromatography (800 mL hexane/ethyl acetate 0–100%, then 200 mL ethyl acetate/methanol 0–10%); *Exo*:*endo* ratio > 19:1; R_f = 0.20 (ethyl acetate); Yield = 60% (42.8 mg, 0.12 mmol); Pale yellow solid; FTIR (ATR) $\tilde{\nu}$ 3340, 3239, 3131, 3064, 3049, 2989, 2926, 1774, 1729, 1700, 1625, 1603, 1528, 1510, 1401, 1360, 1327, 1305, 1293, 1260, 1241, 1204, 1185, 1152, 1092, 1073, 1051, 1021, 1003, 958, 936, 913, 880, 850, 820, 805, 786, 753, 719, 701, 686, 671 cm^{-1}; ^{1}H NMR (DMSO-d_6, 400 MHz) δ 10.37 (s, 1H), δ 7.35 (t, J = 8.8 Hz, 2H), δ 7.25 (dd, J = 8.8, 5.1 Hz, 2H), δ 6.20 (s, 1H), δ 5.30 (s, 1H), δ 3.61 (d, J = 6.6 Hz, 1H), δ 3.34 (d, J = 6.6 Hz, 1H), δ 2.30 (s, 3H), δ 2.00 (s, 3H); ^{19}F NMR (DMSO-d_6, 376 MHz) δ –112.7 ppm; ^{13}C NMR (DMSO-d_6, 100 MHz) δ 202.4, 174.7, 173.6, 168.7, 162.8, 160.3, 144.7, 129.0, 128.9, 128.0(2), 127.9(9), 116.2, 116.0, 110.1, 94.8, 80.4, 52.7, 49.0, 27.0, 23.3 ppm; HRMS (ESI-TOF) m/z $[M+H]^+$ Calculated for $C_{18}H_{16}FN_2O_5^+$ 359.1038, found 359.1041 ($|\Delta m/z|$ = 0.8 ppm).

Diels–Alder adduct **1i**

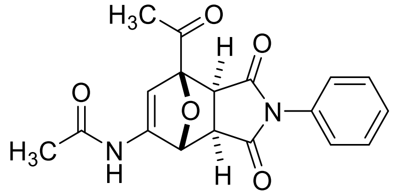

Prepared according to *Procedure B*, starting with 3-acetamido-5-acetylfuran (**3A5AF**, 33.4 mg, 0.20 mmol) and *N*-phenylmaleimide (**7i**, 69.3 mg, 0.40 mmol). Purified by flash chromatography (800 mL hexane/ethyl acetate 0–100%, then 200 mL ethyl acetate/methanol 0–10%); *Exo*:*endo* ratio > 19:1; R_f = 0.20 (ethyl acetate); Yield = 67% (45.6 mg, 0.13 mmol); Pale yellow solid; FTIR (ATR) $\tilde{\nu}$ 3348, 3061, 1778, 1726, 1696, 1637, 1595, 1541, 1526, 1496, 1456, 1386, 1362, 1332, 1300, 1280, 1262, 1242, 1208, 1195, 1185, 1149, 1080, 1061, 1047, 1030, 1017, 992, 943, 935, 917, 882, 856, 829, 808, 795, 780, 763, 755, 707, 701, 691, 677, 670, 625, 616, 608 cm^{-1}; ^{1}H NMR (DMSO-d_6, 400 MHz) δ 10.35 (s, 1H), δ 7.50 (t, J = 7.5 Hz, 2H), δ 7.43 (t, J = 7.5 Hz, 1H), δ 7.20 (t, J = 7.5 Hz, 2H), δ 6.21 (s, 1H), δ 5.31 (s, 1H), δ 3.60 (d, J = 6.6 Hz, 1H), δ 3.35 (d, J = 6.6 Hz, 1H), δ 2.30 (s, 3H), δ 2.01 (s, 3H); ^{13}C NMR (DMSO-d_6, 100 MHz) δ 202.4, 174.7, 173.5, 168.6, 144.7, 131.8, 129.0, 128.6, 126.7, 110.1, 94.8, 80.4, 52.6, 49.0, 26.9, 23.2

ppm; HRMS (ESI-TOF) *m/z* $[M+H]^+$ Calculated for $C_{18}H_{17}N_2O_5^+$ 341.1132, found 341.1126 (|Δ*m/z*|= 1.8 ppm).

Diels–Alder adduct **1j**

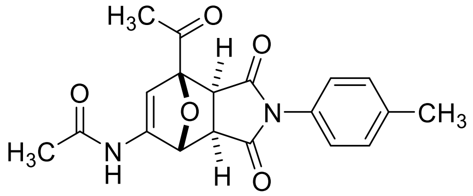

Prepared according to *Procedure B*, starting with 3-acetamido-5-acetylfuran (**3A5AF**, 33.4 mg, 0.20 mmol) and *N*-(4-methylphenyl)maleimide (**7j**, 74.9 mg, 0.40 mmol). Purified by flash chromatography (800 mL hexane/ethyl acetate 0–100%, then 200 mL ethyl acetate/methanol 0–10%); *Exo*:*endo* ratio > 19:1; R_f = 0.25 (ethyl acetate); Yield = 55% (39.0 mg, 0.11 mmol); Pale yellow solid; FTIR (ATR) $\tilde{\nu}$ 3362, 3153, 3004, 2960, 2926, 2855, 1770, 1700, 1633, 1528, 1513, 1457, 1394, 1364, 1334, 1301, 1282, 1241, 1193, 1148, 1107, 1070, 1047, 1032, 1018, 991, 943, 917, 883, 857, 827, 801, 786, 719, 701, 675 cm^{-1}; ^1H NMR (DMSO-*d*$_6$, 400 MHz) *δ* 10.34 (s, 1H), *δ* 7.29 (d, *J* = 8.1 Hz, 2H), *δ* 7.07 (d, *J* = 8.1 Hz, 2H), *δ* 6.19 (s, 1H), *δ* 5.30 (s, 1H), *δ* 3.58 (d, *J* = 6.5 Hz, 1H), *δ* 3.33 (d, *J* = 6.5 Hz, 1H), *δ* 2.34 (s, 3H), *δ* 2.28 (s, 3H), *δ* 2.01 (s, 3H); ^{13}C NMR (DMSO-*d*$_6$, 100 MHz) *δ* 202.4, 174.8, 173.6, 168.6, 144.7, 138.1, 129.5, 129.2, 126.4, 110.1, 94.7, 80.3, 52.6, 48.9, 26.9, 23.2, 20.7 ppm; HRMS (ESI-TOF) *m/z* $[M+H]^+$ Calculated for $C_{19}H_{19}N_2O_5^+$ 355.1288, found 355.1292 (|Δ*m/z*|= 1.1 ppm).

Diels–Alder adduct **1k**

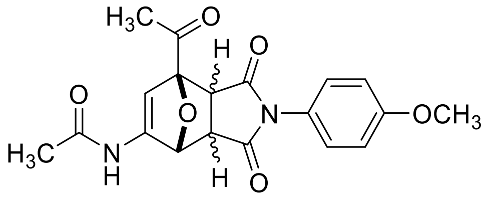

Prepared according to *Procedure B*, starting with 3-acetamido-5-acetylfuran (**3A5AF**, 33.4 mg, 0.20 mmol) and *N*-(4-methoxyphenyl)maleimide (**7k**, 81.3 mg, 0.40 mmol). Purified by flash chromatography (800 mL hexane/ethyl acetate 0–100%, then 200 mL ethyl acetate/methanol 0–10%); *Exo*:*endo* ratio = 19:1; R_f = 0.25 (ethyl acetate); Yield = 57% (42.1 mg, 0.11 mmol); Off-white solid; FTIR (ATR) $\tilde{\nu}$ 3355, 3235, 3150, 3064, 3012, 2963, 2941, 2919, 2840, 1778, 1722, 1700, 1636, 1606, 1528, 1510, 1469, 1446, 1420, 1394, 1364, 1334, 1301, 1278, 1241, 1204, 1185, 1170, 1148, 1111, 1066, 1021, 958, 939, 921, 883, 857, 831, 801, 779, 757, 723, 701, 675 cm^{-1}; ^1H NMR (DMSO-*d*$_6$, 400 MHz) *δ* 10.37 (s, 0.05H), *δ* 10.34 (s, 0.95H), *δ* 7.10 (d, *J* = 8.9 Hz, 1.90H), *δ* 7.03 (d, *J* = 8.9 Hz, 1.90H), *δ* 6.99 (d, *J* = 8.9 Hz, 0.10H), *δ* 6.90 (d, *J* = 8.9 Hz, 0.10H), *δ* 6.26 (s, 0.05H), *δ* 6.19 (s, 0.95H), *δ* 5.40 (d, *J* = 5.4 Hz, 0.05H), *δ* 5.30 (s, 0.95H), *δ* 4.08 (q, *J* = 6.6

Hz, 0.05H), *δ* 3.96 (t, *J* = 6.6 Hz, 0.05H), *δ* 3.78 (s, 2.85H), *δ* 3.77 (s, 0.15H), *δ* 3.57 (d, *J* = 6.6 Hz, 0.95H), *δ* 3.32 (d, *J* = 6.6 Hz, 0.95H), *δ* 2.37 (s, 0.15H), *δ* 2.28 (s, 2.85H), *δ* 2.01 (s, 2.85H), *δ* 1.93 (s, 0.15H); ^{13}C NMR (DMSO-d_6, 100 MHz) *δ* 202.4 (*exo*), 202.2 (*endo*), 174.9 (*exo*), 173.7 (*exo*), 173.3 (*endo*), 173.0 (*endo*), 168.6 (*exo*), 168.5 (*endo*), 159.1 (*endo*), 159.0 (*exo*), 144.7 (*exo*), 139.8 (*endo*), 128.2 (*endo*), 127.9 (*exo*), 124.5 (*endo*), 124.4 (*exo*), 114.3 (*exo*), 114.2 (*endo*), 110.1 (*exo*), 107.1 (*endo*), 94.7 (*exo*), 93.3 (*endo*), 80.3 (*exo*), 78.8 (*endo*), 55.4 (*exo*), 54.9 (*endo*), 52.5 (*exo*), 49.8 (*endo*), 48.8 (*exo*), 47.5 (*endo*), 26.9 (*exo*), 26.2 (*endo*), 23.2 (*exo*), 23.1 (*endo*) ppm; HRMS (ESI-TOF) *m/z* [M+H]$^+$ Calculated for $C_{19}H_{19}N_2O_6^+$ 371.1238, found 371.1245 (|Δ*m/z*|= 1.9 ppm).

Diels–Alder adduct **1l**

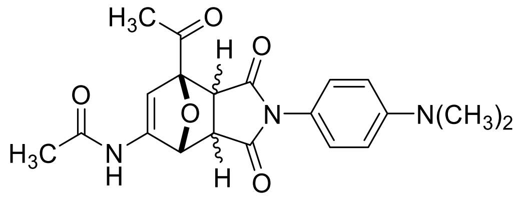

Prepared according to *Procedure B*, starting with 3-acetamido-5-acetylfuran (**3A5AF**, 33.4 mg, 0.20 mmol) and *N*-(4-(dimethylamino)phenyl)maleimide (**7l**, 86.5 mg, 0.40 mmol). Purified by flash chromatography (800 mL hexane/ethyl acetate 0–100%, then 200 mL ethyl acetate/methanol 0–10%); *Exo*:*endo* ratio = 6:1; R_f = 0.25 (ethyl acetate); Yield = 64% (48.8 mg, 0.13 mmol); Pale yellow solid; FTIR (ATR) $\tilde{\nu}$ 3329, 3265, 3235, 3138, 3049, 2967, 2922, 2889, 2848, 2799, 1774, 1726, 1700, 1633, 1606, 1521, 1446, 1409, 1360, 1346, 1305, 1282, 1256, 1234, 1215, 1200, 1182, 1148, 1129, 1055, 1036, 1018, 1003, 965, 939, 924, 887, 861, 831, 805, 775, 727, 708, 693, 675 cm^{-1}; ^{1}H NMR (DMSO-d_6, 400 MHz) *δ* 10.35 (s, 0.14H), *δ* 10.33 (s, 0.86H), *δ* 6.96 (d, *J* = 9.0 Hz, 1.72H), *δ* 6.76 (d, *J* = 9.0 Hz, 0.28H), *δ* 6.75 (d, *J* = 9.0 Hz, 1.72H), *δ* 6.70 (d, *J* = 9.0 Hz, 0.28H), *δ* 6.25 (s, 0.14H), *δ* 6.18 (s, 0.86H), *δ* 5.38 (d, *J* = 5.4 Hz, 0.14H), *δ* 5.28 (s, 0.86H), *δ* 3.93 (dd, *J* = 7.7, 5.4 Hz, 0.14H), *δ* 3.77 (d, *J* = 7.7 Hz, 0.14H), *δ* 3.53 (d, *J* = 6.6 Hz, 0.86H), *δ* 3.29 (d, *J* = 6.6 Hz, 0.86H), *δ* 2.92 (s, 5.16H), *δ* 2.90 (s, 0.84H), *δ* 2.37 (s, 0.42H), *δ* 2.27 (s, 2.58H), *δ* 2.00 (s, 2.58H), *δ* 1.93 (s, 0.42H); ^{13}C NMR (DMSO-d_6, 100 MHz) *δ* 202.5 (*exo*), 202.4 (*endo*), 175.1 (*exo*), 173.9 (*exo*), 173.3 (*endo*), 173.0 (*endo*), 168.6 (*exo*), 168.5 (*endo*), 150.2 (*endo*), 150.1 (*exo*), 144.6 (*exo*), 142.1 (*endo*), 127.5 (*endo*), 127.2 (*exo*), 120.2 (*endo*), 120.1 (*exo*), 111.9 (*exo*), 110.1 (*exo*), 107.0 (*endo*), 105.7 (*endo*), 94.7 (*exo*), 93.3 (*endo*), 80.3 (*exo*), 78.8 (*endo*), 52.5 (*exo*), 49.7 (*endo*), 48.7 (*exo*), 47.4 (*endo*), 40.0(4) (*endo*), 40.0(2) (*exo*), 26.9 (*exo*), 26.2 (*endo*), 23.2 (*exo*), 23.1 (*endo*) ppm; HRMS (ESI-TOF) *m/z* [M+H]$^+$ Calculated for $C_{20}H_{22}N_3O_5^+$ 384.1554, found 384.1555 (|Δ*m/z*|= 0.3 ppm).

3-Acetamido-5-furfural (**3A5F**)

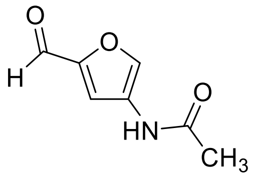

Prepared according to *Procedure C*, starting with *N*-acetylglucosamine (NAG, 4.0 g, 18 mmol). Purified by flash chromatography (2.0 L hexane/ethyl acetate 0–80%); R_f = 0.10 (hexane/ethyl acetate 50%); Yield = 36% (1.0 g, 6.5 mmol); Beige solid; FTIR (ATR) $\tilde{\nu}$ 3204, 3171, 3051, 2849, 1995, 1902, 1828, 1674, 1659, 1623, 1570, 1510, 1460, 1442, 1417, 1385, 1357, 1344, 1323, 1294, 1277, 1251, 1222, 1209, 1194, 1142, 1126, 1088, 1069, 1047, 1017, 1001, 980, 968, 938, 849, 829, 794, 768, 744, 702, 696, 688, 675, 648, 626 cm^{-1}; ^{1}H NMR (DMSO-d_6, 400 MHz) δ 10.29 (s, 1H), δ 9.56 (s, 1H), δ 8.29 (s, 1H), δ 7.36 (s, 1H), δ 2.03 (s, 3H); ^{13}C NMR (DMSO-d_6, 100 MHz) δ 178.8, 167.8, 150.1, 136.8, 127.4, 115.3, 22.8 ppm.

Hydrazone **2**

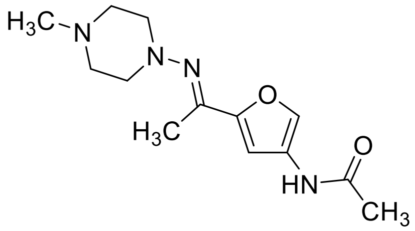

Prepared according to *Procedure D*, starting with 3-acetamido-5-acetylfuran (**3A5AF**, 167 mg, 1.0 mmol) and 1-amino-4-methylpiperazine (364 µL, 3.0 mmol). Purified by flash chromatography (1.0 L dichloromethane/methanol 0–20%); R_f = 0.20 (dichloromethane/methanol 10%); Yield = 64% (168 mg, 0.64 mmol); Yellow solid; FTIR (ATR) $\tilde{\nu}$ 3458, 3332, 3225, 3164, 3119, 2959, 2919, 2889, 2849, 2832, 2799, 2769, 2747, 2705, 2681, 1794, 1659, 1613, 1593, 1522, 1458, 1443, 1414, 1365, 1356, 1299, 1283, 1273, 1239, 1222, 1207, 1187, 1148, 1138, 1131, 1123, 1089, 1076, 1055, 1038, 1031, 1010, 1001, 984, 961, 930, 902, 831, 783, 772, 757, 736, 705, 650, 618 cm^{-1}; ^{1}H NMR (DMSO-d_6, 400 MHz) δ 9.38 (br s, 1H), δ 8.68 (s, 1H), δ 7.78 (s, 1H), δ 2.89–2.87 (m, 4H), δ 2.54 (br s, 4H), δ 2.26 (s, 3H), δ 2.20 (s, 3H), δ 2.09 (s, 3H); ^{13}C NMR (DMSO-d_6, 100 MHz) δ 168.5, 147.4, 145.0, 137.6, 125.0, 116.7, 54.5, 49.5, 45.6, 23.9, 18.6 ppm; HRMS (ESI-TOF) *m/z* $[M+H]^+$ Calculated for $C_{13}H_{21}N_4O_2^+$ 265.1659, found 265.1657 ($|\Delta m/z|$ = 0.8 ppm).

Hydrazone **3**

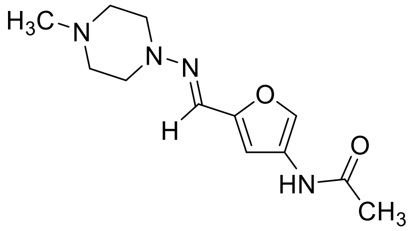

Prepared according to *Procedure E*, starting with 3-acetamido-5-furfural (**3A5F**, 153 mg, 1.0 mmol) and 1-amino-4-methylpiperazine (132 µL, 1.1 mmol). Purified by flash chromatography (1.0 L dichloromethane/methanol 0–15%) and precipitation (10 mL hexane/chloroform 20%); R_f = 0.25 (dichloromethane/methanol 10%); Yield = 81% (chromatography, 202 mg, 0.81 mmol) and 73% (precipitation, 182 mg, 0.73 mmol); Yellow solid; FTIR (ATR) $\tilde{\nu}$ 3330, 3276, 3171, 3008, 2973, 2936, 2857, 2842, 2813, 1654, 1577, 1513, 1453, 1375, 1364, 1339, 1300, 1280, 1225, 1166, 1158, 1136, 1121, 1104, 1075, 1052, 1029, 1020, 995, 969, 938, 885, 865, 854, 832, 826, 800, 777, 750, 726, 711, 669, 637, 623 cm^{-1}; ^{1}H NMR (DMSO-*d*$_6$, 400 MHz) δ 10.01 (s, 1H), δ 7.88 (d, *J* = 0.6 Hz, 1H), δ 7.44 (s, 1H), δ 6.43 (d, *J* = 1.2 Hz, 1H), δ 3.07–3.05 (m, 4H), δ 2.51–2.49 (m, 4H), δ 2.24 (s, 3H), δ 1.99 (s, 3H); ^{13}C NMR (DMSO-*d*$_6$, 100 MHz) δ 167.2, 149.7, 130.6, 126.5, 126.0, 102.5, 53.7, 50.2, 45.3, 22.8 ppm; HRMS (ESI-TOF) *m/z* [M+H]$^+$ Calculated for $C_{12}H_{19}N_4O_2^+$ 251.1503, found 251.1499 ($|\Delta m/z|$= 1.6 ppm).

Phthalimide **3a**

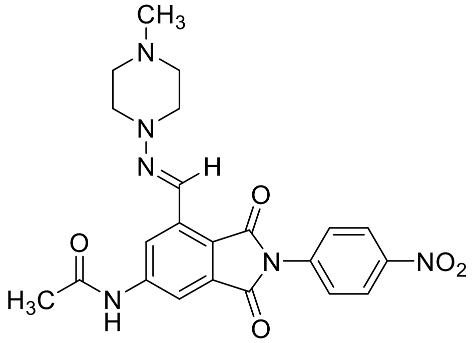

Prepared according to *Procedures F* and *G*, starting with hydrazone **3** (15.0 mg, 0.06 mmol) and *N*-(4-nitrophenyl)maleimide (**7b**, 10.9 mg; 0.05 mmol). Purified by flash chromatography (0.5 L dichloromethane/methanol 0–15%; R_f = 0.20 (dichloromethane/methanol 5%); Yield = 66% (*Procedure F*, 14.8 mg, 0.33 mmol) and 64% (*Procedure G*, 14.4 mg, 0.32 mmol); Yellow solid; FTIR (ATR) $\tilde{\nu}$ 3362, 2940, 2841, 2800, 1788, 1711, 1632, 1575, 1522, 1449, 1395, 1368, 1341, 1313, 1280, 1241, 1205, 1171, 1143, 1117, 1108, 1076, 993, 922, 888, 872, 857, 844, 813, 802, 782, 754, 745, 722, 707, 669 cm^{-1}; ^{1}H NMR (400 MHz, DMSO-*d*$_6$) δ 10.61 (s, 1H), δ 8.38 (d, *J* = 9.2 Hz, 2H), δ 8.32 (s, 1H), δ 8.25 (d, *J* = 1.8 Hz, 1H), δ 8.23 (d, *J* = 1.8 Hz, 1H), δ 7.78 (d, *J* = 9.2 Hz, 2H), δ 3.25–3.21 (m, 4H), δ 2.54–2.51 (m, 4H), δ 2.25 (s, 3H), δ 2.12 (s, 3H); ^{13}C

NMR (100 MHz, DMSO-d_6) δ 169.4, 166.4, 166.0, 145.9, 144.7, 137.8, 136.2, 133.5, 127.5, 127.0, 124.1, 119.5, 117.0, 112.3, 53.7, 50.1, 45.5, 24.2; HRMS (ESI-TOF) *m/z* $[M+H]^+$ Calculated for $C_{22}H_{23}N_6O_5^+$ 451.1724, found 451.1720 (|Δ*m/z*|= 0.9 ppm).

Phthalimide **3b**

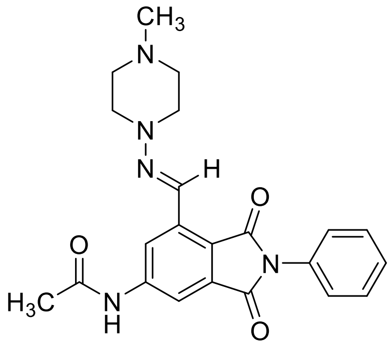

Prepared according to *Procedures F* and *G*, starting with hydrazone **3** (15.0 mg, 0.06 mmol) and *N*-phenylmaleimide (**7i**, 8.7 mg; 0.05 mmol). Purified by flash chromatography (0.5 L dichloromethane/methanol 0–15%); R_f = 0.25 (dichloromethane/methanol 5%); Yield = 79% (*Procedure F*, 16.0 mg, 0.40 mmol) and 66% (*Procedure G*, 13.4 mg, 0.33 mmol); Yellow solid; FTIR (ATR) $\tilde{\nu}$ 3368, 2938, 2835, 2802, 1764, 1702, 1621, 1596, 1543, 1488, 1458, 1393, 1364, 1346, 1314, 1281, 1253, 1201, 1164, 1141, 1110, 1076, 1068, 1020, 994, 921, 889, 862, 828, 804, 784, 758, 749, 718, 704, 690 cm^{-1}; ^{1}H NMR (400 MHz, DMSO-d_6) δ 10.59 (s, 1H), δ 8.34 (s, 1H), δ 8.22 (s, 2H), δ 7.53–7.50 (m, 2H), δ 7.44–7.41 (m, 3H), δ 3.23–3.21 (m, 4H), δ 2.53–2.51 (m, 4H), δ 2.24 (s, 3H), δ 2.12 (s, 3H); ^{13}C NMR (100 MHz, DMSO-d_6) δ 169.3, 167.1, 166.6, 144.5, 135.9, 133.6, 131.9, 128.7, 127.9, 127.3(2), 127.2(8), 119.6, 116.8, 112.2, 53.7, 50.2, 45.5, 24.2; HRMS (ESI-TOF) *m/z* $[M+H]^+$ Calculated for $C_{22}H_{24}N_5O_3^+$ 406.1874, found 406.1872 (|Δ*m/z*|= 0.5 ppm).

Scale-up reaction: Performed according to *Procedure F* starting with hydrazone **3** (150 mg, 0.60 mmol) and *N*-phenylmaleimide (**7i**, 86.6 mg; 0.50 mmol). Purified by recrystallisation (3 mL acetonitrile/ethanol 30%); Yield = 76% (154 mg, 0.38 mmol). Alternatively, the reaction mixture can be purified by flash chromatography (1.0 L dichloromethane/methanol 0–15%, 25 g silica gel); Yield = 72% (145 mg, 0.36 mmol).

Phthalimide **3c**

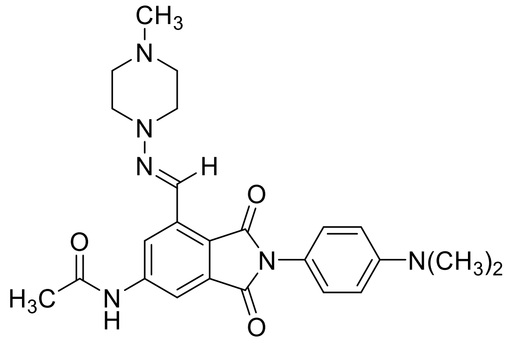

Prepared according to *Procedures F* and *G*, starting with hydrazone **3** (15.0 mg, 0.06 mmol) and *N*-(4-(dimethylamino)phenyl)maleimide (**7l**, 10.8 mg; 0.05 mmol). Purified by flash chromatography (0.5 L dichloromethane/methanol 0–15%); R_f = 0.25 (dichloromethane/methanol 5%); Yield = 46% (*Procedure F*, 10.4 mg, 0.02 mmol) and 45% (*Procedure G*, 10.2 mg, 0.03 mmol); Yellow solid; FTIR (ATR) $\tilde{\nu}$ 3318, 3295, 2939, 2843, 2801, 1762, 1702, 1613, 1556, 1519, 1477, 1449, 1418, 1367, 1347, 1305, 1284, 1266, 1253, 1210, 1186, 1163, 1137, 1128, 1096, 1076, 1053, 1022, 1022, 997, 965, 945, 914, 885, 863, 848, 812, 804, 787, 750, 741, 720, 699, 676 cm^{-1}; ^1H NMR (400 MHz, DMSO-d_6) δ 10.58 (s, 1H), δ 8.33 (s, 1H), δ 8.21 (d, J = 1.8 Hz, 1H), δ 8.18 (d, J = 1.8 Hz, 1H), δ 7.17 (d, J = 9.0 Hz, 2H), δ 6.78 (d, J = 9.0 Hz, 2H), δ 3.22–3.20 (m, 4H), δ 2.94 (s, 6H), δ 2.53–2.51 (m, 4H), δ 2.24 (s, 3H), δ 2.11 (s, 3H); ^{13}C NMR (100 MHz, DMSO-d_6) δ 169.3, 167.7, 167.1, 149.9, 144.3, 135.7, 133.6, 128.0, 127.4, 120.2, 119.8, 116.7, 112.1, 111.9, 53.7, 50.2, 45.5, 40.1, 24.2; HRMS (ESI-TOF) m/z $[M+H]^+$ Calculated for $C_{24}H_{29}N_6O_3^+$ 449.2296, found 449.2296 ($|\Delta m/z|$= 0).

Phthalimide **3d**

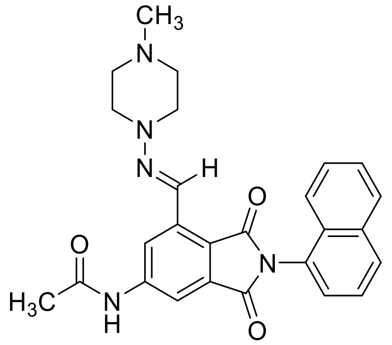

Prepared according to *Procedures F* and *G*, starting with hydrazone **3** (15.0 mg, 0.06 mmol) and *N*-(naphthalene-1-yl)maleimide (**7m**, 11.2 mg; 0.05 mmol). Purified by flash chromatography (0.5 L dichloromethane/methanol 0–15%); R_f = 0.30 (dichloromethane/methanol 5%); Yield = 54% (*Procedure F*, 12.2 mg, 0.03 mmol) and 48% (*Procedure G*, 11.0 mg, 0.02 mmol); Yellow solid; FTIR (ATR) $\tilde{\nu}$ 3226, 3094, 3061, 2941, 2841, 2789, 1760, 1697, 1618, 1598, 1551, 1473, 1465, 1401, 1363, 1306, 1250, 1161, 1151, 1104, 1077, 1051, 999, 876, 800, 746, 729, 707, 628 cm^{-1}; ^1H NMR (400

MHz, DMSO-d_6) δ 10.63 (s, 1H), δ 8.34 (s, 1H), δ 8.28 (d, *J* = 1.8 Hz, 1H), δ 8.27 (d, *J* = 1.8 Hz, 1H), δ 8.10 (d, *J* = 8.2 Hz, 1H), δ 8.06 (d, *J* = 8.2 Hz, 1H), δ 7.76 (d, *J* = 8.2 Hz, 1H), δ 7.67–7.62 (m, 2H), δ 7.60 (ddd, *J* = 8.2, 6.8, 1.2 Hz, 1H), δ 7.53 (ddd, *J* = 8.2, 6.8, 1.2 Hz, 1H), δ 3.22–3.20 (m, 4H), δ 2.51–2.50 (m, 4H), δ 2.23 (s, 3H), δ 2.14 (s, 3H); ^{13}C NMR (100 MHz, DMSO-d_6) δ 169.4, 167.7, 167.2, 144.5, 136.1, 133.9, 133.7, 130.2, 129.4, 128.6, 128.2, 127.4, 127.3, 127.1, 126.5, 125.6, 122.9, 120.0, 116.9, 112.4, 53.7, 50.1, 45.5, 24.2; HRMS (ESI-TOF) *m/z* [M+H]$^+$ Calculated for $C_{26}H_{26}N_5O_3^+$ 456.2030, found 456.2026 (|Δ*m/z*|= 0.9 ppm).

Phthalimide **3e**

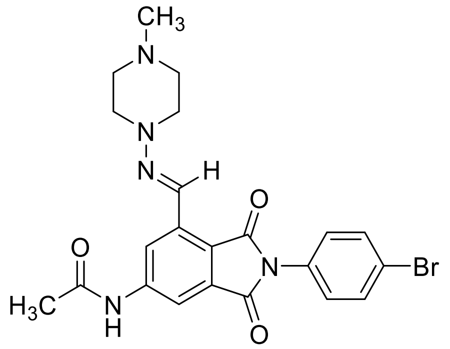

Prepared according to *Procedures F* and *G*, starting with hydrazone **3** (15.0 mg, 0.06 mmol) and *N*-(4-bromophenyl)maleimide (**7f**, 12.6 mg; 0.05 mmol). Purified by flash chromatography (0.5 L dichloromethane/methanol 0–15%); R_f = 0.20 (dichloromethane/methanol 5%); Yield = 64% (*Procedure F*, 15.4 mg, 0.03 mmol) and 78% (*Procedure G*, 18.8 mg, 0.04 mmol); Yellow solid; FTIR (ATR) $\tilde{\nu}$ 3385, 3275, 2941, 2842, 2797, 1765, 1701, 1676, 1620, 1553, 1478, 1456, 1368, 1348, 1306, 1282, 1254, 1199, 1166, 1142, 1108, 1108, 1076, 1048, 1023, 995, 889, 826, 800, 747, 722 cm^{-1}; ^{1}H NMR (400 MHz, DMSO-d_6) δ 10.57 (s, 1H), δ 8.29 (s, 1H), δ 8.20 (d, *J* = 1.7 Hz, 1H), δ 8.19 (d, *J* = 1.7 Hz, 1H), δ 7.71 (d, *J* = 8.7 Hz, 2H), δ 7.41 (d, *J* = 8.7 Hz, 2H), δ 3.21–3.19 (m, 4H), δ 2.52–2.50 (m, 4H), δ 2.24 (s, 3H), δ 2.11 (s, 3H); ^{13}C NMR (100 MHz, DMSO-d_6) δ 169.3, 166.7, 166.3, 144.5, 136.0, 133.5, 131.7, 131.2, 129.2, 127.1, 120.7, 119.5, 116.8, 112.2, 53.7, 50.1, 45.5, 24.2; HRMS (ESI-TOF) *m/z* [M+H]$^+$ Calculated for $C_{22}H_{23}BrN_5O_3^+$ 484.0979, found 484.0980 (|Δ*m/z*|= 0.2 ppm).

Phthalimide **3f**

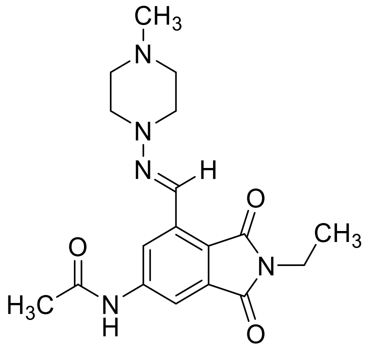

Prepared according to *Procedures F* and *G*, starting with hydrazone **3** (15.0 mg, 0.06 mmol) and *N*-ethylmaleimide (6.3 mg; 0.05 mmol). Purified by flash chromatography (0.5 L dichloromethane/methanol 0–15%); R_f = 0.25 (dichloromethane/methanol 5%); Yield = 64% (*Procedure F*, 11.4 mg, 0.03 mmol) and 71% (*Procedure G*, 12.7 mg, 0.04 mmol); Yellow solid; FTIR (ATR) $\tilde{\nu}$ 3547, 2969, 2950, 2870, 2817, 1755, 1691, 1602, 1555, 1458, 1448, 1399, 1375, 1365, 1354, 1346, 1335, 1281, 1255, 1201, 1171, 1157, 1139, 1116, 1077, 1052, 1032, 1017, 995, 973, 915, 882, 812, 801, 789, 753, 719, 698, 677 cm^{-1}; ^{1}H NMR (400 MHz, DMSO-*d*$_6$) δ 10.52 (s, 1H), δ 8.29 (s, 1H), δ 8.14 (d, *J* = 1.7 Hz, 1H), δ 8.11 (d, *J* = 1.7 Hz, 1H), δ 3.56 (q, *J* = 7.2 Hz, 2H), δ 3.32–3.20 (m, 4H), δ 2.54–2.52 (m, 4H), δ 2.25 (s, 3H), δ 2.10 (s, 3H), δ 1.15 (t, *J* = 7.2 Hz, 3H); ^{13}C NMR (100 MHz, DMSO-*d*$_6$) δ 169.2, 167.9, 167.3, 144.2, 135.5, 133.7, 127.3, 119.9, 116.4, 111.9, 53.7, 50.1, 45.5, 32.2, 24.2, 13.6; HRMS (ESI-TOF) *m/z* [M+H]$^+$ Calculated for $C_{18}H_{24}N_5O_3^+$ 358.1874, found 358.1872 ($|\Delta m/z|$= 0.6 ppm).

Phthalimide **3g**

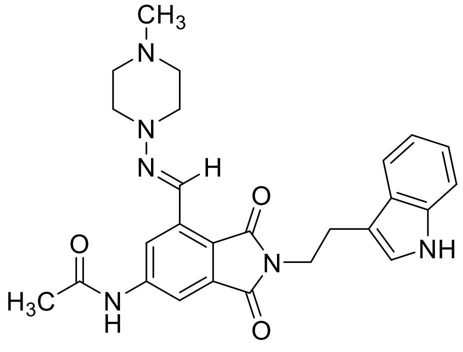

Prepared according to *Procedures F* and *G*, starting with hydrazone **3** (15.0 mg, 0.06 mmol) and *N*-(2-(1*H*-indol-3-yl)ethyl)maleimide (**7o**, 12.0 mg; 0.05 mmol). Purified by flash chromatography (0.5 L dichloromethane/methanol 0–15%); R_f = 0.15 (dichloromethane/methanol 5%); Yield = 46% (*Procedure F*, 10.8 mg, 0.02 mmol) and 60% (*Procedure G*, 14.2 mg, 0.03 mmol); Yellow solid; FTIR (ATR) $\tilde{\nu}$ 3357, 3271, 2942, 2799, 2360, 2332, 1755, 1696, 1685, 1631, 1599, 1554, 1457, 1440, 1404, 1368, 1349, 1337, 1270, 1231, 1201, 1175, 1143, 1123, 1123, 1101, 1076, 1063, 1016, 1001, 989,

962, 919, 905, 884, 847, 816, 802, 787, 757, 739, 732, 720, 696, 681 cm^{-1}; ^{1}H NMR (400 MHz, DMSO-d_6) δ 10.84 (s, 1H), δ 10.53 (s, 1H), δ 8.30 (s, 1H), δ 8.15 (d, J = 1.8 Hz, 1H), δ 8.11 (d, J = 1.8 Hz, 1H), δ 7.56 (d, J = 8.0 Hz, 1H), δ 7.33 (d, J = 8.0 Hz, 1H), δ 7.18 (d, J = 2.3 Hz, 1H), δ 7.08–7.05 (m, 1H), δ 7.00–6.97 (m, 1H), δ 3.81–3.77 (m, 2H), δ 3.23–3.21 (m, 4H), δ 3.02–2.98 (m, 2H), δ 2.54–2.52 (m, 4H), δ 2.25 (s, 3H), δ 2.10 (s, 3H); ^{13}C NMR (100 MHz, DMSO-d_6) δ 169.3, 168.0, 167.4, 144.2, 136.2, 135.5, 133.6, 127.3, 127.0, 122.9, 121.0, 119.8, 118.4, 118.0, 116.4, 112.0, 111.5, 110.6, 53.7, 50.2, 45.5, 38.2, 24.2, 24.0; HRMS (ESI-TOF) m/z [M+H]$^+$ Calculated for $C_{26}H_{29}N_6O_3^+$ 473.2296, found 473.2297 ($|\Delta m/z|$= 0.2 ppm).

Phthalimide **3h**

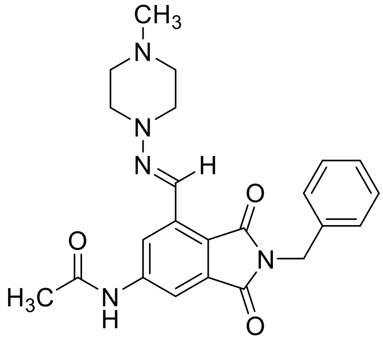

Prepared according to *Procedures F* and *G*, starting with hydrazone **3** (15.0 mg, 0.06 mmol) and *N*-benzylmaleimide (**7n**, 9.4 mg; 0.05 mmol). Purified by flash chromatography (0.5 L dichloromethane/methanol 0–15%); R_f = 0.25 (dichloromethane/methanol 5%); Yield = 69% (*Procedure F*, 14.5 mg, 0.03 mmol) and 47% (*Procedure G*, 9.8 mg, 0.02 mmol); Yellow solid; FTIR (ATR) $\tilde{\nu}$ 3372, 2941, 2827, 2360, 2344, 1759, 1696, 1600, 1554, 1457, 1442, 1402, 1365, 1345, 1277, 1257, 1168, 1137, 1107, 1072, 996, 939, 923, 879, 791, 751, 722, 703 cm^{-1}; ^{1}H NMR (400 MHz, DMSO-d_6) δ 10.56 (s, 1H), δ 8.29 (s, 1H), δ 8.17 (d, J = 1.8 Hz, 1H), δ 8.15 (d, J = 1.8 Hz, 1H), δ 7.34–7.25 (m, 5H), δ 4.72 (s, 2H), δ 3.22–3.20 (m, 4H), δ 2.53–2.51 (m, 4H), δ 2.24 (s, 3H), δ 2.10 (s, 3H); ^{13}C NMR (100 MHz, DMSO-d_6) δ 169.3, 167.8, 167.3, 144.4, 136.8, 135.8, 133.6, 128.6, 127.5, 127.4, 127.2, 119.7, 116.6, 112.1, 53.7, 50.1, 45.5, 40.8, 24.2; HRMS (ESI-TOF) m/z [M+H]$^+$ Calculated for $C_{23}H_{26}N_5O_3^+$ 420.2030, found 420.2024 ($|\Delta m/z|$= 1.4 ppm).

Phthalimide **3i**

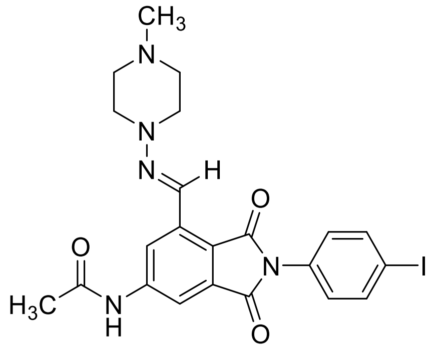

Prepared according to *Procedures F* and *G*, starting with hydrazone **3** (15.0 mg, 0.06 mmol) and *N*-(4-iodophenyl)maleimide (**7e**, 15.0 mg; 0.05 mmol). Purified by flash chromatography (0.5 L dichloromethane/methanol 0–15%); R_f = 0.20 (dichloromethane/methanol 5%); Yield = 42% (*Procedure F*, 11.1 mg, 0.02 mmol) and 41% (*Procedure G*, 10.8 mg, 0.02 mmol); Yellow solid; FTIR (ATR) $\tilde{\nu}$ 3374, 2939, 2836, 2801, 2767, 1733, 1706, 1666, 1622, 1539, 1489, 1441, 1398, 1340, 1300, 1281, 1266, 1142, 1113, 1076, 1060, 995, 921, 890, 863, 826, 802, 783, 749, 721, 711, 664 cm^{-1}; ^{1}H NMR (400 MHz, DMSO-d_6) δ 10.59 (s, 1H), δ 8.31 (s, 1H), δ 8.21 (d, J = 1.8 Hz, 1H), δ 8.20 (d, J = 1.8 Hz, 1H), δ 7.87 (d, J = 8.6 Hz, 2H), δ 7.25 (d, J = 8.6 Hz, 2H), δ 3.22–3.20 (m, 4H), δ 2.53–2.51 (m, 4H), δ 2.24 (s, 3H), δ 2.11 (s, 3H); ^{13}C NMR (100 MHz, DMSO-d_6) δ 169.4, 166.8, 166.3, 144.5, 137.6, 136.0, 133.6, 131.7, 129.3, 127.2, 119.6, 116.8, 112.2, 93.8, 53.7, 50.2, 45.5, 24.2; HRMS (ESI-TOF) *m/z* $[M+H]^+$ Calculated for $C_{22}H_{23}IN_5O_3^+$ 532.0840, found 532.0837 ($|\Delta m/z|$ = 0.6 ppm).

Phthalimide **3j**

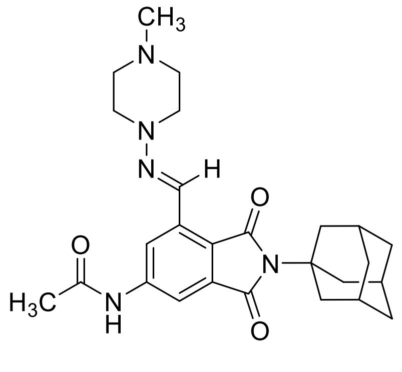

Prepared according to *Procedures F* and *G*, starting with hydrazone **3** (15.0 mg, 0.06 mmol) and *N*-(1-adamantyl)maleimide (**7p**, 11.6 mg; 0.05 mmol). Purified by flash chromatography (0.5 L dichloromethane/methanol 0–15%); R_f = 0.25 (dichloromethane/methanol 5%); Yield = 65% (*Procedure F*, 15.1 mg, 0.03 mmol) and 48% (*Procedure G*, 11.1 mg, 0.02 mmol); Yellow solid; FTIR (ATR) $\tilde{\nu}$ 2908, 2850, 2804, 2362, 1753, 1696, 1627, 1601, 1555, 1480, 1453, 1371, 1308, 1287, 1269, 1164, 1142, 1111, 1082, 999, 892, 819, 794, 754, 704, 668 cm^{-1}; ^{1}H NMR (400 MHz, DMSO-d_6) δ

10.48 (s, 1H), δ 8.30 (s, 1H), δ 8.12 (d, J = 1.8 Hz, 1H), δ 8.03 (d, J = 1.8 Hz, 1H), δ 3.21–3.19 (m, 4H), δ 2.53–2.52 (m, 4H), δ 2.43 (d, J = 2.6 Hz, 6H), δ 2.25 (s, 3H), δ 2.09 (br s, 3H), δ 2.08 (s, 3H), δ 1.71–1.65 (m, 6H); ^{13}C NMR (100 MHz, DMSO-d_6) δ 169.6, 169.2, 168.6, 144.1, 135.1, 133.4, 127.5, 119.4, 116.7, 111.3, 59.4, 53.7, 50.2, 45.5, 40.1, 35.7, 29.2, 24.1; HRMS (ESI-TOF) *m/z* $[M+H]^+$ Calculated for $C_{26}H_{34}N_5O_3^+$ 464.2656, found 464.2652 (|Δ*m/z*|= 0.9 ppm).

Phthalimide **3k**

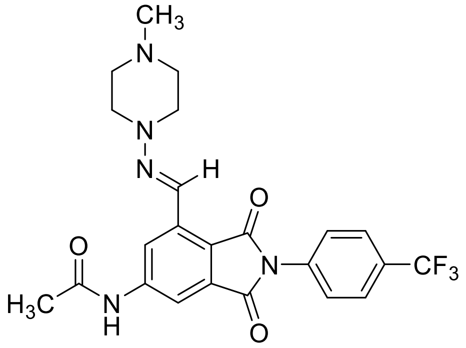

Prepared according to *Procedures F* and *G*, starting with hydrazone **3** (15.0 mg, 0.06 mmol) and *N*-(4-(trifluoromethyl)phenyl)maleimide (**7d**, 12.1 mg; 0.05 mmol). Purified by flash chromatography (0.5 L dichloromethane/methanol 0–15%); R_f = 0.30 (dichloromethane/methanol 5%); Yield = 63% (*Procedure F*, 14.9 mg, 0.03 mmol) and 50% (*Procedure G*, 11.8 mg, 0.03 mmol); Yellow solid; FTIR (ATR) $\tilde{\nu}$ 3387, 2939, 2836, 2805, 1766, 1701, 1624, 1597, 1540, 1458, 1452, 1395, 1364, 1347, 1323, 1282, 1247, 1206, 1158, 1146, 1115, 1078, 1068, 1020, 994, 949, 922, 891, 867, 825, 811, 789, 757, 750, 716, 703, 677, 665 cm^{-1}; ^{1}H NMR (400 MHz, DMSO-d_6) δ 10.60 (s, 1H), δ 8.31 (s, 1H), δ 8.23 (d, J = 1.8 Hz, 1H), δ 8.22 (d, J = 1.8 Hz, 1H), δ 7.90 (d, J = 8.4 Hz, 2H), δ 7.71 (d, J = 8.4 Hz, 2H), δ 3.23–3.21 (m, 4H), δ 2.53–2.51 (m, 4H), δ 2.24 (s, 3H), δ 2.12 (s, 3H); ^{19}F NMR (376 MHz, DMSO-d_6) δ –60.92 ppm; ^{13}C NMR (100 MHz, DMSO-d_6) δ 169.4, 166.6, 166.2, 144.6, 136.1, 135.6, 133.5, 128.2, 128.0, 127.8, 127.5(9), 127.5(5), 127.1, 126.7, 125.8(5), 125.8(2), 125.8(0), 125.7(7), 124.9, 123.1, 121.3, 119.5, 116.9, 112.2, 53.7, 50.1, 45.5, 24.2; HRMS (ESI-TOF) *m/z* $[M+H]^+$ Calculated for $C_{23}H_{23}F_3N_5O_3^+$ 474.1748, found 474.1748 (|Δ*m/z*|= 0).

Phthalimide **3l**

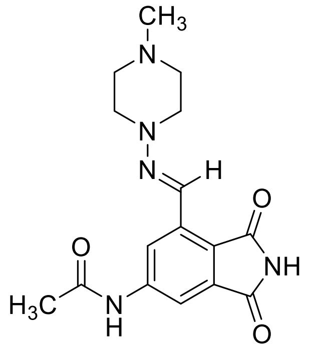

Prepared according to *Procedures F* and *G*, starting with hydrazone **3** (15.0 mg, 0.06 mmol) and maleimide (**7a**, 4.9 mg; 0.05 mmol). Purified by flash chromatography (0.5 L dichloromethane/methanol 0–15%); R_f = 0.20 (dichloromethane/methanol 5%); Yield = 20% (*Procedure F*, 3.3 mg, 0.01 mmol) and 22% (*Procedure G*, 3.6 mg, 0.01 mmol); Beige solid; FTIR (ATR) $\tilde{\nu}$ 3351, 2958, 2852, 2815, 1746, 1694, 1627, 1605, 1569, 1544, 1461, 1420, 1393, 1372, 1364, 1351, 1315, 1281, 1239, 1222, 1165, 1137, 1105, 1072, 1054, 1026, 1019, 997, 973, 944, 916, 907, 896, 877, 850, 824, 805, 794, 757, 738, 686, 677, 664, 638 cm^{-1}; ^{1}H NMR (400 MHz, DMSO-*d6*) δ 10.58 (s, 1H), δ 8.29 (s, 1H), δ 8.14 (d, *J* = 1.7 Hz, 1H), δ 8.06 (d, *J* = 1.7 Hz, 1H), δ 3.20–3.18 (m, 4H), δ 2.52–2.51 (m, 4H), δ 2.24 (s, 3H), δ 2.09 (s, 3H); ^{13}C NMR (100 MHz, DMSO-*d6*) δ 170.3, 169.4, 169.2, 144.1, 135.4, 134.9, 127.6, 121.2, 116.5, 111.8, 53.8, 50.2, 45.5, 24.2; HRMS (ESI-TOF) *m/z* $[M+H]^+$ Calculated for $C_{16}H_{20}N_5O_3^+$ 330.1561, found 330.1555 ($|\Delta m/z|$= 1.8 ppm).

Phthalimide **4**

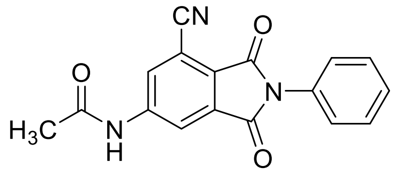

Prepared according to *Procedure H*, starting with phthalimide **3b** (20.3 mg, 0.05 mmol). Purified by flash chromatography (0.5 L dichloromethane/methanol 0–15%); R_f = 0.20 (dichloromethane/methanol 2%); Yield = 66% (10.1 mg, 0.03 mmol); Beige solid; FTIR (ATR) $\tilde{\nu}$ 2644, 2523, 2368, 1670, 1637, 1618, 1585, 1560, 1491, 1400, 1375, 1277, 1261, 1152, 1139, 1123, 1070, 1004, 973, 893, 828, 795, 759, 733, 693, 669, 640 cm^{-1}; ^{1}H NMR (400 MHz, DMSO-*d6*) δ 10.87 (s, 1H), δ 8.38 (d, *J* = 1.8 Hz, 1H), δ 8.29 (d, *J* = 1.8 Hz, 1H), δ 7.56–7.53 (m, 2H), δ 7.48–7.44 (m, 3H), δ 2.17 (s, 3H); ^{13}C NMR (100 MHz, DMSO-*d6*) δ 169.8, 165.3, 164.2, 145.1, 134.2, 131.5, 128.9, 128.3, 127.2, 126.2, 126.0, 116.3, 114.7, 107.0, 24.3; HRMS (ESI-TOF) *m/z* $[M+H]^+$ Calculated for $C_{17}H_{12}N_3O_3^+$ 306.0873, found 306.0871 ($|\Delta m/z|$= 0.7 ppm).

Phthalimide **5**

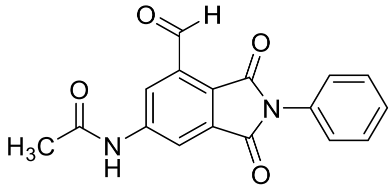

Prepared according to *Procedure I*, starting with phthalimide **3b** (20.3 mg, 0.05 mmol). Purified by flash chromatography (0.5 L dichloromethane/methanol 0–15%); R_f = 0.15 (dichloromethane/methanol 2%); Yield = 54% (8.3 mg, 0.03 mmol); Beige solid; FTIR (ATR) $\tilde{\nu}$ 3333, 3066, 2927, 2368, 1773, 1701, 1618, 1599, 1550, 1485, 1457, 1425, 1370, 1306, 1249, 1205, 1143, 1111, 1068, 1001, 991, 892, 855, 796, 757, 706, 689, 668, 655, 626 cm^{-1}; ^{1}H NMR (400 MHz, DMSO-d_6) δ 10.82 (s, 1H), δ 10.80 (s, 1H), δ 8.47 (d, J = 1.9 Hz, 1H), δ 8.29 (d, J = 1.9 Hz, 1H), δ 7.57–7.53 (m, 2H), δ 7.48–7.45 (m, 3H), δ 2.16 (s, 3H); ^{13}C NMR (100 MHz, DMSO-d_6) δ 188.6, 169.6, 166.2, 166.1, 144.9, 134.1(1), 134.1(0), 131.6, 128.9, 128.3, 127.4, 125.2, 119.2, 116.9, 24.3; HRMS (ESI-TOF) *m/z* $[M+H]^+$ Calculated for $C_{17}H_{13}N_2O_4^+$ 309.0870, found 309.0869 ($|\Delta m/z|$= 0.3 ppm).

Phthalimide **6**

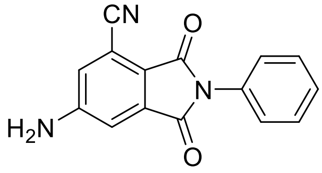

Prepared according to *Procedure J*, starting with phthalimide **3b** (20.3 mg, 0.05 mmol). Purified by flash chromatography (0.5 L dichloromethane/methanol 0–15%); R_f = 0.20 (dichloromethane/methanol 2%); Yield = 40% (5.3 mg, 0.02 mmol); Yellow solid; FTIR (ATR) $\tilde{\nu}$ 3461, 3357, 3236, 2234, 1764, 1716, 1699, 1638, 1630, 1608, 1596, 1581, 1514, 1503, 1459, 1448, 1393, 1374, 1352, 1293, 1248, 1218, 1158, 1133, 1110, 1101, 1072, 1036, 1002, 964, 950, 894, 865, 854, 786, 750, 735, 686, 672 cm^{-1}; ^{1}H NMR (400 MHz, DMSO-d_6) δ 7.51–7.48 (m, 2H), δ 7.42–7.36 (m, 3H), δ 7.01 (d, J = 2.0 Hz, 1H), δ 6.98 (d, J = 2.0 Hz, 1H), δ 6.63 (s, 2H); ^{13}C NMR (100 MHz, DMSO-d_6) δ 167.0, 166.5, 154.9, 138.9, 134.7, 132.2, 128.7, 127.6, 127.3, 114.1, 113.0, 107.2, 99.1; HRMS (ESI-TOF) *m/z* $[M+H]^+$ Calculated for $C_{15}H_{10}N_3O_2^+$ 264.0768, found 264.0768 ($|\Delta m/z|$= 0).

N-(4-Nitrophenyl)maleimide (**7b**)

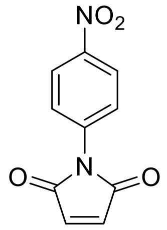

Prepared according to *Procedure K*, starting with 4-nitroaniline (691 mg, 5.0 mmol). Purified by flash chromatography (1.0 L hexane/ethyl acetate 20–80%); R_f = 0.30 (hexane/ethyl acetate 50%); Yield = 27% (295 mg, 1.4 mmol); Light yellow solid; mp = 169–170 °C; FTIR (ATR) $\tilde{\nu}$ 3478, 3120, 3105, 3083, 2974, 2863, 1916, 1782, 1763, 1711, 1599, 1517, 1502, 1387, 1346, 1323, 1301, 1215, 1182, 1141, 1114, 1055, 1029, 954, 846, 824, 753, 731, 697, 682 cm^{-1}; ^{1}H NMR ($CDCl_3$, 400 MHz) δ 8.35–8.31 (m, 2H), δ 7.70–7.66 (m, 2H), δ 6.93 (s, 2H); ^{13}C NMR ($CDCl_3$, 100 MHz) δ 168.6, 146.3, 137.2, 134.7, 125.6, 124.6 ppm.

N-(4-Cyanophenyl)maleimide (**7c**)

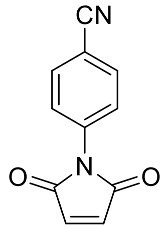

Prepared according to *Procedure K*, starting with 4-aminobenzonitrile (591 mg, 5.0 mmol). Purified by flash chromatography (1.0 L hexane/ethyl acetate 20–80%); R_f = 0.35 (hexane/ethyl acetate 50%); Yield = 51% (505 mg, 2.5 mmol); Off-white solid; mp = 139–140 °C; FTIR (ATR) $\tilde{\nu}$ 3463, 3418, 3209, 3168, 3131, 3094, 3008, 2892, 2822, 2236, 1905, 1797, 1770, 1715, 1603, 1588, 1513, 1413, 1390, 1372, 1316, 1282, 1215, 1178, 1141, 1077, 1029, 950, 839, 827, 786, 764, 719, 686, 660 cm^{-1}; ^{1}H NMR ($CDCl_3$, 400 MHz) δ 7.78–7.75 (m, 2H), δ 7.61–7.58 (m, 2H), δ 6.91 (s, 2H); ^{13}C NMR ($CDCl_3$, 100 MHz) δ 168.7, 135.6, 134.7, 133.1, 125.8, 118.3, 111.3 ppm.

N-(4-(Trifluoromethyl)phenyl)maleimide (**7d**)

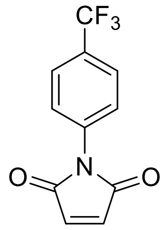

Prepared according to *Procedure K*, starting with 4-(trifluoromethyl)aniline (628 µL mg, 5.0 mmol). Purified by flash chromatography (1.0 L hexane/ethyl acetate 10–50%); R_f = 0.45 (hexane/ethyl acetate 50%); Yield = 49% (590 mg, 2.4 mmol); White solid; mp = 154–155 °C; FTIR (ATR) $\tilde{\nu}$ 3489, 3172, 3112, 3068, 2971, 2933, 2643, 2598, 2557, 2374, 2344, 2289, 1990, 1931, 1916, 1882, 1804, 1782, 1752, 1718, 1703, 1651, 1614, 1580, 1562, 1524, 1439, 1413, 1390, 1331, 1316, 1223, 1185, 1155, 1126, 1103, 1070, 1055, 1036, 1021, 954, 839, 816, 757, 738, 712, 690 cm^{-1}; ^{1}H NMR ($CDCl_3$, 400 MHz) δ 7.74 (d, *J* = 8.4 Hz, 2H), δ 7.55 (d, *J* = 8.4 Hz, 2H), δ 6.90 (s, 2H); ^{19}F NMR ($CDCl_3$, 376 MHz) δ –62.7 ppm; ^{13}C NMR ($CDCl_3$, 100 MHz) δ 169.0, 134.6, 130.3, 130.0, 129.7, 129.3, 127.9, 126.4(5), 126.4(1), 126.3(7), 126.3(4), 125.9, 125.2, 122.5, 119.8 ppm.

N-(4-Iodophenyl)maleimide (**7e**)

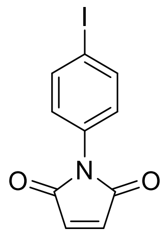

Prepared according to *Procedure K*, starting with 4-iodoaniline (1095 mg, 5.0 mmol). Purified by flash chromatography (1.0 L hexane/ethyl acetate 20–60%); R_f = 0.40 (hexane/ethyl acetate 50%); Yield = 71% (1066 mg, 3.6 mmol); Light yellow solid; mp = 160–161 °C; FTIR (ATR) $\tilde{\nu}$ 3466, 3172, 3097, 3041, 2963, 2538, 2344, 1983, 1897, 1882, 1793, 1767, 1700, 1584, 1562, 1543, 1483, 1446, 1405, 1387, 1293, 1271, 1211, 1170, 1152, 1100, 1051, 1032, 1006, 962, 947, 839, 816, 760, 727, 708, 682 cm^{-1}; ^{1}H NMR ($CDCl_3$, 400 MHz) δ 7.81–7.77 (m, 2H), δ 7.15–7.11 (m, 2H), δ 6.86 (s, 2H); ^{13}C NMR ($CDCl_3$, 100 MHz) δ 169.2, 138.4, 134.5, 131.1, 127.7, 93.1 ppm.

N-(4-Bromophenyl)maleimide (**7f**)

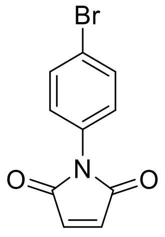

Prepared according to *Procedure K*, starting with 4-bromoaniline (860 mg, 5.0 mmol). Purified by flash chromatography (1.0 L hexane/ethyl acetate 20–80%); R_f = 0.40 (hexane/ethyl acetate 50%); Yield = 61% (771 mg, 3.1 mmol); Yellow solid; mp = 129–130 °C; FTIR (ATR) $\tilde{\nu}$ 3463, 3161, 3109, 3090, 2974, 2878, 2781, 2658, 2516, 2371, 2344, 1990, 1893, 1774, 1707, 1592, 1580, 1543, 1510, 1487, 1442, 1416, 1398, 1383, 1305, 1275, 1208, 1174, 1144, 1062, 1029, 1010, 947, 824, 764, 731, 704, 682 cm^{-1}; ^{1}H NMR ($CDCl_3$, 400 MHz) δ 7.61–7.57 (m, 2H), δ 7.28–7.24 (m, 2H), δ 6.86 (s, 2H); ^{13}C NMR ($CDCl_3$, 100 MHz) δ 169.2, 134.4, 132.4, 130.4, 127.5, 121.8 ppm.

N-(4-Chlorophenyl)maleimide (**7g**)

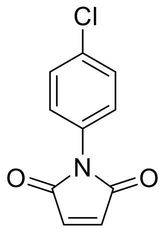

Prepared according to *Procedure K*, starting with 4-chloroaniline (638 mg, 5.0 mmol). Purified by flash chromatography (1.0 L hexane/ethyl acetate 10–60%); R_f = 0.40 (hexane/ethyl acetate 50%); Yield = 65% (674 mg, 3.2 mmol); Light yellow solid; mp = 117–118 °C; FTIR (ATR) $\tilde{\nu}$ 3463, 3165, 3116, 3083, 2982, 2881, 2792, 2516, 2344, 1897, 1774, 1707, 1595, 1584, 1543, 1495, 1454, 1420, 1401, 1383, 1308, 1275, 1211, 1174, 1144, 1096, 1066, 1032, 1018, 965, 947, 831, 764, 745, 708, 682 cm^{-1}; ^{1}H NMR ($CDCl_3$, 400 MHz) δ 7.46–7.42 (m, 2H), δ 7.34–7.30 (m, 2H), δ 6.86 (s, 2H); ^{13}C NMR ($CDCl_3$, 100 MHz) δ 169.3, 134.5, 133.8, 129.9, 129.5, 127.2 ppm.

N-(4-Fluorophenyl)maleimide (**7h**)

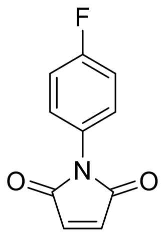

Prepared according to *Procedure K*, starting with 4-fluoroaniline (474 µL, 5.0 mmol). Purified by flash chromatography (1.0 L hexane/ethyl acetate 10–80%); R_f = 0.40 (hexane/ethyl acetate 50%); Yield = 60% (572 mg, 3.0 mmol); Light yellow solid; mp = 155–156 °C; FTIR (ATR) $\tilde{\nu}$ 3455, 3168, 3105, 3071, 2982, 2881, 2523, 2374, 2344, 1987, 1901, 1823, 1774, 1707, 1603, 1584, 1513, 1405, 1390, 1312, 1293, 1264, 1230, 1148, 1088, 1070, 1032, 950, 936, 831, 820, 764, 712, 682 cm^{-1}; ^{1}H NMR ($CDCl_3$, 400 MHz) δ 7.35–7.30 (m, 2H), δ 7.19–7.13 (m, 2H), δ 6.86 (s, 2H); ^{19}F NMR ($CDCl_3$, 376 MHz) δ –113.2 ppm; ^{13}C NMR ($CDCl_3$, 100 MHz) δ 169.5, 163.2, 160.7, 134.4, 128.1, 128.0, 127.3, 127.2, 116.4, 116.2 ppm.

N-Phenylmaleimide (**7i**)

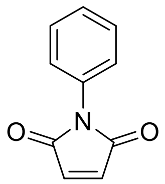

Prepared according to *Procedure K*, starting with aniline (457 µL, 5.0 mmol). Purified by flash chromatography (1.0 L hexane/ethyl acetate 0–15%); R_f = 0.15 (hexane/ethyl acetate 10%); Yield = 52% (453 mg, 2.6 mmol); Yellow solid; mp = 86–87 °C; FTIR (ATR) $\tilde{\nu}$ 3094, 3074, 2986, 2874, 1775, 1702, 1596, 1586, 1501, 1489, 1458, 1376, 1310, 1247, 1208, 1143, 1072, 1031, 949, 908, 829, 755, 693, 626 cm^{-1}; ^{1}H NMR ($CDCl_3$, 400 MHz) δ 7.48–7.45 (m, 2H), δ 7.38–7.32 (m, 3H), δ 6.82 (s, 2H); ^{13}C NMR ($CDCl_3$, 100 MHz) δ 169.6, 134.3, 131.3, 129.2, 128.0, 126.1 ppm.

N-(4-Methylphenyl)maleimide (**7j**)

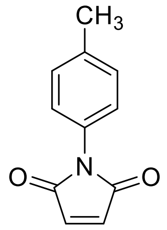

Prepared according to *Procedure K*, starting with 4-toluidine (536 mg, 5.0 mmol). Purified by flash chromatography (1.0 L hexane/ethyl acetate 10–60%); R_f = 0.40 (hexane/ethyl acetate 50%); Yield = 53% (492 mg, 2.6 mmol); Yellow solid; mp = 151–152 °C; FTIR (ATR) $\tilde{\nu}$ 3452, 3168, 3094, 2982, 2926, 2885, 2378, 2344, 1901, 1826, 1770, 1703, 1584, 1513, 1405, 1387, 1312, 1290, 1208, 1178, 1152, 1073, 1036, 1025, 950, 831, 809, 764, 708, 682 cm^{-1}; ^{1}H NMR ($CDCl_3$, 400 MHz) δ 7.27 (d, J = 8.4 Hz, 2H), δ 7.22–7.19 (m, 2H), δ 6.84 (s, 2H), δ 2.38 (s, 3H); ^{13}C NMR ($CDCl_3$, 100 MHz) δ 169.8, 138.2, 134.3, 129.9, 128.7, 126.2, 21.3 ppm.

N-(4-Methoxyphenyl)maleimide (**7k**)

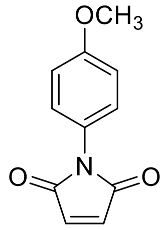

Prepared according to *Procedure K*, starting with 4-anisidine (616 mg, 5.0 mmol). Purified by flash chromatography (1.0 L hexane/ethyl acetate 20–80%); R_f = 0.35 (hexane/ethyl acetate 50%); Yield = 62% (627 mg, 3.1 mmol); Yellow solid; mp = 148–150 °C; FTIR (ATR) $\tilde{\nu}$ 3466, 3172, 3109, 3079, 3012, 2967, 2941, 2915, 2837, 2546, 1987, 1897, 1770, 1700, 1606, 1584, 1506, 1469, 1439, 1413, 1398, 1301, 1249, 1215, 1182, 1148, 1107, 1055, 1025, 950, 939, 827, 816, 798, 764, 719, 682 cm^{-1}; ^{1}H NMR ($CDCl_3$, 400 MHz) δ 7.25–7.21 (m, 2H), δ 7.00–6.96 (m, 2H), δ 6.83 (s, 2H), δ 3.83 (s, 3H); ^{13}C NMR ($CDCl_3$, 100 MHz) δ 170.0, 159.3, 134.3, 127.7, 123.9, 114.7, 55.7 ppm.

N-(4-(Dimethylamino)phenyl)maleimide (**7l**)

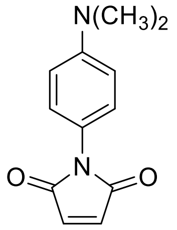

Prepared according to *Procedure K*, starting with 4-(dimethylamino)aniline (681 mg, 5.0 mmol). Purified by flash chromatography (1.0 L hexane/ethyl acetate 20–60%); R_f = 0.40 (hexane/ethyl acetate 50%); Yield = 65% (703 mg, 3.3 mmol); Red solid; mp = 156–157 °C; FTIR (ATR) $\tilde{\nu}$ 3459, 3168, 3097, 3068, 2982, 2892, 2870, 2810, 2374, 2330, 1882, 1852, 1823, 1770, 1696, 1618, 1584, 1524, 1446, 1413, 1398, 1357, 1323, 1301, 1230, 1182, 1152, 1122, 1059, 1032, 939, 827, 805, 760, 719, 682, 663 cm^{-1}; ^{1}H NMR ($CDCl_3$, 400 MHz) δ 7.15–7.11 (m, 2H), δ 6.81 (s, 2H), δ 6.78–6.74 (m, 2H), δ 2.97 (s, 6H); ^{13}C NMR ($CDCl_3$, 100 MHz) δ 170.4, 150.4, 134.2, 127.4, 119.7, 112.7, 40.7 ppm.

N-(Naphthalene-1-yl)maleimide (**7m**)

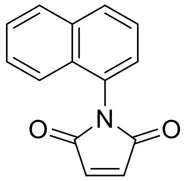

Prepared according to *Procedure K*, starting with 1-naphthylamine (716 mg, 5.0 mmol). Purified by flash chromatography (1.0 L hexane/ethyl acetate 0–30%); R_f = 0.10 (hexane/ethyl acetate 10%); Yield = 86% (960 mg, 4.3 mmol); Yellow solid; mp = 115–118 °C; FTIR (ATR) $\tilde{\nu}$ 3102, 3057, 2983, 2938, 2871, 1775, 1702, 1596, 1560, 1508, 1465, 1402, 1370, 1348, 1232, 1187, 1143, 1044, 1029, 1018, 953, 932, 908, 828, 800, 774, 710, 693, 682, 656, 628, 621 cm^{-1}; ^{1}H NMR ($CDCl_3$, 400 MHz) δ 7.98–7.93 (m, 2H), δ 7.58–7.54 (m, 4H), δ 7.38 (dd, *J* = 7.3, 1.0 Hz, 1H), δ 6.92 (s, 2H); ^{13}C NMR ($CDCl_3$, 100 MHz) δ 170.1, 134.6, 134.5, 130.4, 130.0, 128.7, 127.7, 127.3, 127.1, 126.7, 125.5, 122.3 ppm.

N-Benzylmaleimide (**7n**)

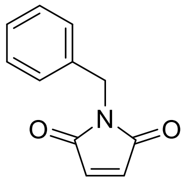

Prepared according to *Procedure K*, starting with 1-benzylamine (546 µL, 5.0 mmol). Purified by flash chromatography (1.0 L hexane/ethyl acetate 0–20%); R_f = 0.20 (hexane/ethyl acetate 10%); Yield = 60% (562 mg, 3.0 mmol); Pale yellow solid; mp = 69–71 °C; FTIR (ATR) $\tilde{\nu}$ 3093, 3070, 3040, 2949, 1763, 1696, 1497, 1458, 1435, 1400, 1385, 1351, 1340, 1310, 1290, 1208, 1158, 1137, 1079, 1033, 919, 880, 839, 781, 721, 692, 643, 623 cm^{-1}; ^{1}H NMR ($CDCl_3$, 400 MHz) *δ* 7.34–7.24 (m, 5H), *δ* 6.69 (s, 2H), *δ* 4.67 (s, 2H); ^{13}C NMR ($CDCl_3$, 100 MHz) *δ* 170.5, 136.3, 134.3, 128.8, 128.4, 127.9, 41.5 ppm.

N-(2-(1*H*-indol-3-yl)ethyl)maleimide (**7o**)

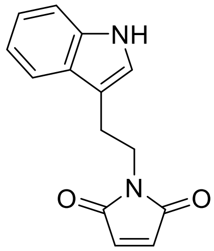

Prepared according to *Procedure K*, starting with tryptamine (801 mg, 5.0 mmol). Purified by flash chromatography (1.0 L hexane/ethyl acetate 0–40%); R_f = 0.20 (hexane/ethyl acetate 25%); Yield = 66% (795 mg, 3.3 mmol); Pale green solid; mp = 132–134 °C; FTIR (ATR) $\tilde{\nu}$ 3342, 3294, 3087, 3016, 2979, 2939, 2919, 2855, 1769, 1745, 1690, 1621, 1583, 1552, 1495, 1451, 1444, 1407, 1367, 1355, 1340, 1328, 1284, 1264, 1232, 1178, 1157, 1139, 1123, 1100, 1048, 1010, 993, 975, 952, 924, 828, 795, 743, 693, 653, 639, 621, 600 cm^{-1}; ^{1}H NMR ($CDCl_3$, 400 MHz) *δ* 8.06 (br s, 1H), *δ* 7.68–7.66 (m, 1H), *δ* 7.35–7.33 (m, 1H), *δ* 7.21–7.18 (m, 1H), *δ* 7.15–7.13 (m, 1H), *δ* 7.03 (d, *J* = 2.3 Hz, 1H), *δ* 6.64 (s, 2H), *δ* 3.86–3.83 (m, 2H), *δ* 3.08–3.06 (m, 2H); ^{13}C NMR ($CDCl_3$, 100 MHz) *δ* 170.9, 136.3, 134.1, 127.5, 122.2, 122.1, 119.6, 118.9, 112.3, 111.3, 38.6, 24.5 ppm.

N-(1-Adamantyl)maleimide (**7p**)

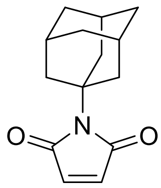

Prepared according to *Procedure K*, starting with 1-adamantylamine (756 mg, 5.0 mmol). Purified by flash chromatography (1.0 L hexane/ethyl acetate 0–10%); R_f = 0.45 (hexane/ethyl acetate 10%); Yield = 29% (333 mg, 1.4 mmol); White solid; mp = 114–116 °C; FTIR (ATR) $\tilde{\nu}$ 3111, 3088, 2908, 2889, 2848, 1705, 1685, 1480, 1455, 1394, 1343, 1308, 1197, 1140, 1118, 1100, 1054, 1023, 997, 973, 934, 850, 829, 769, 695, 681, 643, 633 cm^{-1}; ^{1}H NMR ($CDCl_3$, 400 MHz) δ 6.46 (s, 2H), δ 2.35 (d, J = 2.8 Hz, 6H), δ 2.10 (m, 3H), δ 1.73–1.71 (m, 3H), δ 1.67–1.64 (m, 3H); ^{13}C NMR ($CDCl_3$, 100 MHz) δ 172.4, 133.8, 59.9, 40.3, 36.2, 29.8 ppm.

NMR Spectra

Figure S4. ^{1}H NMR Spectrum (400 MHz, DMSO-d_6) for 3-acetamido-5-acetylfuran (**3A5AF**)

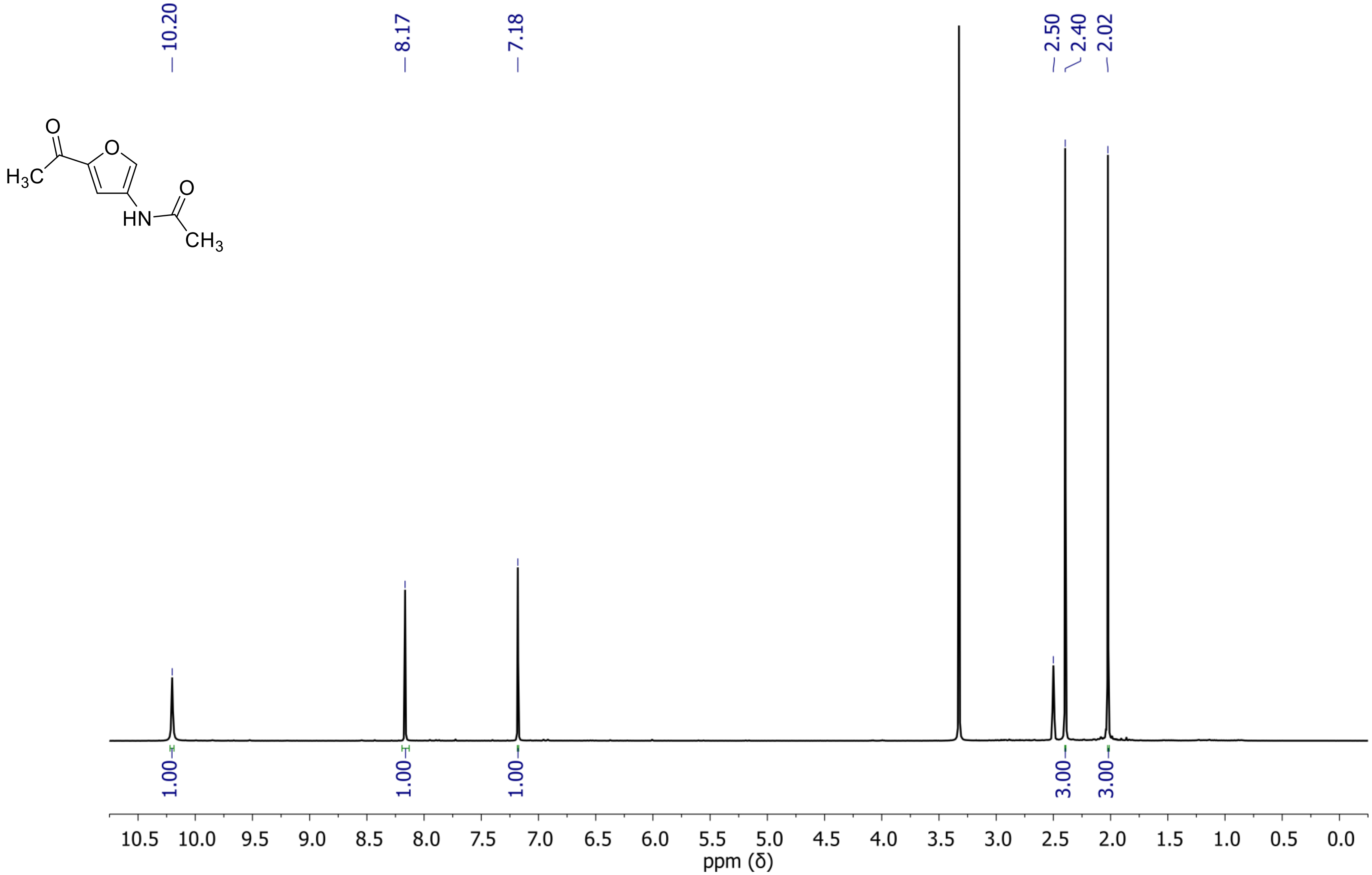

Figure S5. ^{13}C NMR Spectrum (100 MHz, DMSO-d_6) for 3-acetamido-5-acetylfuran (**3A5AF**)

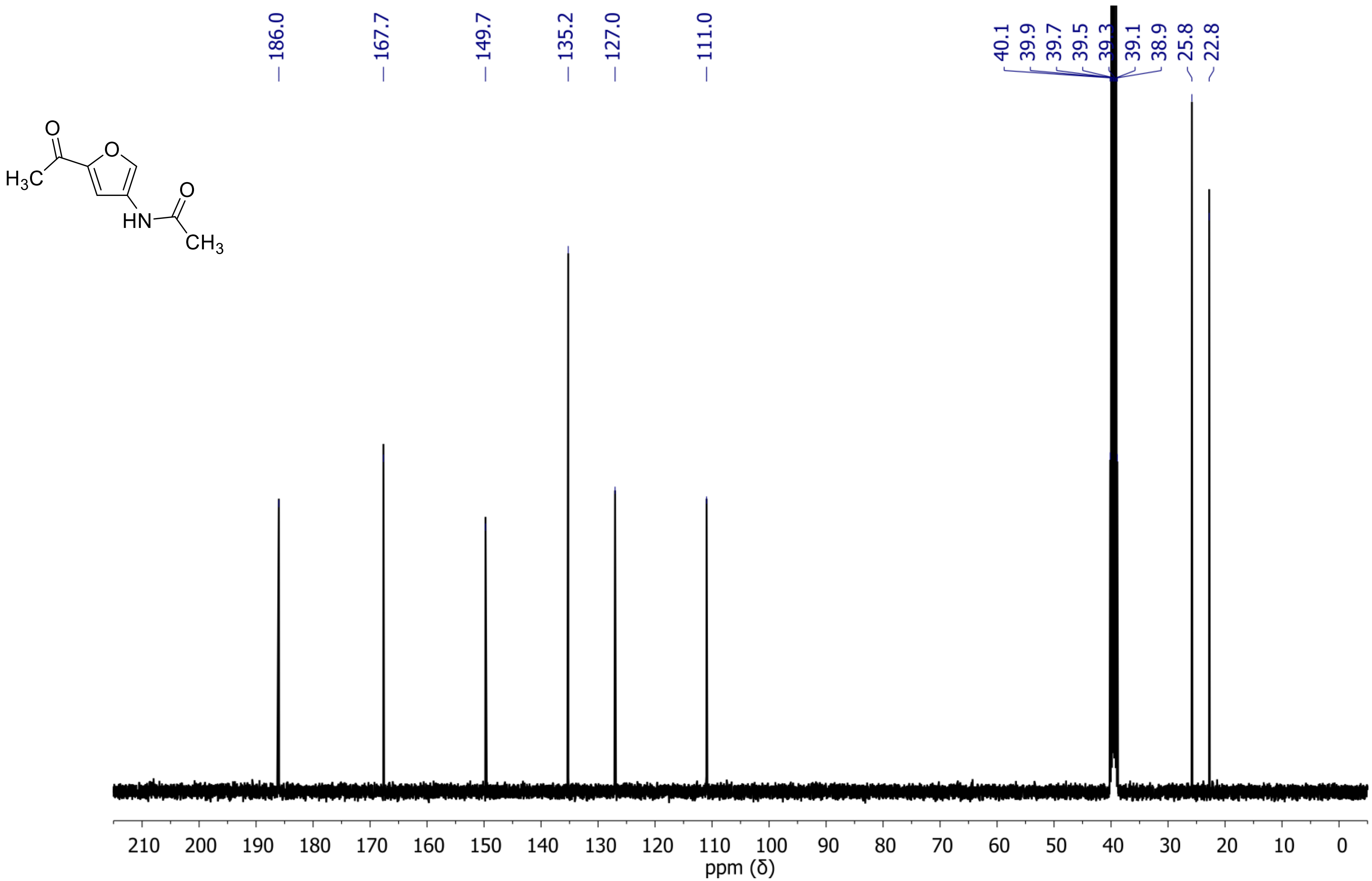

Figure S6. ^{1}H NMR Spectrum (400 MHz, DMSO-d_6) for Diels–Alder adduct **1a**

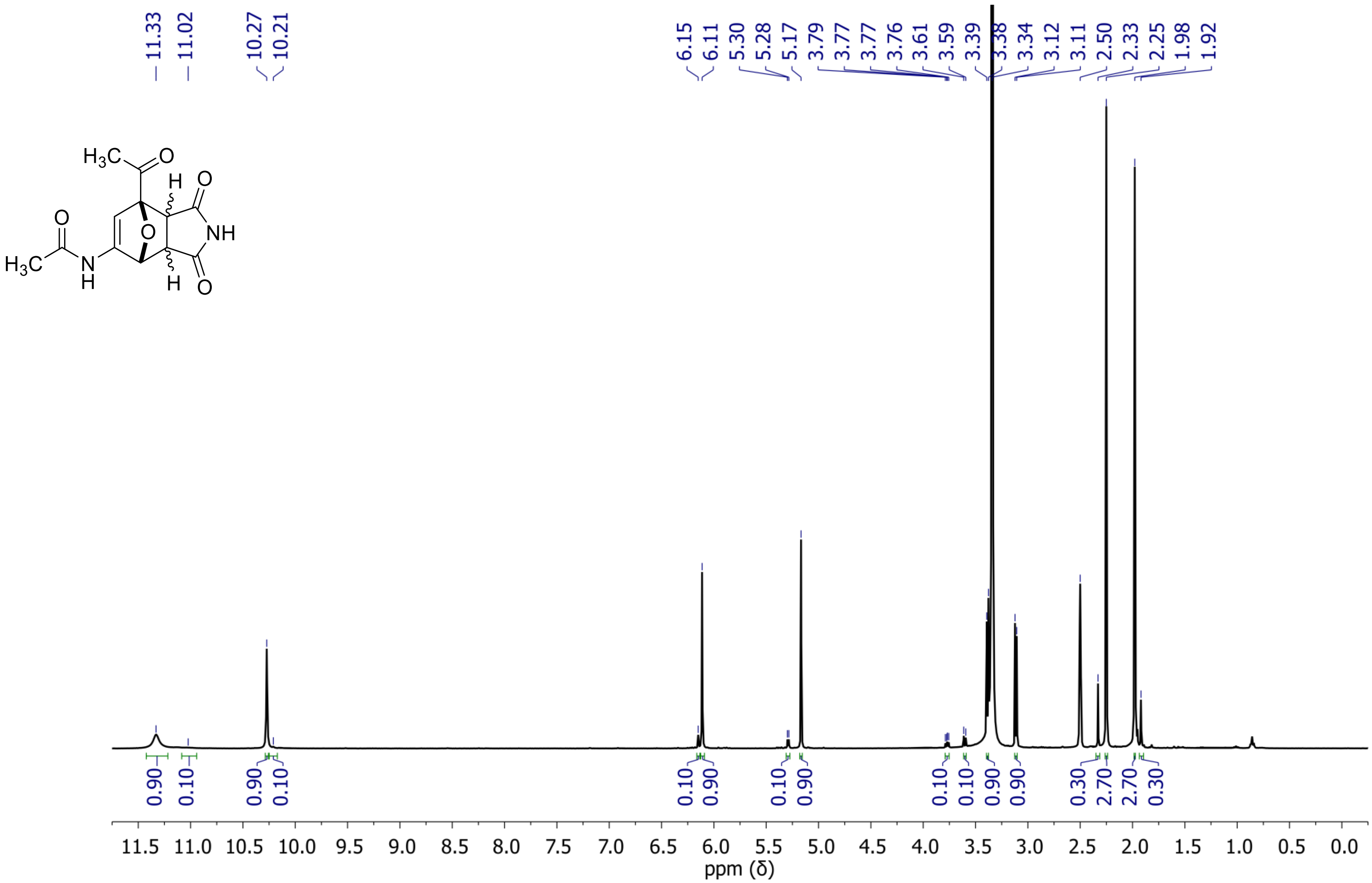

Figure S7. ^{13}C NMR Spectrum (100 MHz, DMSO-d_6) for Diels–Alder adduct **1a**

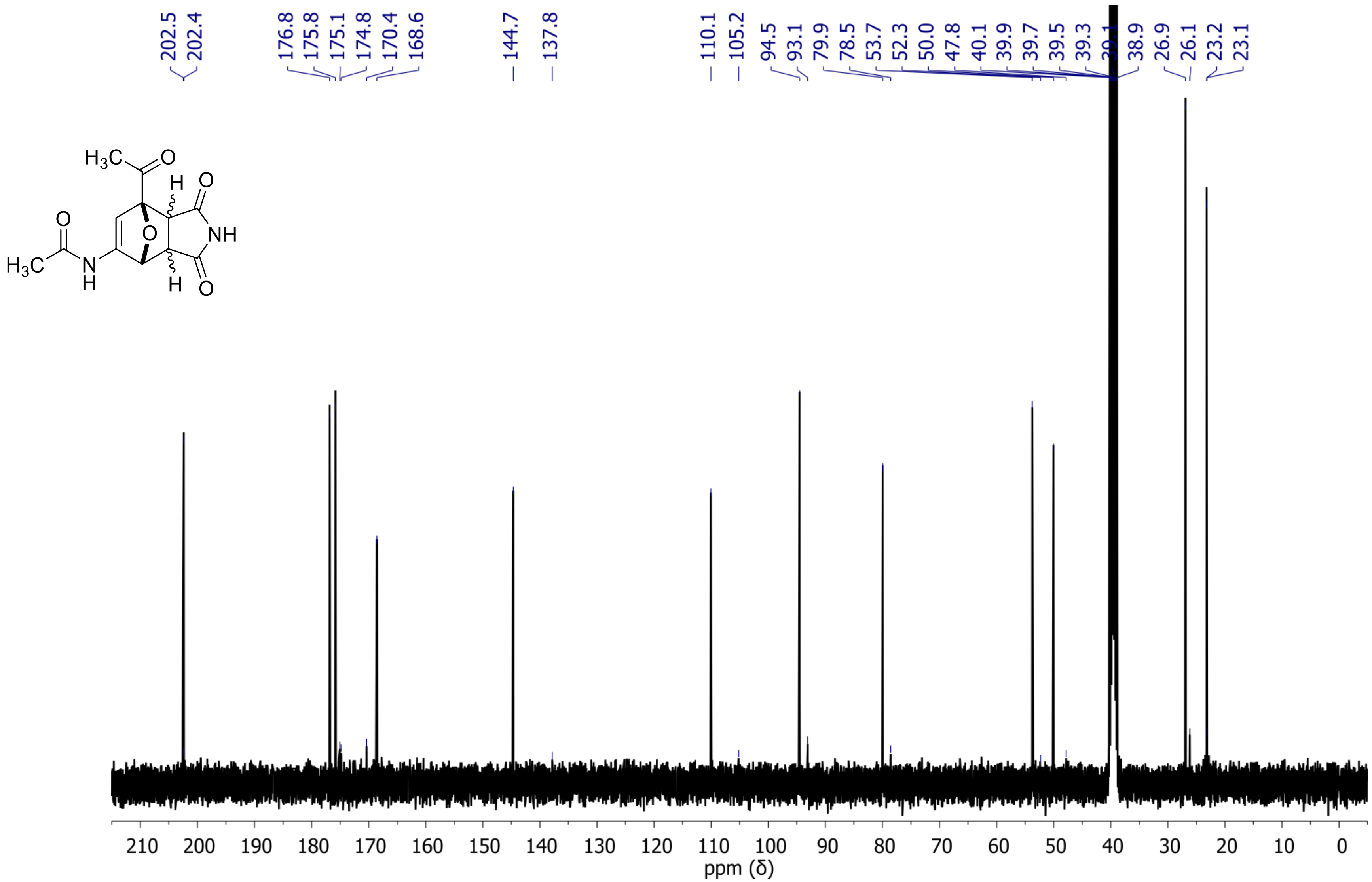

Figure S8. ^{1}H NMR Spectrum (400 MHz, DMSO-d_6) for Diels–Alder adduct **1b**

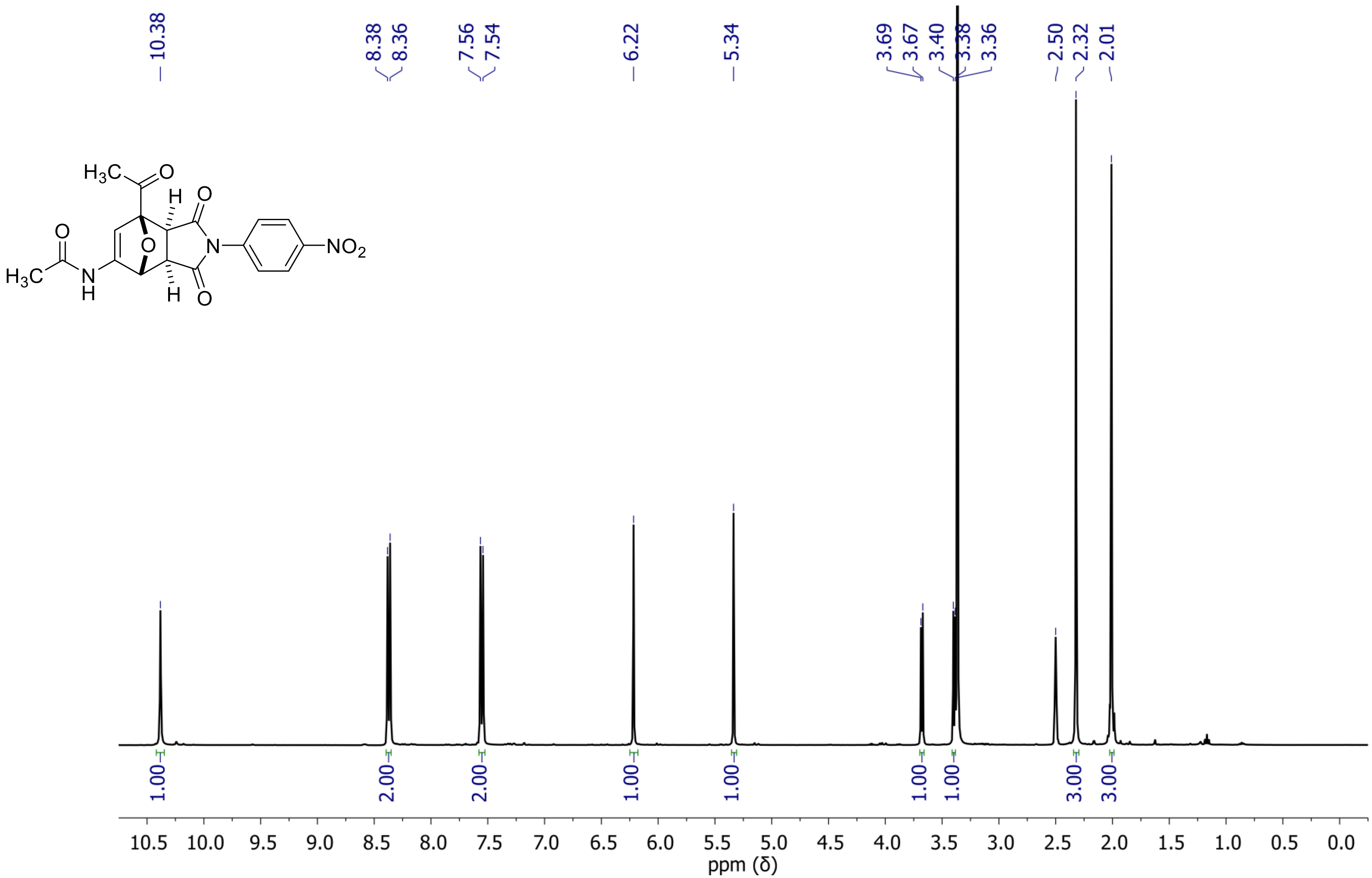

Figure S9. ^{13}C NMR Spectrum (100 MHz, DMSO-d_6) for Diels–Alder adduct **1b**

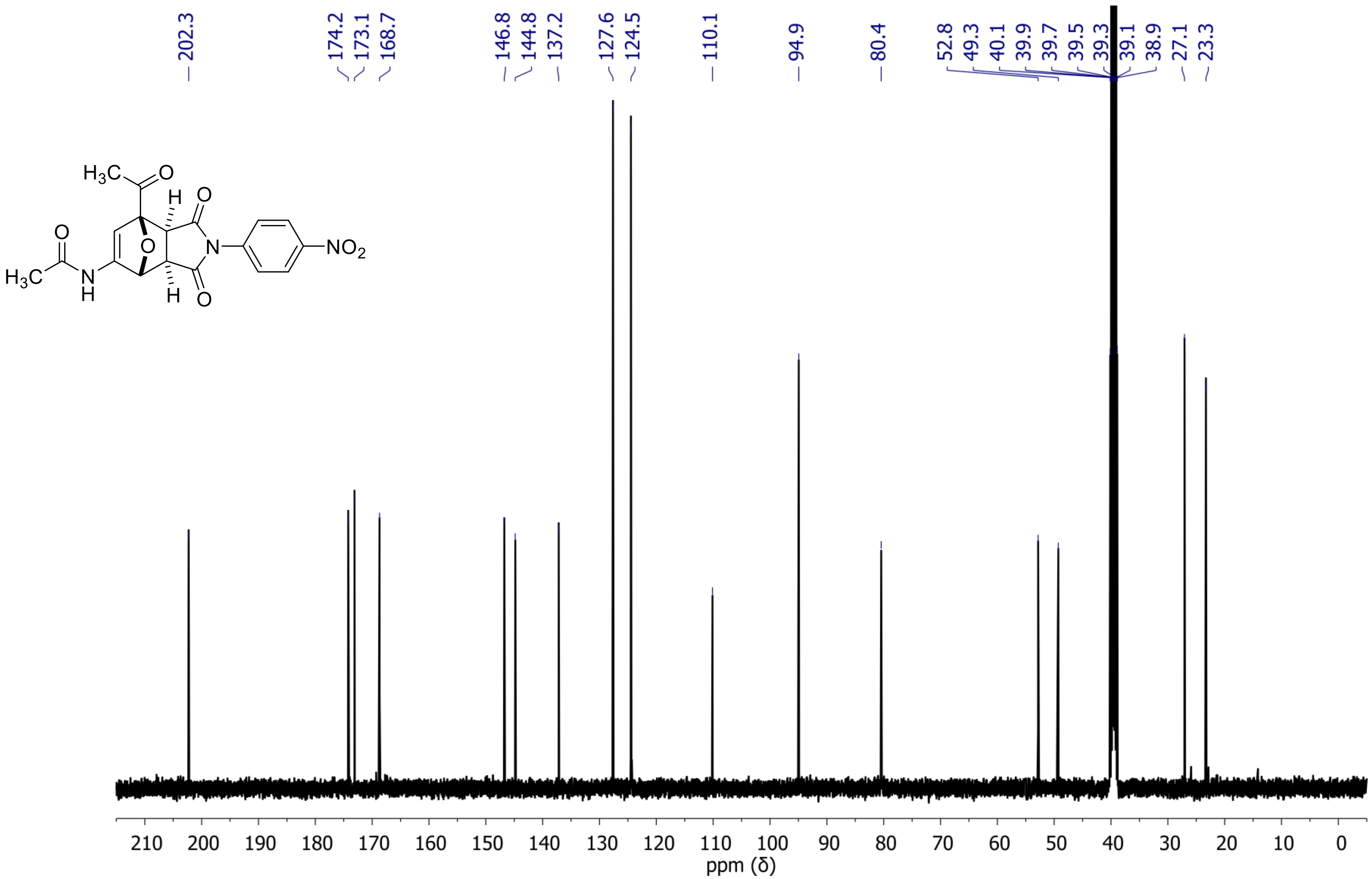

Figure S10. ^{1}H NMR Spectrum (400 MHz, DMSO-d_6) for Diels–Alder adduct **1c**

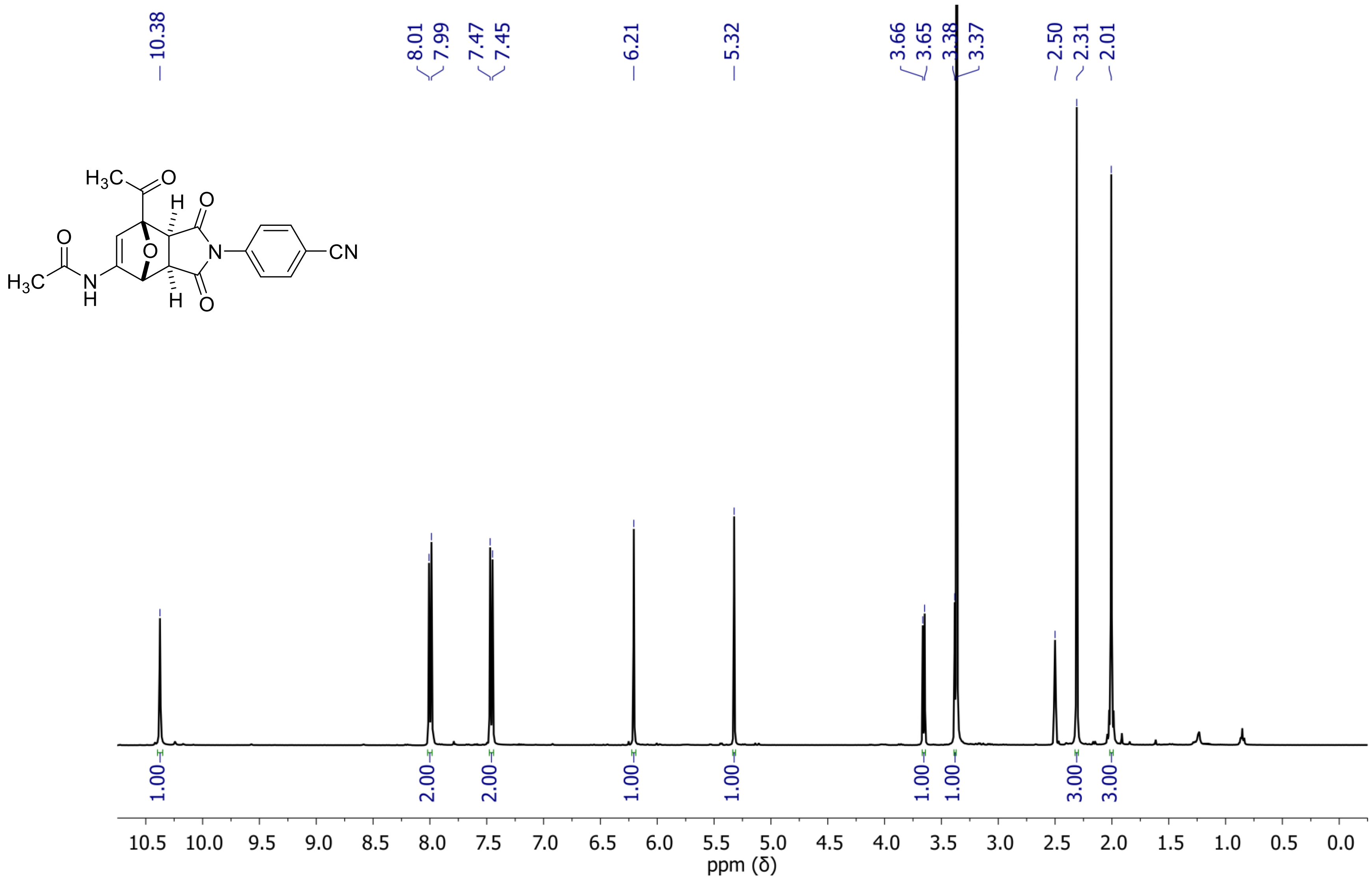

Figure S11. ^{13}C NMR Spectrum (100 MHz, DMSO-d_6) for Diels–Alder adduct **1c**

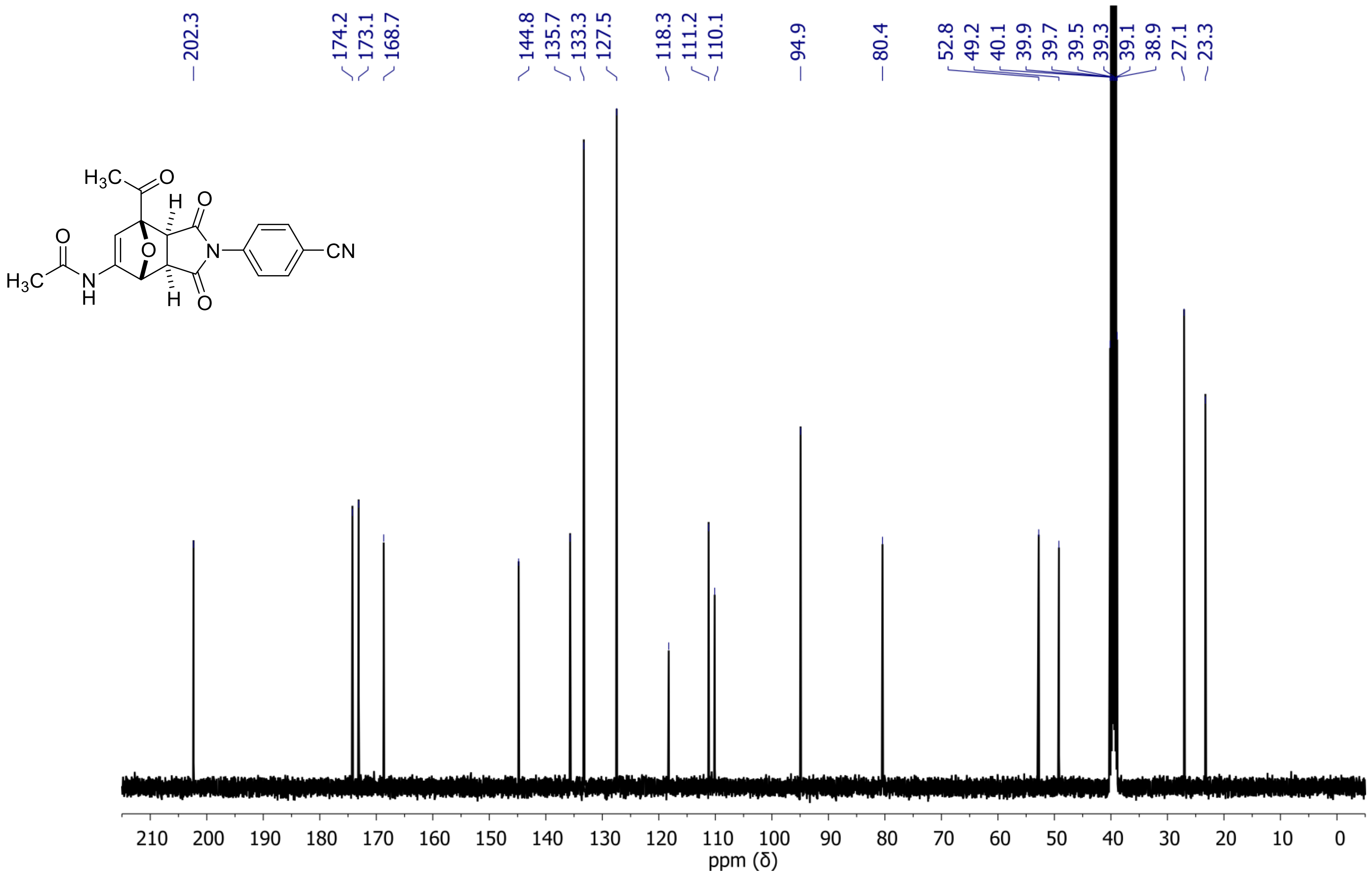

Figure S12. ^{1}H NMR Spectrum (400 MHz, DMSO-d_6) for Diels–Alder adduct **1d**

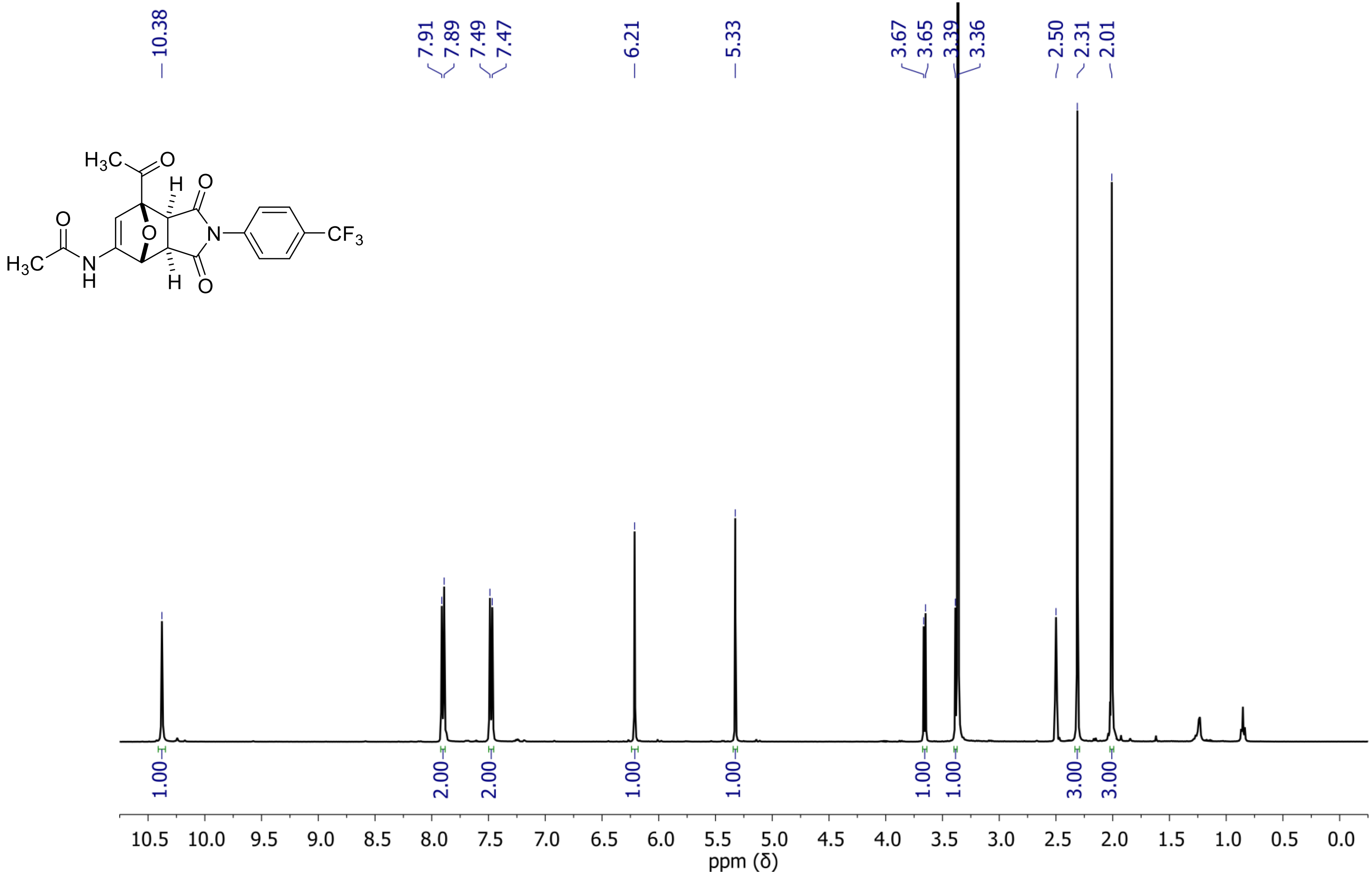

Figure S13. ^{19}F NMR Spectrum (376 MHz, DMSO-d_6) for Diels–Alder adduct **1d**

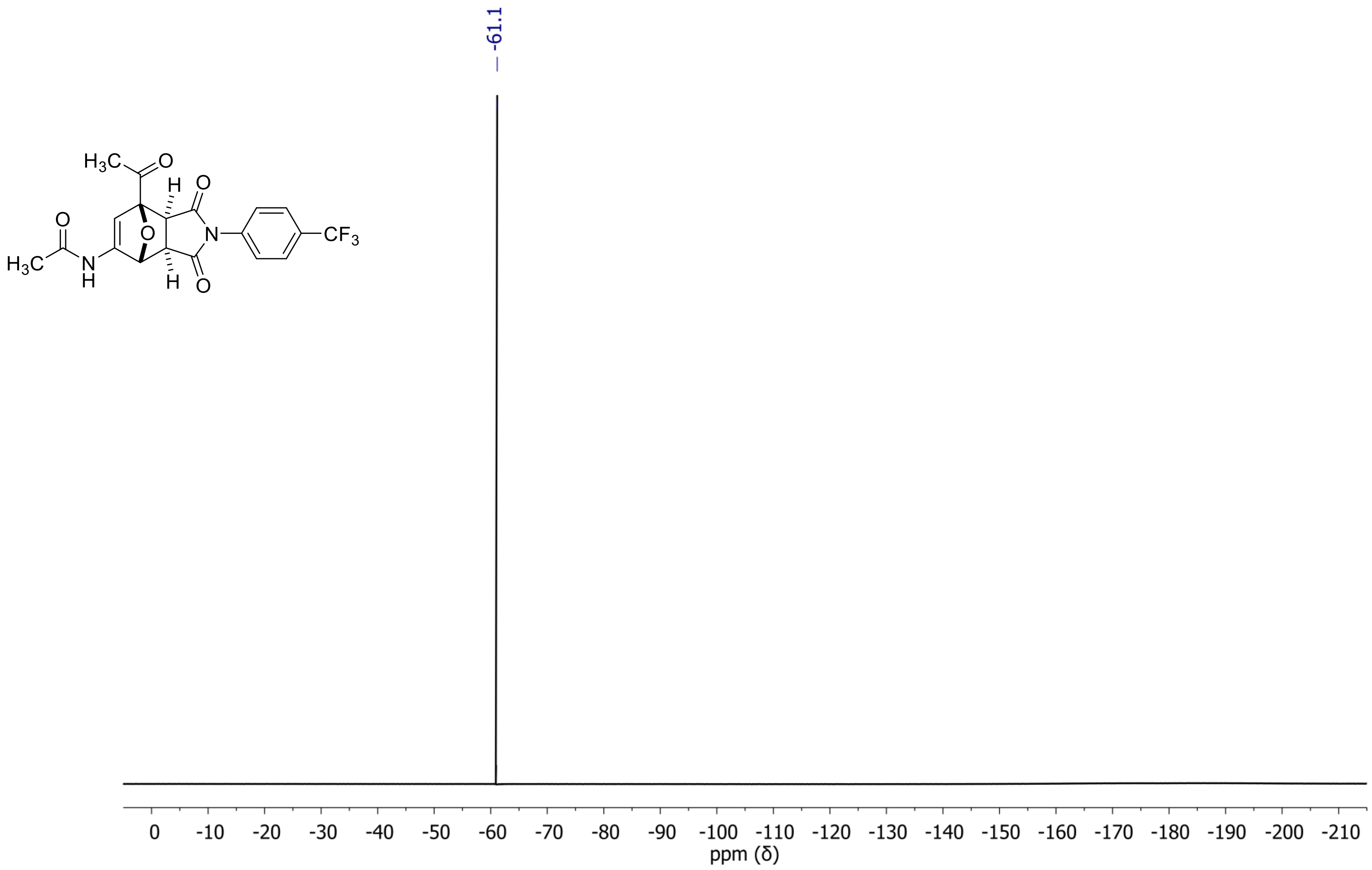

Figure S14. ^{13}C NMR Spectrum (100 MHz, DMSO-d_6) for Diels–Alder adduct **1d**

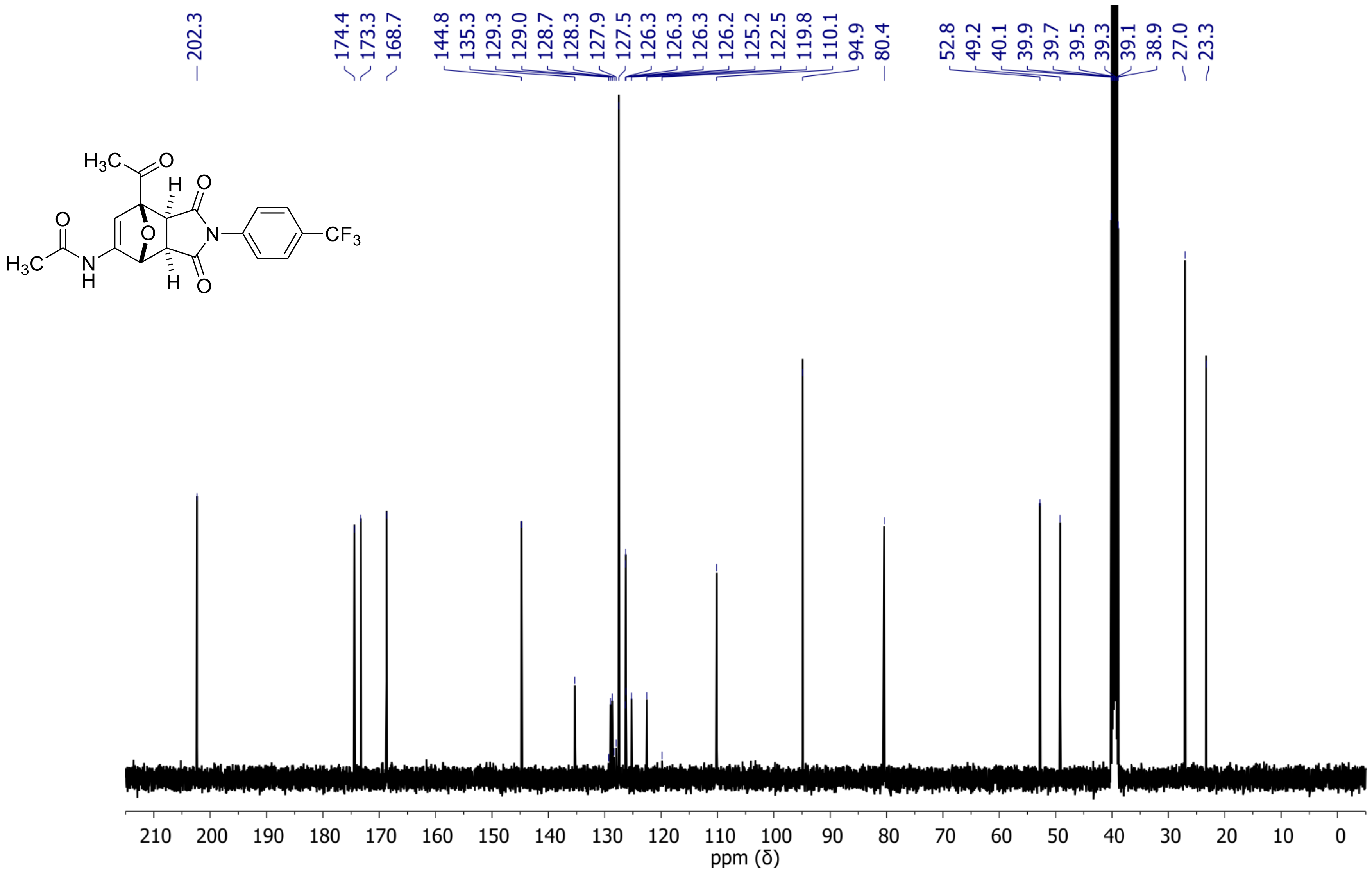

Figure S15. ^{1}H NMR Spectrum (400 MHz, DMSO-d_6) for Diels–Alder adduct **1e**

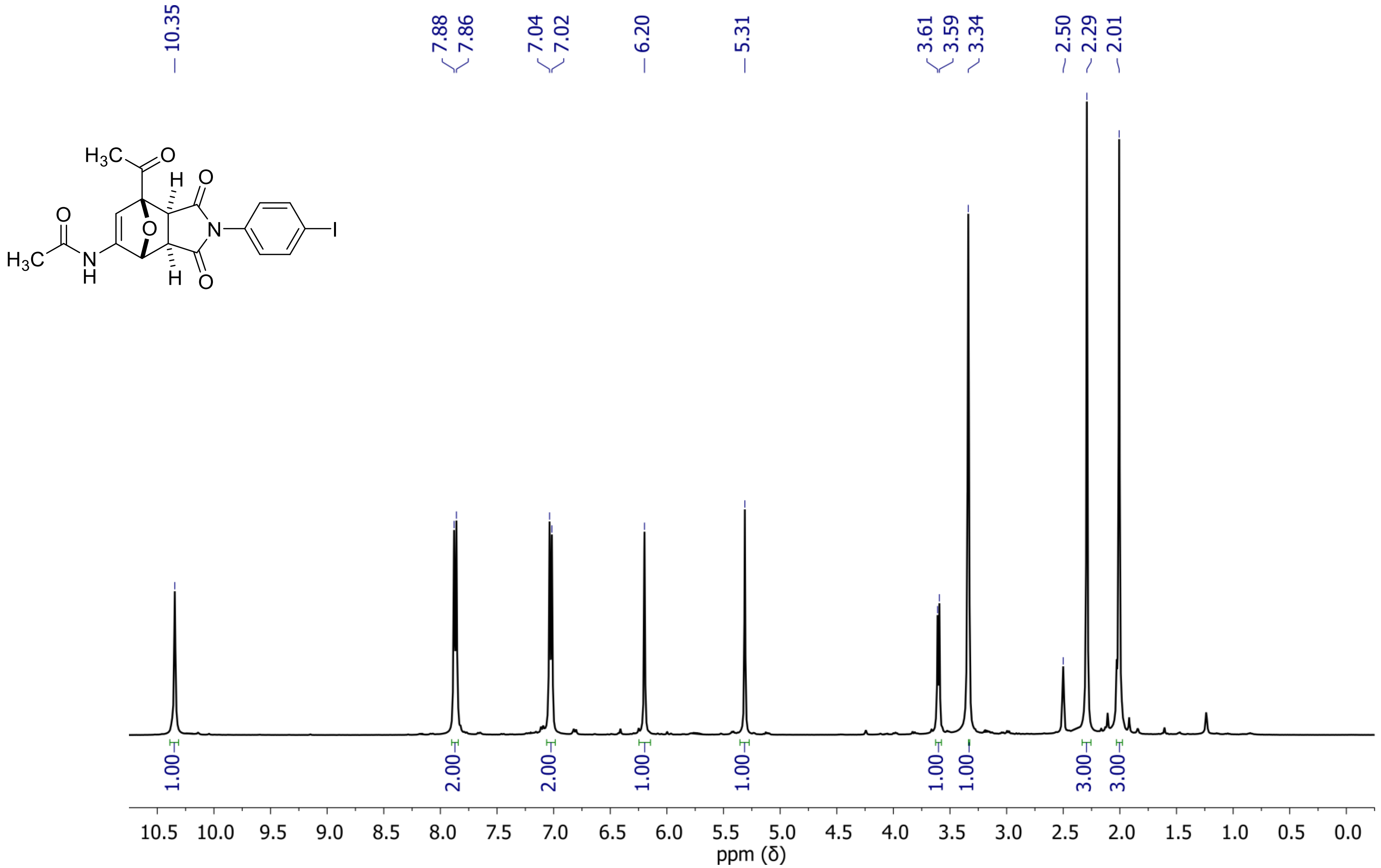

Figure S16. ^{13}C NMR Spectrum (100 MHz, DMSO-d_6) for Diels–Alder adduct **1e**

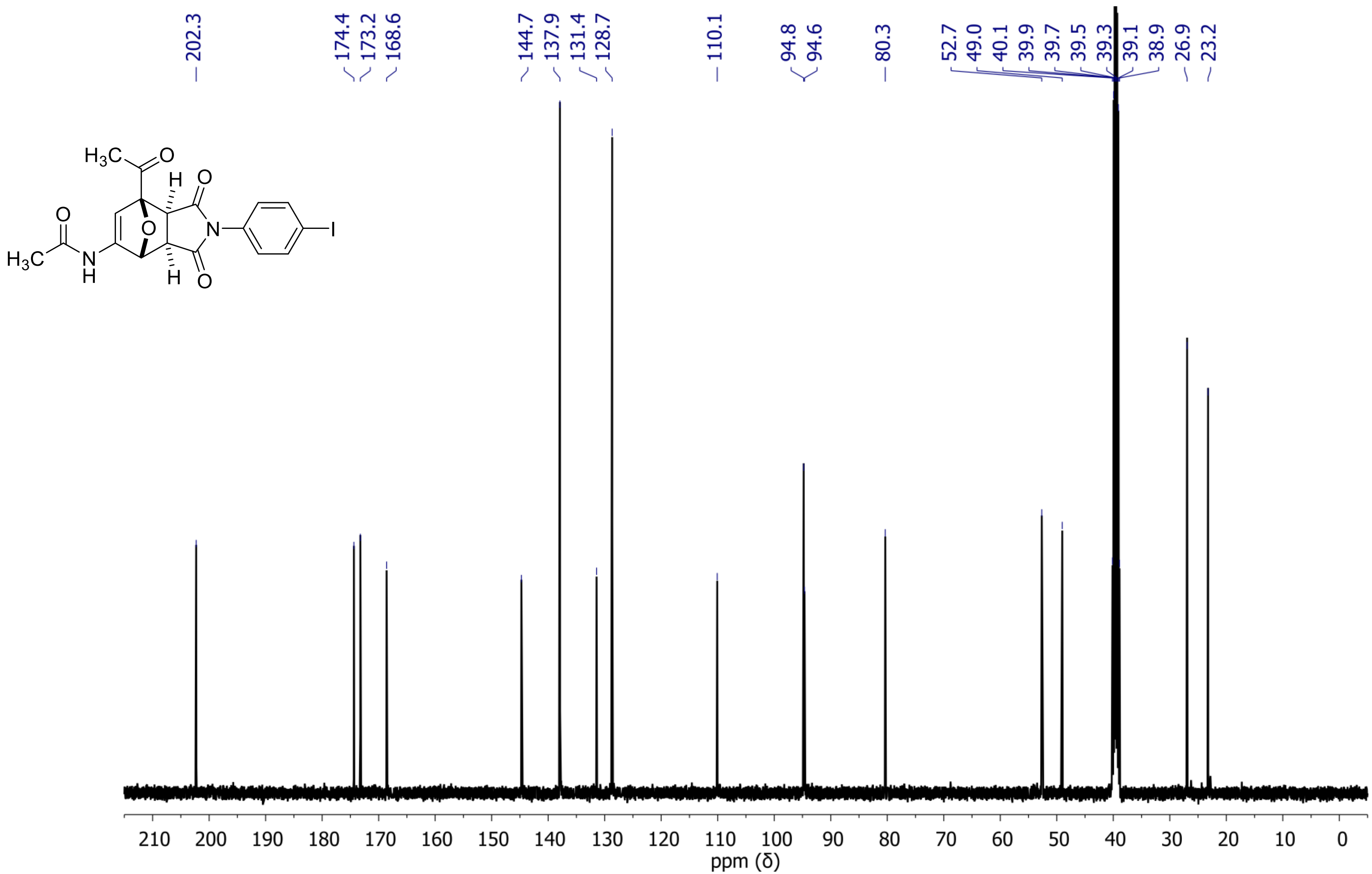

Figure S17. ^{1}H NMR Spectrum (400 MHz, DMSO-d_6) for Diels–Alder adduct **1f**

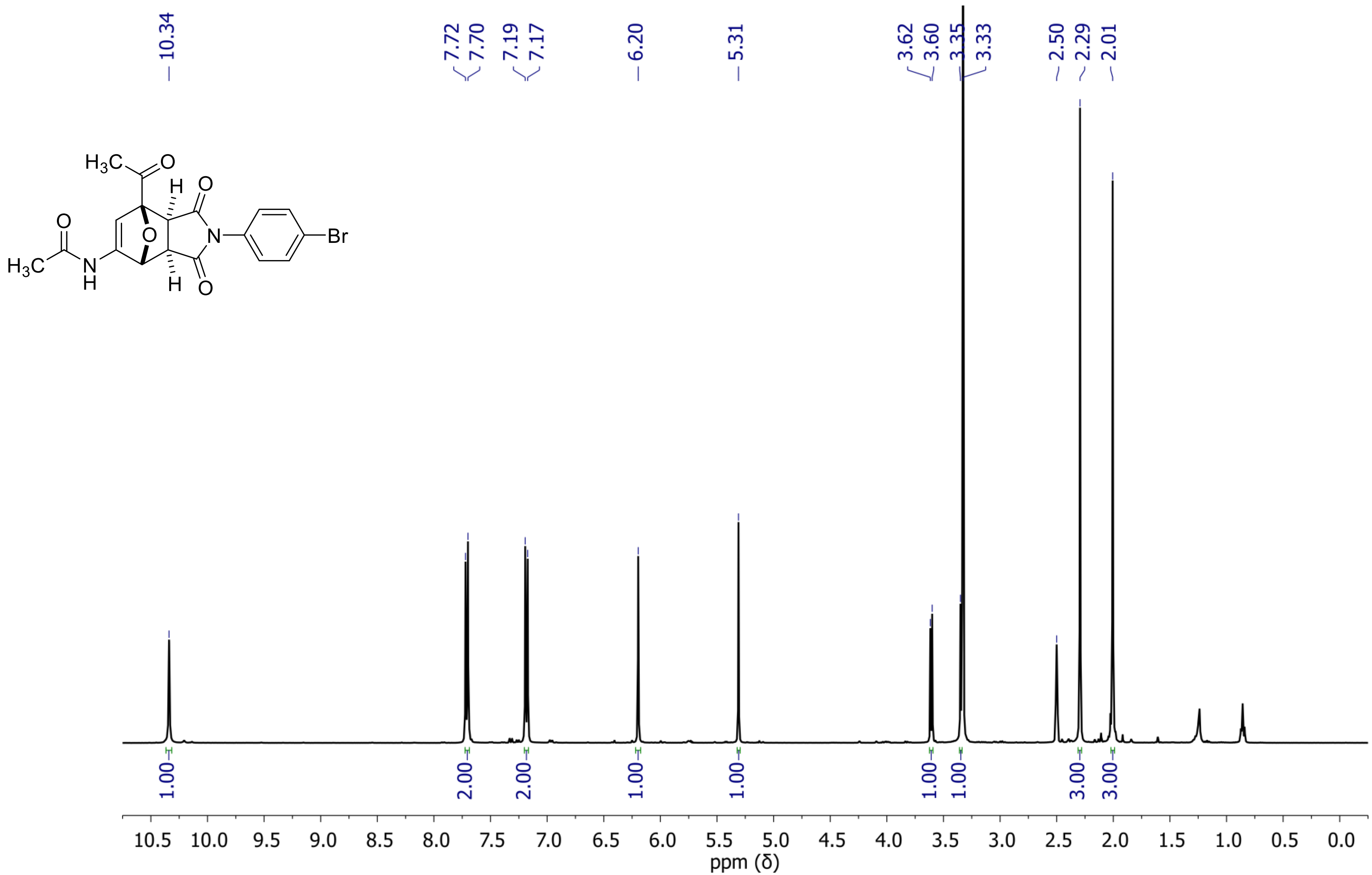

Figure S18. ^{13}C NMR Spectrum (100 MHz, DMSO-d_6) for Diels–Alder adduct **1f**

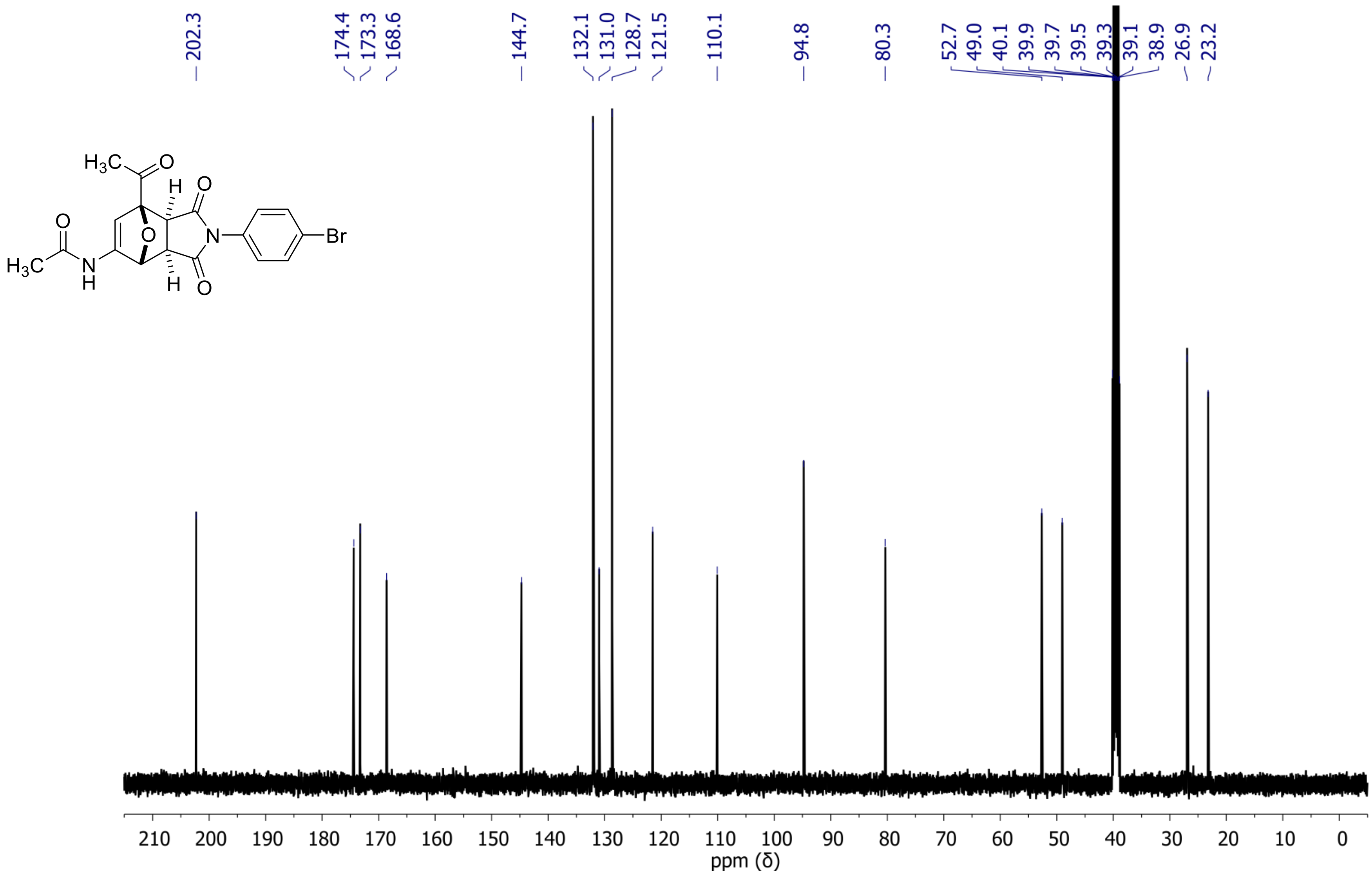

Figure S19. ^{1}H NMR Spectrum (400 MHz, DMSO-d_6) for Diels–Alder adduct **1g**

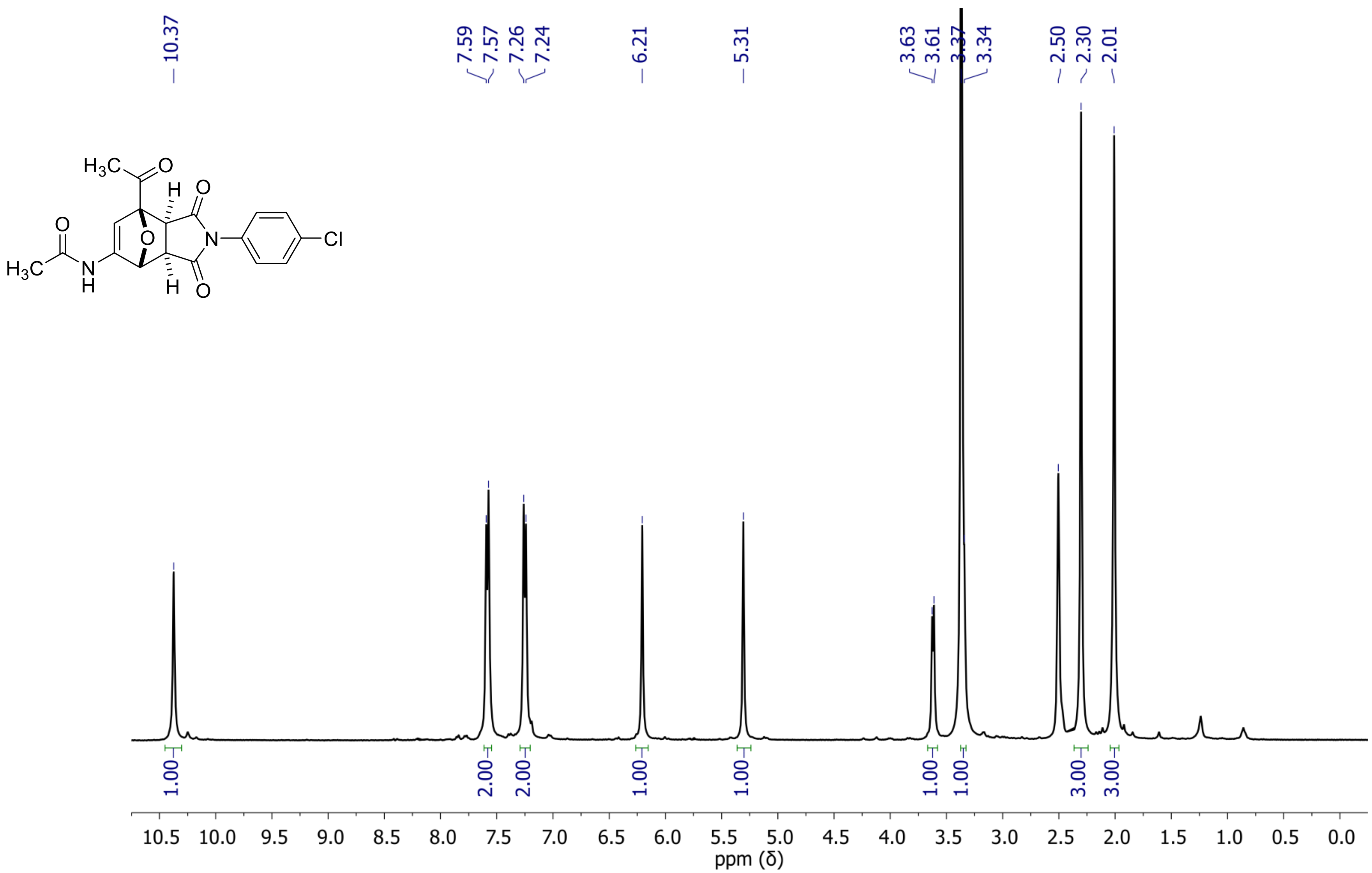

Figure S20. ^{13}C NMR Spectrum (100 MHz, DMSO-d_6) for Diels–Alder adduct **1g**

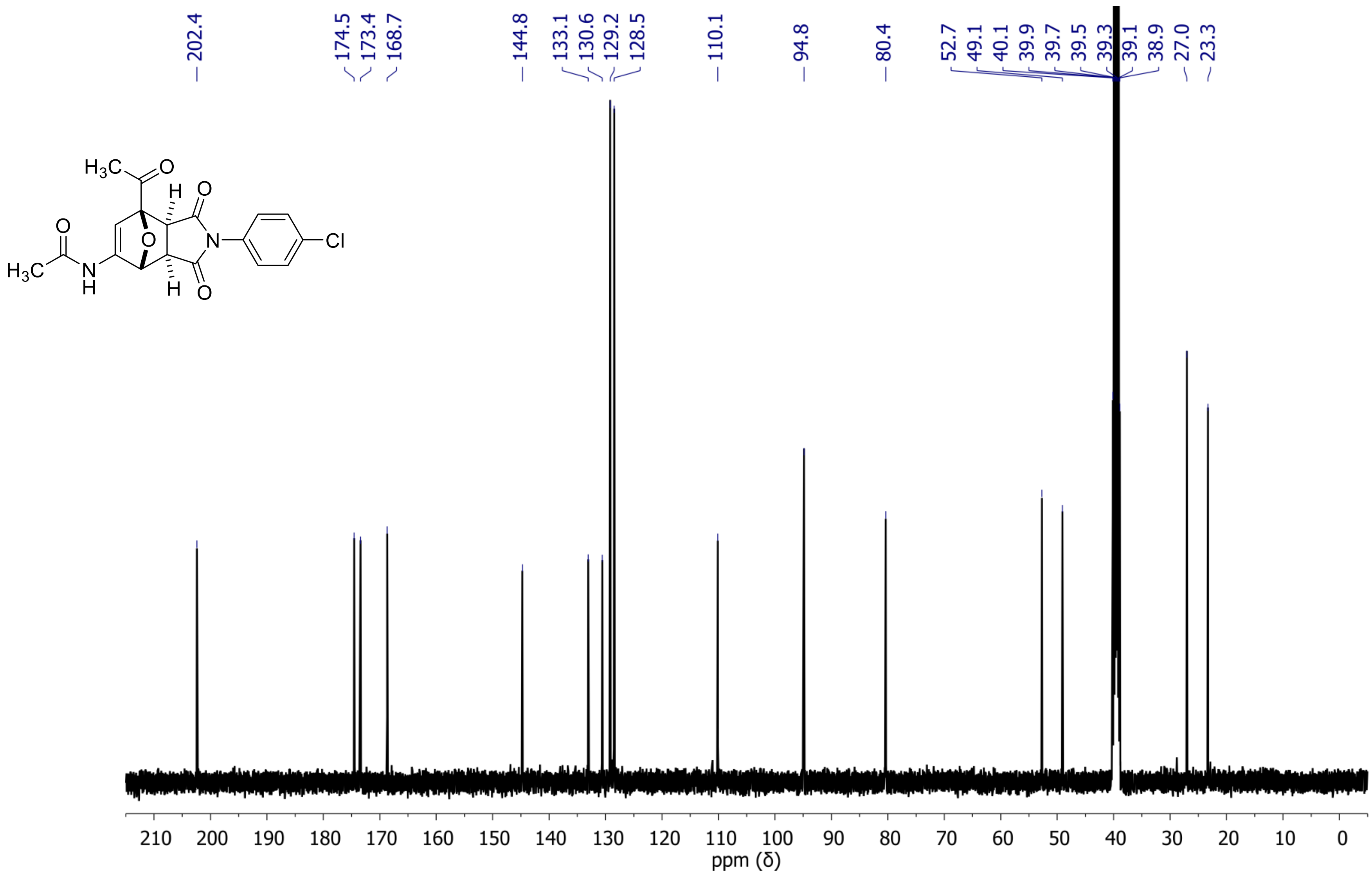

Figure S21. ^{1}H NMR Spectrum (400 MHz, DMSO-d_6) for Diels–Alder adduct **1h**

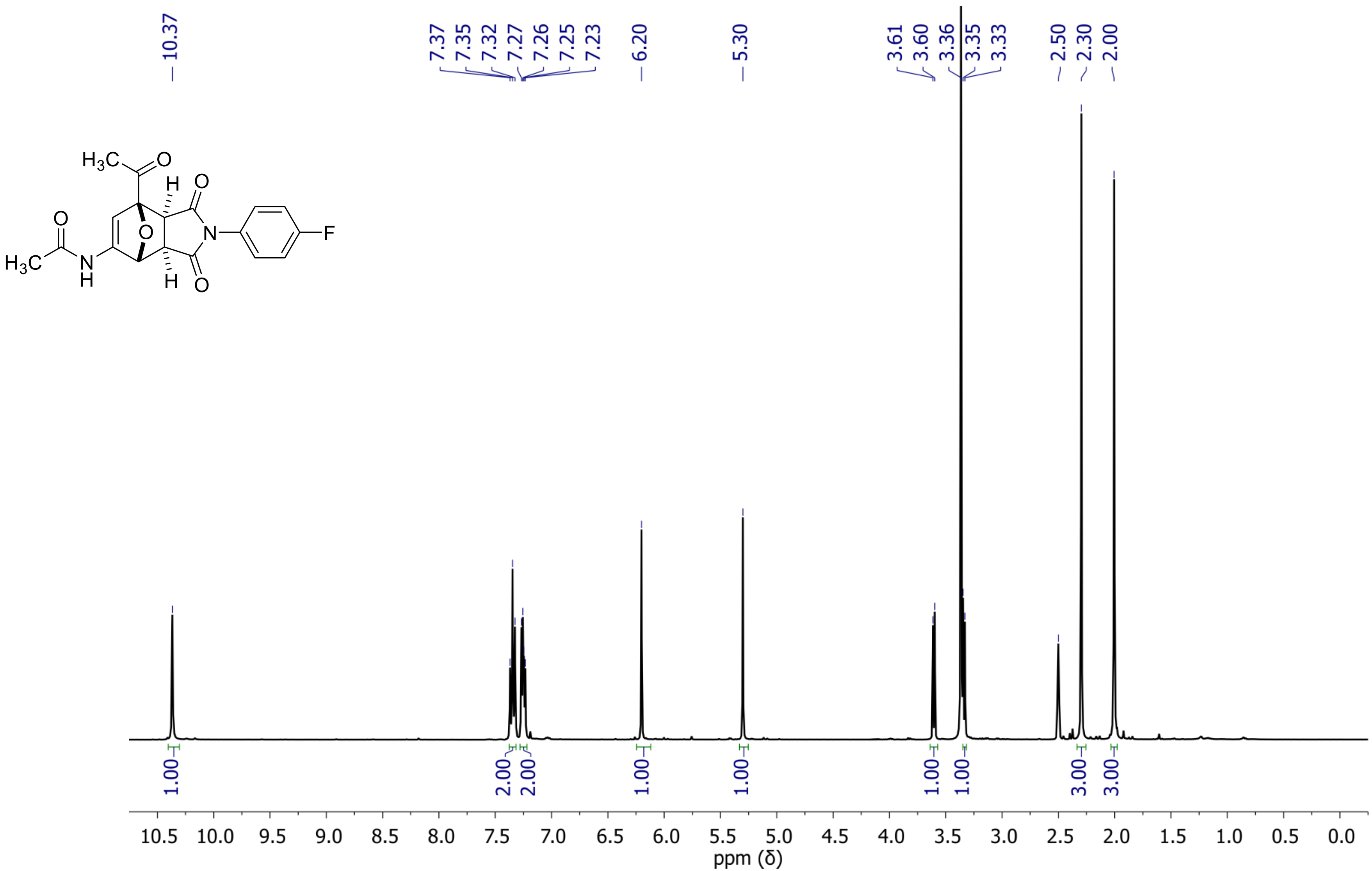

Figure S22. ^{19}F NMR Spectrum (376 MHz, DMSO-d_6) for Diels–Alder adduct **1h**

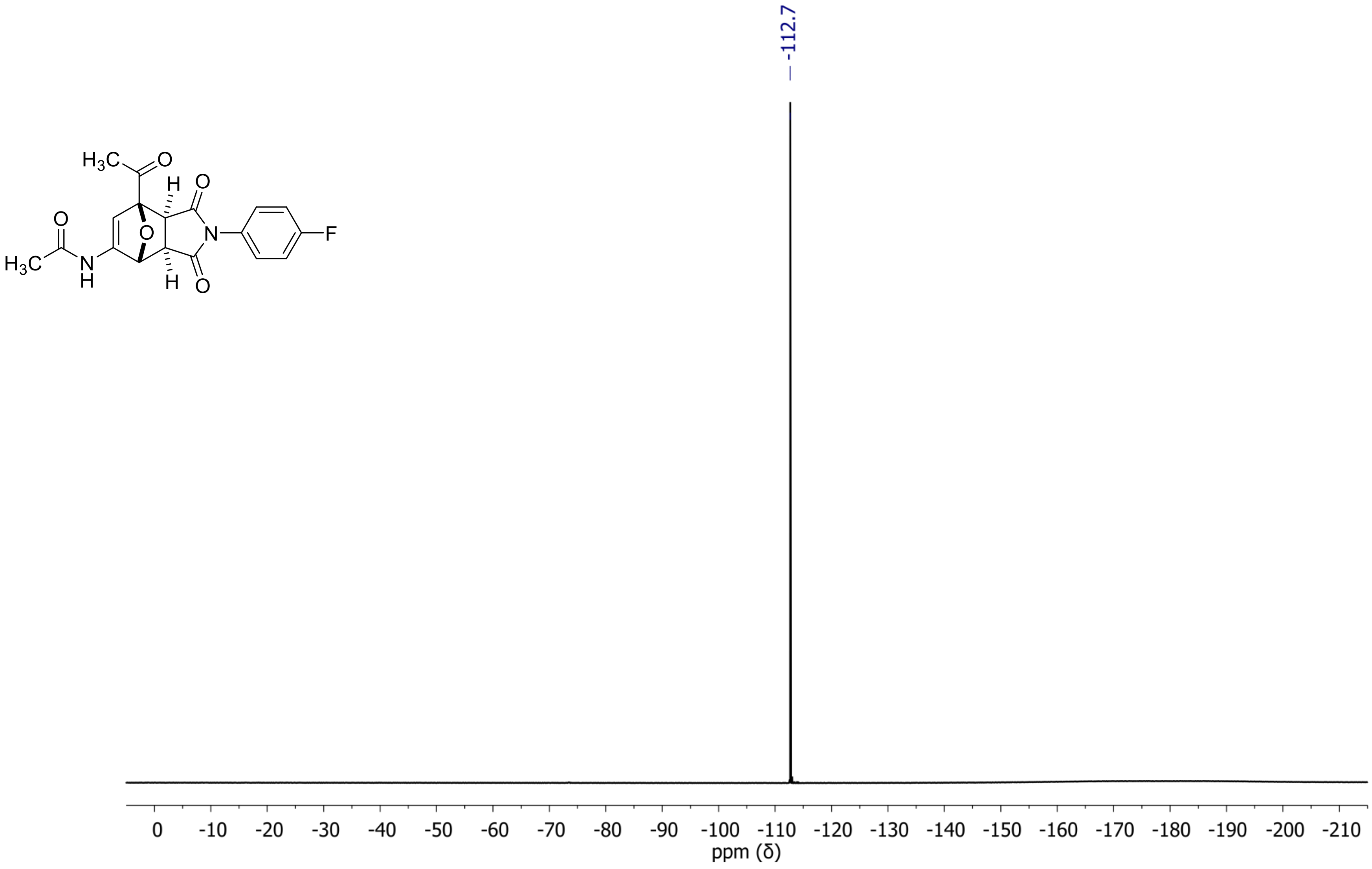

Figure S23. ^{13}C NMR Spectrum (100 MHz, DMSO-d_6) for Diels–Alder adduct **1h**

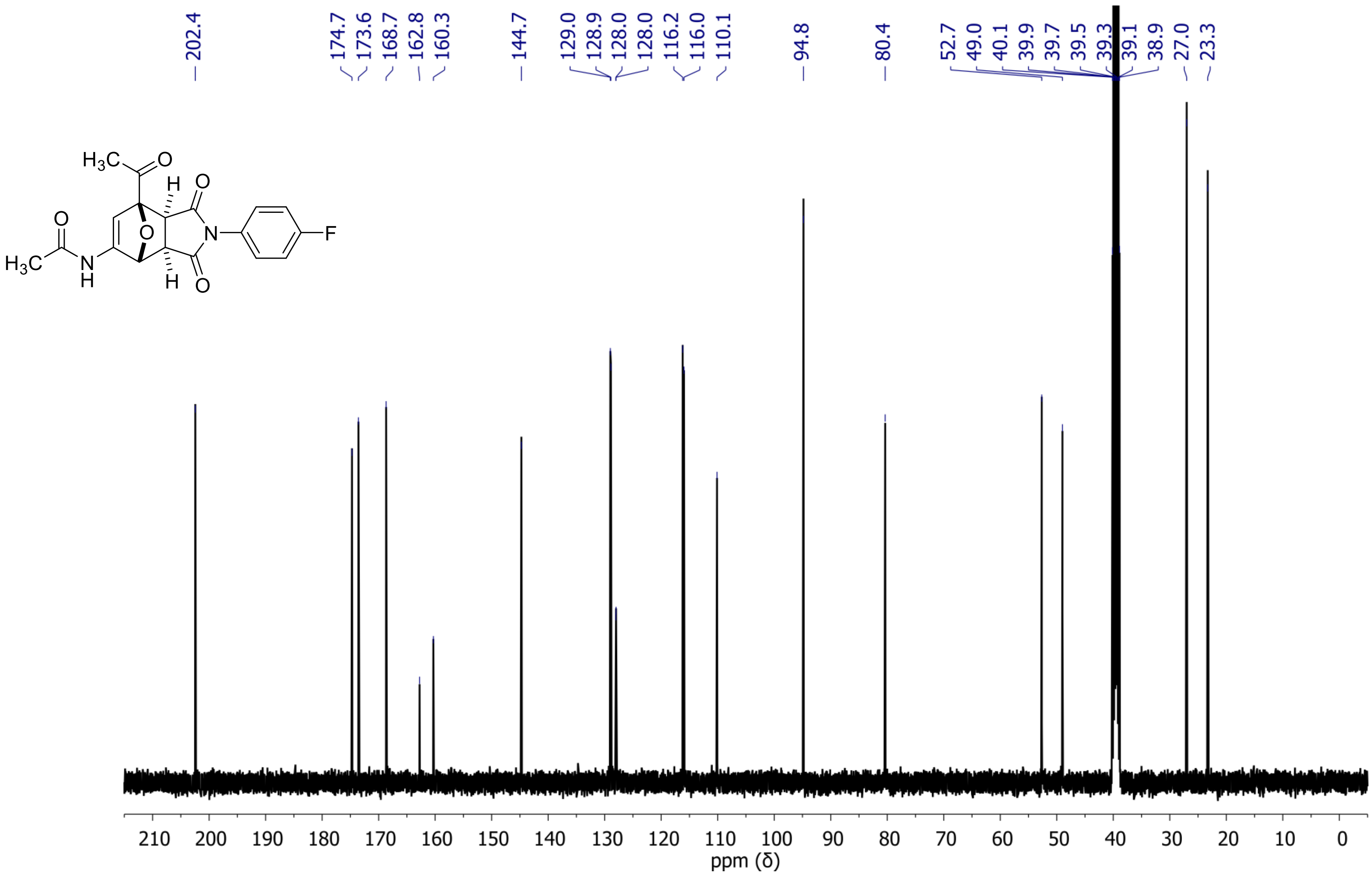

Figure S24. ^{1}H NMR Spectrum (400 MHz, DMSO-d_6) for Diels–Alder adduct **1i**

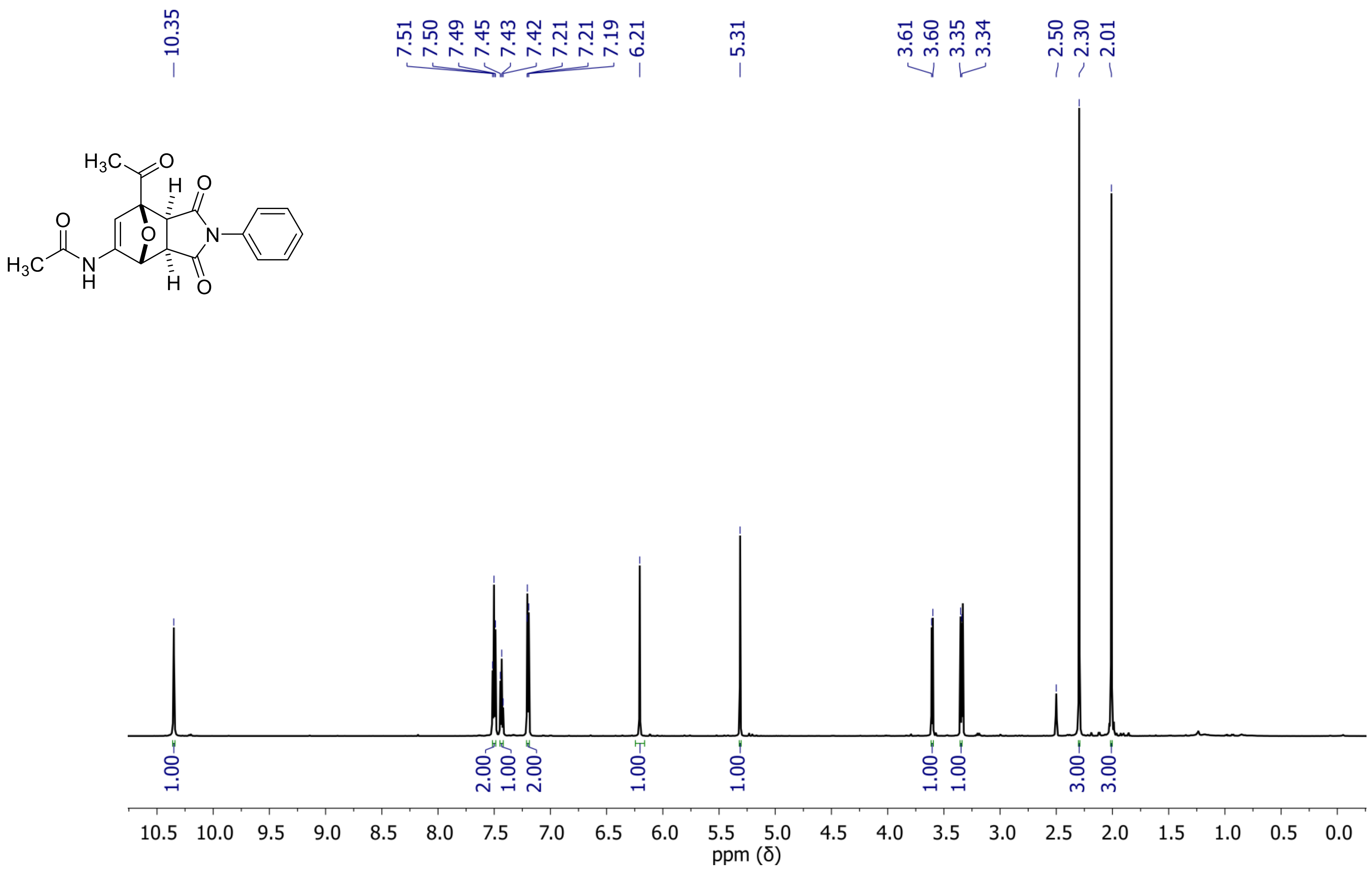

Figure S25. ^{13}C NMR Spectrum (100 MHz, DMSO-d_6) for Diels–Alder adduct **1i**

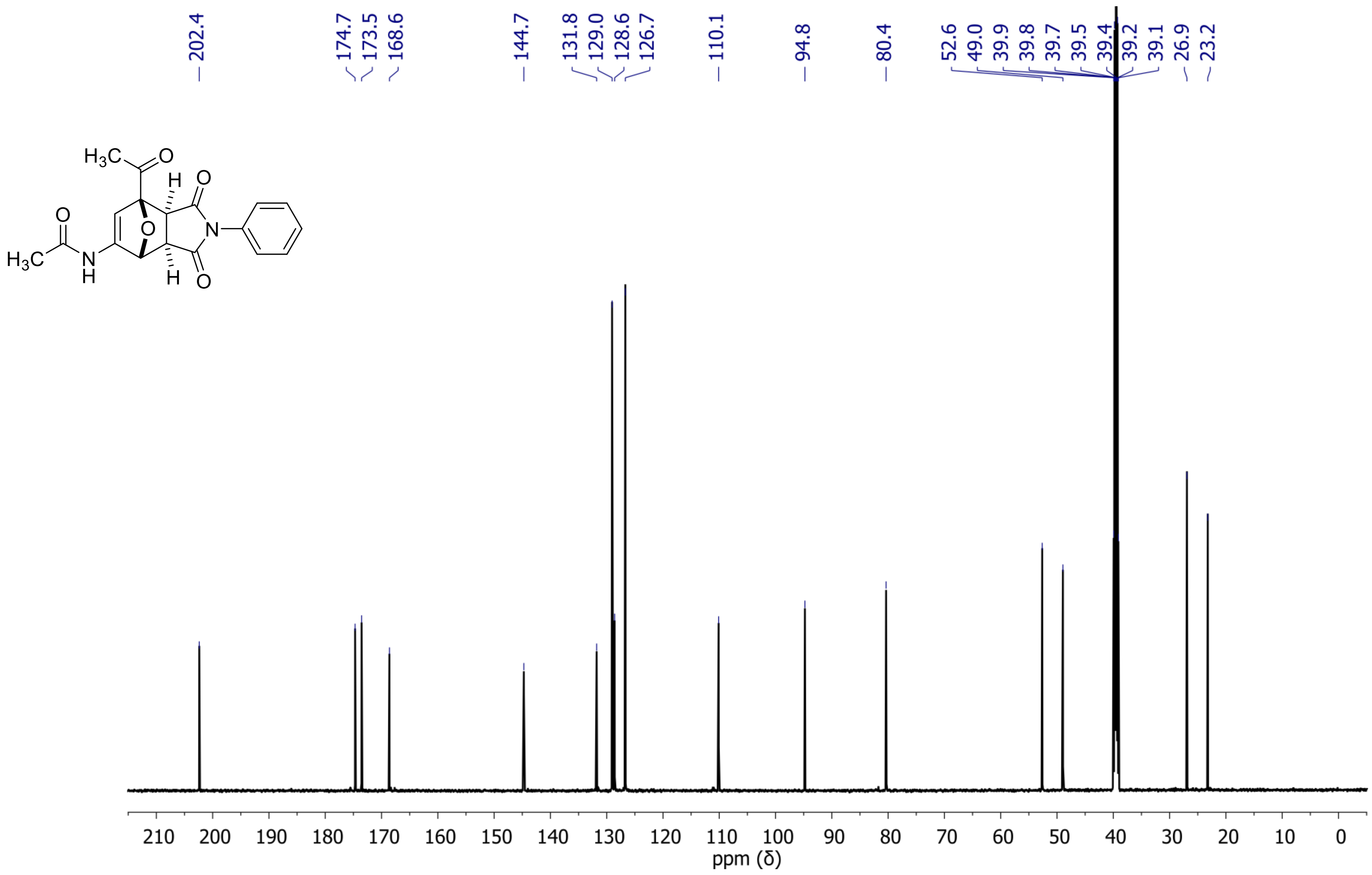

Figure S26. ^{1}H NMR Spectrum (400 MHz, DMSO-d_6) for Diels–Alder adduct **1j**

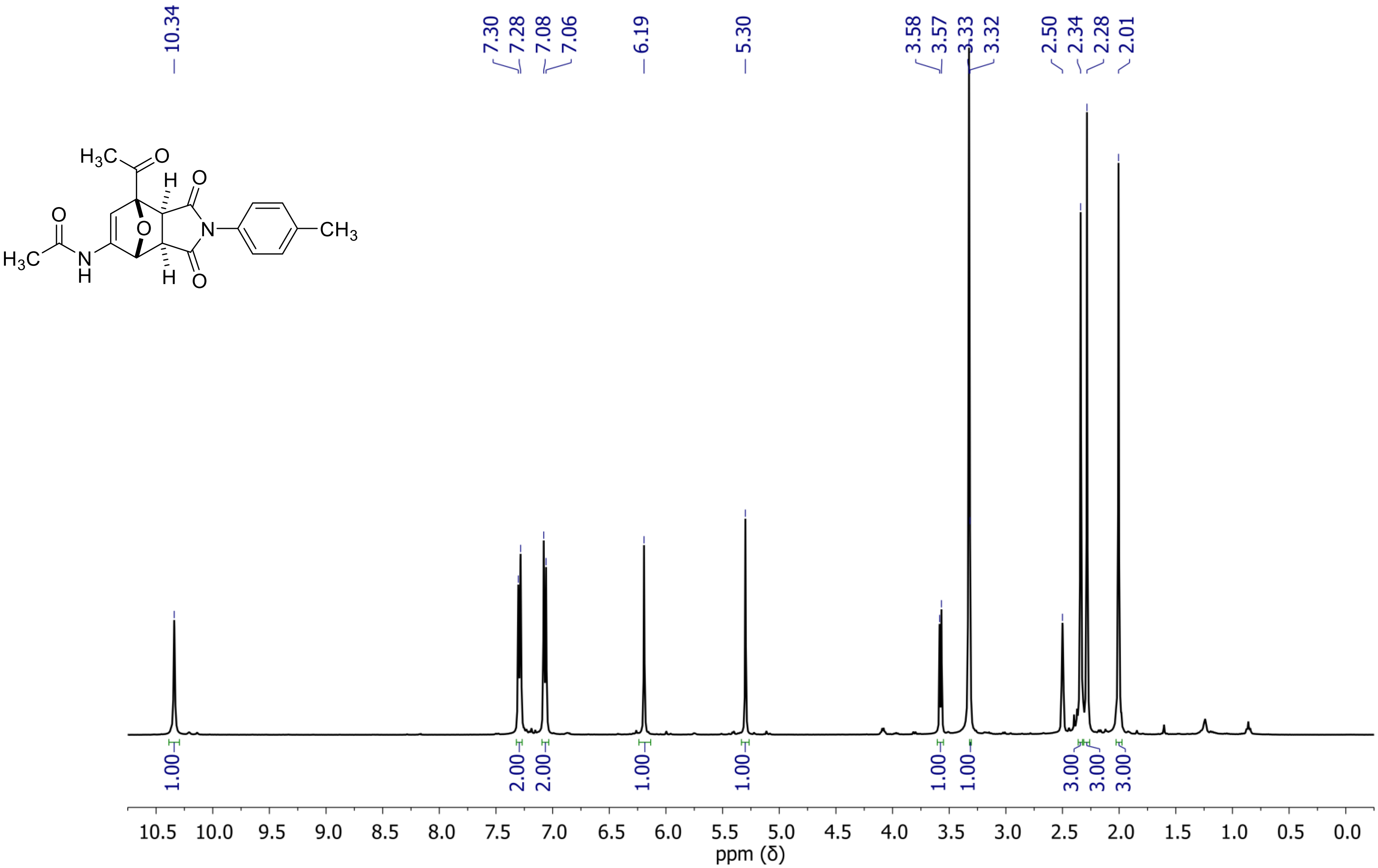

Figure S27. ^{13}C NMR Spectrum (100 MHz, DMSO-d_6) for Diels–Alder adduct **1j**

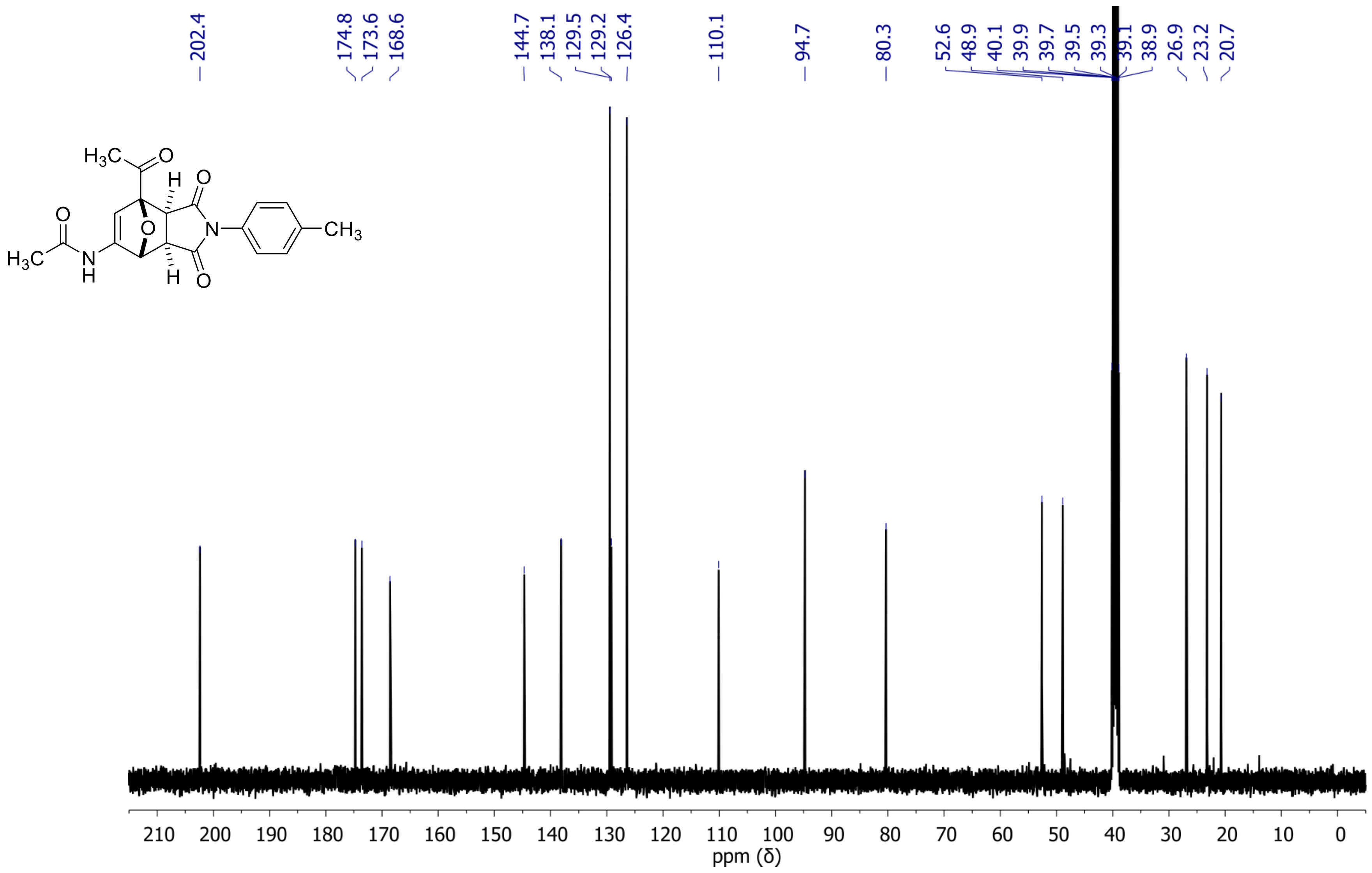

Figure S28. ^{1}H NMR Spectrum (400 MHz, DMSO-d_6) for Diels–Alder adduct **1k**

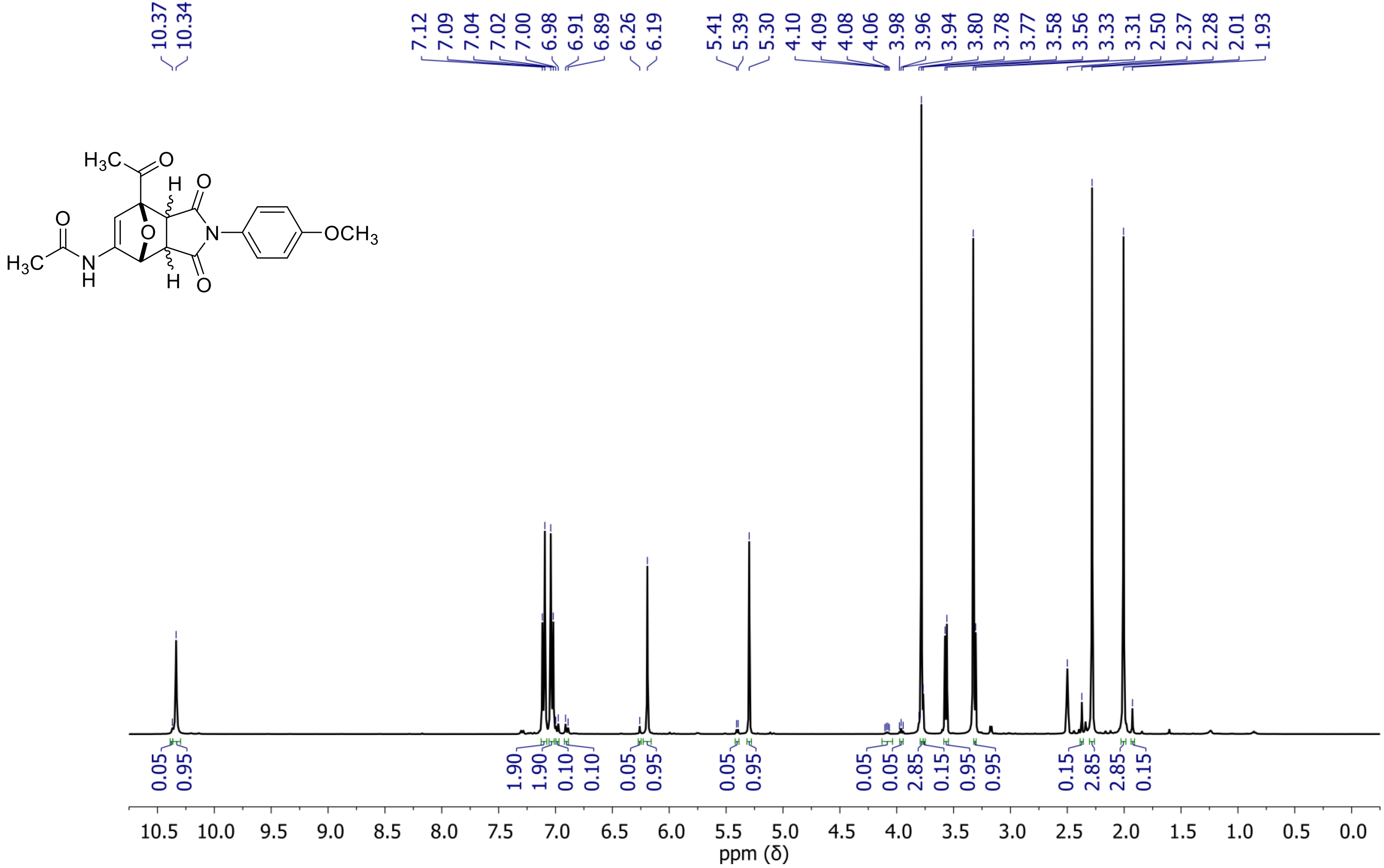

Figure S29. ^{13}C NMR Spectrum (100 MHz, DMSO-d_6) for Diels–Alder adduct **1k**

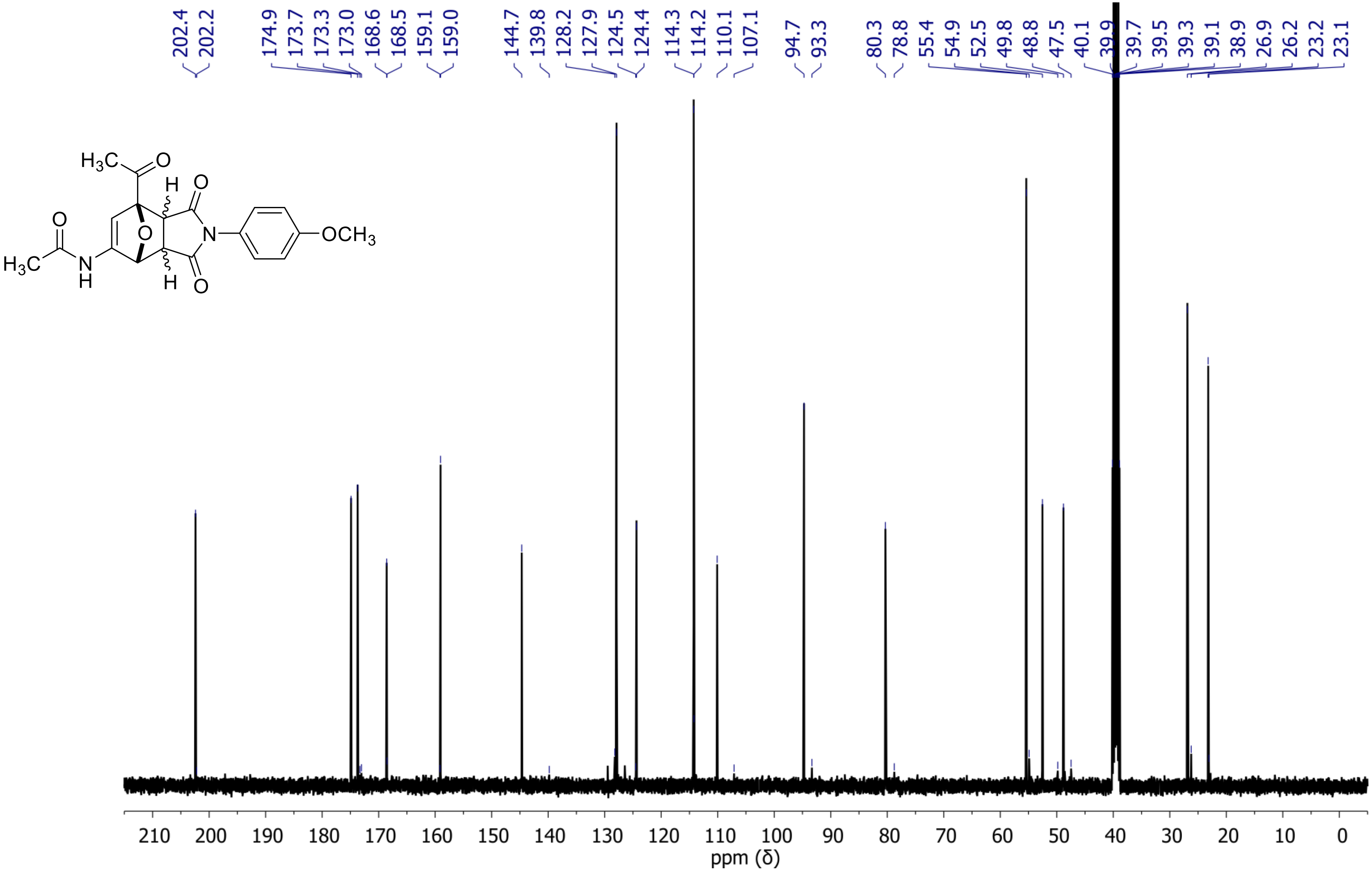

Figure S30. ^{1}H NMR Spectrum (400 MHz, DMSO-d_6) for Diels–Alder adduct **1l**

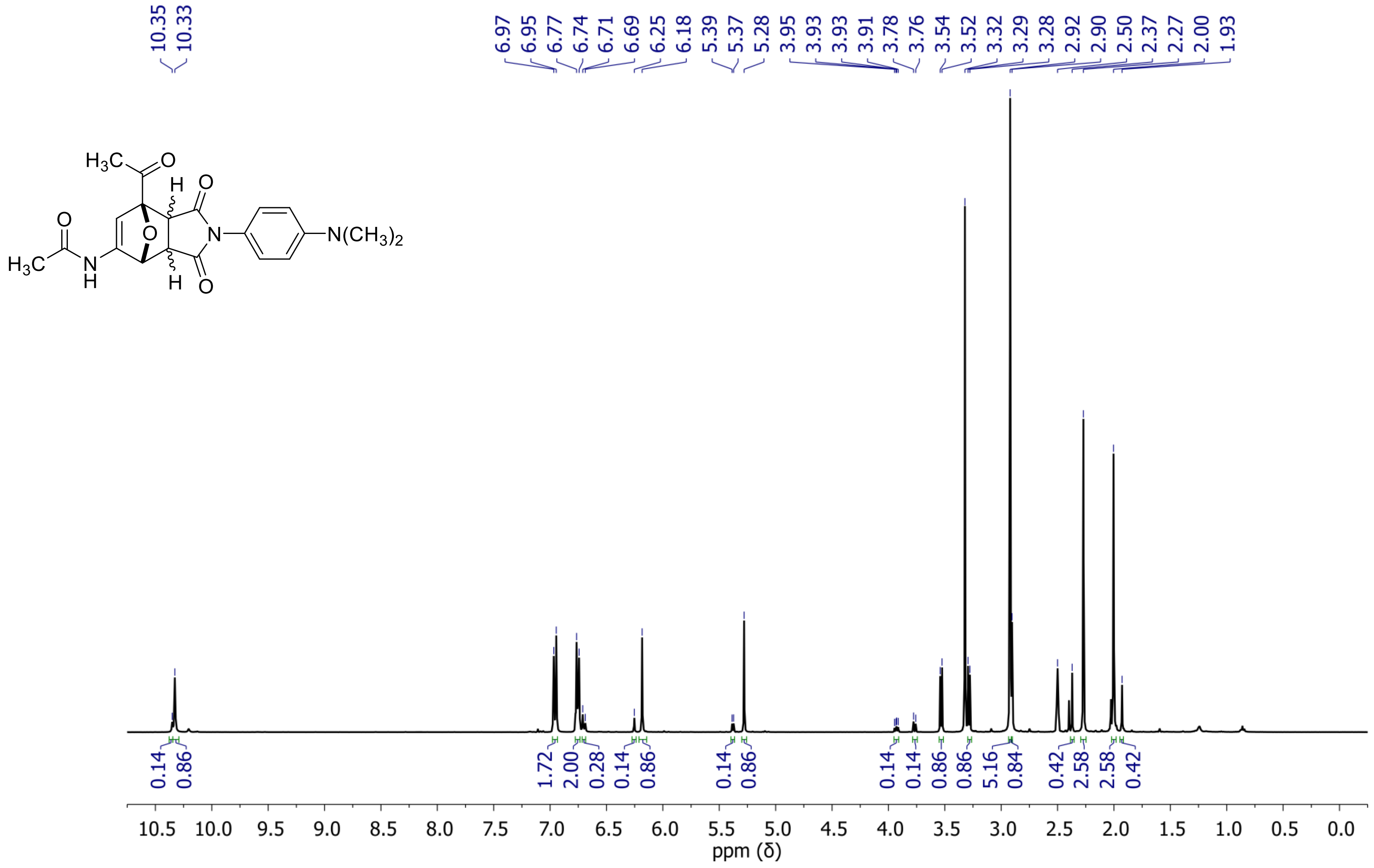

Figure S31. ^{13}C NMR Spectrum (100 MHz, DMSO-d_6) for Diels–Alder adduct **1l**

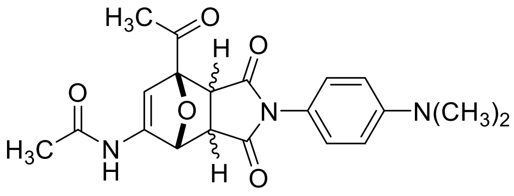

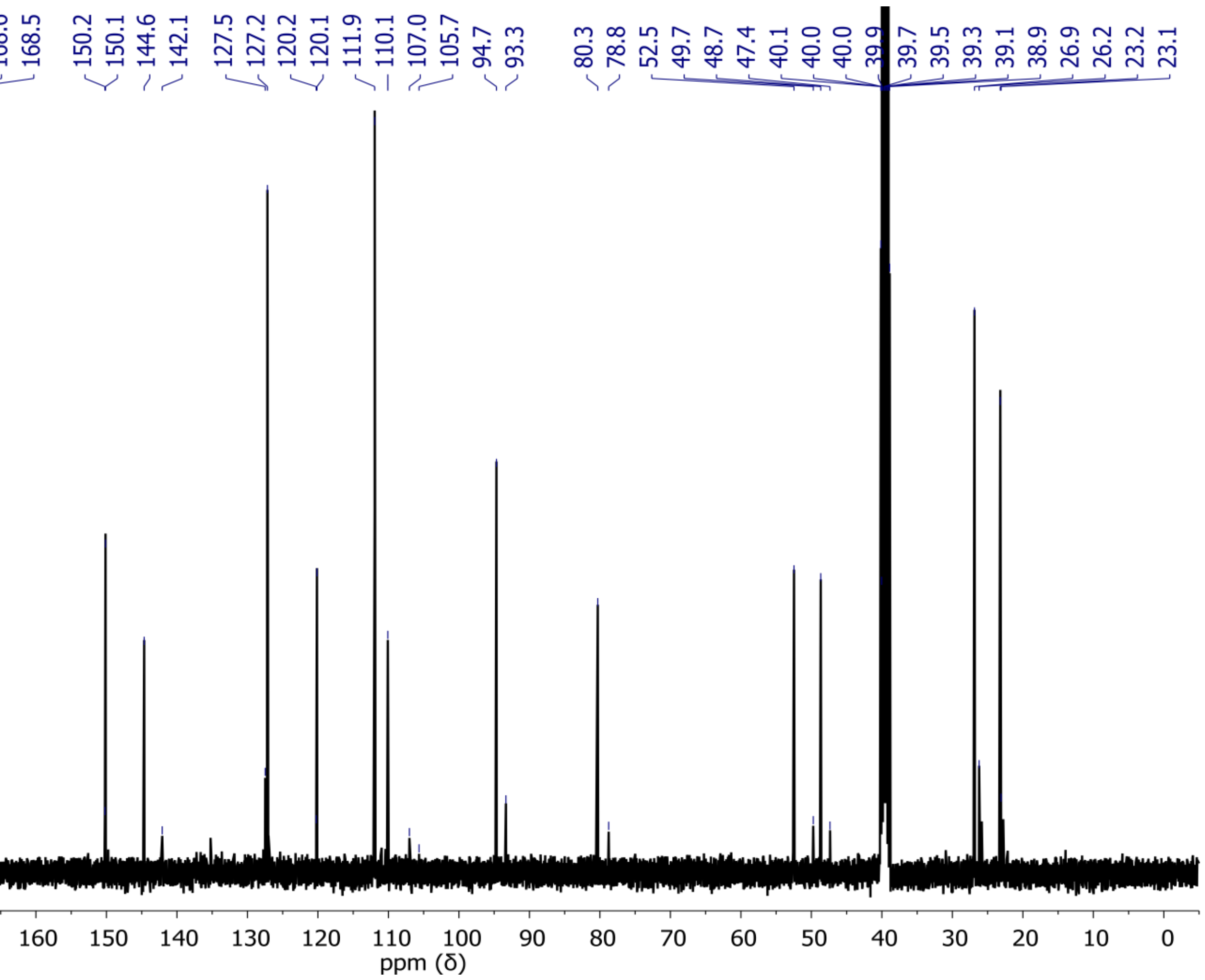

Figure S32. ^{1}H NMR Spectrum (400 MHz, DMSO-d_6) for 3-acetamido-5-furfural (**3A5F**)

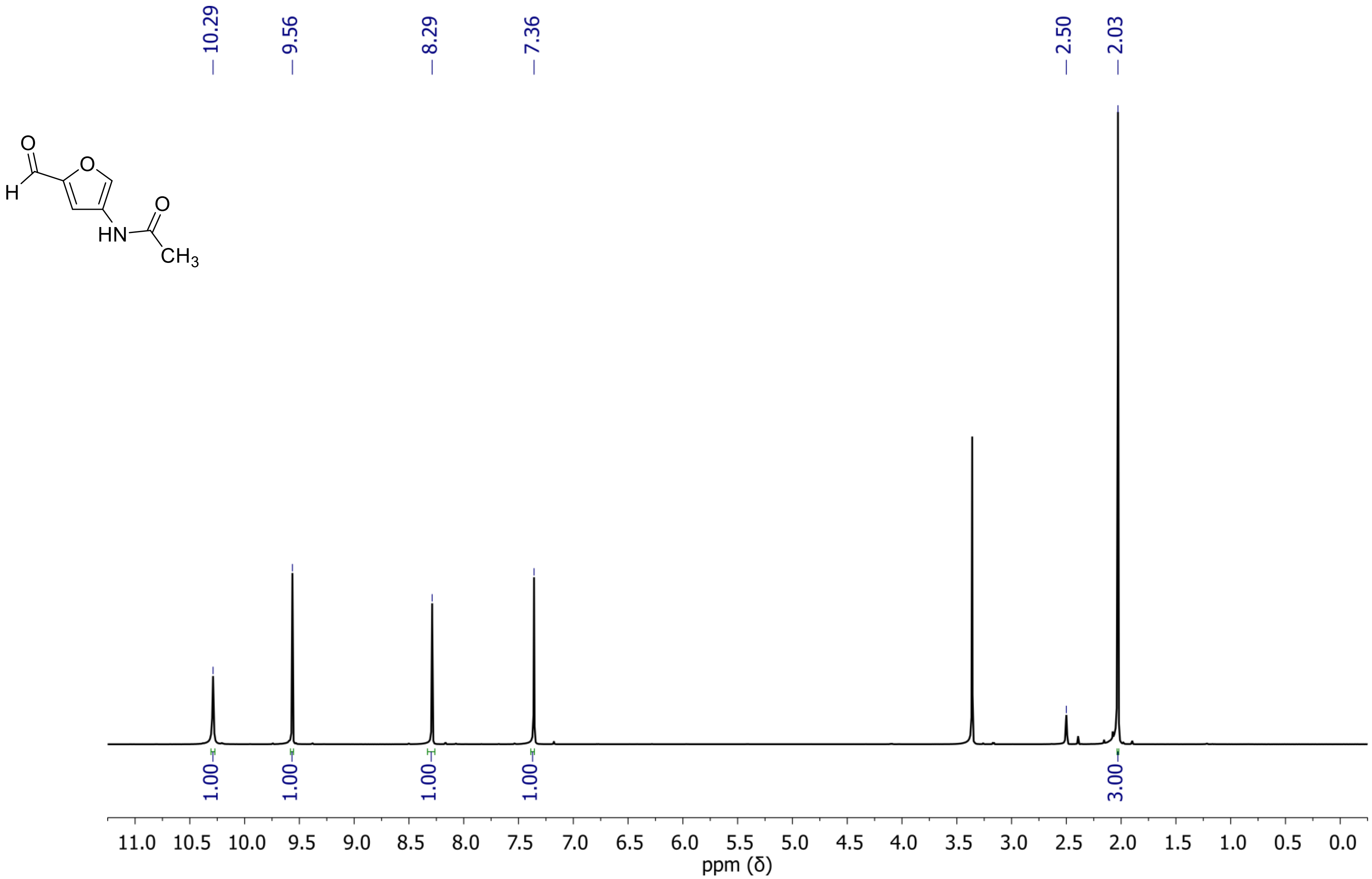

Figure S33. ^{13}C NMR Spectrum (100 MHz, DMSO-d_6) for 3-acetamido-5-furfural (**3A5F**)

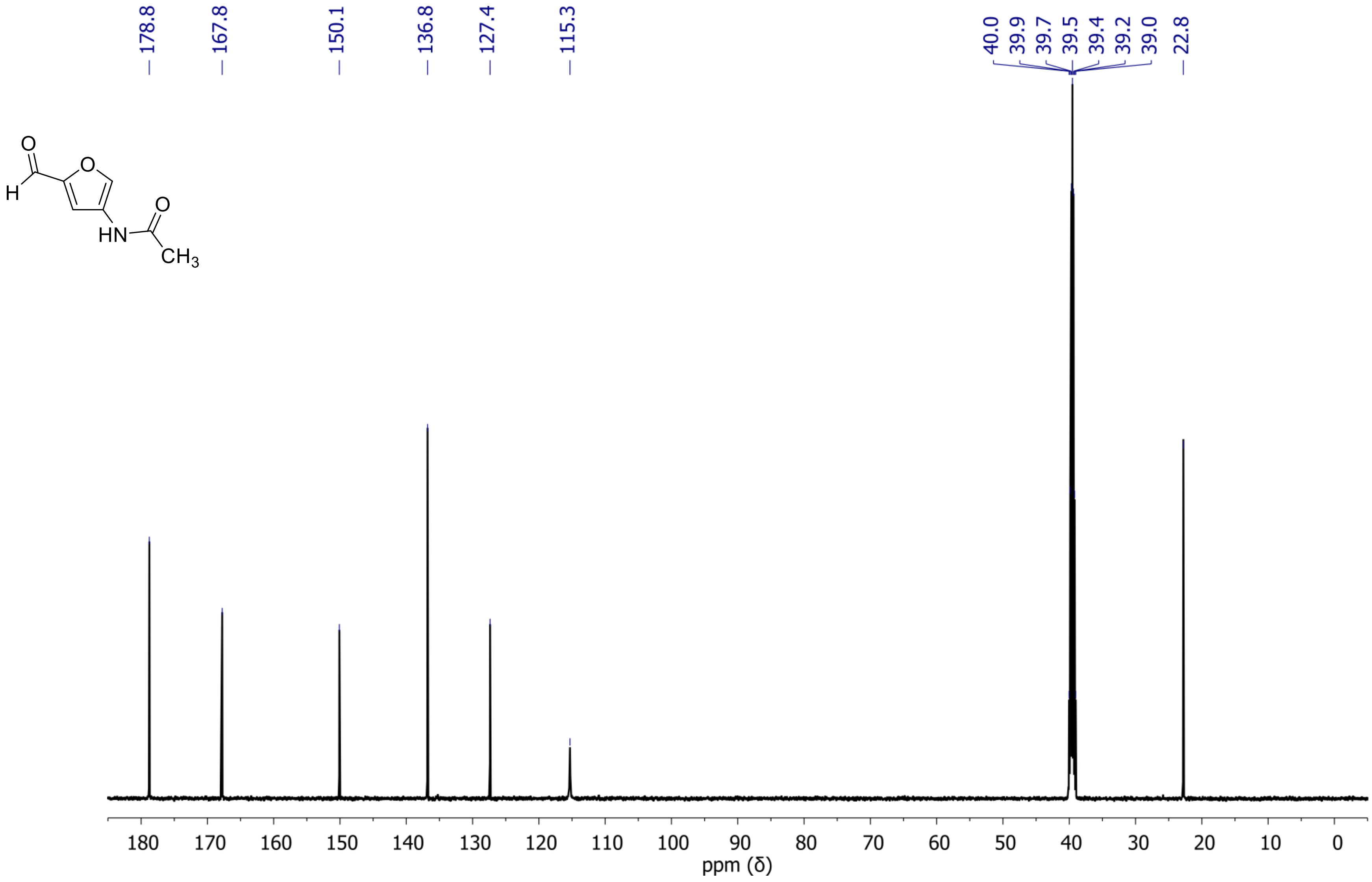

Figure S34. ^{1}H NMR Spectrum (400 MHz, DMSO-d_6) for hydrazone **2**

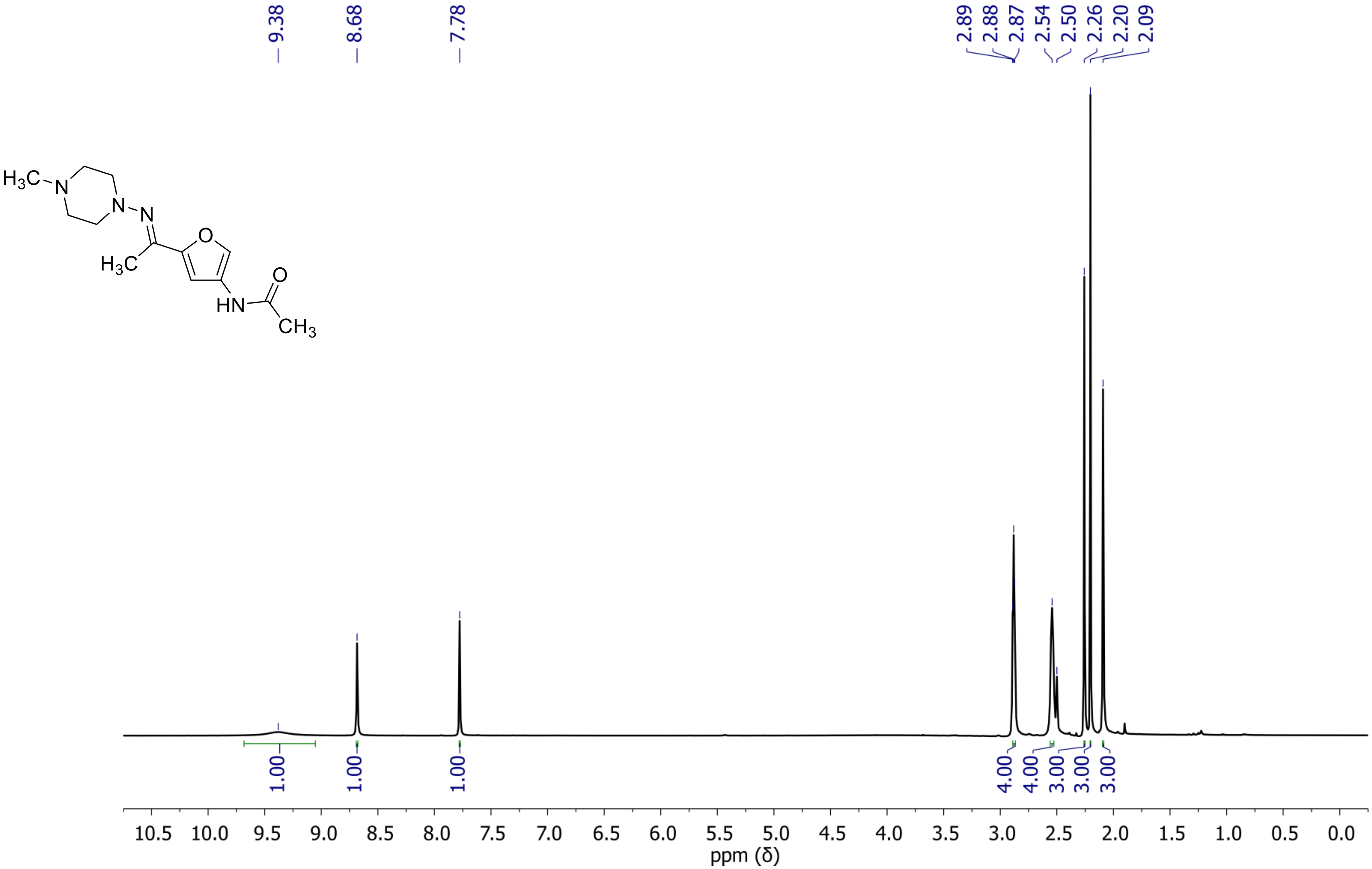

Figure S35. ^{13}C NMR Spectrum (100 MHz, DMSO-d_6) for hydrazone **2**

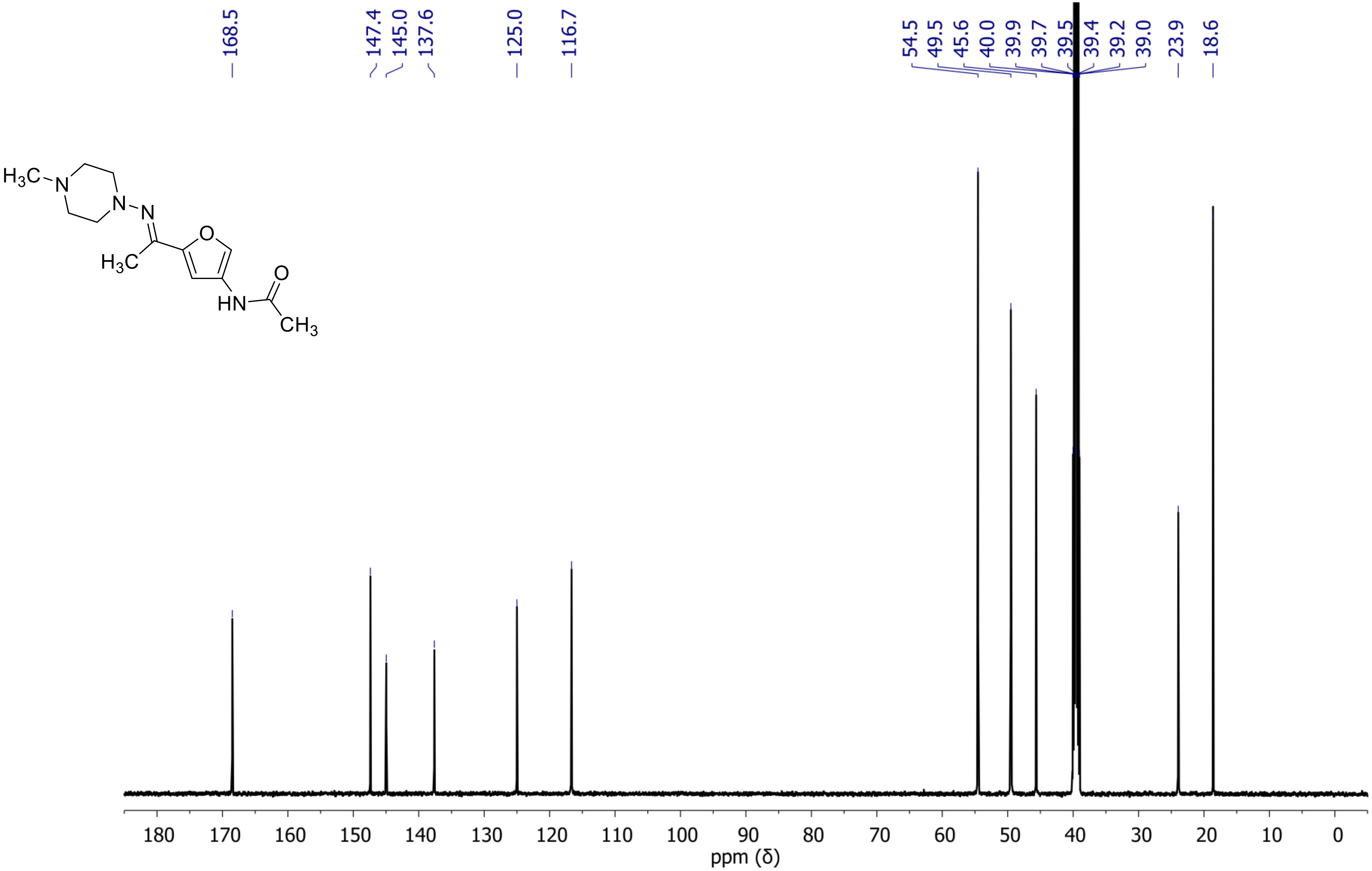

Figure S36. DEPT-135 NMR Spectrum (400 MHz, DMSO-*d*$_6$) for hydrazone **2**

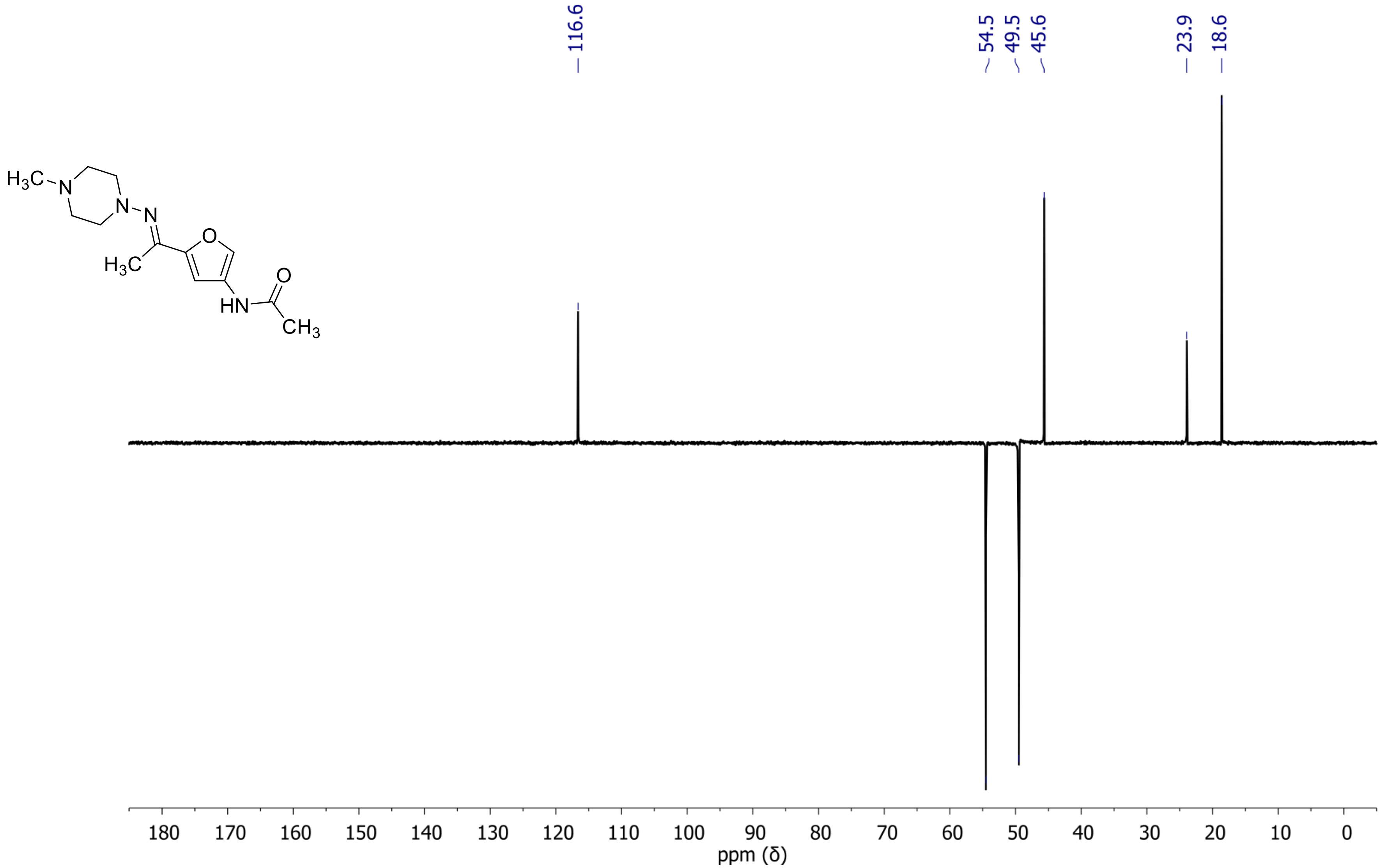

Figure S37. HSQC NMR Spectrum (400 MHz, DMSO-d_6) for hydrazone **2**

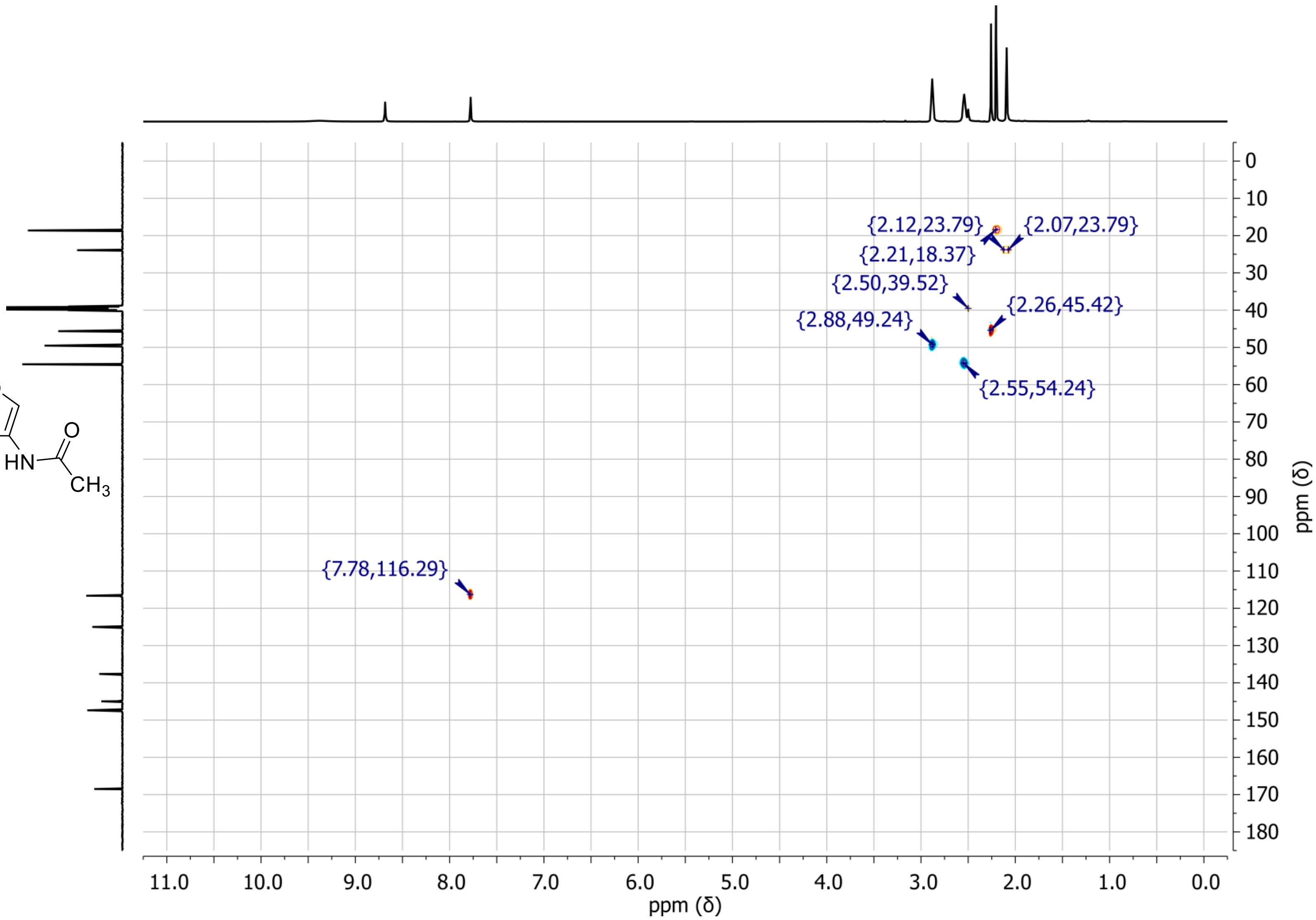

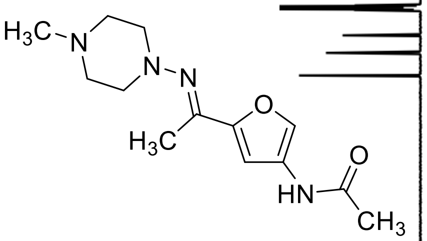

Figure S38. HMBC NMR Spectrum (400 MHz, DMSO-d_6) for hydrazone **2**

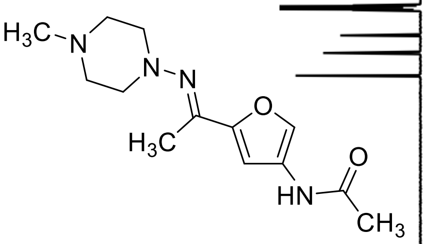

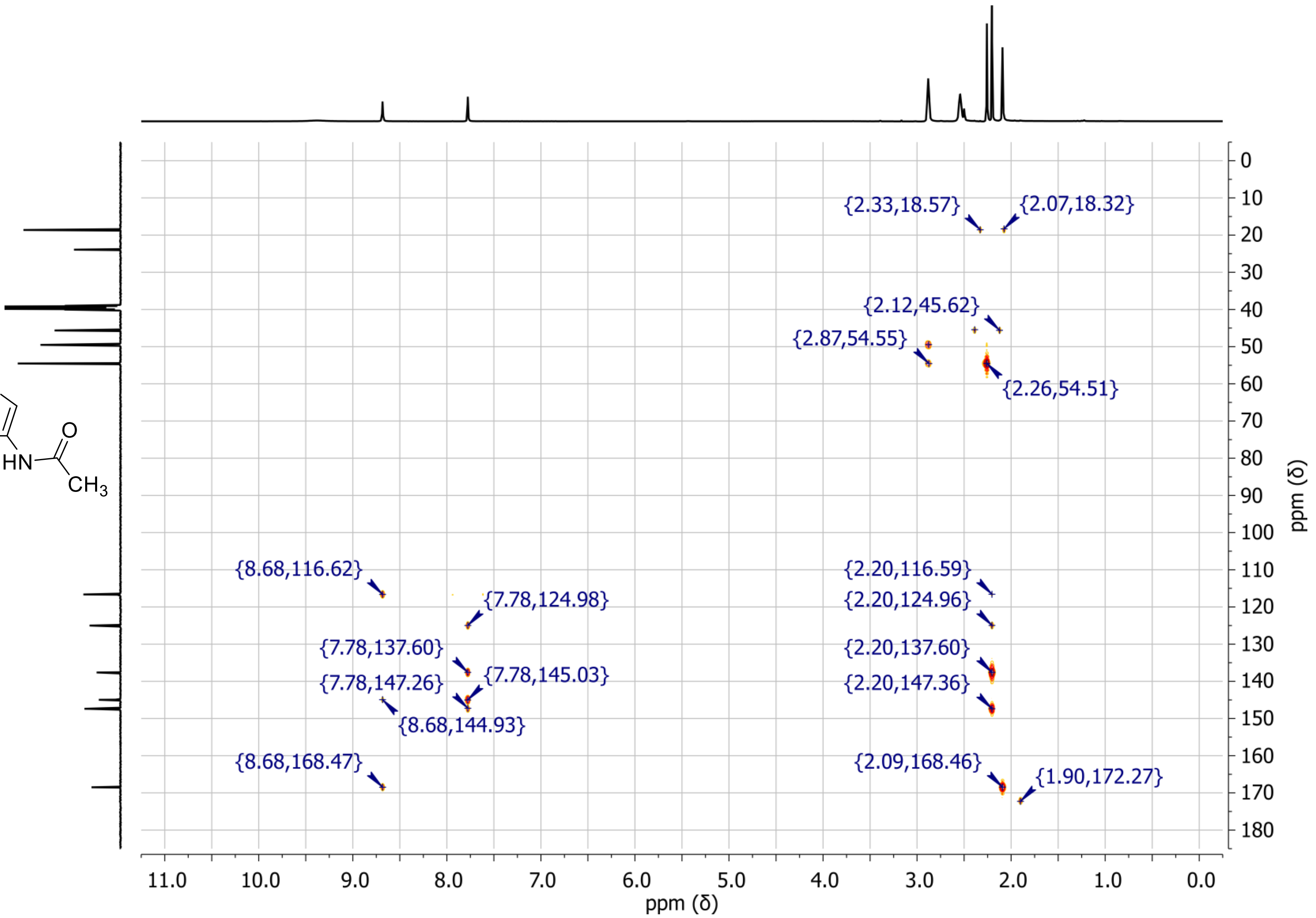

Figure S39. NOESY NMR Spectrum (400 MHz, DMSO-d_6) for hydrazone **2**

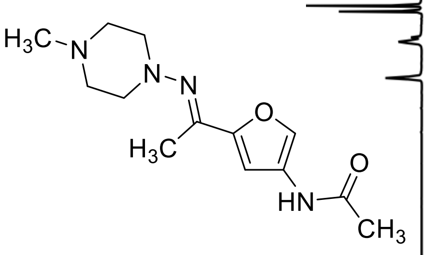

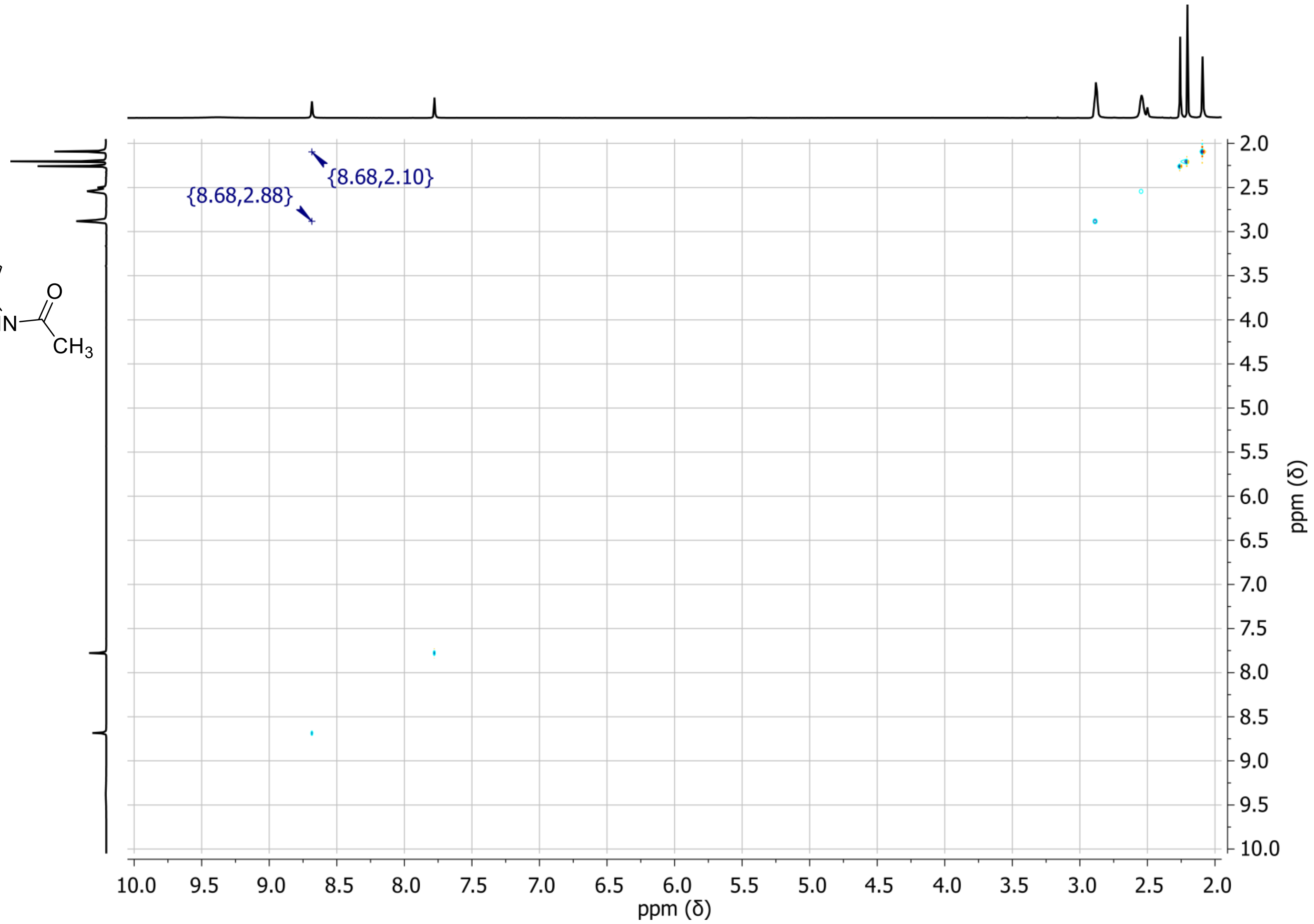

Figure S40. ^{1}H NMR Spectrum (400 MHz, DMSO-d_6) for hydrazone **3**

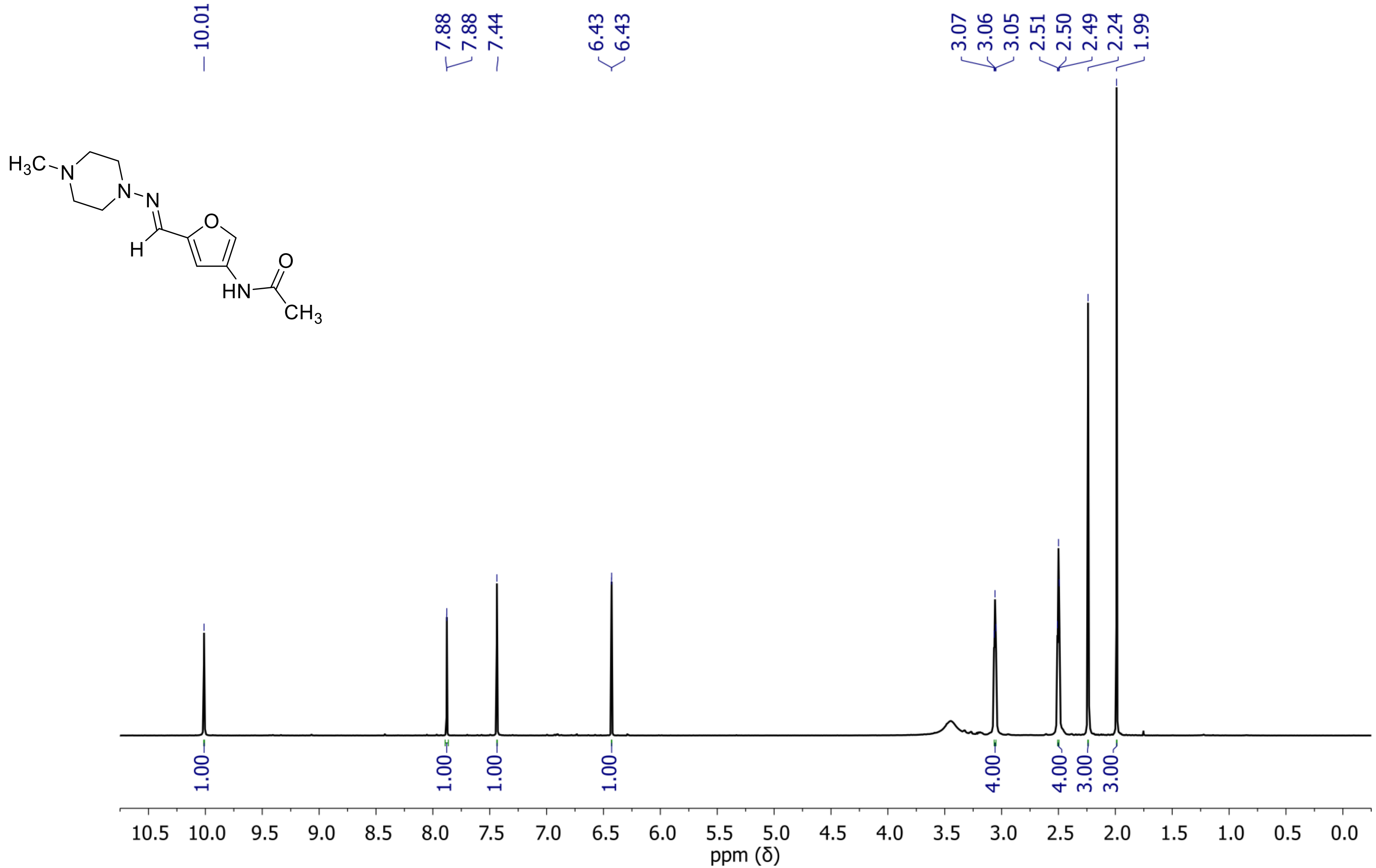

Figure S41. ^{13}C NMR Spectrum (100 MHz, DMSO-d_6) for hydrazone **3**

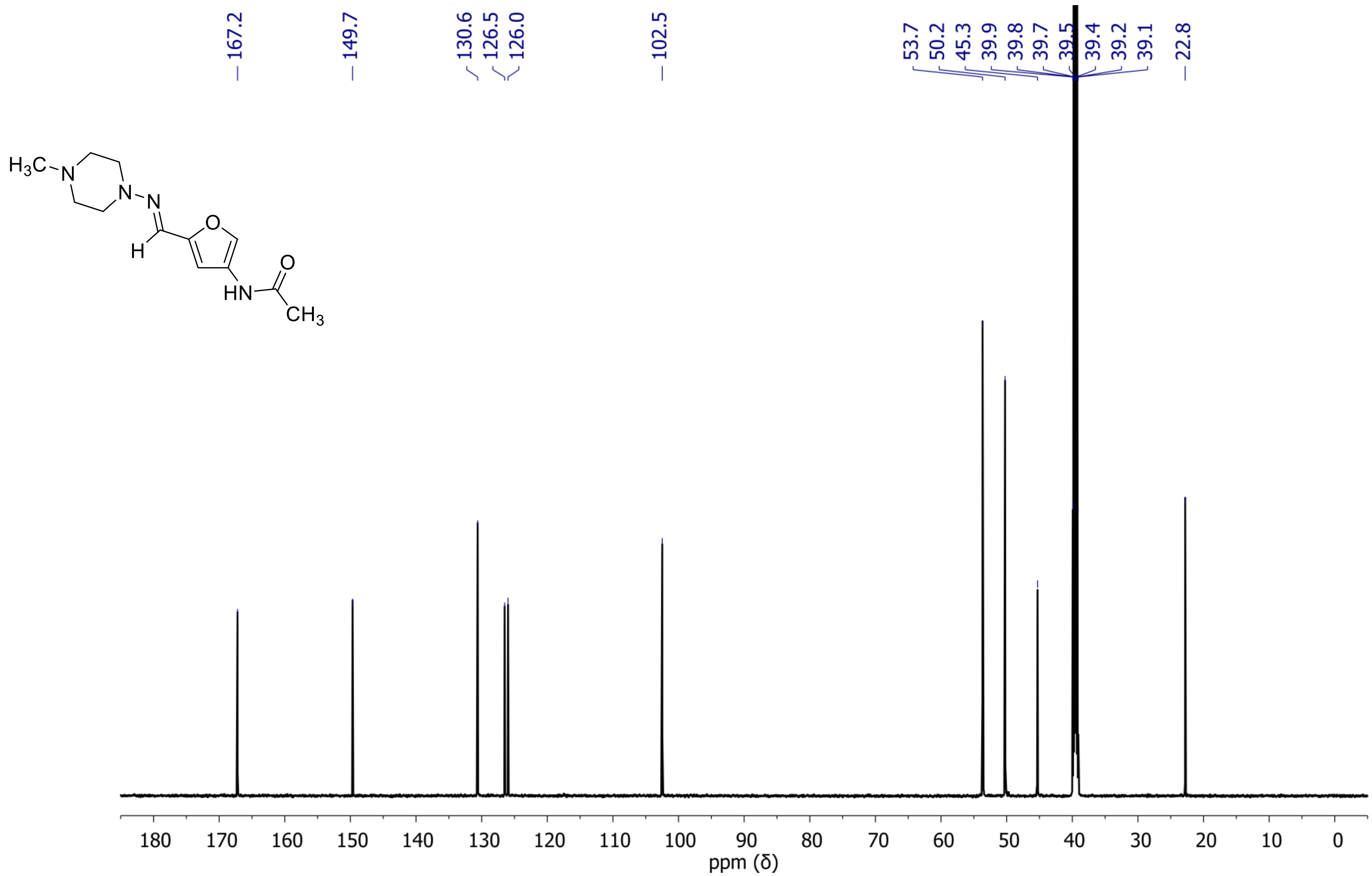

Figure S42. ^{1}H NMR Spectrum (400 MHz, DMSO-d_6) for phthalimide **3a**

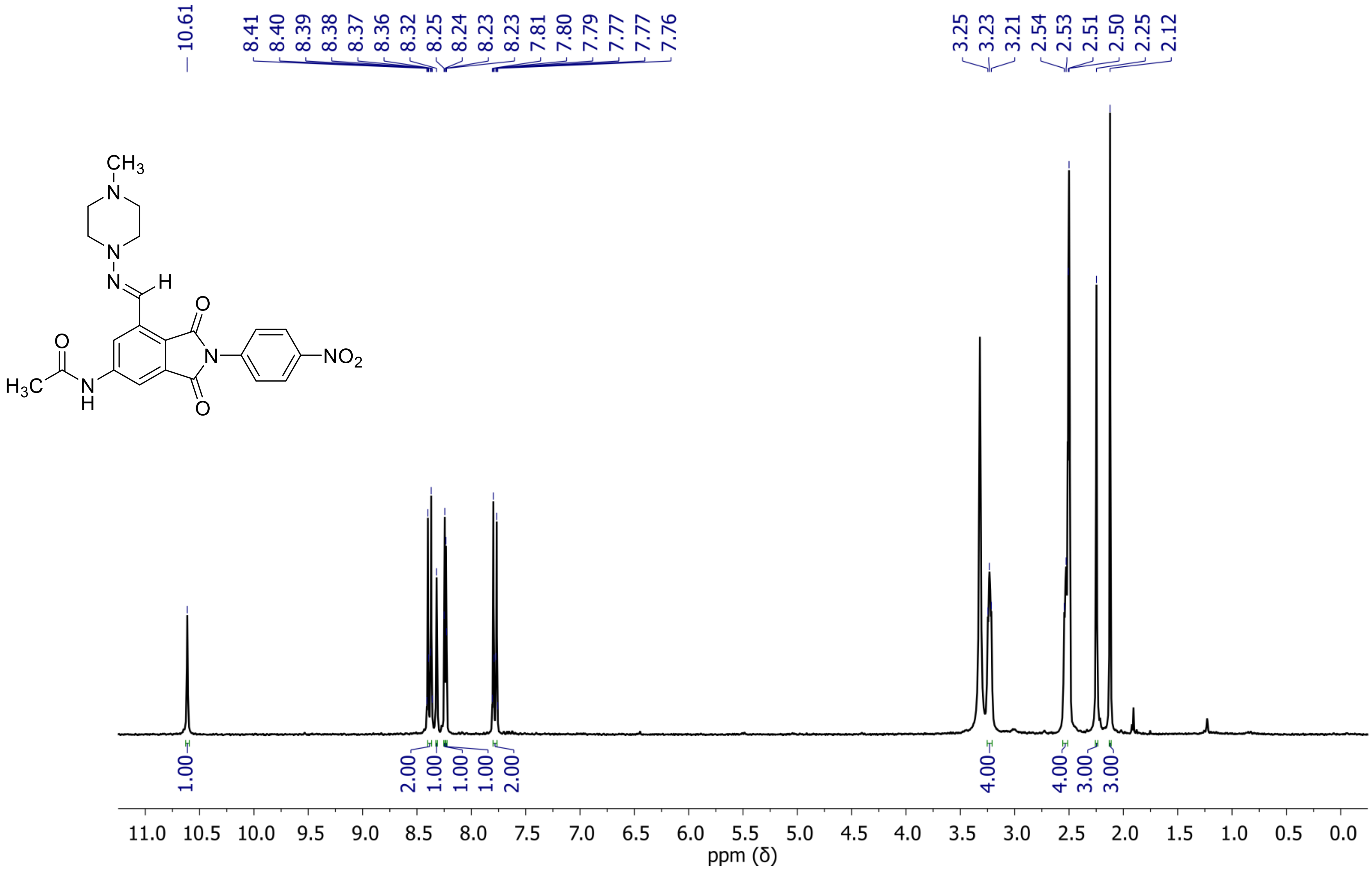

Figure S43. ^{13}C NMR Spectrum (100 MHz, DMSO-d_6) for phthalimide **3a**

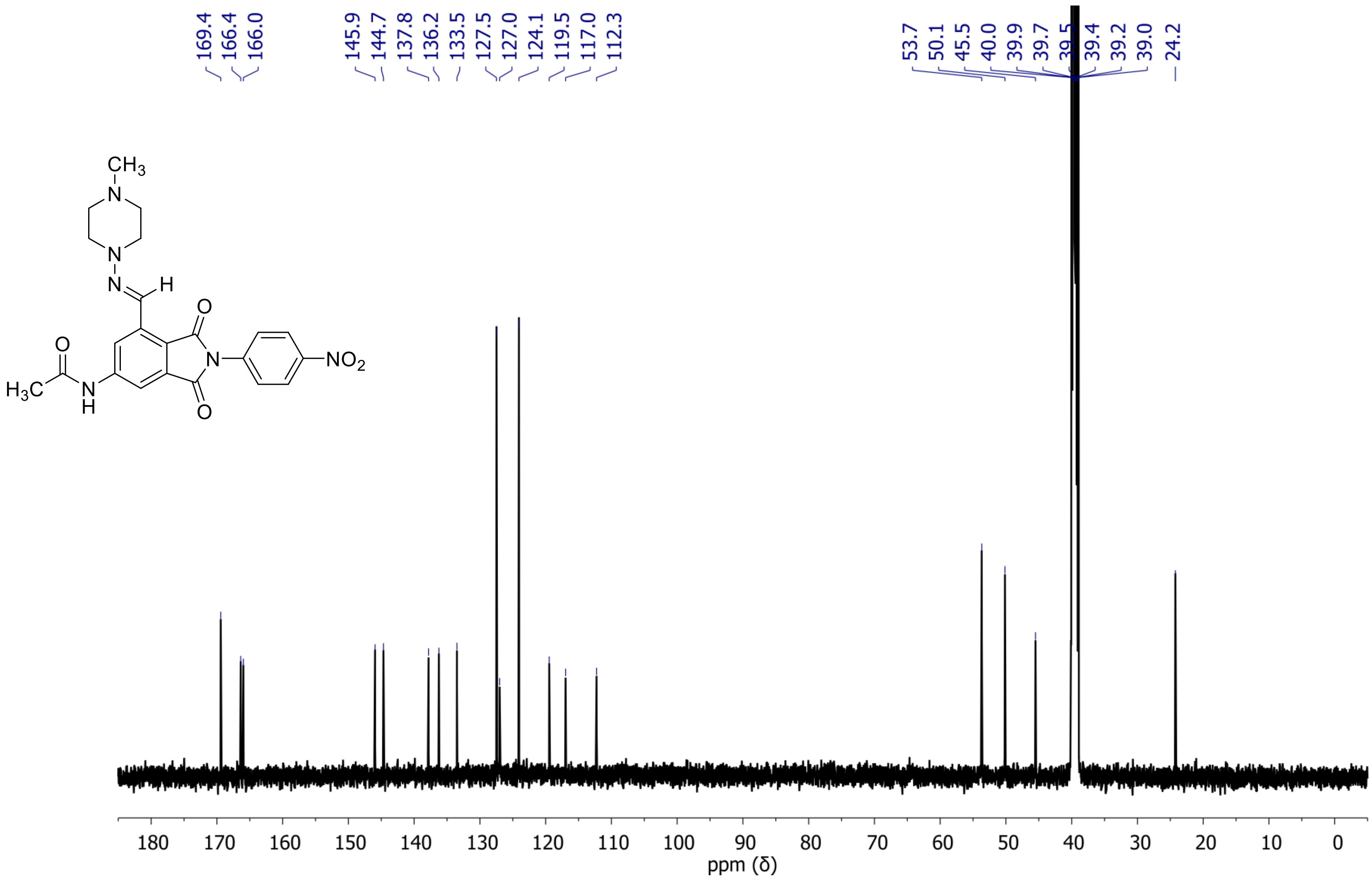

Figure S44. ^{1}H NMR Spectrum (400 MHz, DMSO-d_6) for phthalimide **3b**

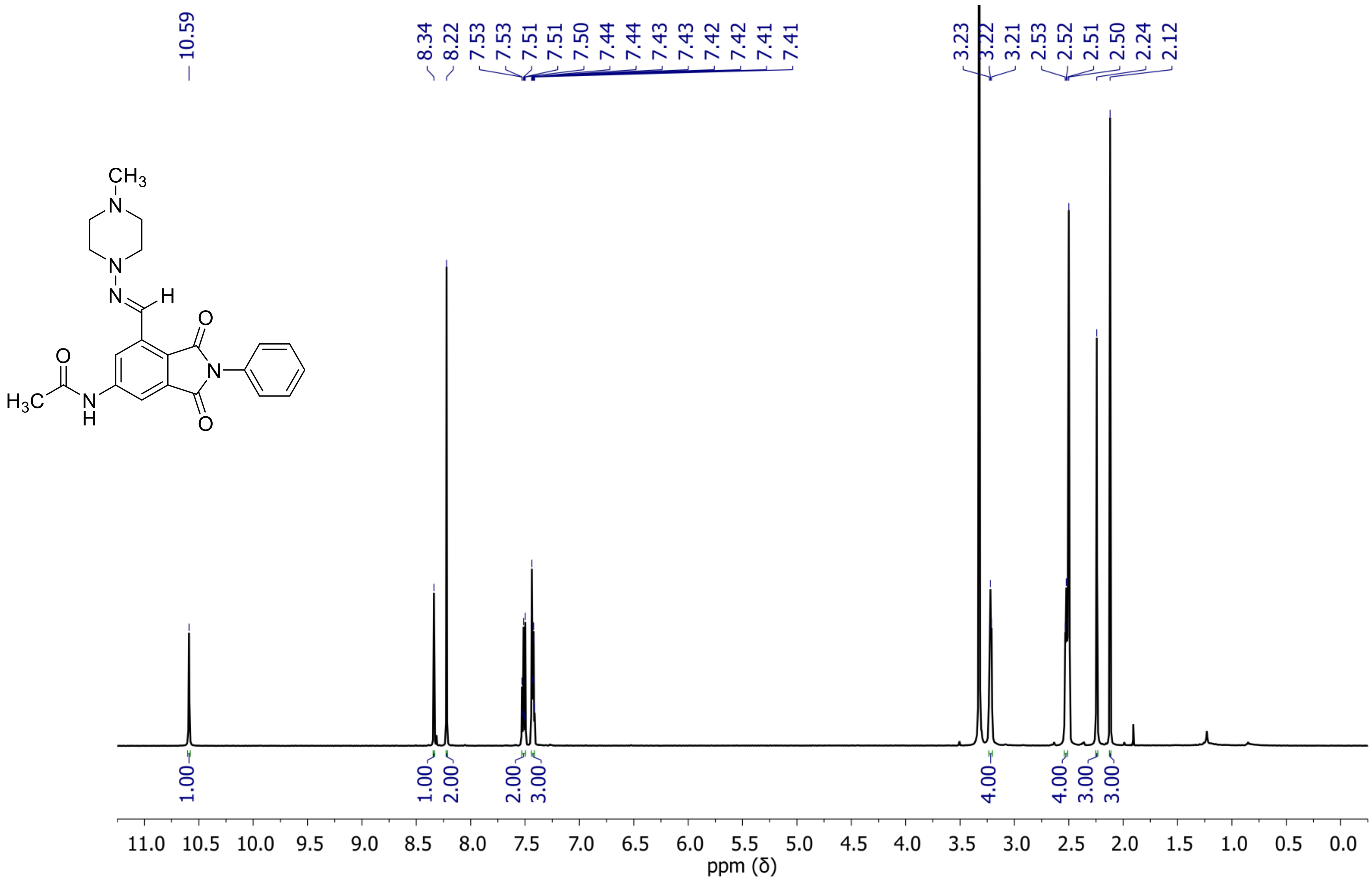

Figure S45. ^{13}C NMR Spectrum (100 MHz, DMSO-d_6) for phthalimide **3b**

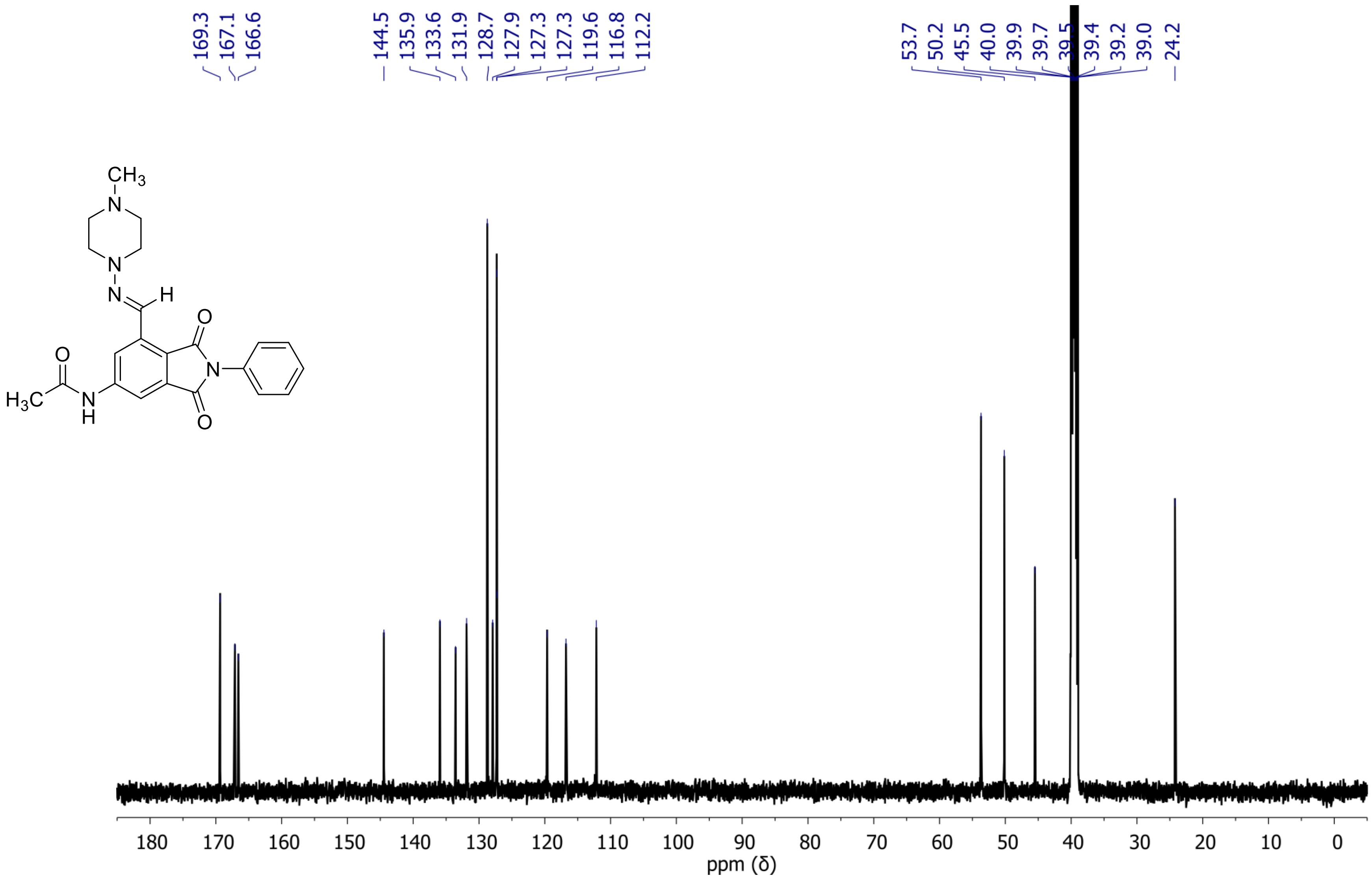

Figure S46. ^{1}H NMR Spectrum (400 MHz, DMSO-d_6) for phthalimide **3c**

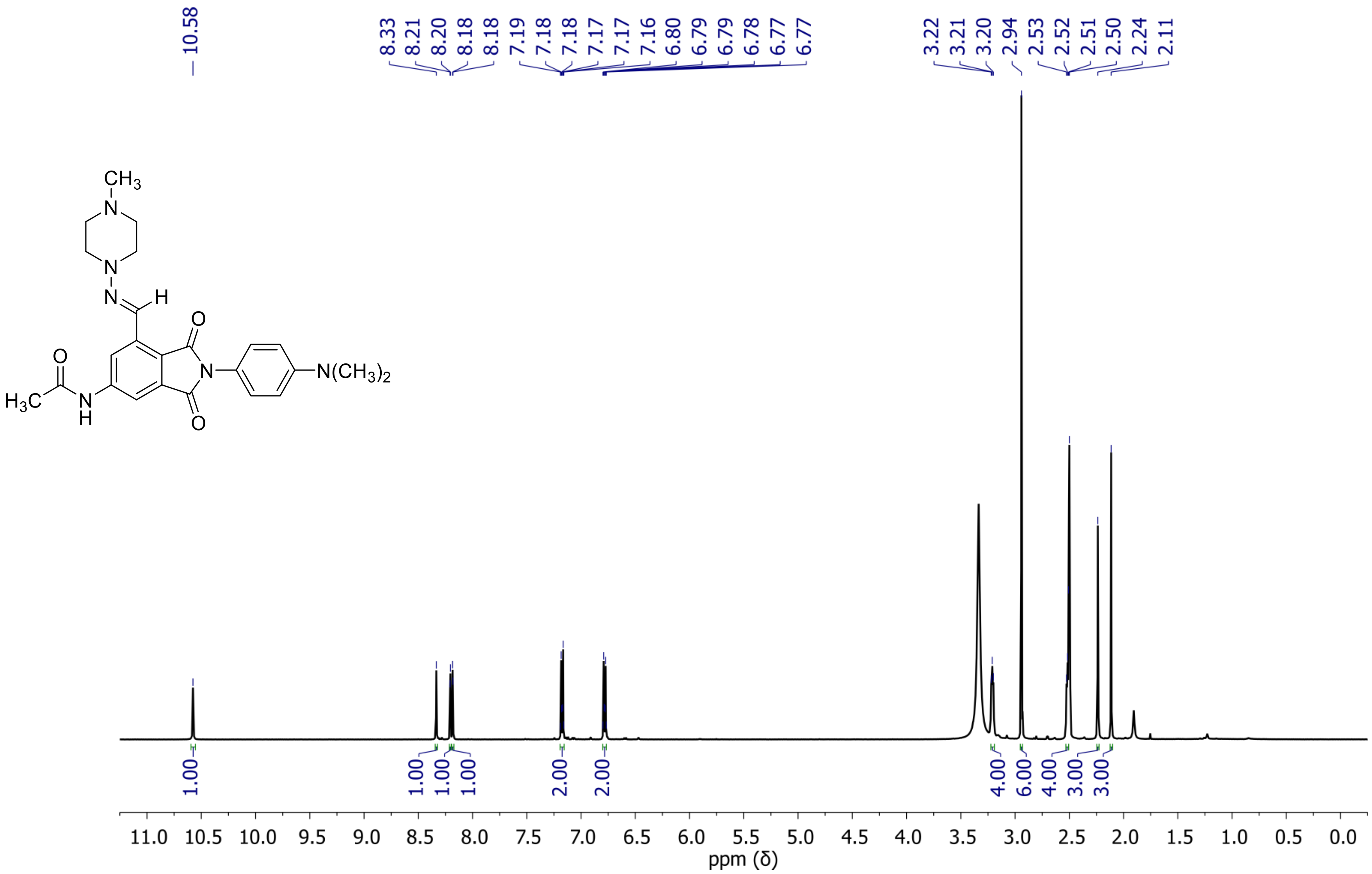

Figure S47. ^{13}C NMR Spectrum (100 MHz, DMSO-d_6) for phthalimide **3c**

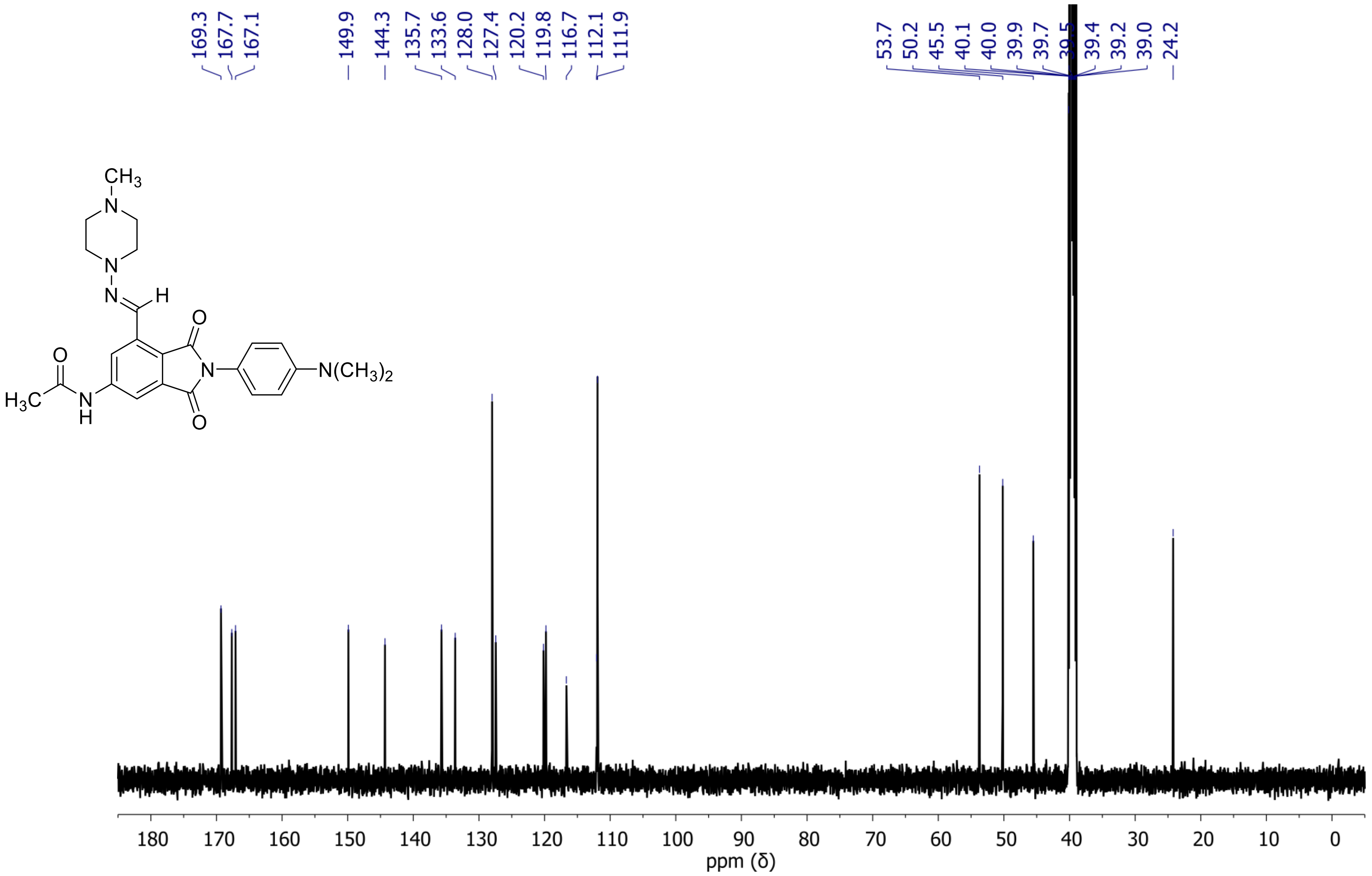

Figure S48. ^{1}H NMR Spectrum (400 MHz, DMSO-d_6) for phthalimide **3d**

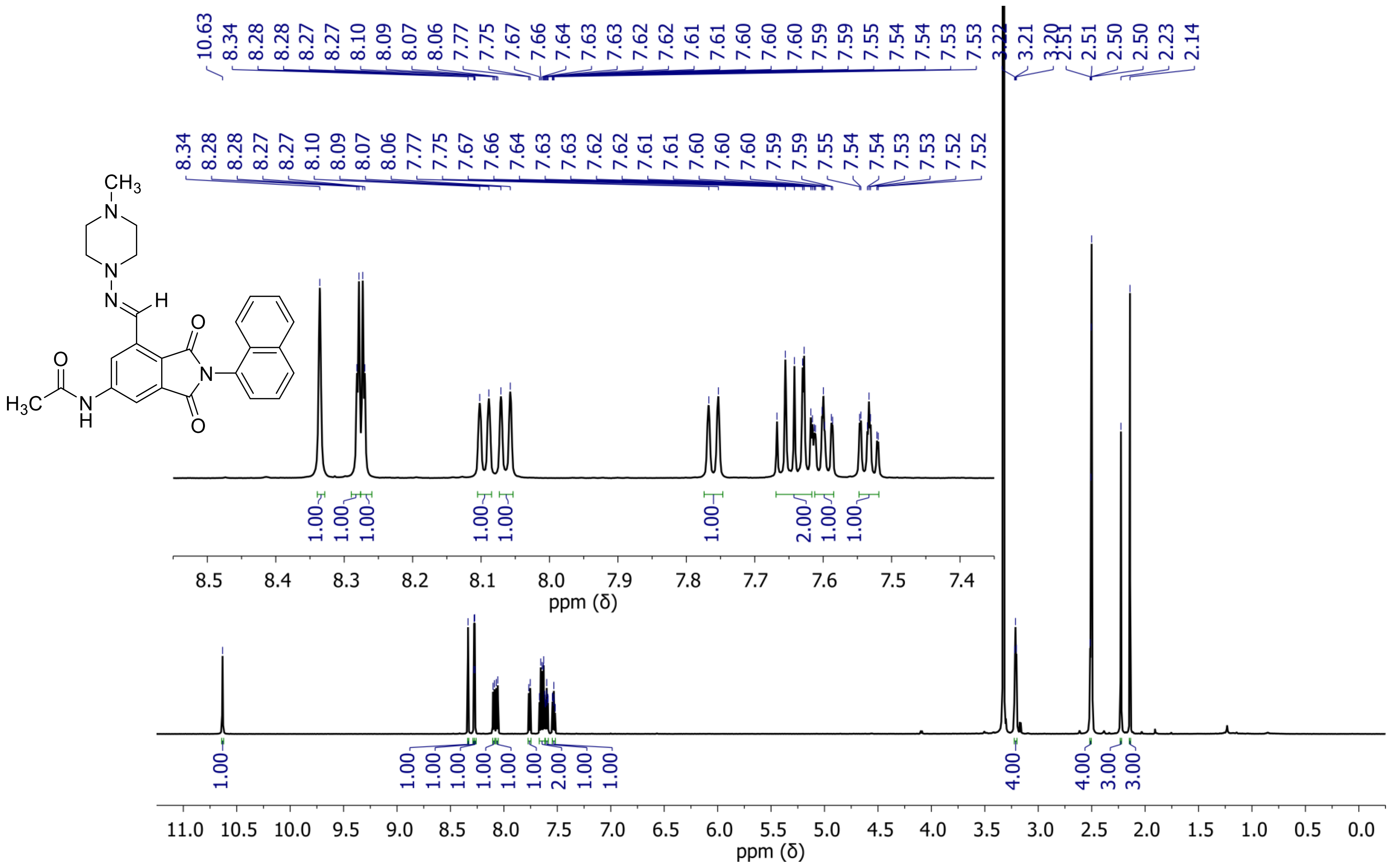

Figure S49. ^{13}C NMR Spectrum (100 MHz, DMSO-d_6) for phthalimide **3d**

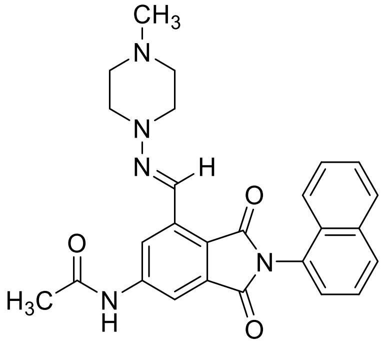

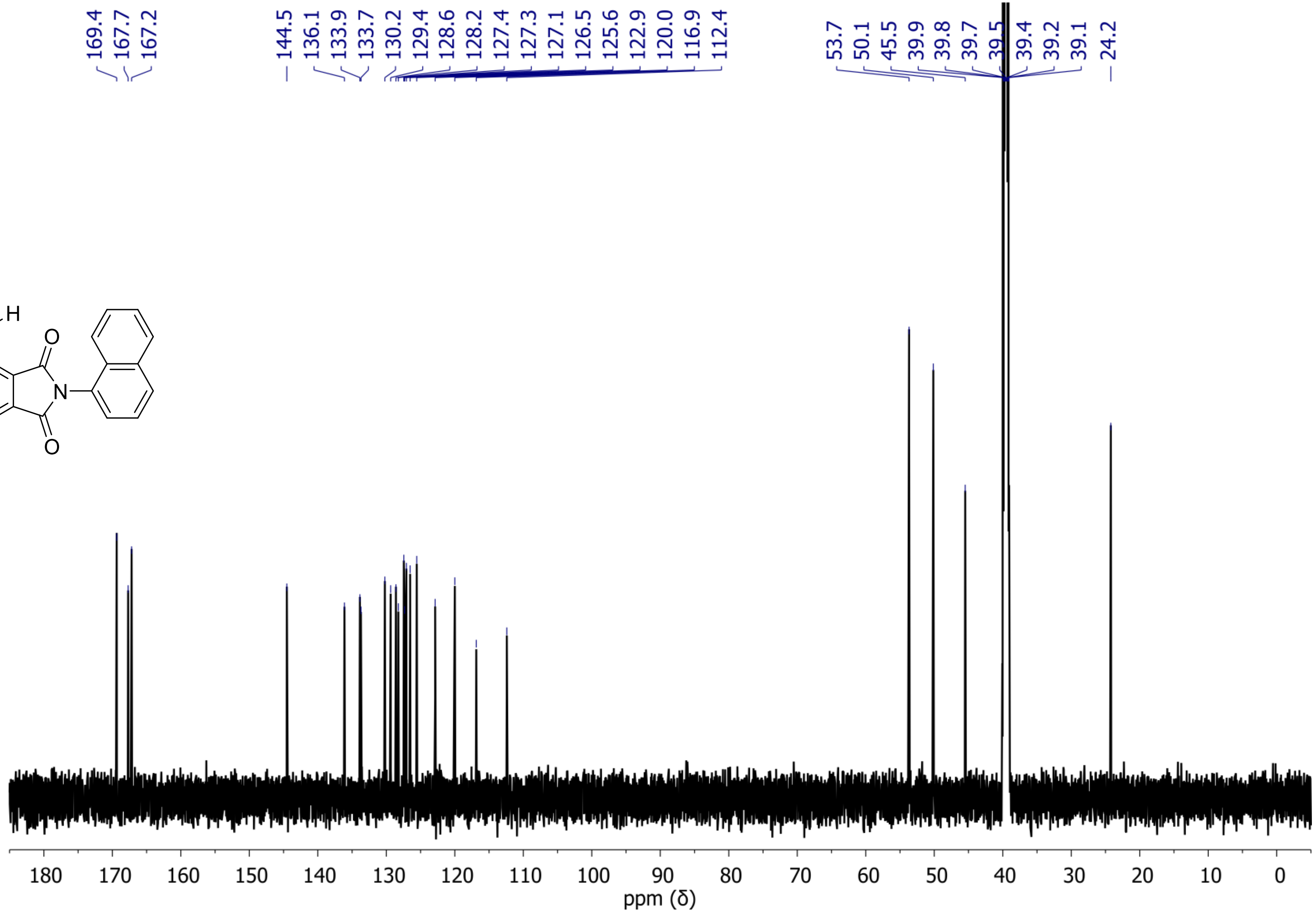

Figure S50. ^{1}H NMR Spectrum (400 MHz, DMSO-d_6) for phthalimide **3e**

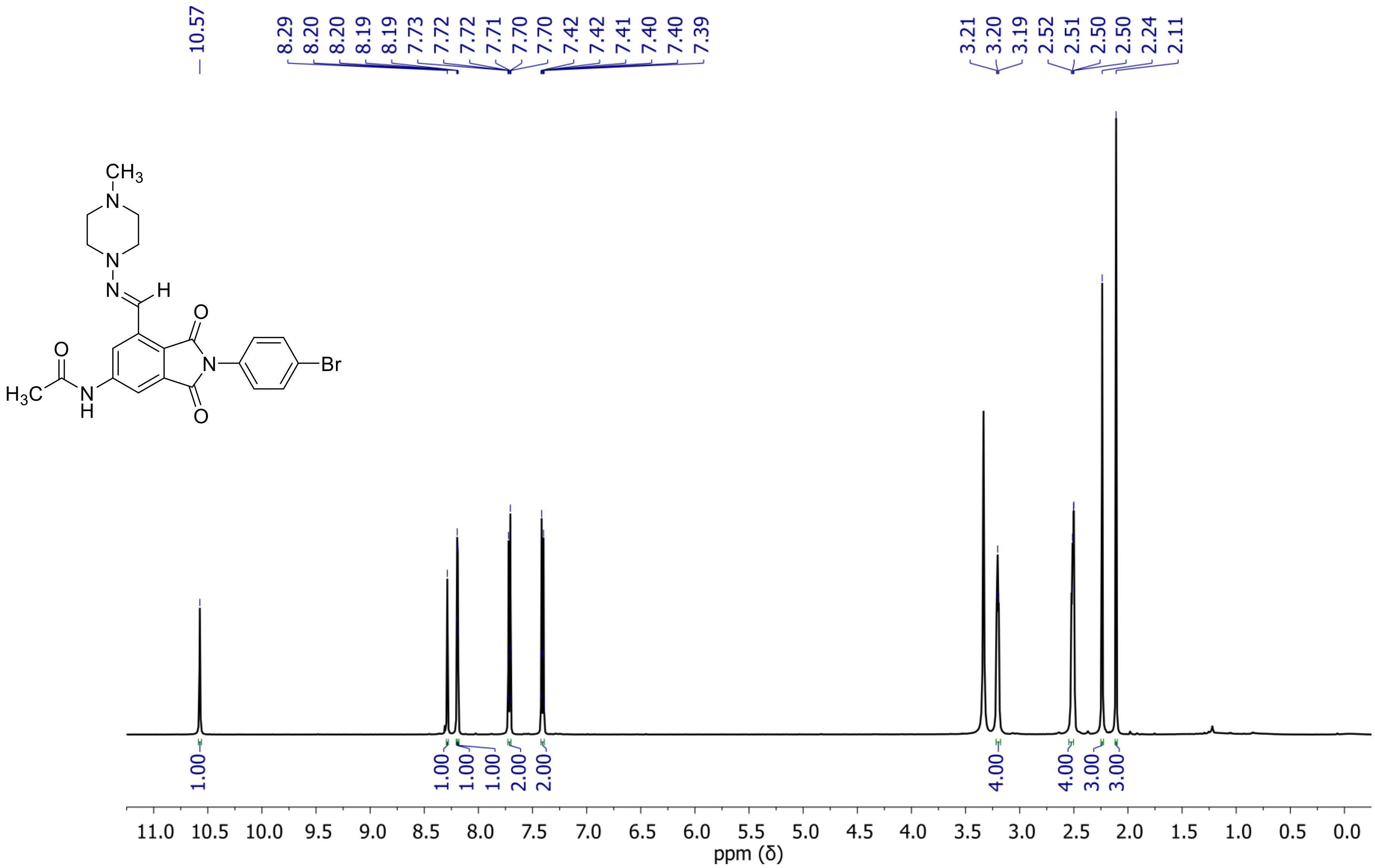

Figure S51. ^{13}C NMR Spectrum (100 MHz, DMSO-d_6) for phthalimide **3e**

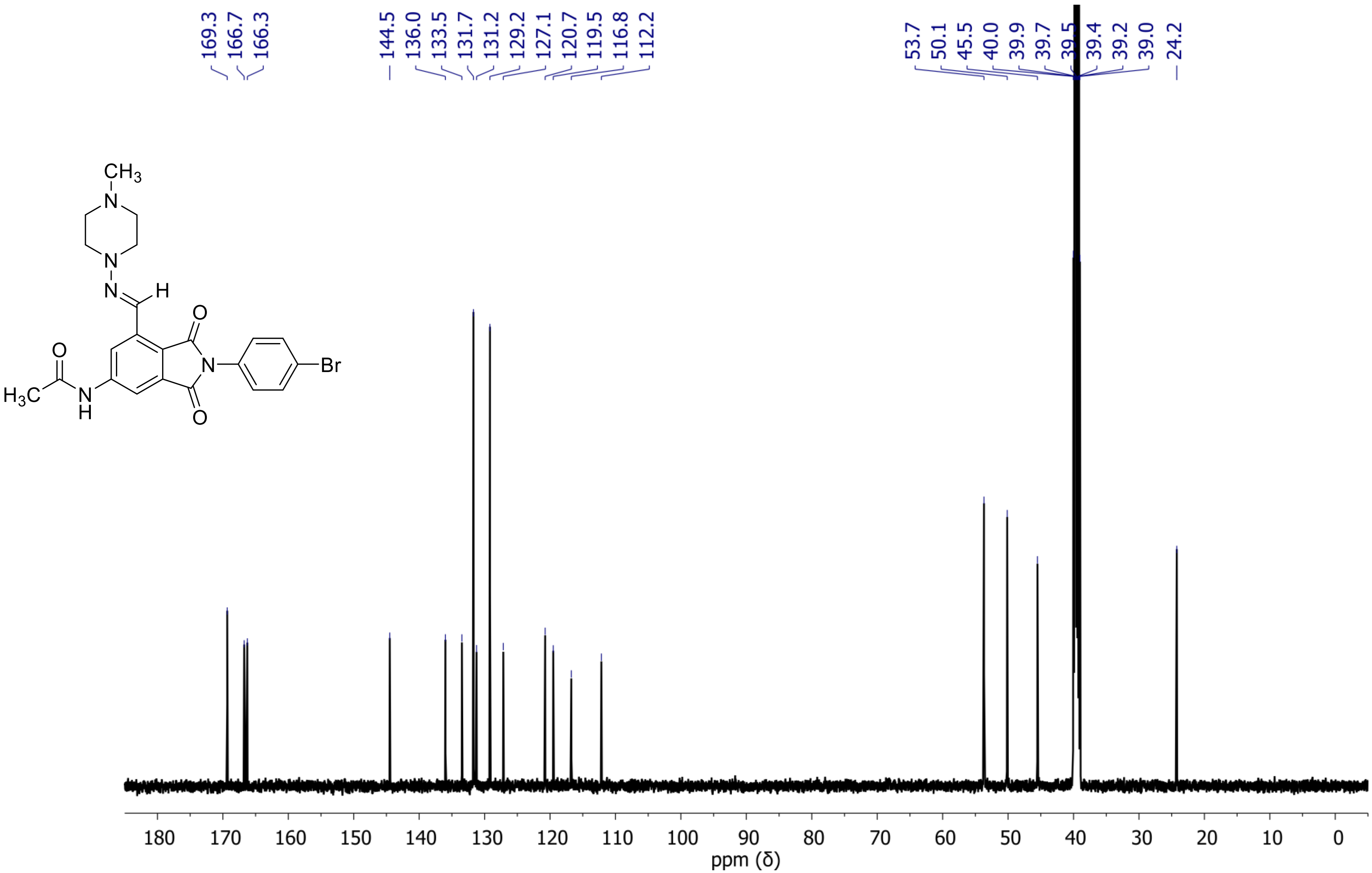

Figure S52. ^{1}H NMR Spectrum (400 MHz, DMSO-d_6) for phthalimide **3f**

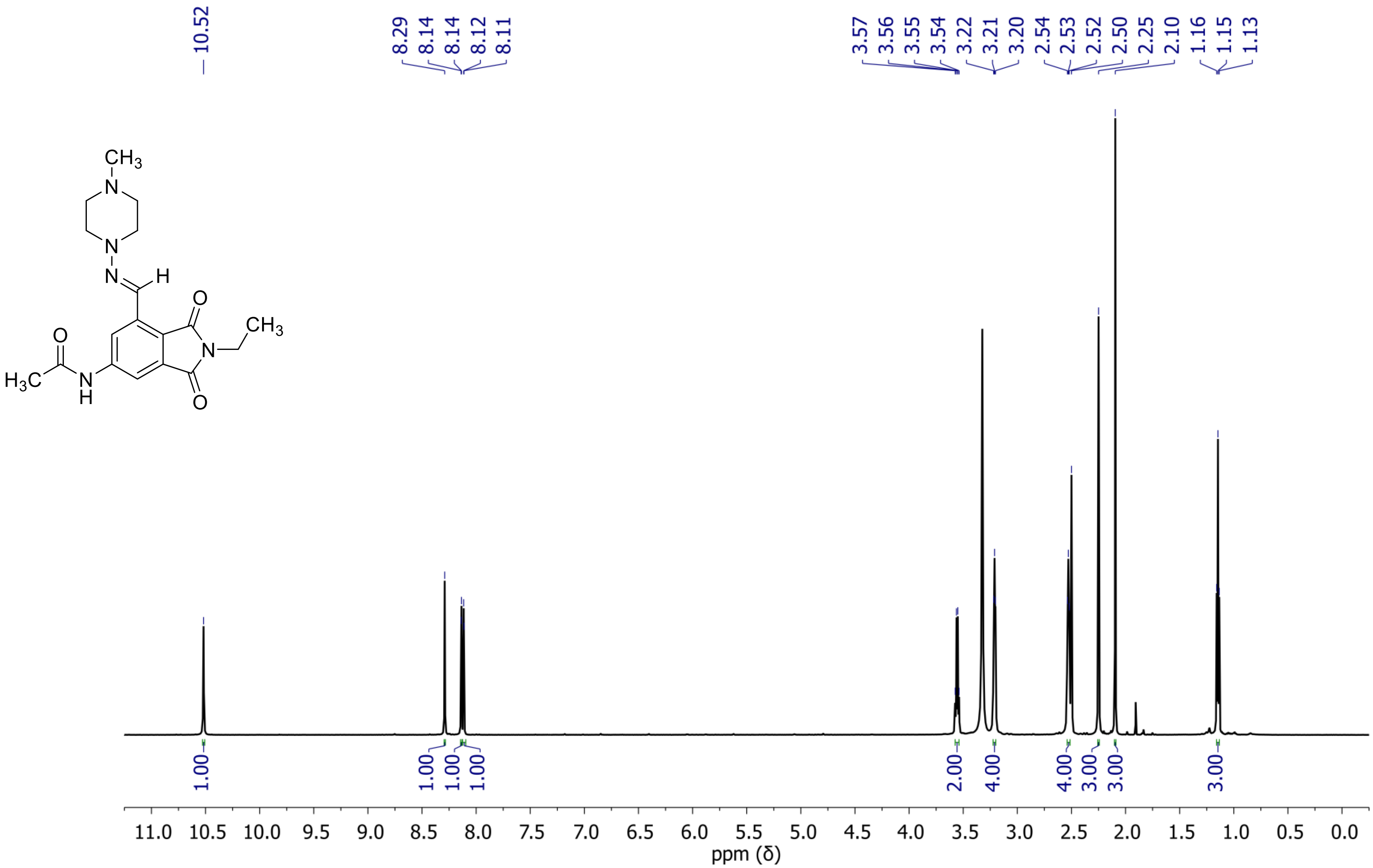

Figure S53. ^{13}C NMR Spectrum (100 MHz, DMSO-d_6) for phthalimide **3f**

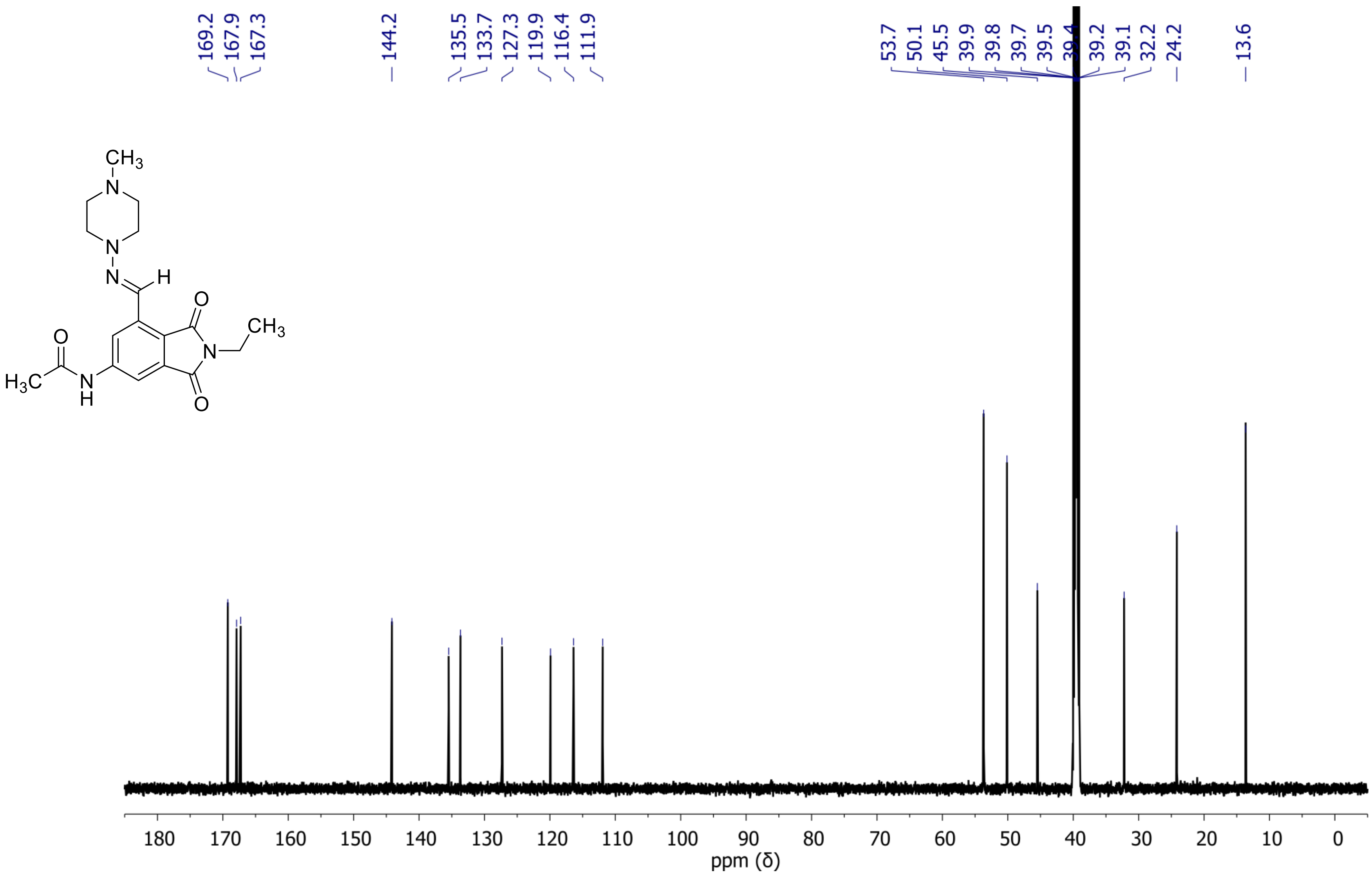

Figure S54. ^{1}H NMR Spectrum (400 MHz, DMSO-d_6) for phthalimide **3g**

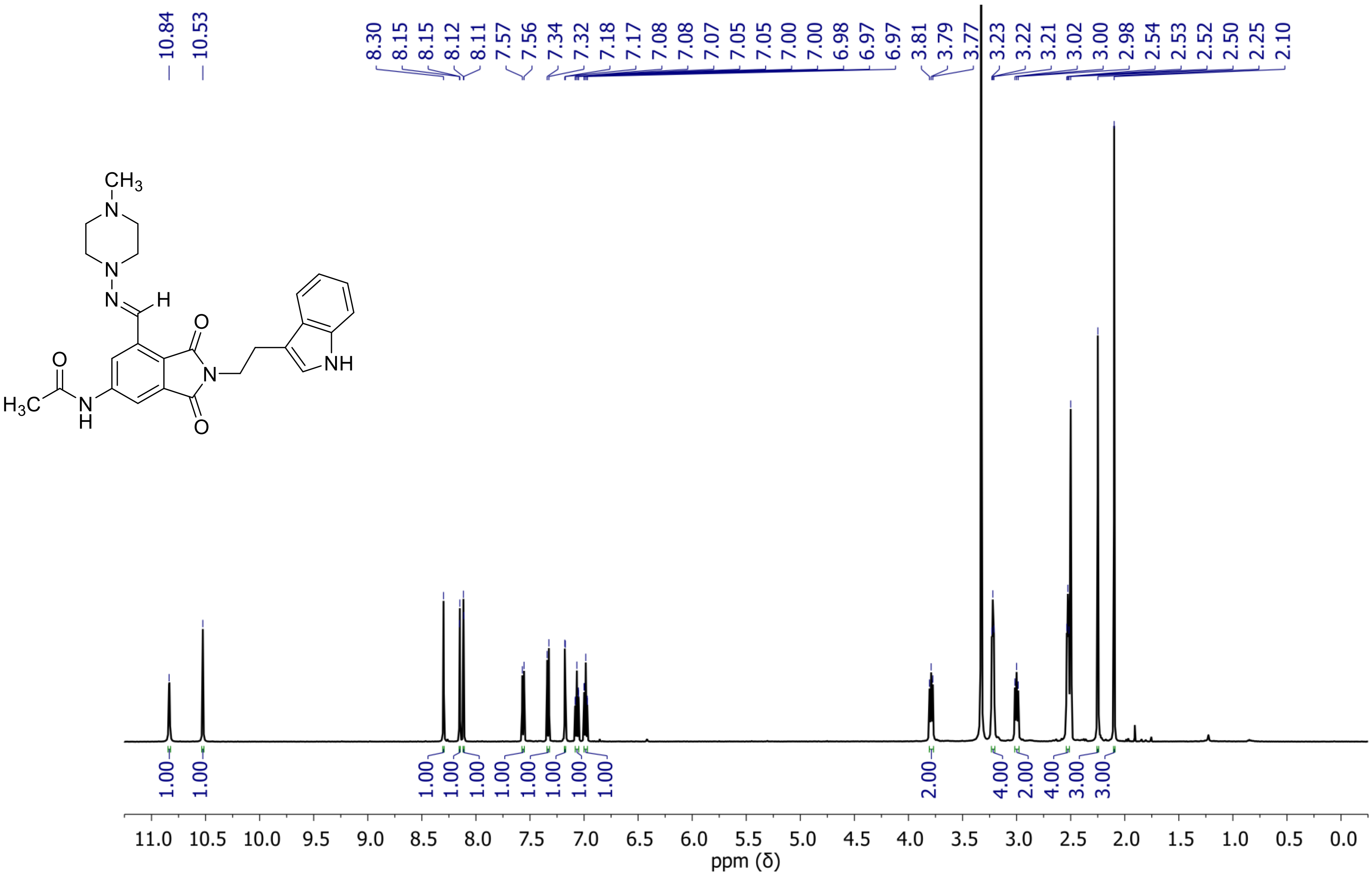

Figure S55. ^{13}C NMR Spectrum (100 MHz, DMSO-d_6) for phthalimide **3g**

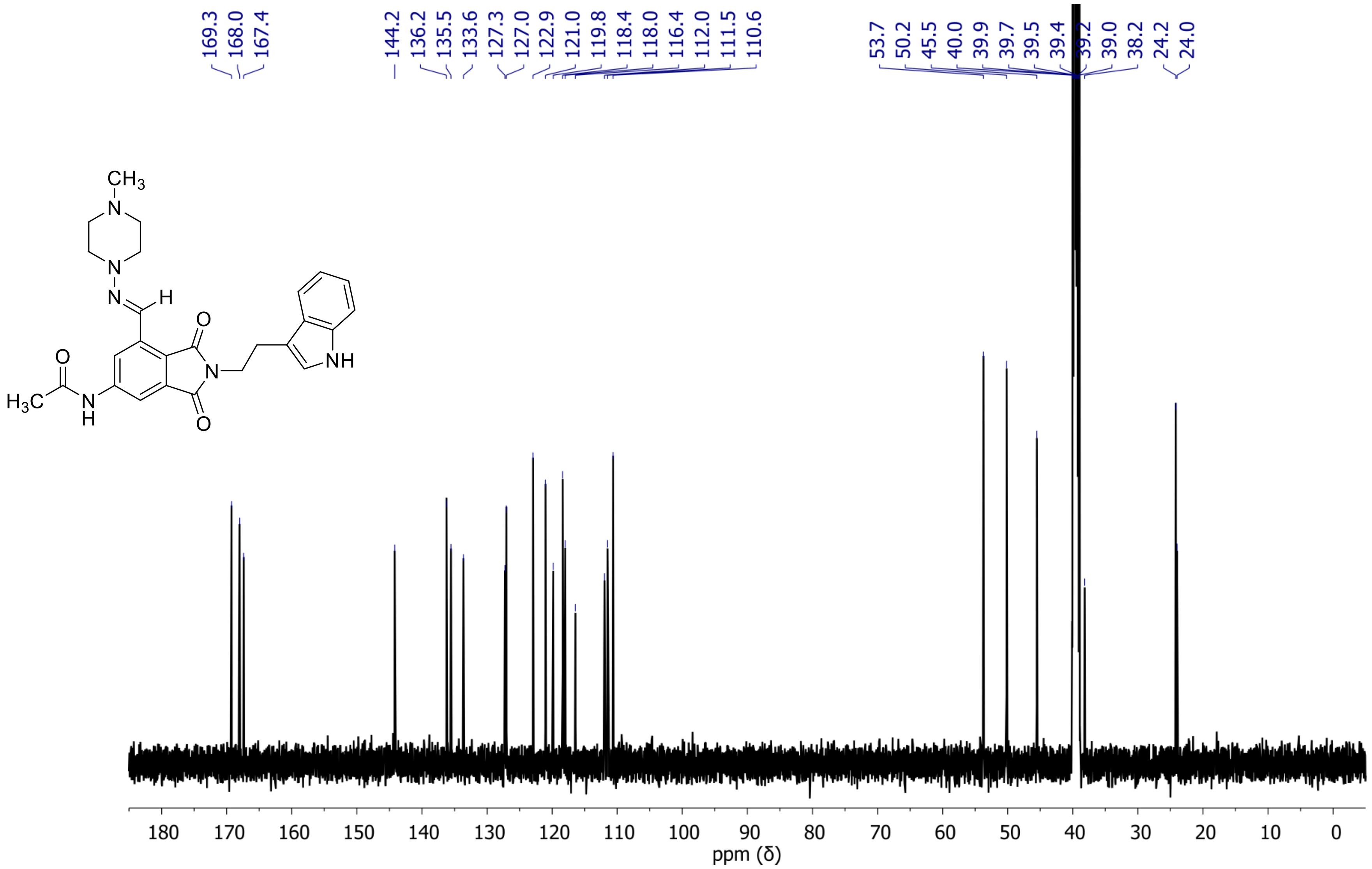

Figure S56. ^{1}H NMR Spectrum (400 MHz, DMSO-d_6) for phthalimide **3h**

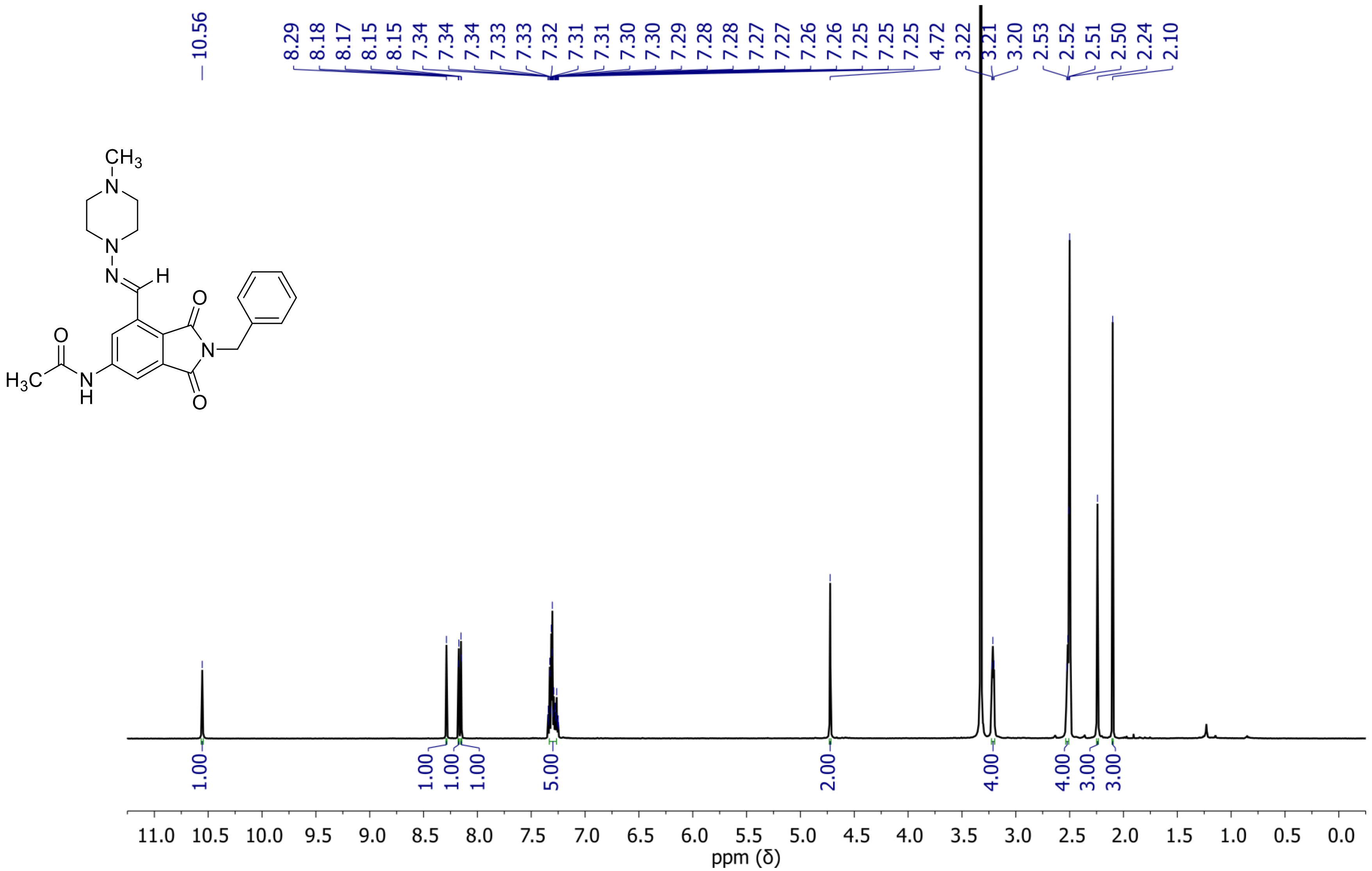

Figure S57. ^{13}C NMR Spectrum (100 MHz, DMSO-d_6) for phthalimide **3h**

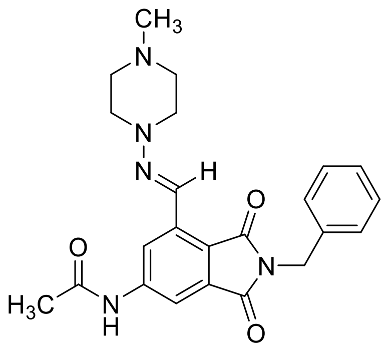

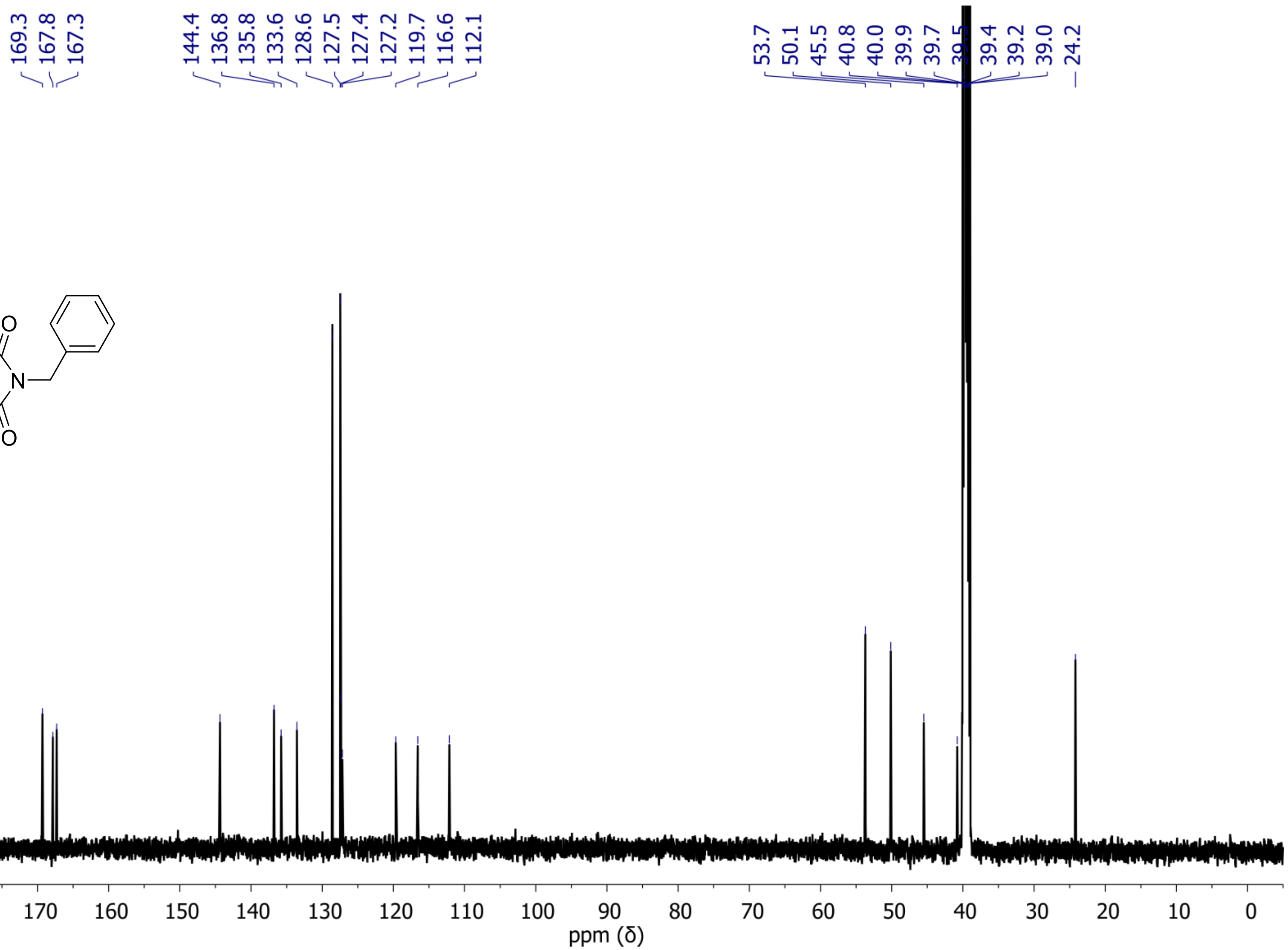

Figure S58. ^{1}H NMR Spectrum (400 MHz, DMSO-d_6) for phthalimide **3i**

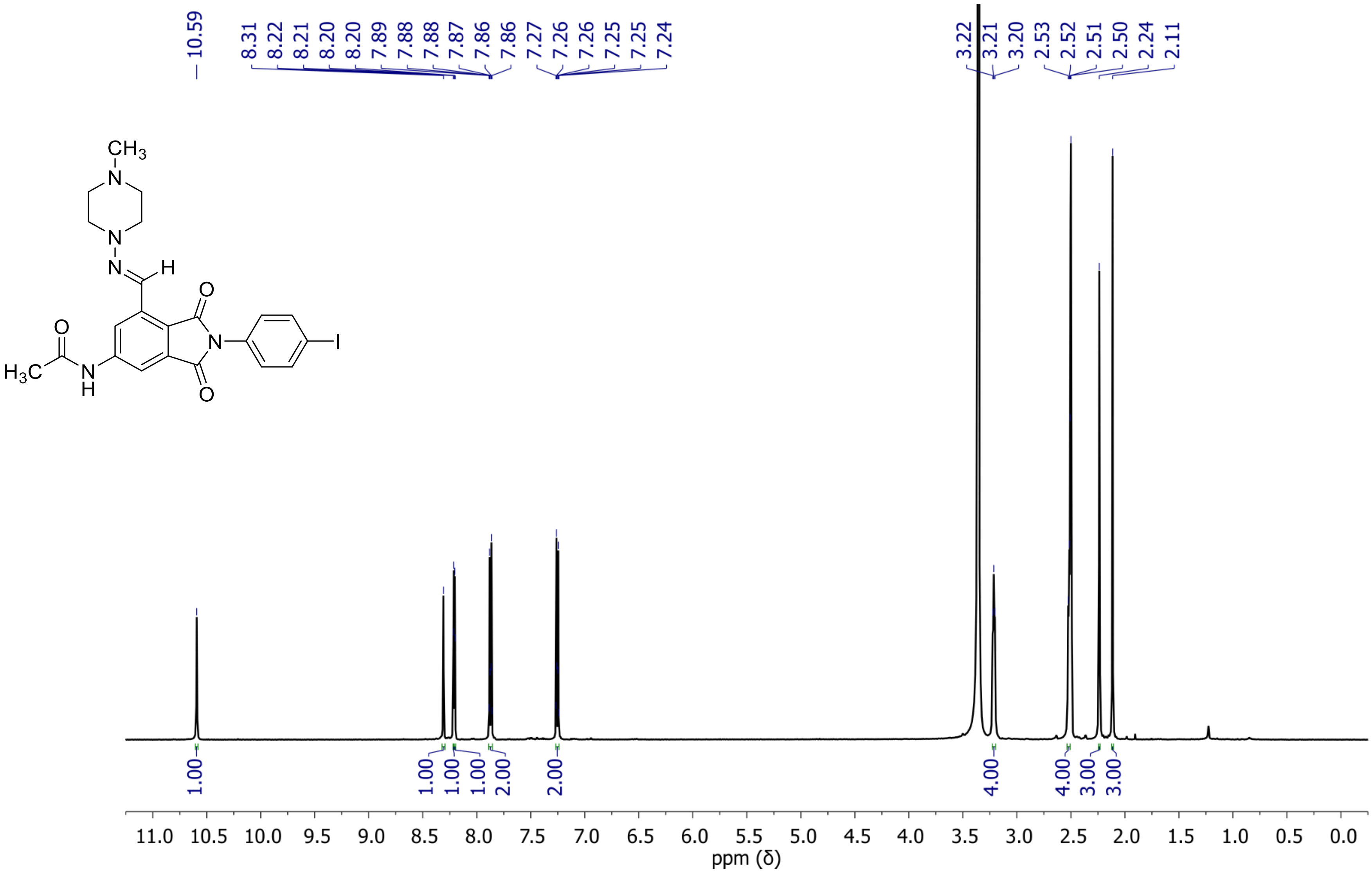

Figure S59. ^{13}C NMR Spectrum (100 MHz, DMSO-d_6) for phthalimide **3i**

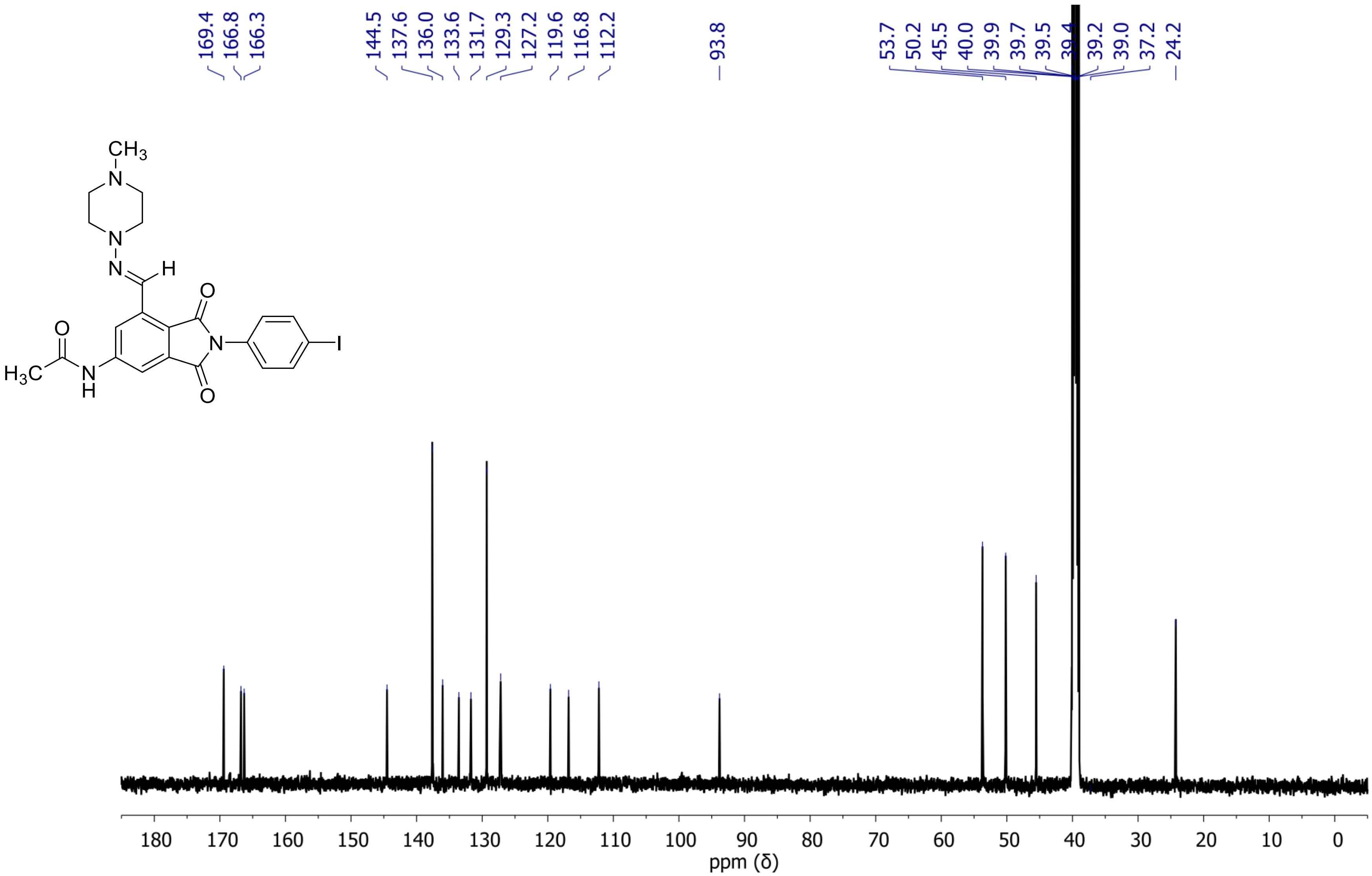

Figure S60. ^{1}H NMR Spectrum (400 MHz, DMSO-d_6) for phthalimide **3j**

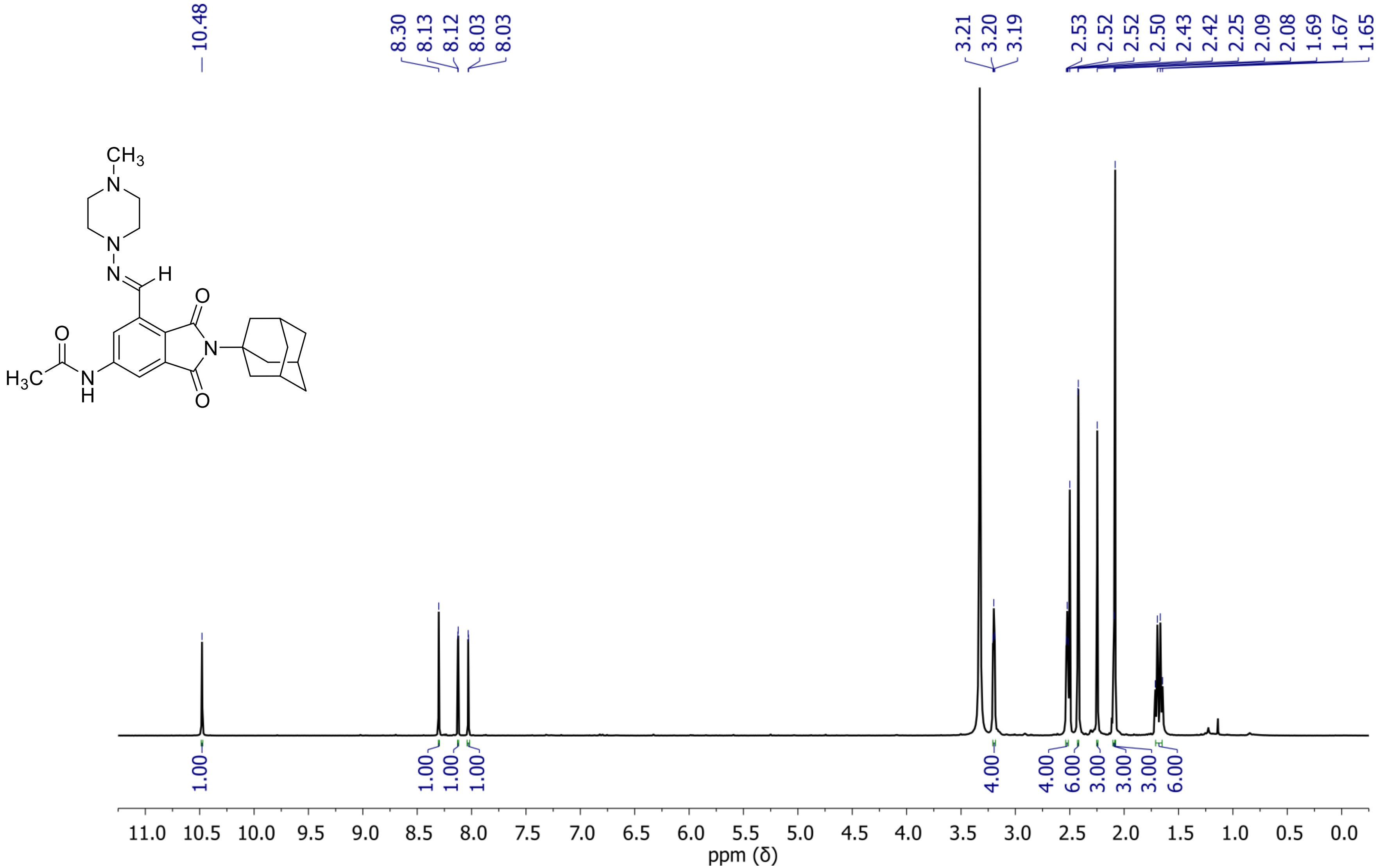

Figure S61. ^{13}C NMR Spectrum (100 MHz, DMSO-d_6) for phthalimide **3j**

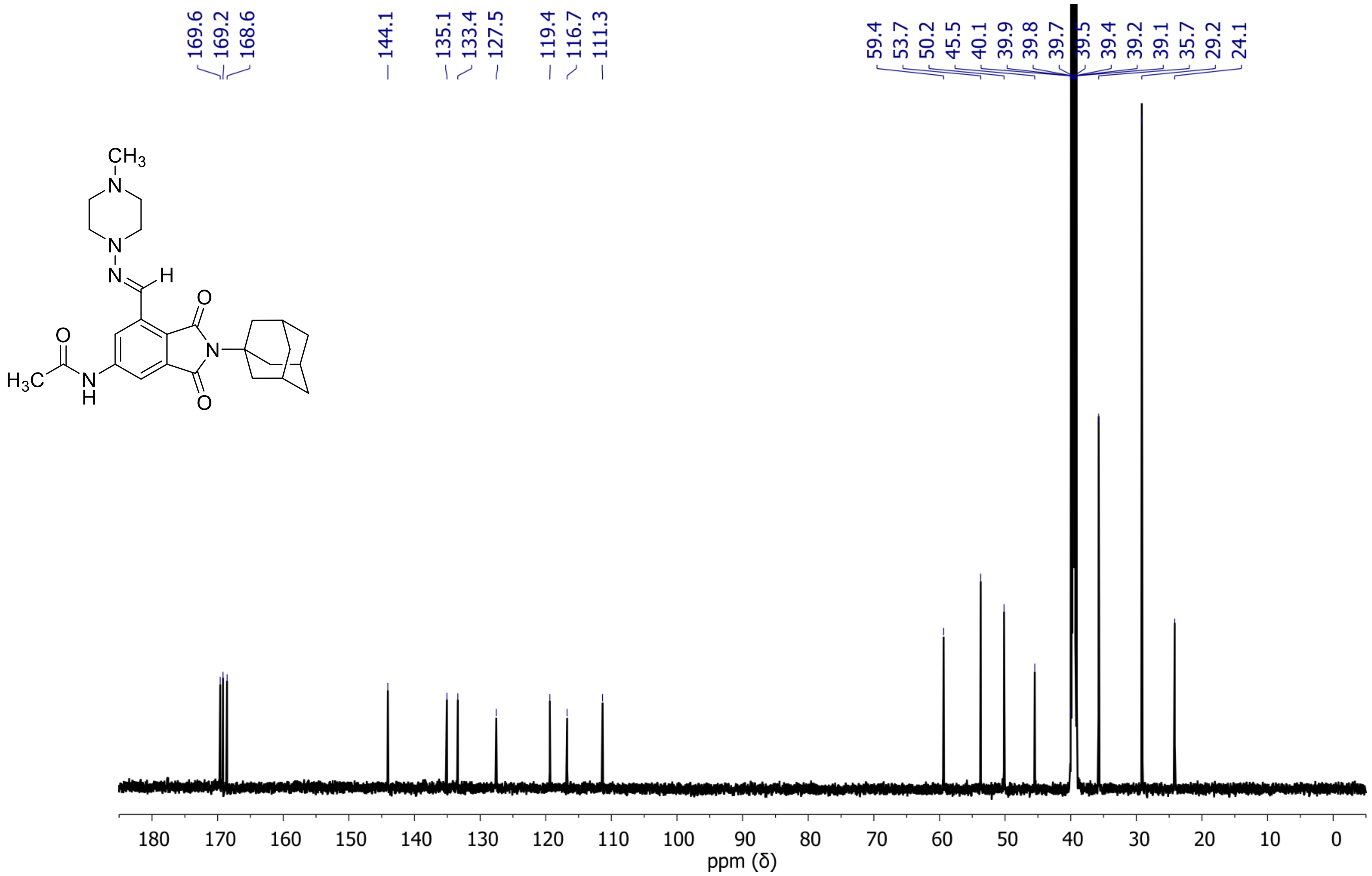

Figure S62. ^{1}H NMR Spectrum (400 MHz, DMSO-d_6) for phthalimide **3k**

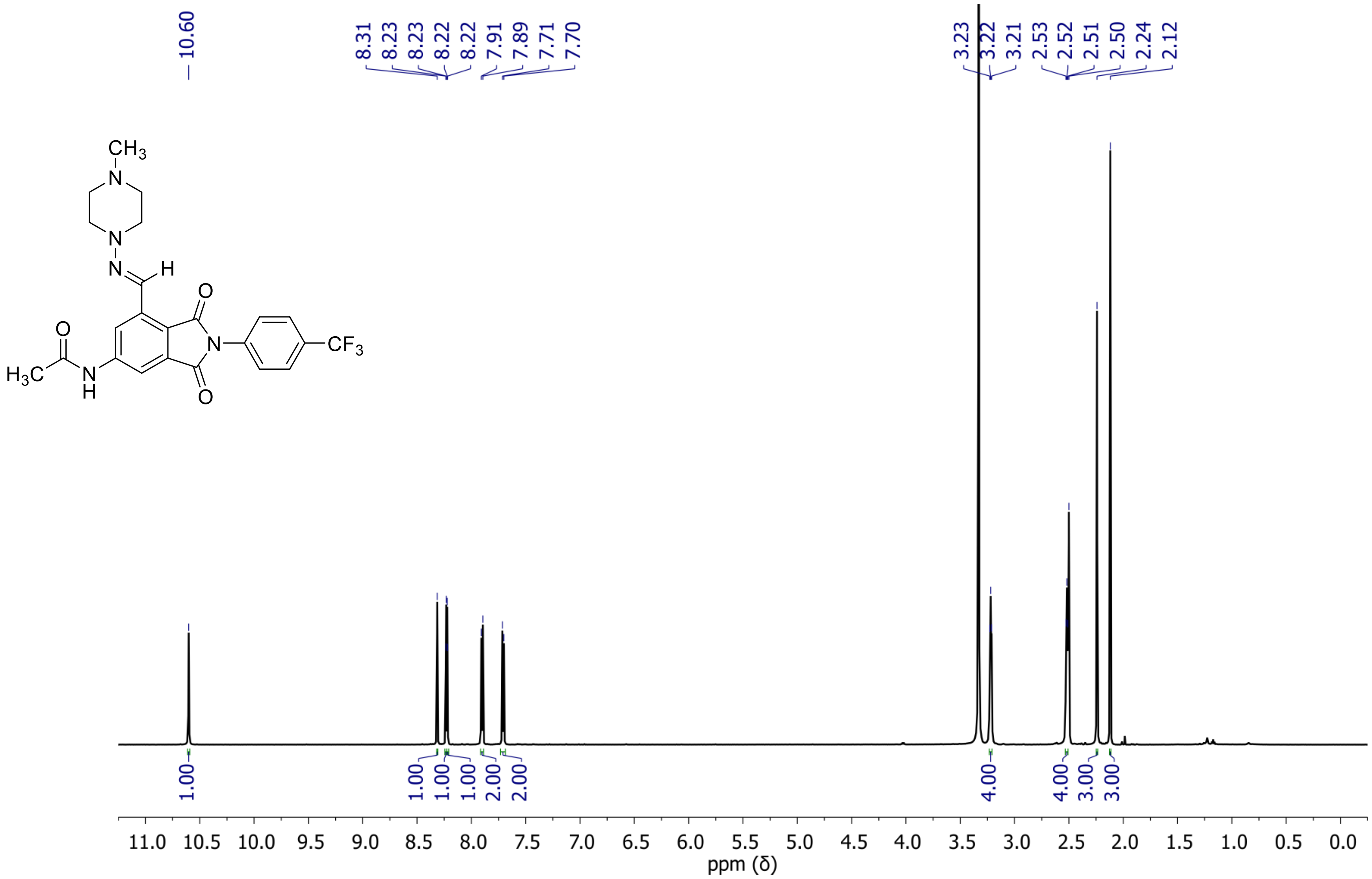

Figure S63. ^{19}F NMR Spectrum (376 MHz, DMSO-d_6) for phthalimide **3k**

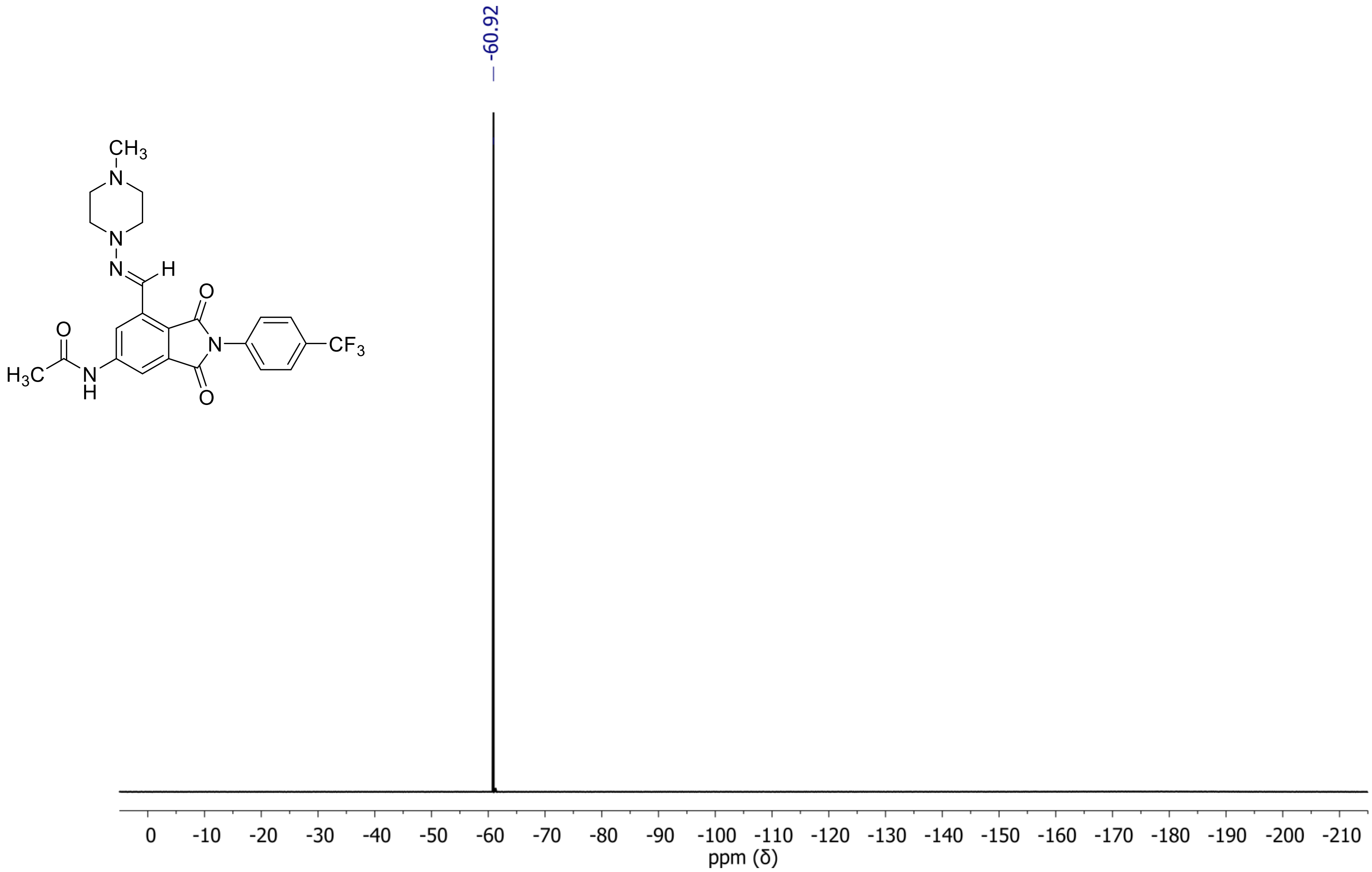

Figure S64. ^{13}C NMR Spectrum (100 MHz, DMSO-d_6) for phthalimide **3k**

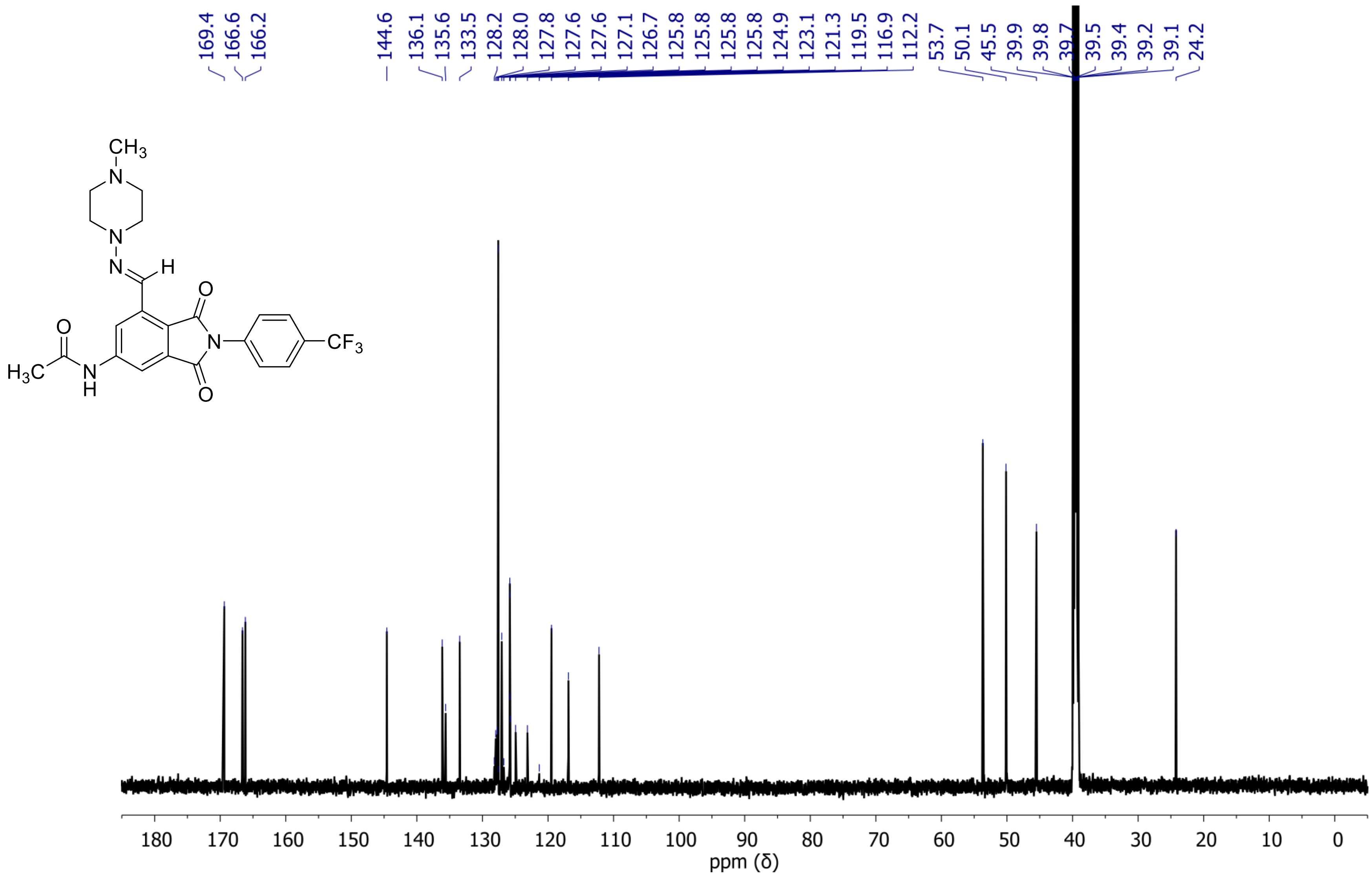

Figure S65. ^{1}H NMR Spectrum (400 MHz, DMSO-d_6) for phthalimide **3l**

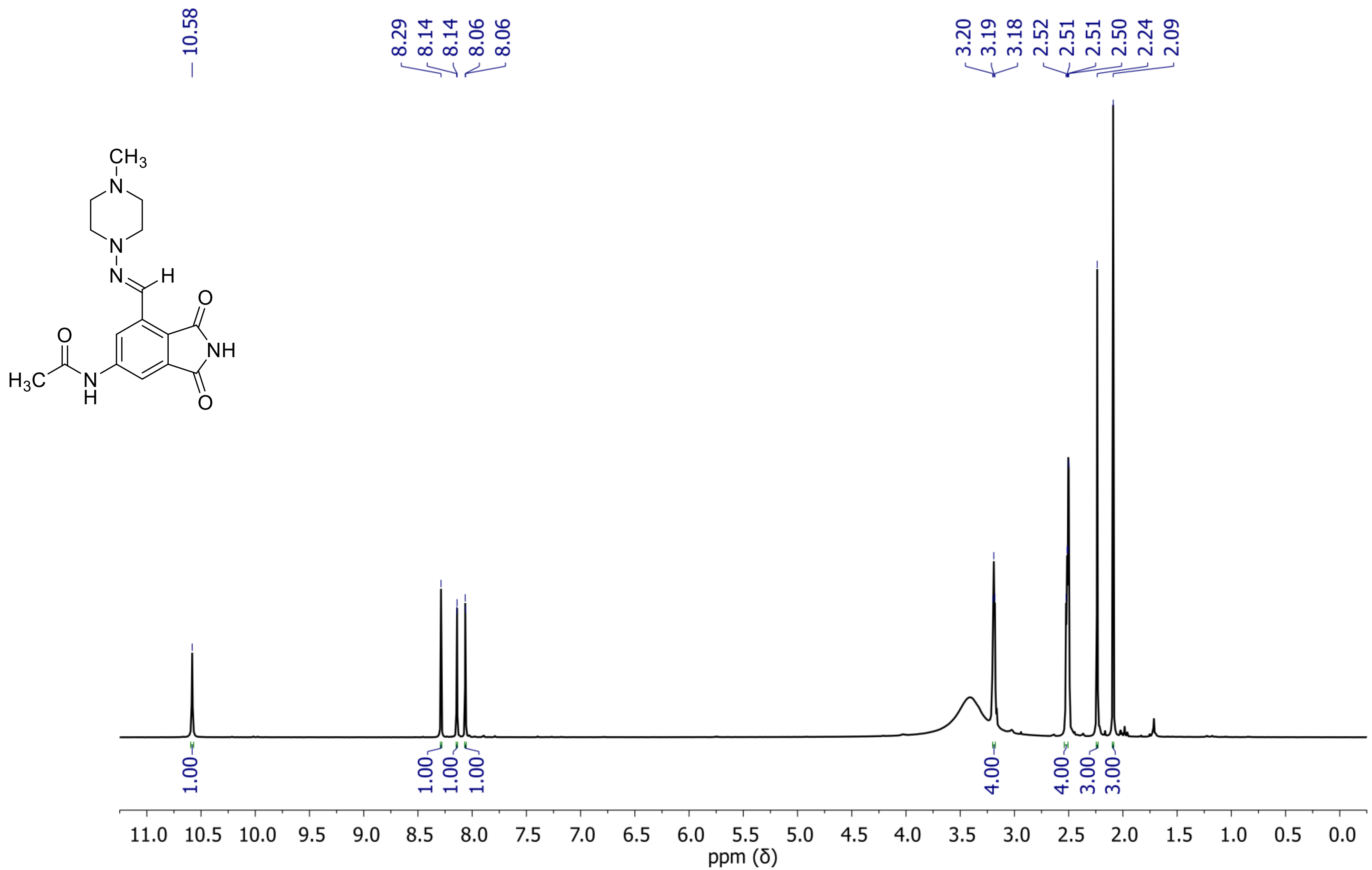

Figure S66. ^{13}C NMR Spectrum (100 MHz, DMSO-d_6) for phthalimide **3l**

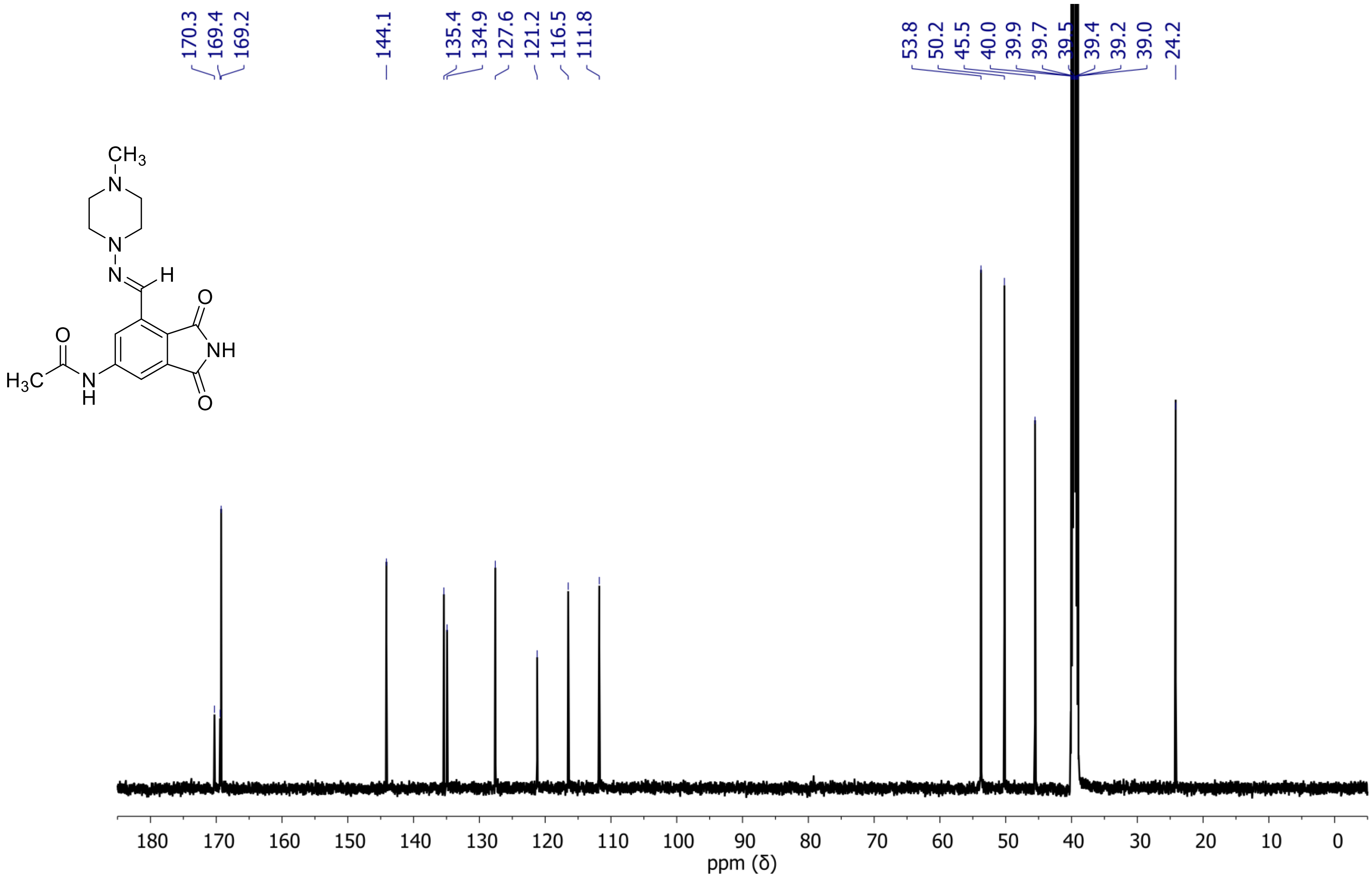

Figure S67. ^{1}H NMR Spectrum (400 MHz, DMSO-d_6) for phthalimide **4**

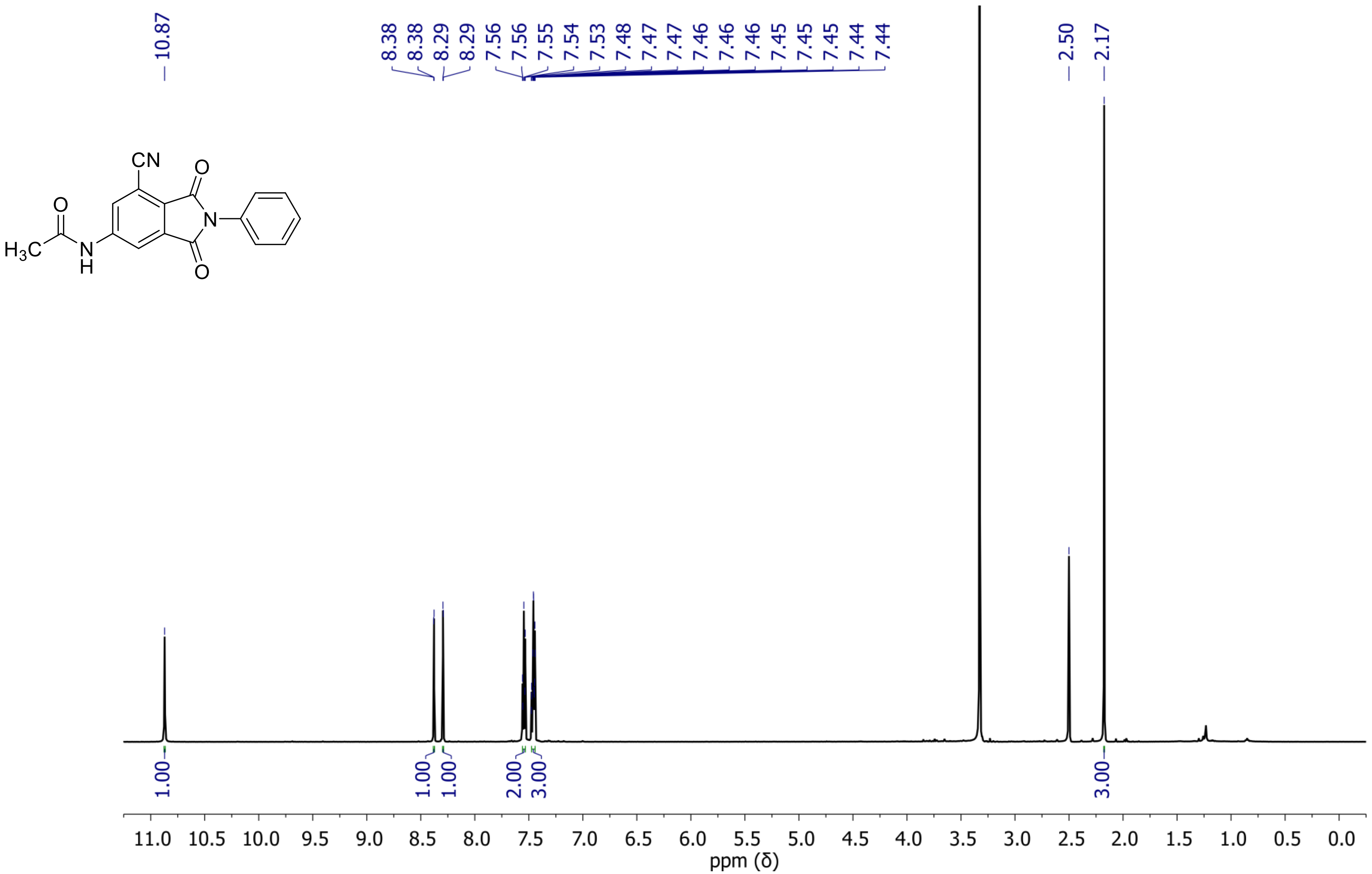

Figure S68. ^{13}C NMR Spectrum (100 MHz, DMSO-d_6) for phthalimide **4**

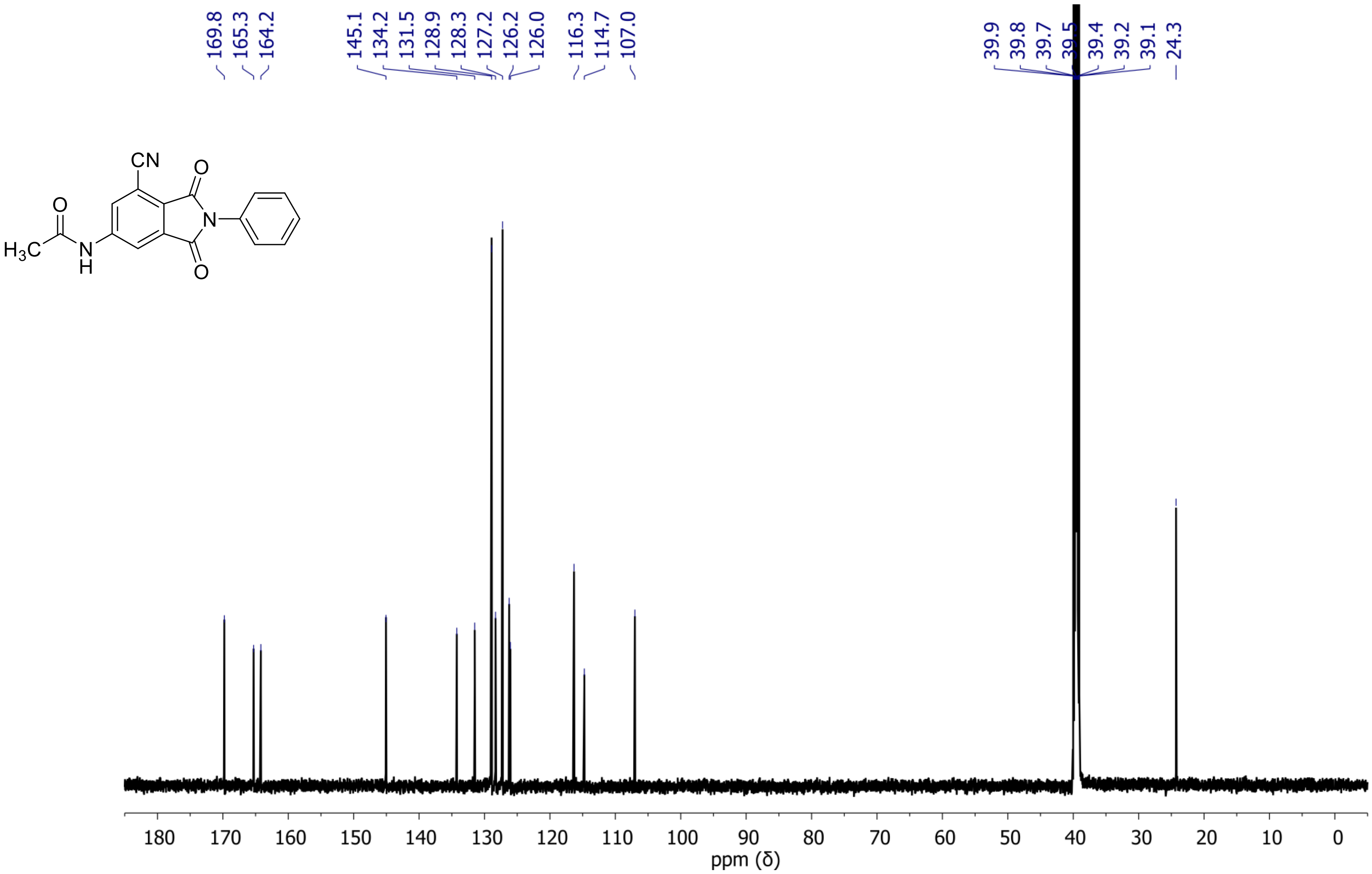

Figure S69. ^{1}H NMR Spectrum (400 MHz, DMSO-d_6) for phthalimide **5**

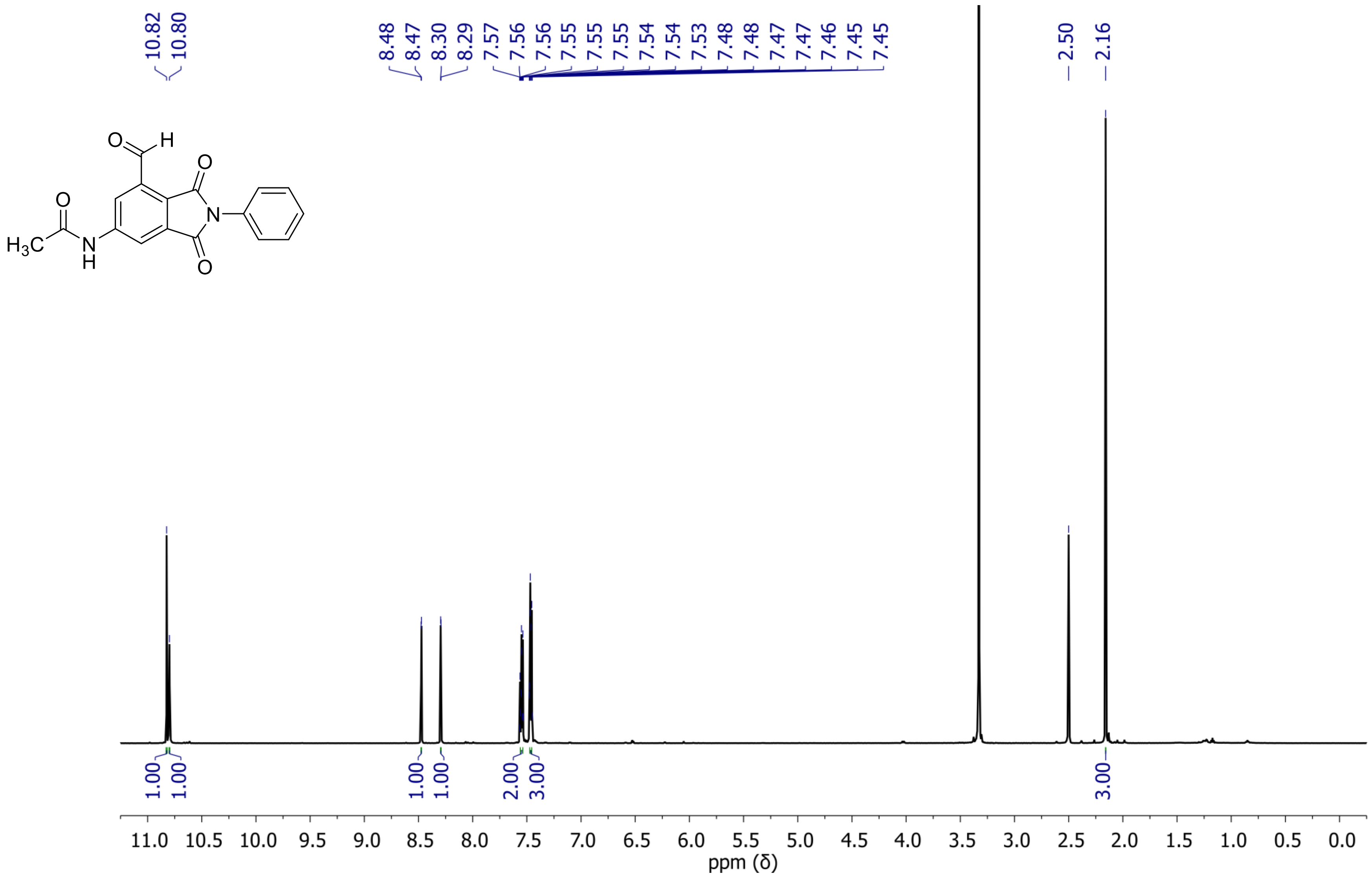

Figure S70. ^{13}C NMR Spectrum (100 MHz, DMSO-d_6) for phthalimide **5**

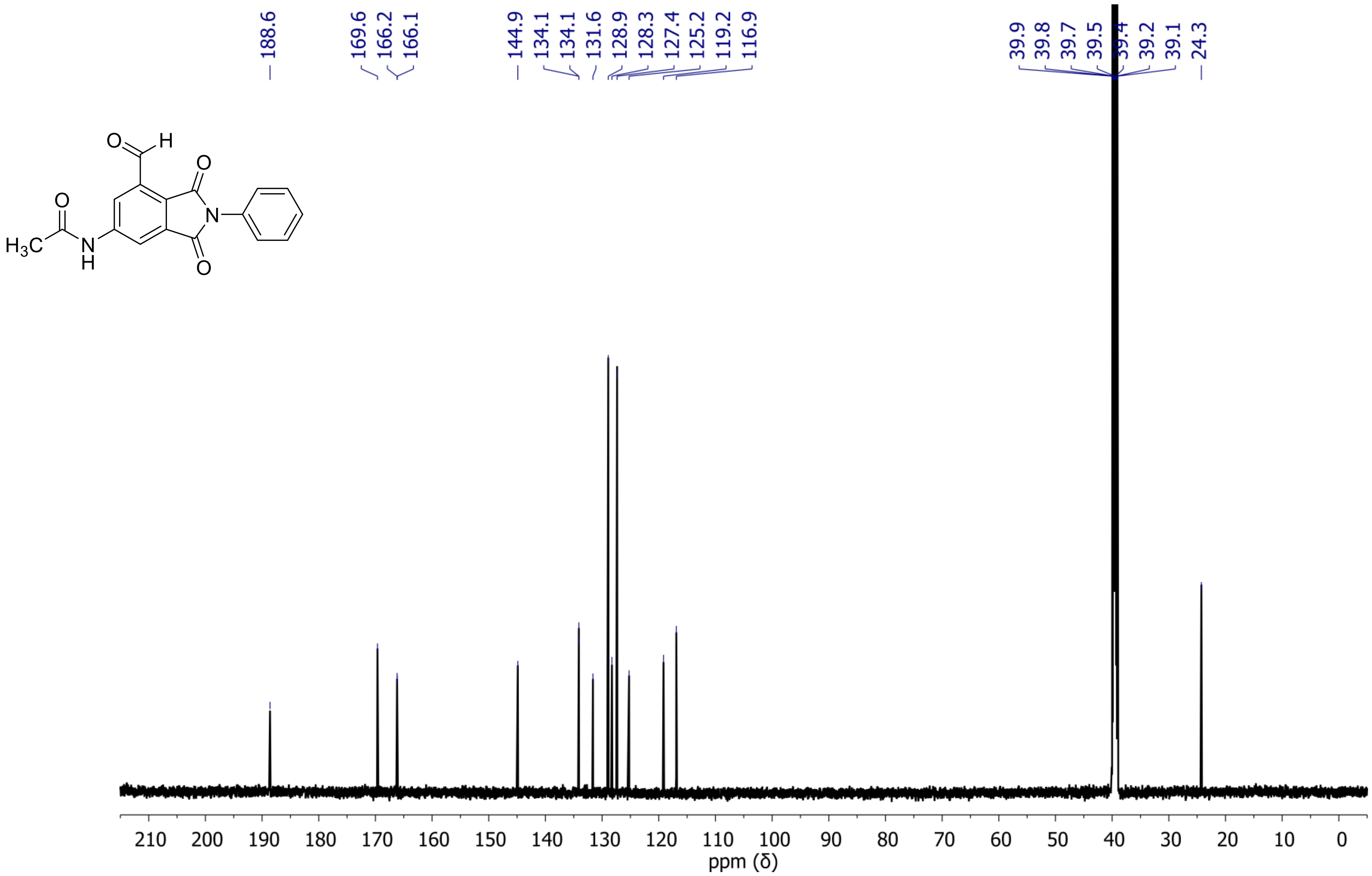

Figure S71. ^{1}H NMR Spectrum (400 MHz, DMSO-d_6) for phthalimide **6**

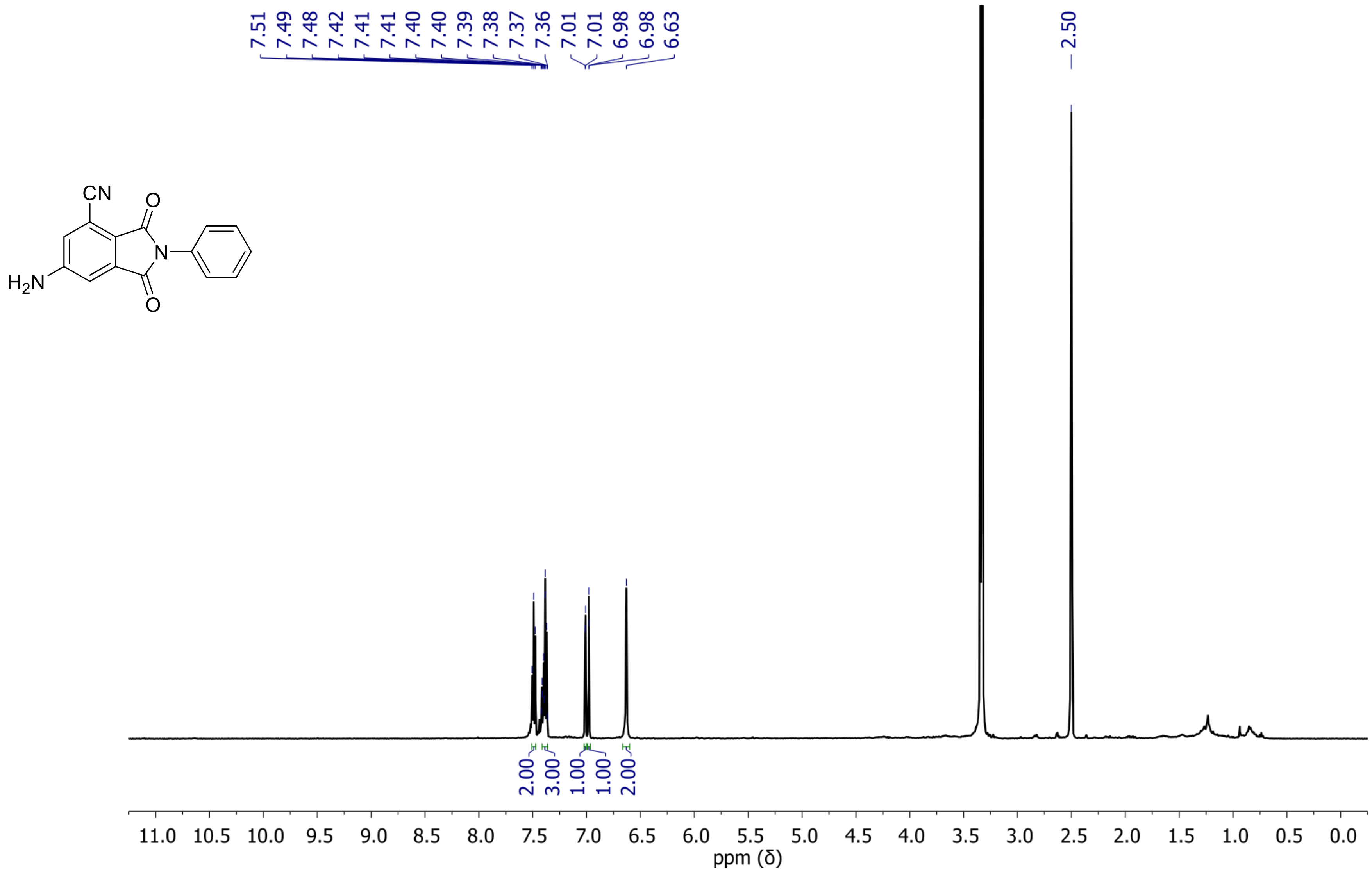

Figure S72. ^{13}C NMR Spectrum (100 MHz, DMSO-d_6) for phthalimide **6**

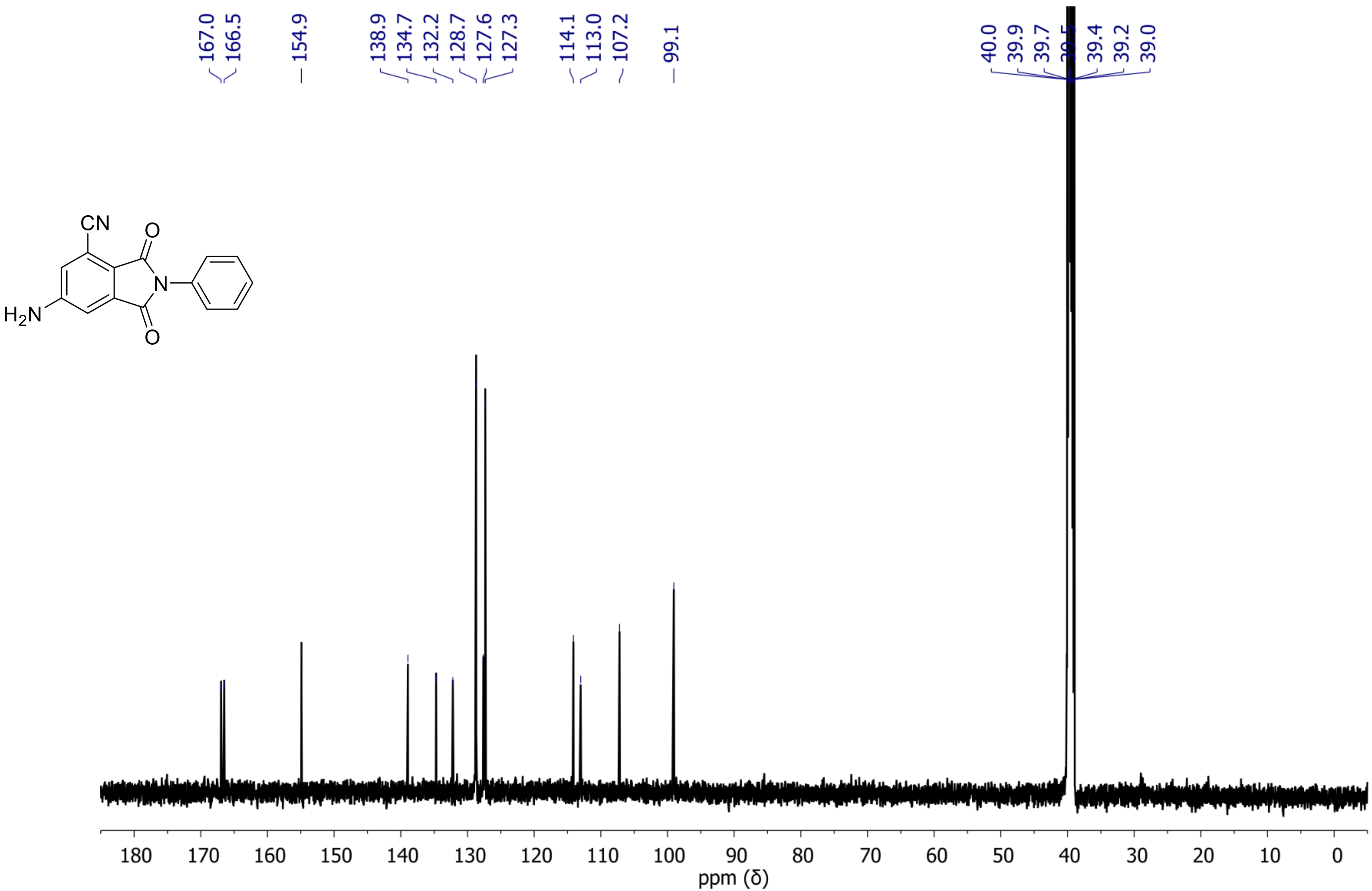

Figure S73. ^{1}H NMR Spectrum (400 MHz, $CDCl_3$) for *N*-(4-nitrophenyl)maleimide (**7b**)

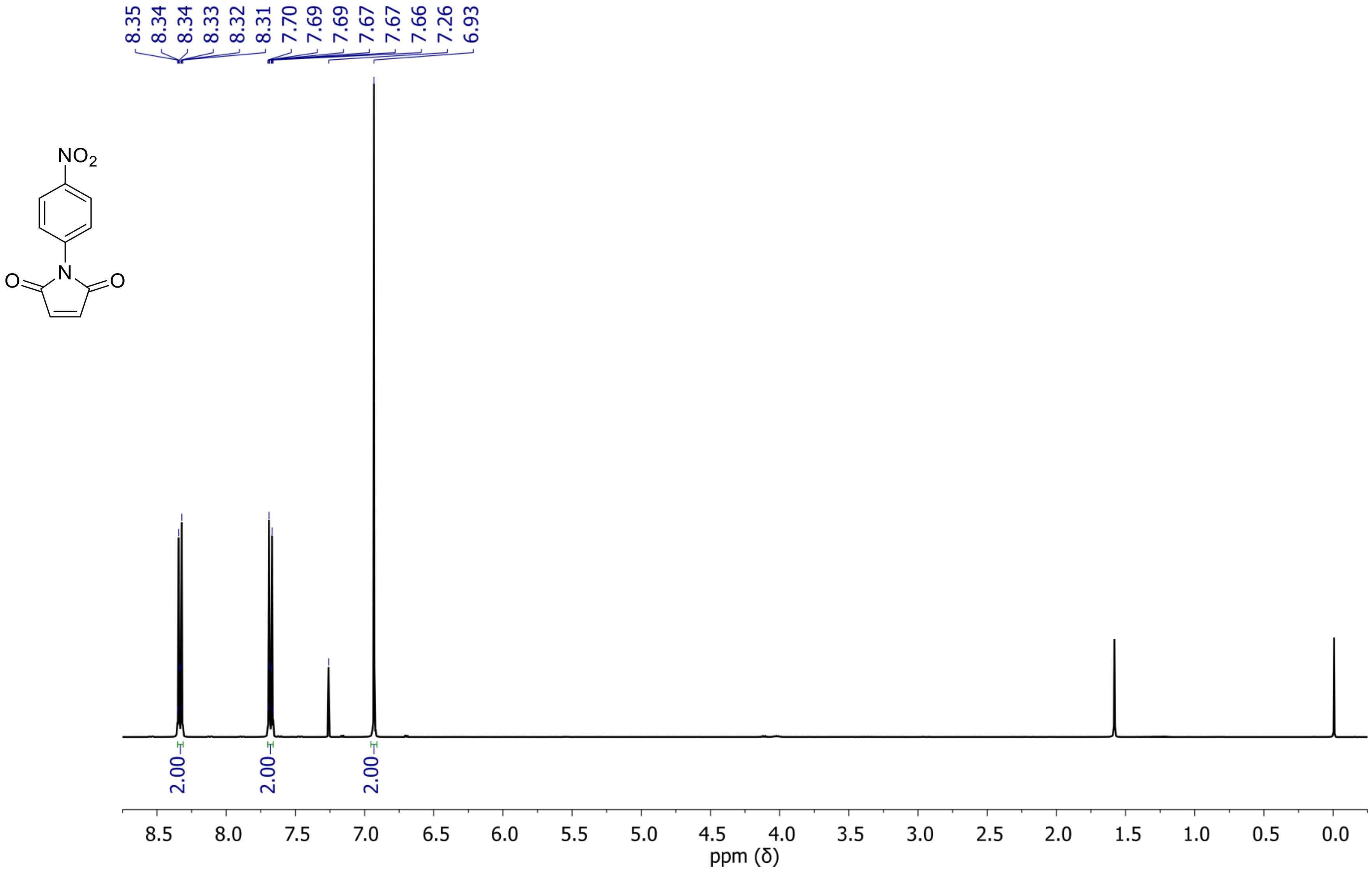

Figure S74. ^{13}C NMR Spectrum (100 MHz, $CDCl_3$) for *N*-(4-nitrophenyl)maleimide (**7b**)

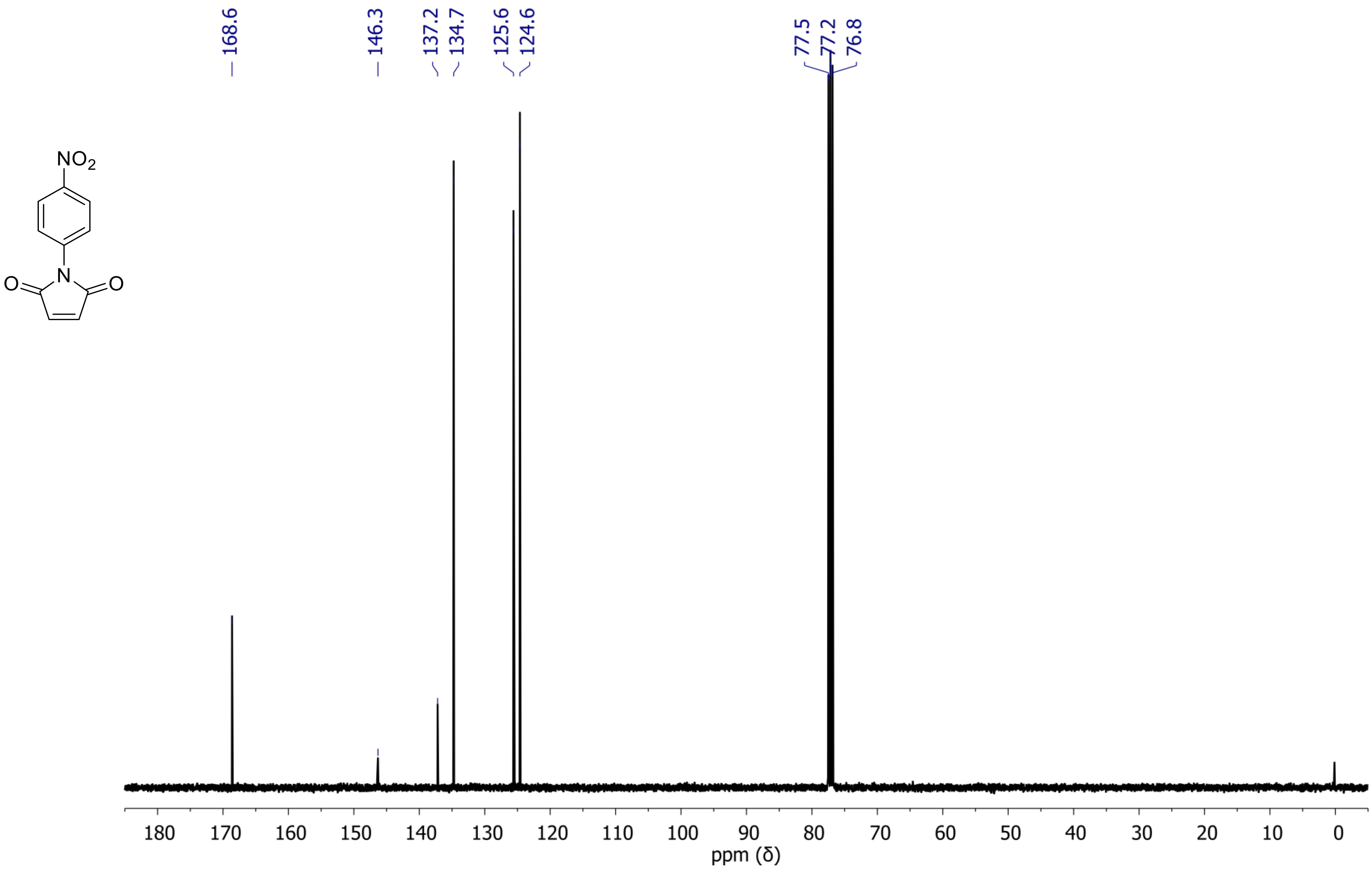

Figure S75. ^{1}H NMR Spectrum (400 MHz, $CDCl_3$) for *N*-(4-cyanophenyl)maleimide (**7c**)

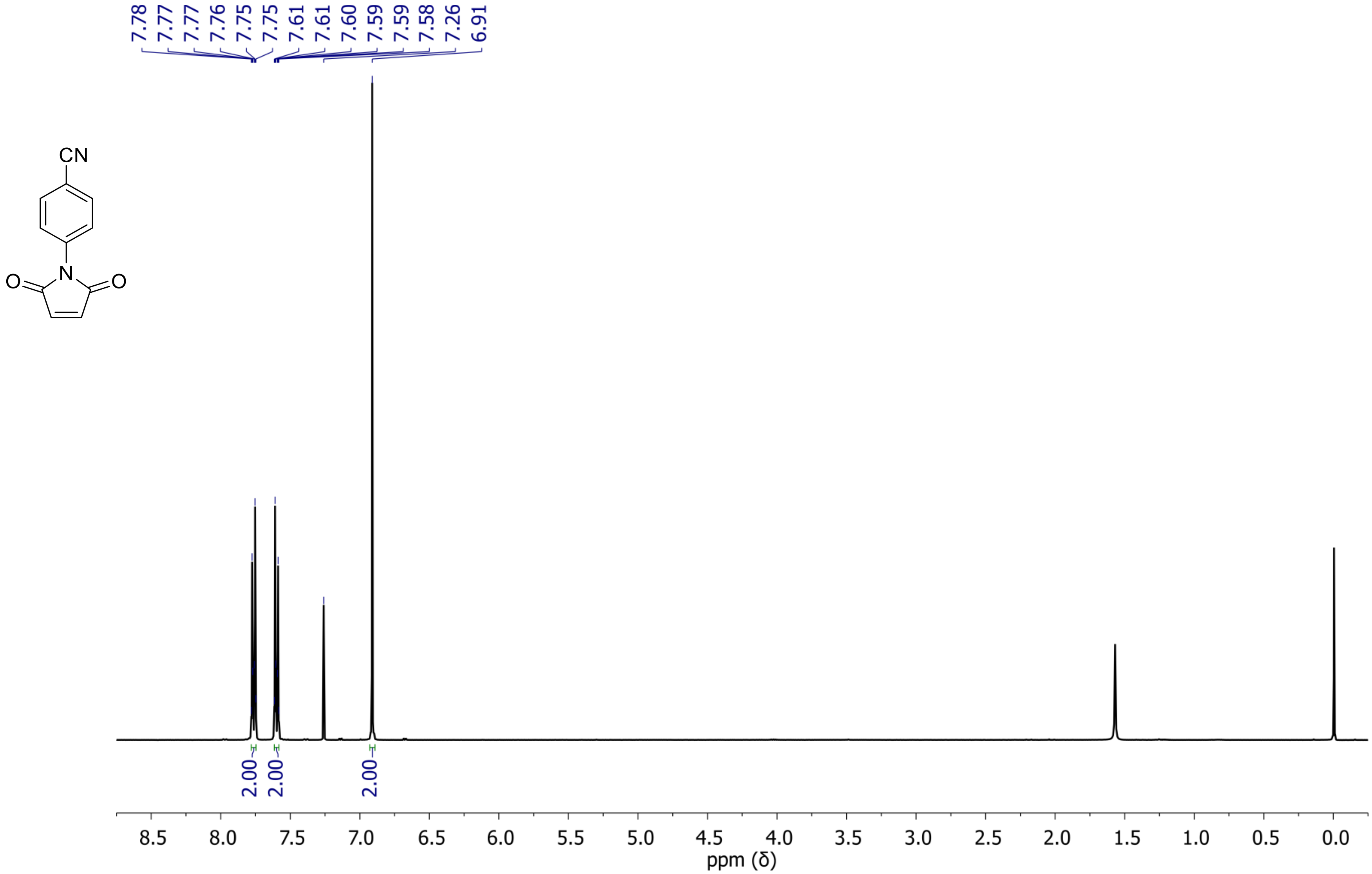

Figure S76. ^{13}C NMR Spectrum (100 MHz, $CDCl_3$) for *N*-(4-cyanophenyl)maleimide (**7c**)

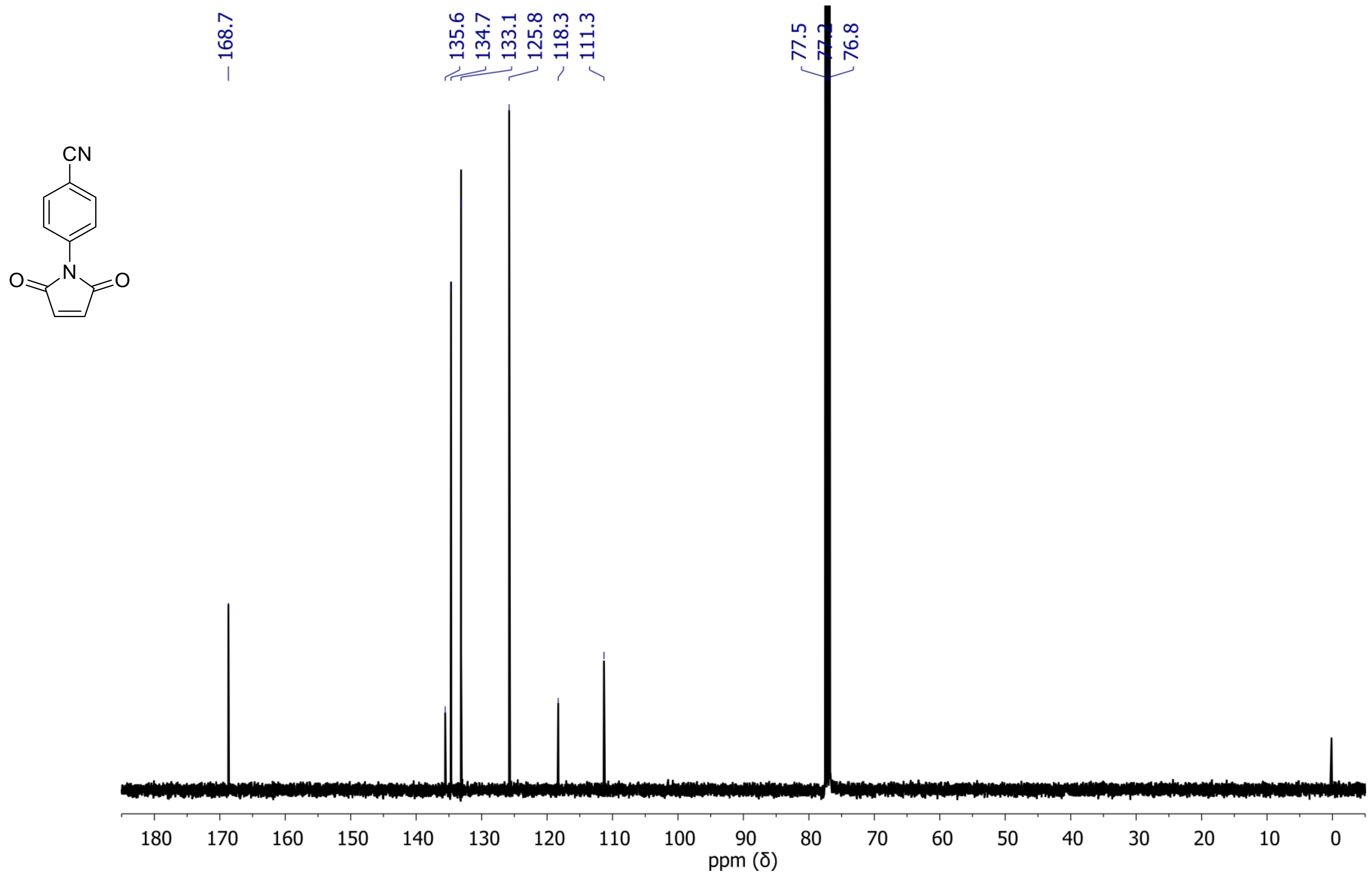

Figure S77. ^{1}H NMR Spectrum (400 MHz, $CDCl_3$) for *N*-(4-(trifluoromethyl)phenyl)maleimide (**7d**)

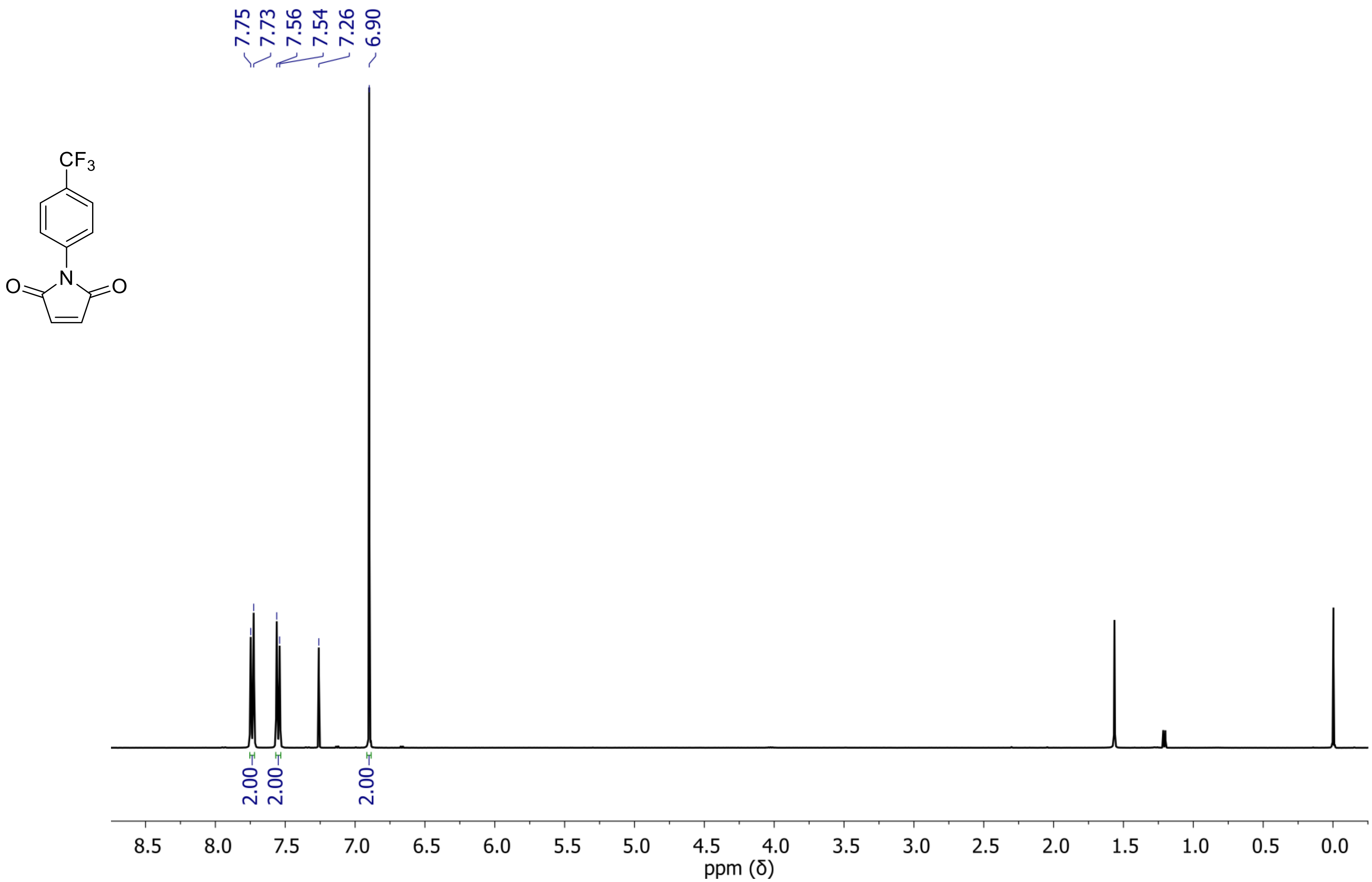

Figure S78. ^{19}F NMR Spectrum (376 MHz, $CDCl_3$) for *N*-(4-(trifluoromethyl)phenyl)maleimide (**7d**)

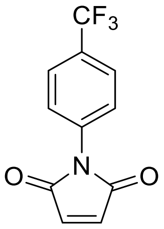

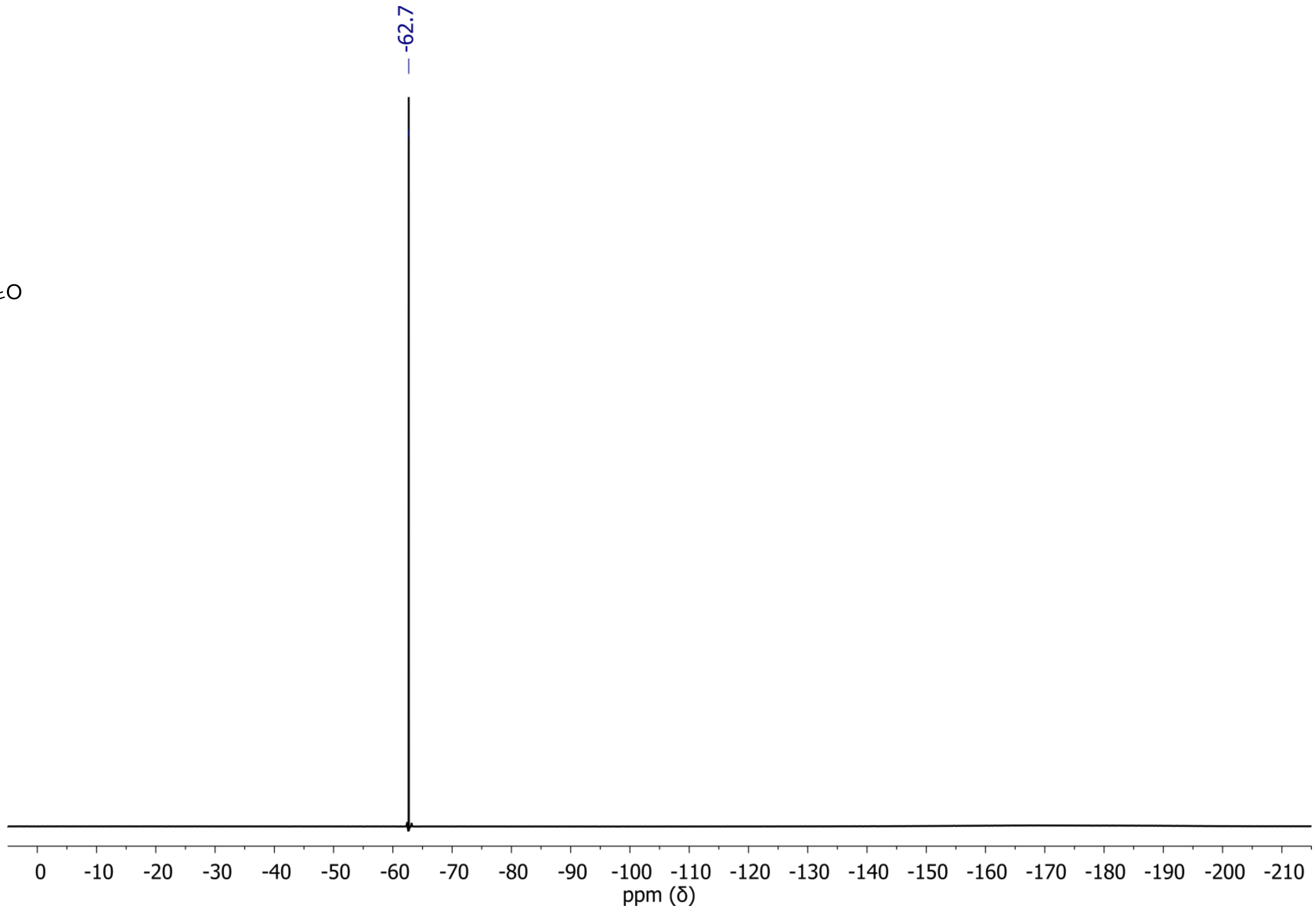

Figure S79. ^{13}C NMR Spectrum (100 MHz, $CDCl_3$) for *N*-(4-(trifluoromethyl)phenyl)maleimide (**7d**)

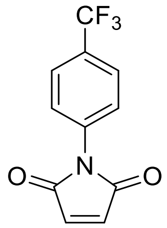

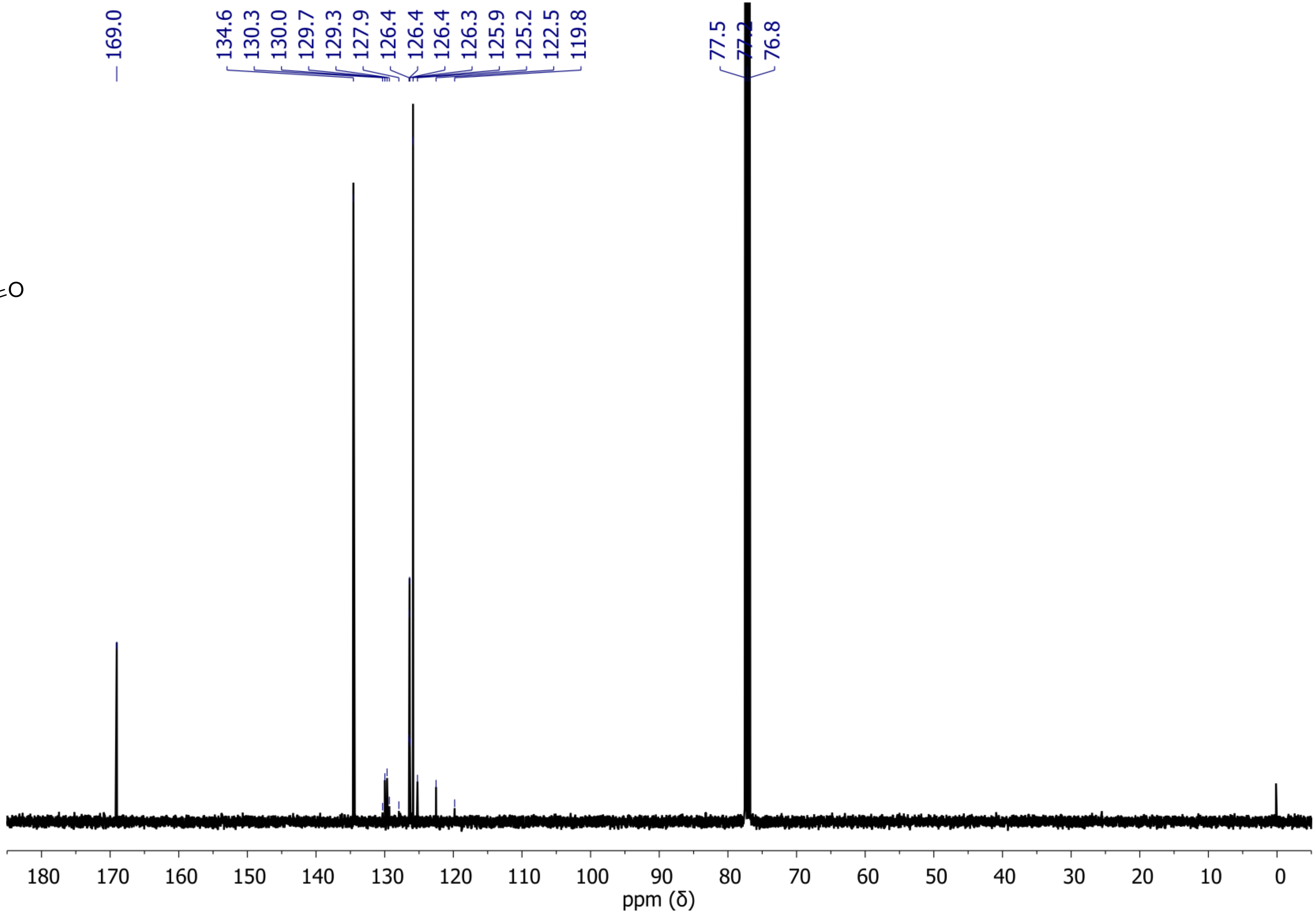

Figure S80. ^{1}H NMR Spectrum (400 MHz, $CDCl_3$) for *N*-(4-iodophenyl)maleimide (**7e**)

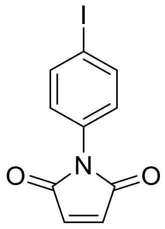

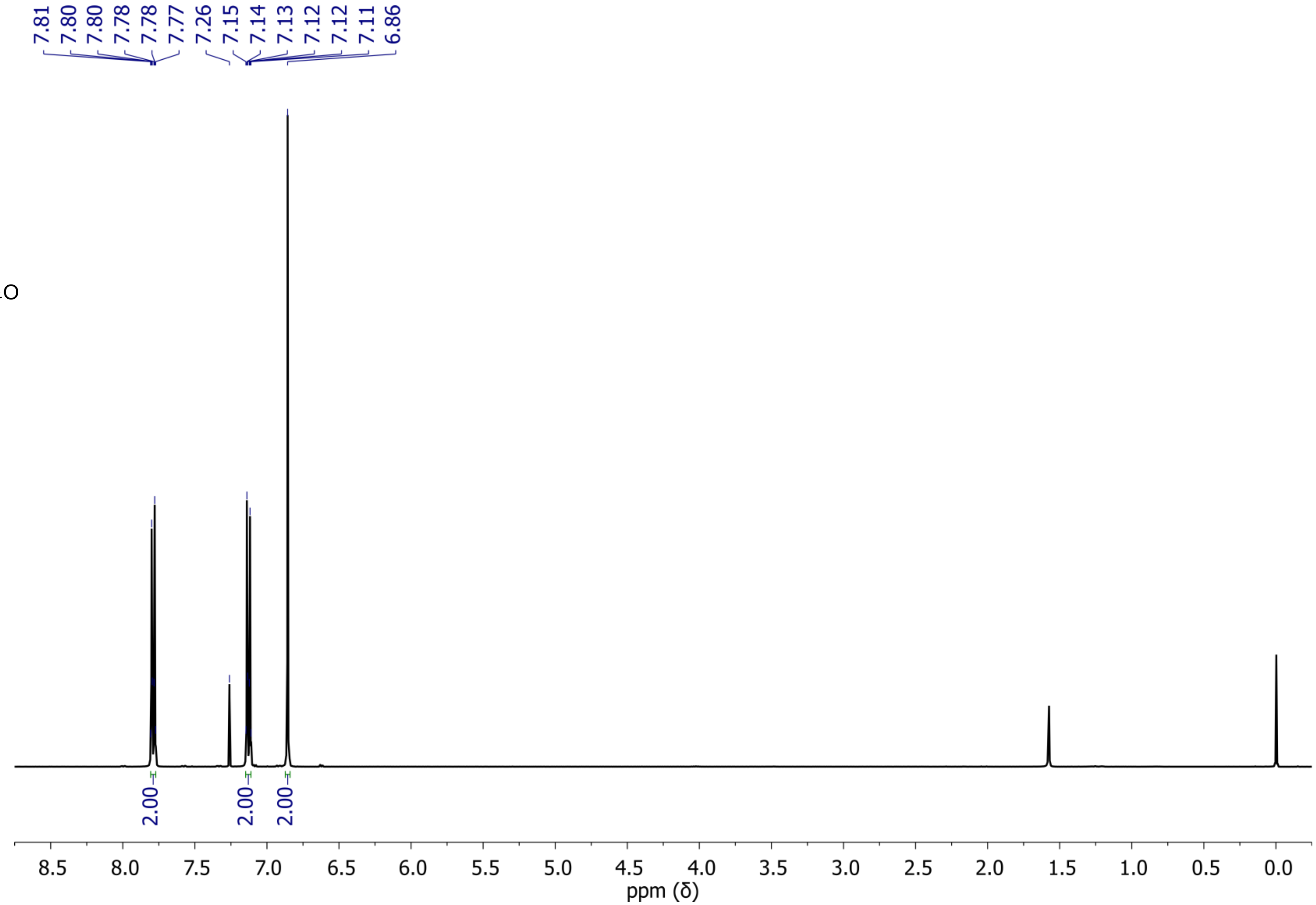

Figure S81. ^{13}C NMR Spectrum (100 MHz, $CDCl_3$) for *N*-(4-iodophenyl)maleimide (**7e**)

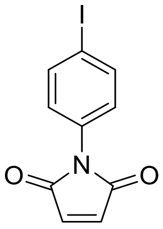

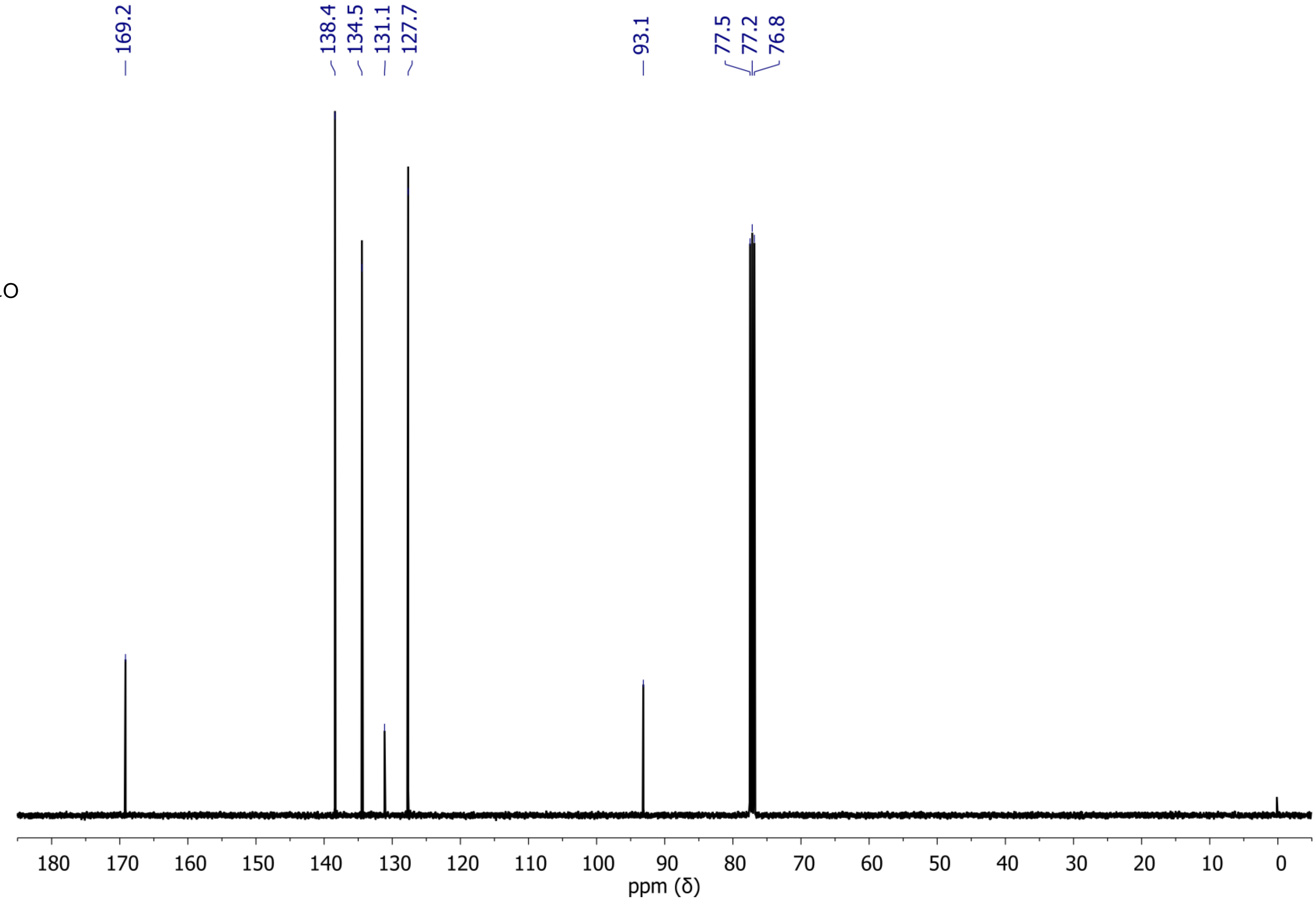

Figure S82. ^{1}H NMR Spectrum (400 MHz, $CDCl_3$) for *N*-(4-bromophenyl)maleimide (**7f**)

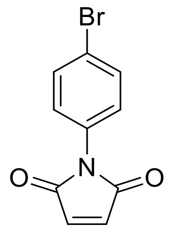

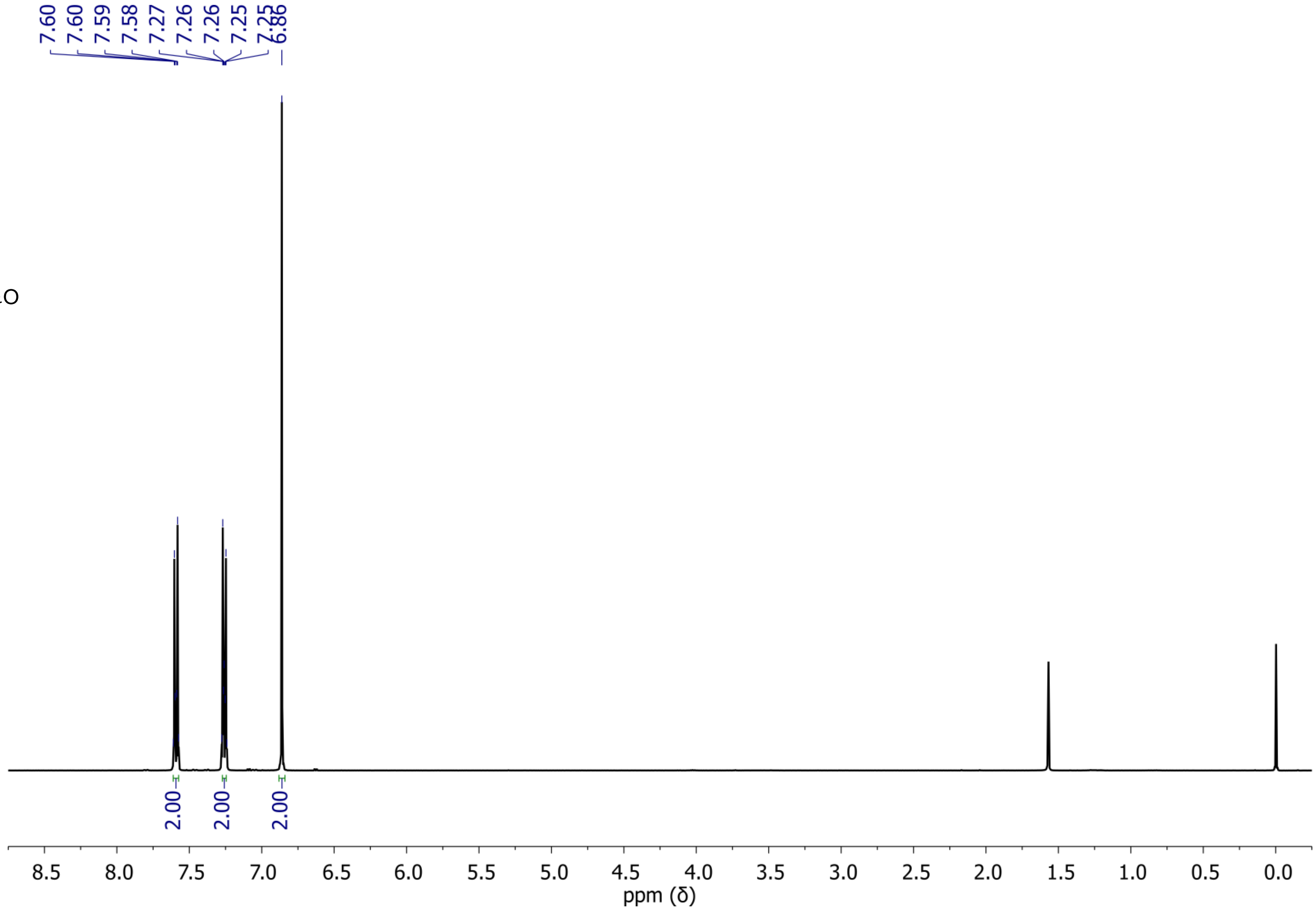

Figure S83. ^{13}C NMR Spectrum (100 MHz, $CDCl_3$) for *N*-(4-bromophenyl)maleimide (**7f**)

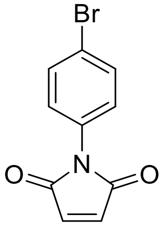

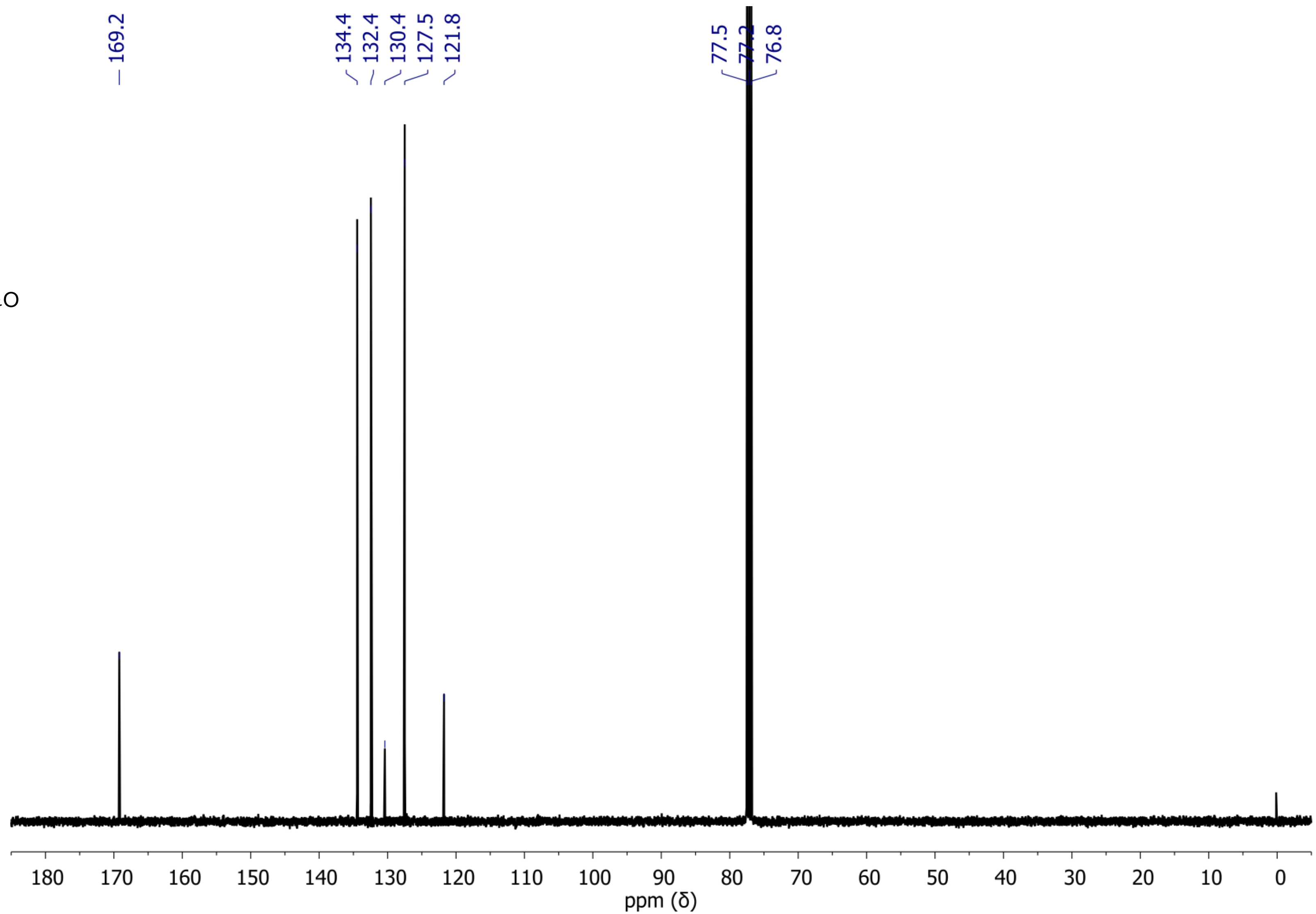

Figure S84. ^{1}H NMR Spectrum (400 MHz, $CDCl_3$) for *N*-(4-chlorophenyl)maleimide (**7g**)

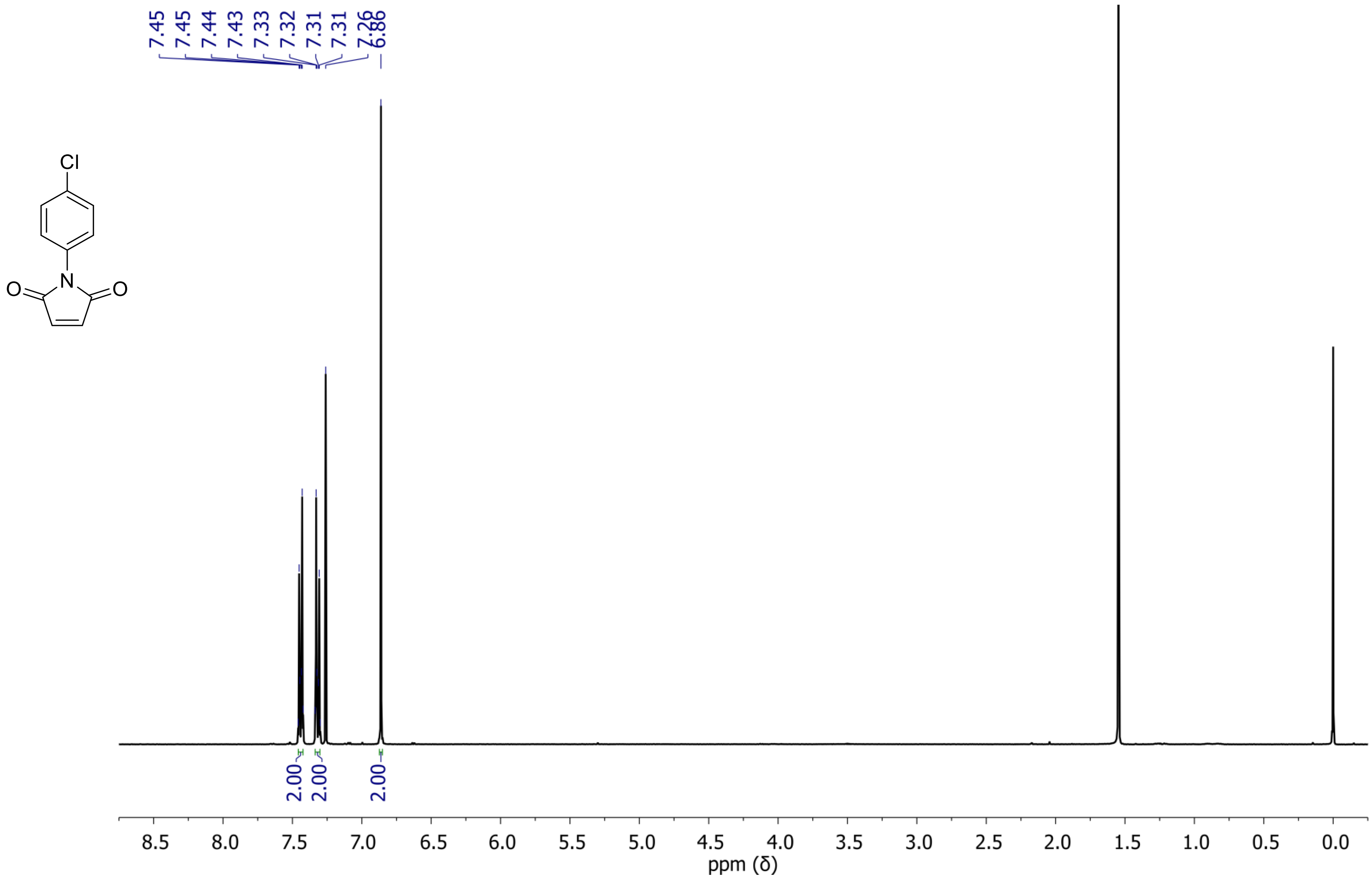

Figure S85. ^{13}C NMR Spectrum (100 MHz, $CDCl_3$) for *N*-(4-chlorophenyl)maleimide (**7g**)

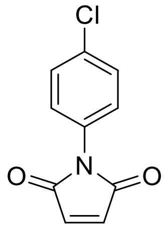

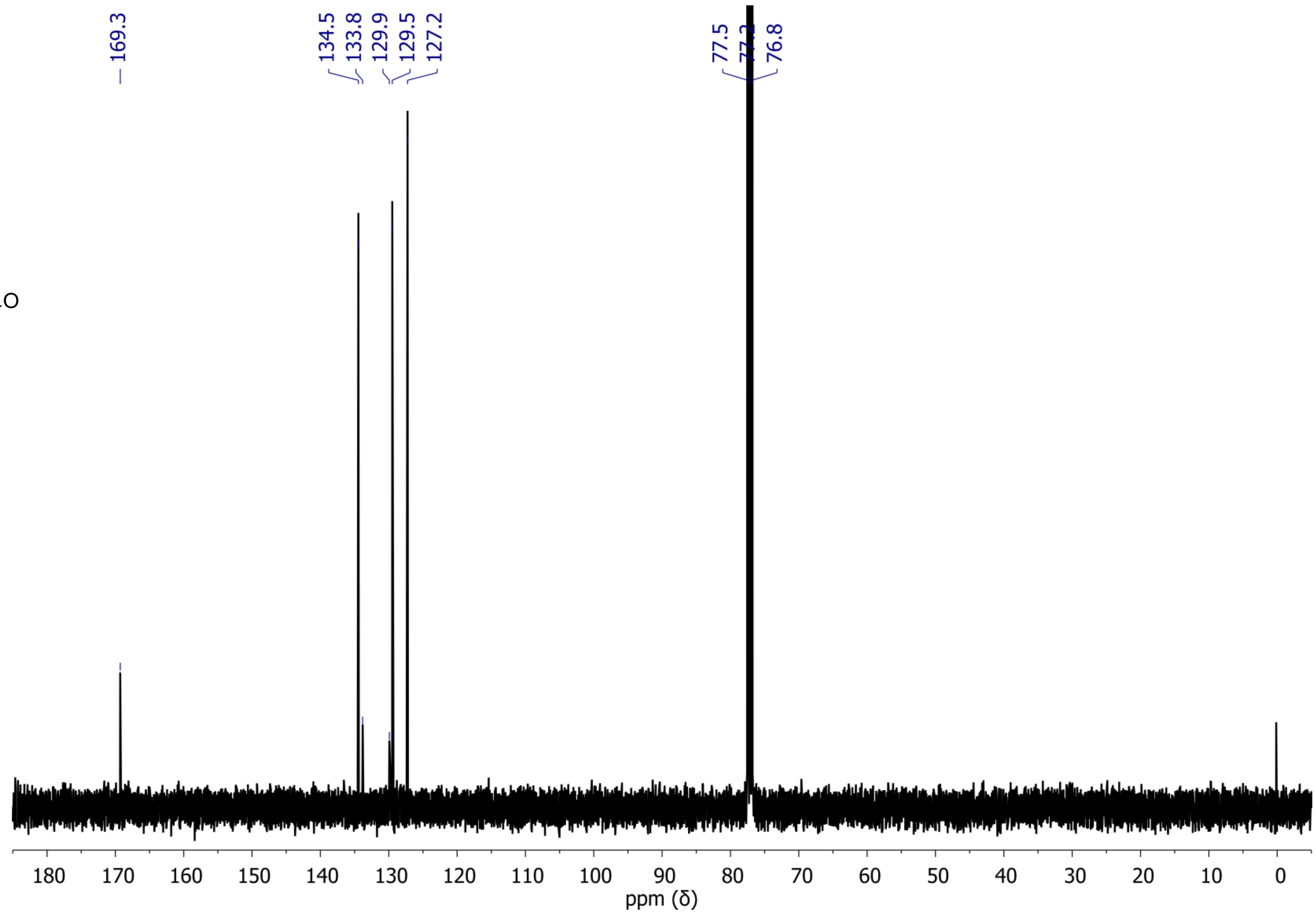

Figure S86. ^{1}H NMR Spectrum (400 MHz, $CDCl_3$) for *N*-(4-fluorophenyl)maleimide (**7h**)

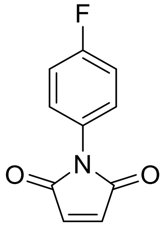

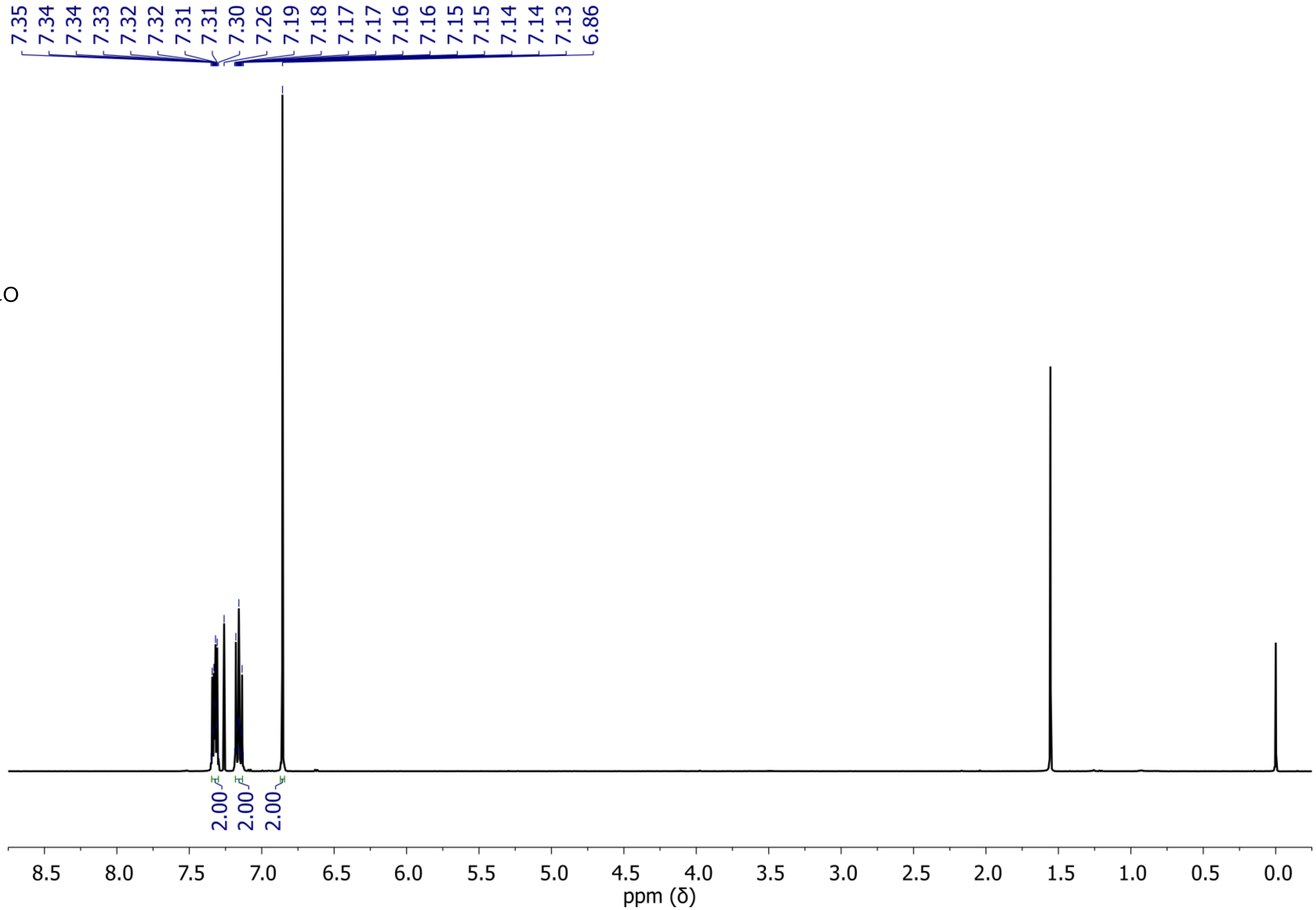

Figure S87. ^{19}F NMR Spectrum (376 MHz, $CDCl_3$) for *N*-(4-fluorophenyl)maleimide (**7h**)

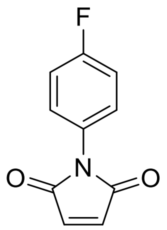

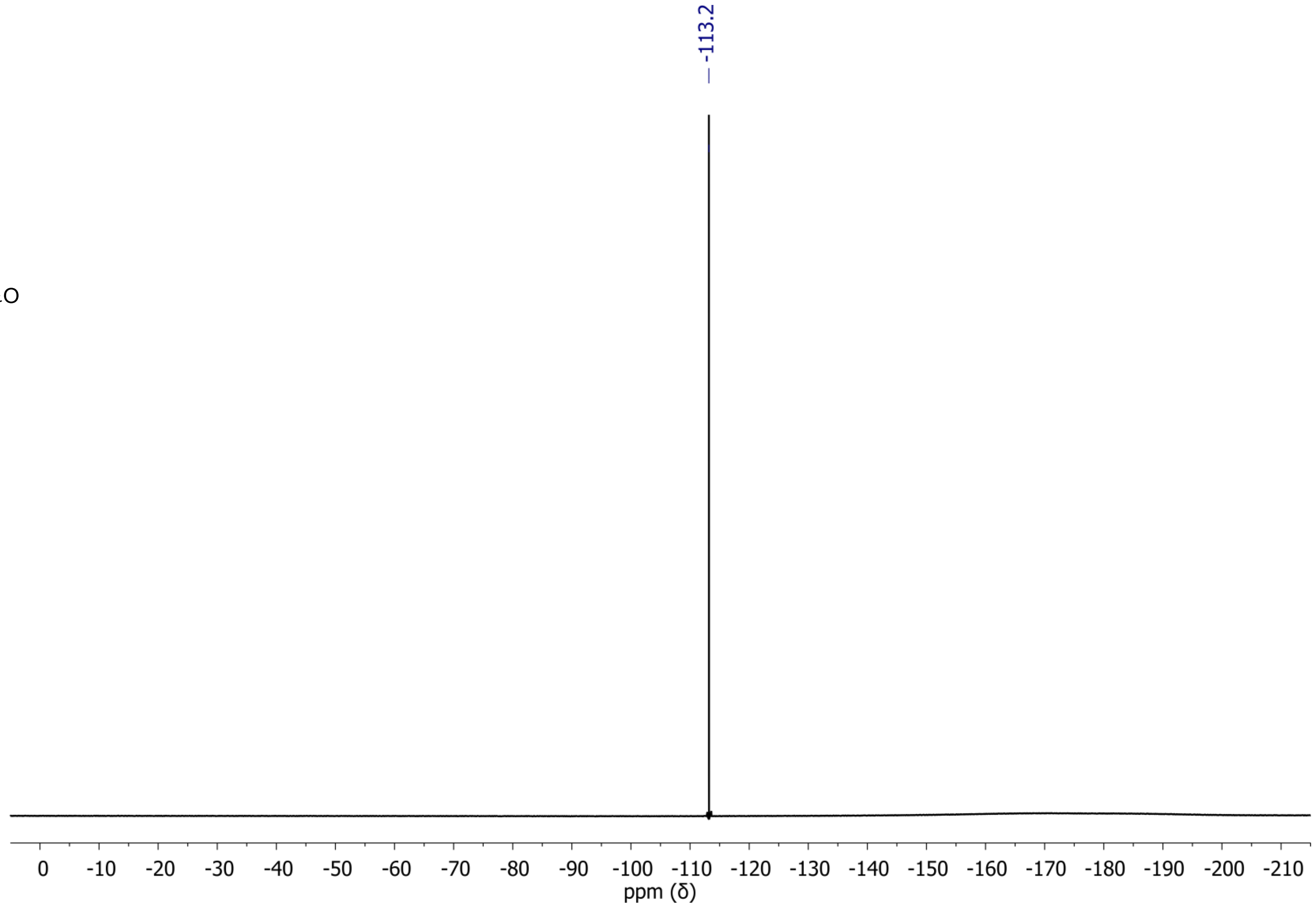

Figure S88. ^{13}C NMR Spectrum (100 MHz, $CDCl_3$) for *N*-(4-fluorophenyl)maleimide (**7h**)

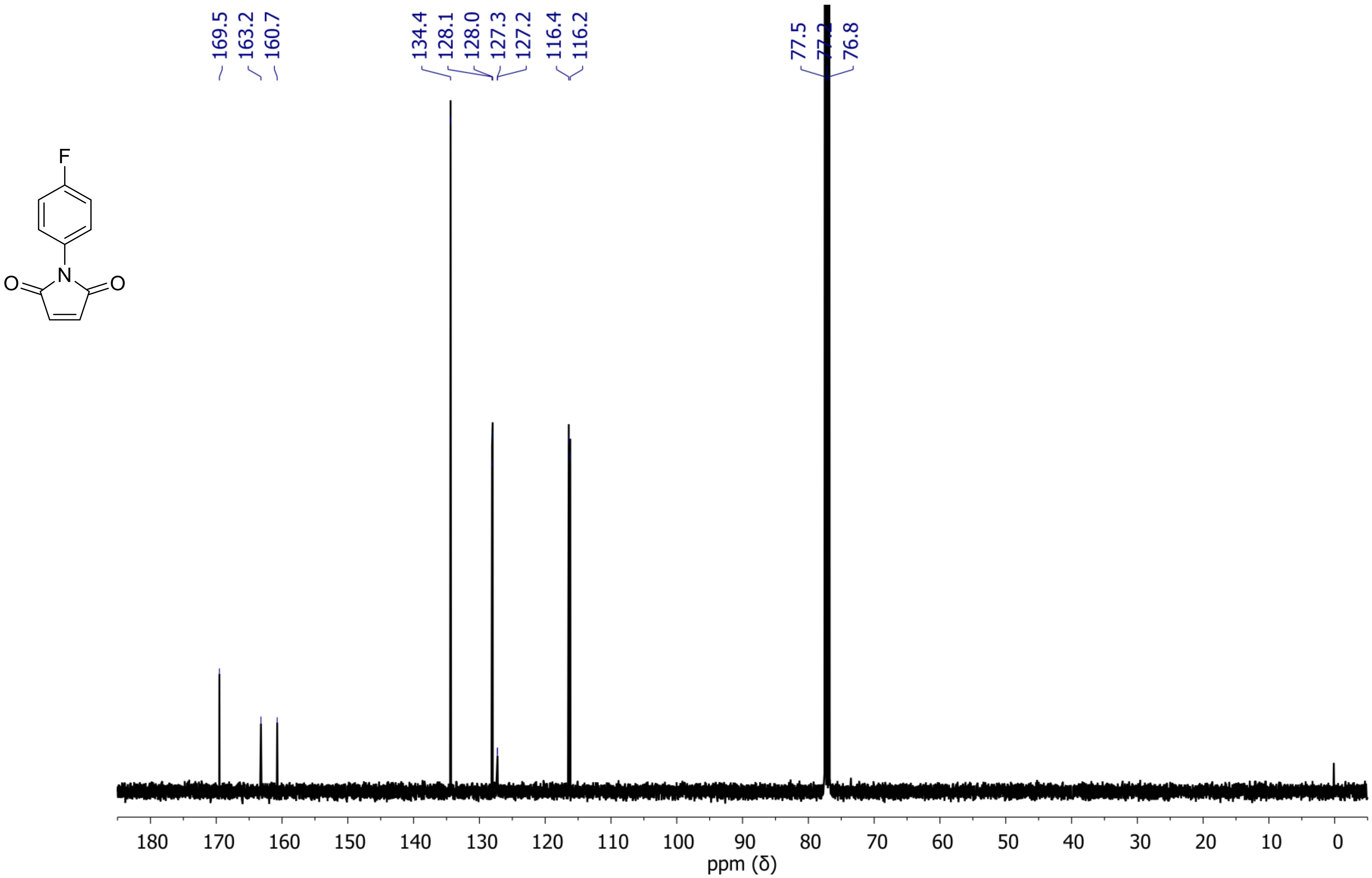

Figure S89. ^{1}H NMR Spectrum (400 MHz, $CDCl_3$) for *N*-phenylmaleimide (**7i**)

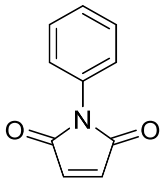

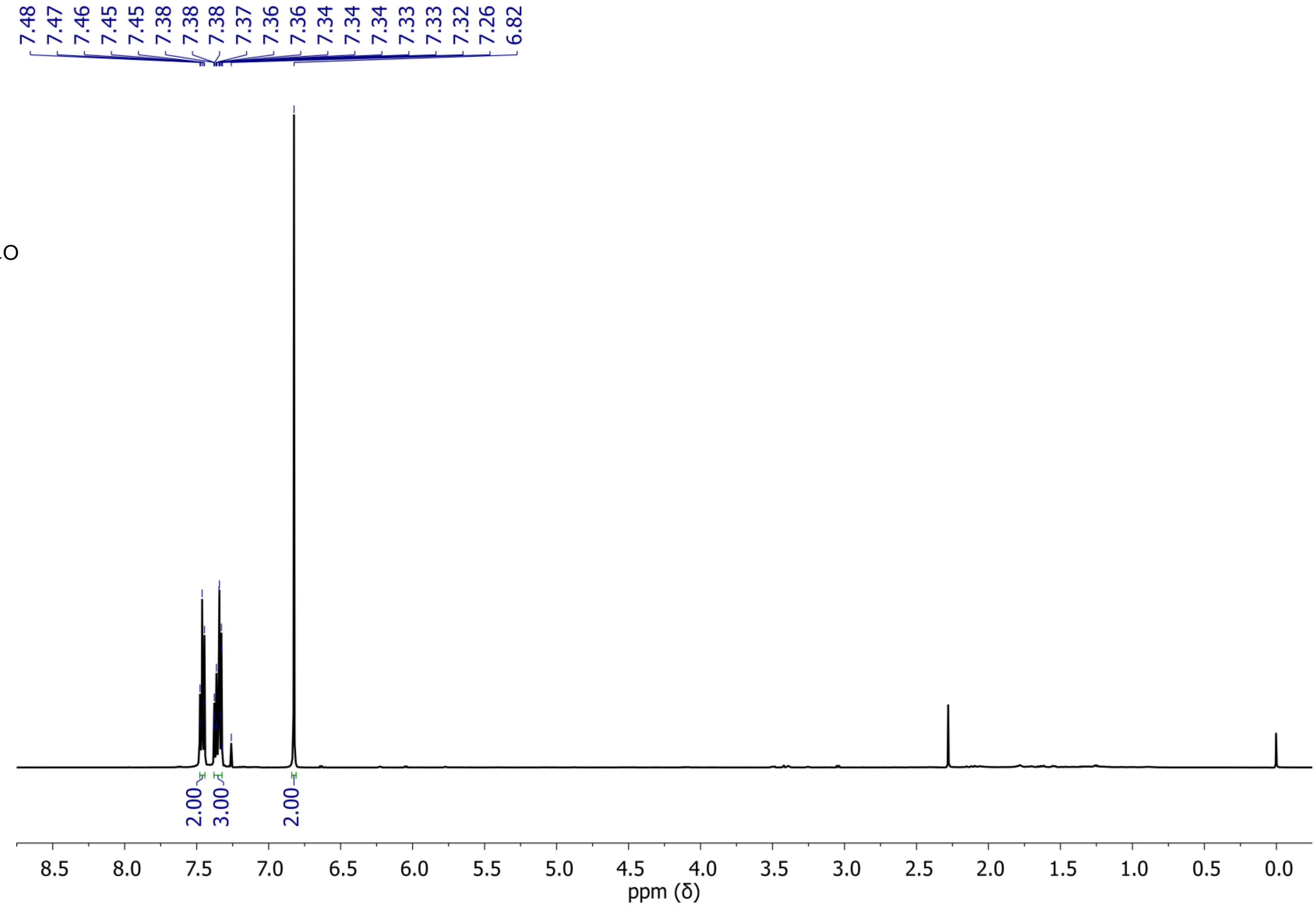

Figure S90. ^{13}C NMR Spectrum (100 MHz, $CDCl_3$) for *N*-phenylmaleimide (**7i**)

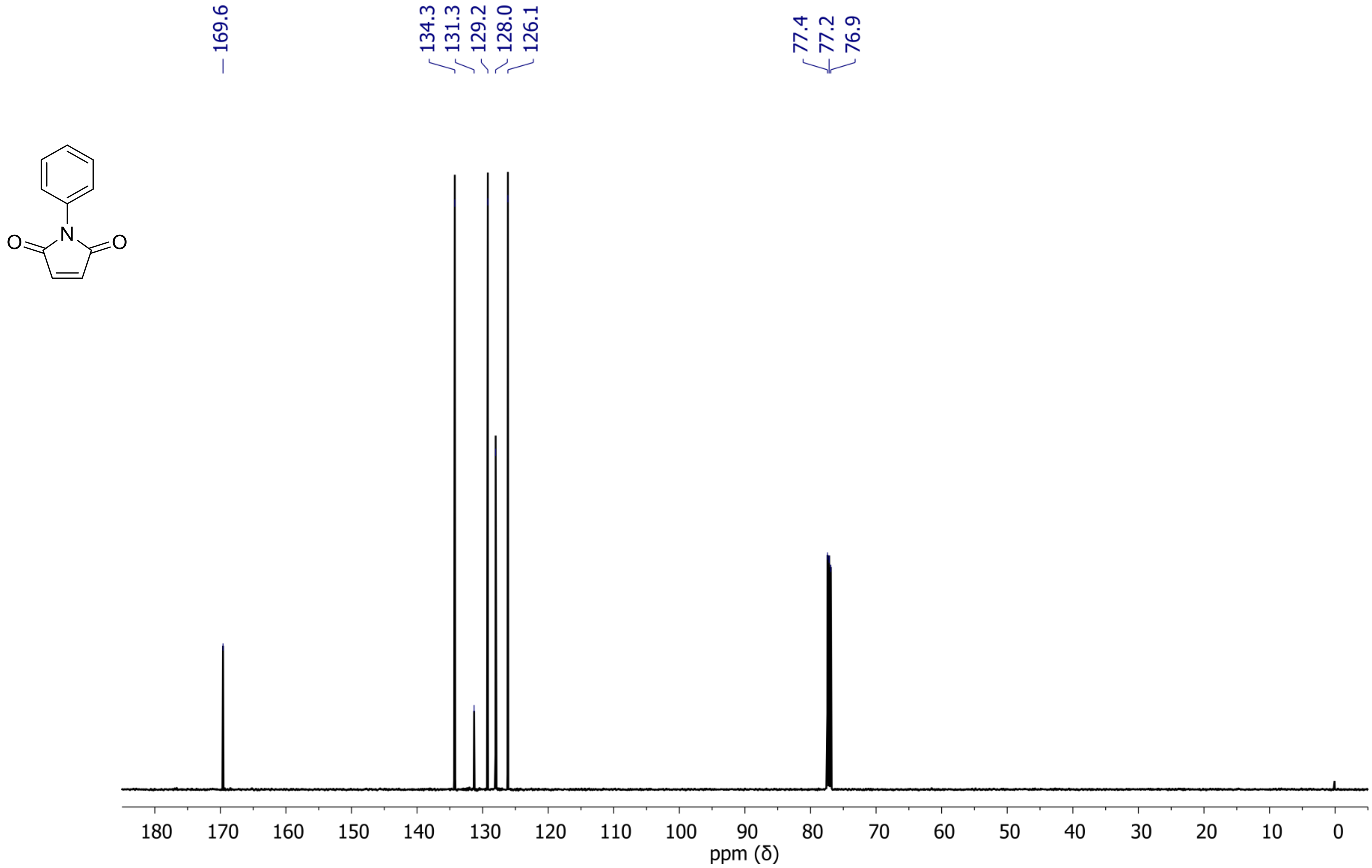

Figure S91. ^{1}H NMR Spectrum (400 MHz, $CDCl_3$) for *N*-(4-methylphenyl)maleimide (**7j**)

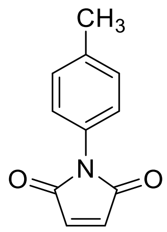

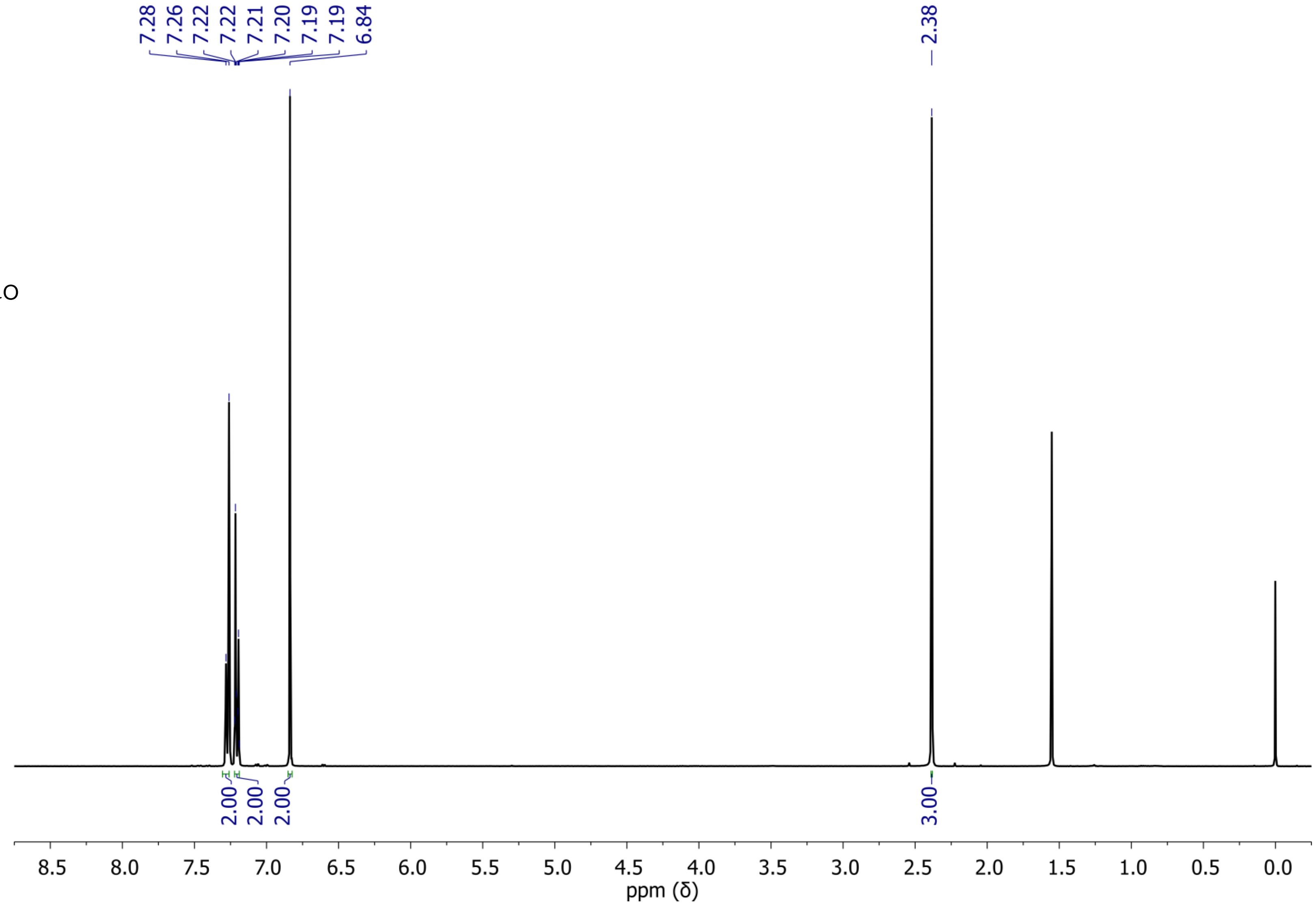

Figure S92. ^{13}C NMR Spectrum (100 MHz, $CDCl_3$) for *N*-(4-methylphenyl)maleimide (**7j**)

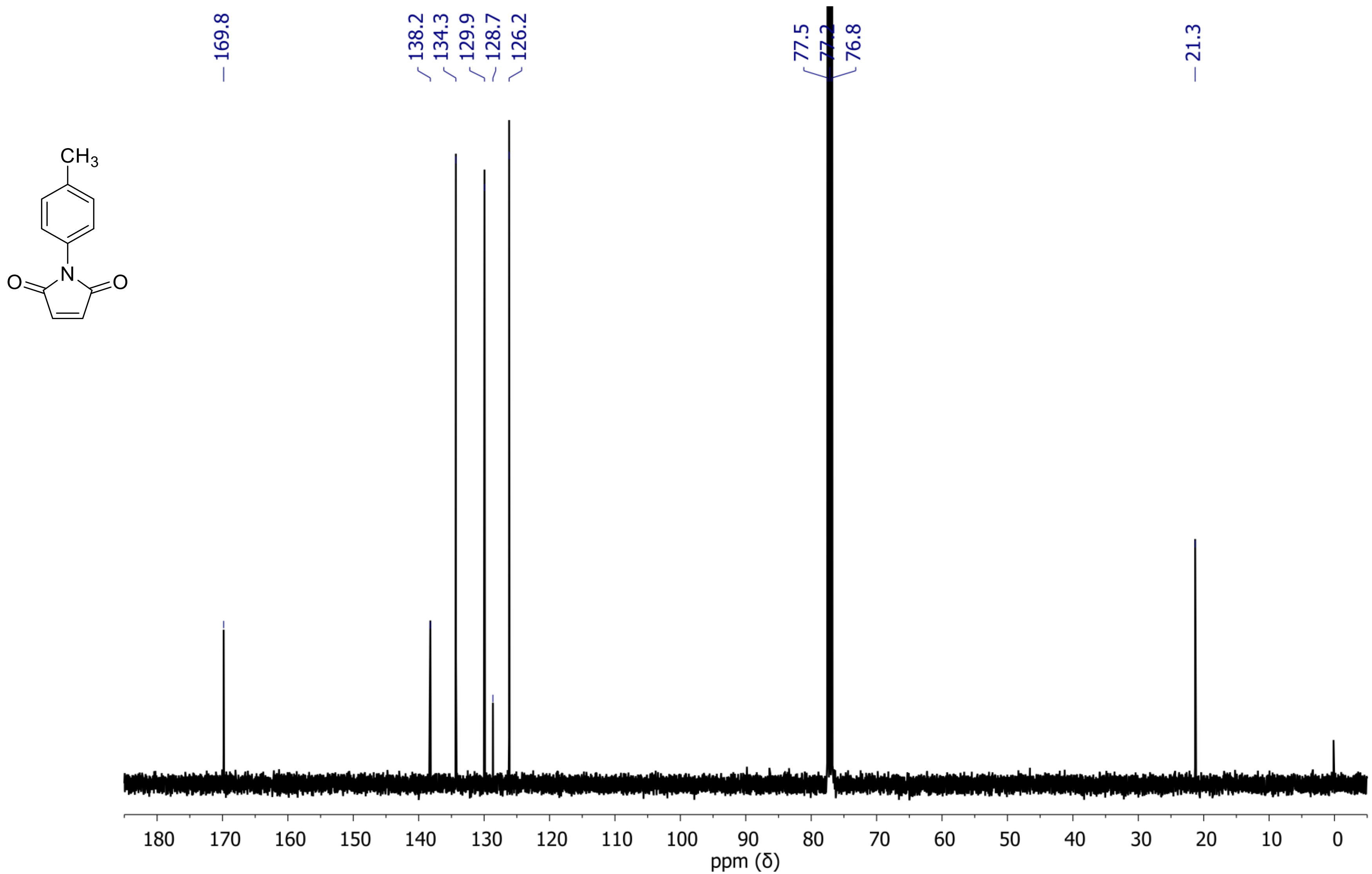

Figure S93. ^{1}H NMR Spectrum (400 MHz, $CDCl_3$) for *N*-(4-methoxyphenyl)maleimide (**7k**)

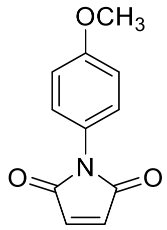

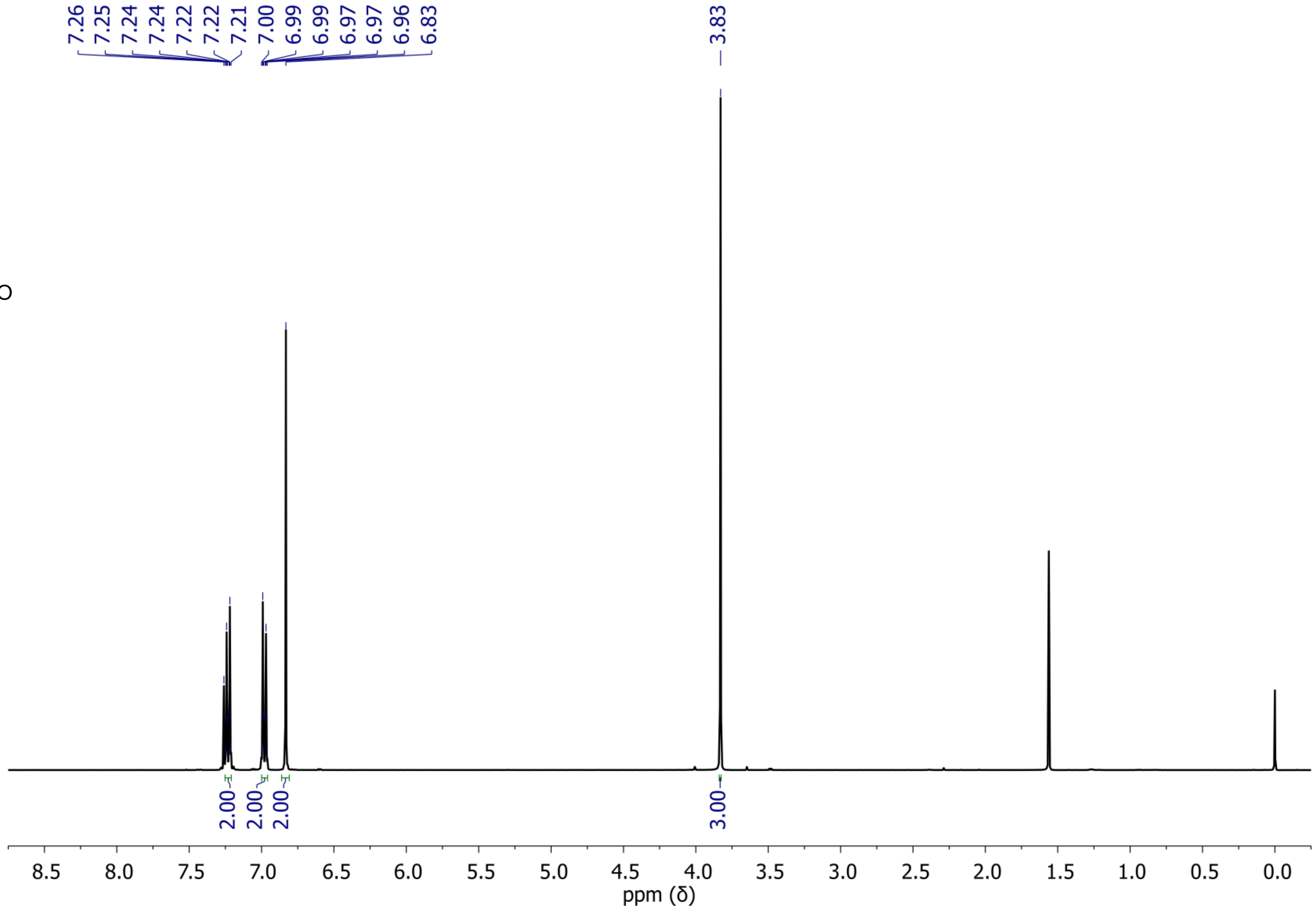

Figure S94. ^{13}C NMR Spectrum (100 MHz, $CDCl_3$) for *N*-(4-methoxyphenyl)maleimide (**7k**)

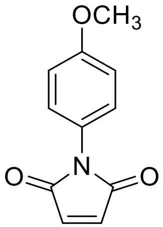

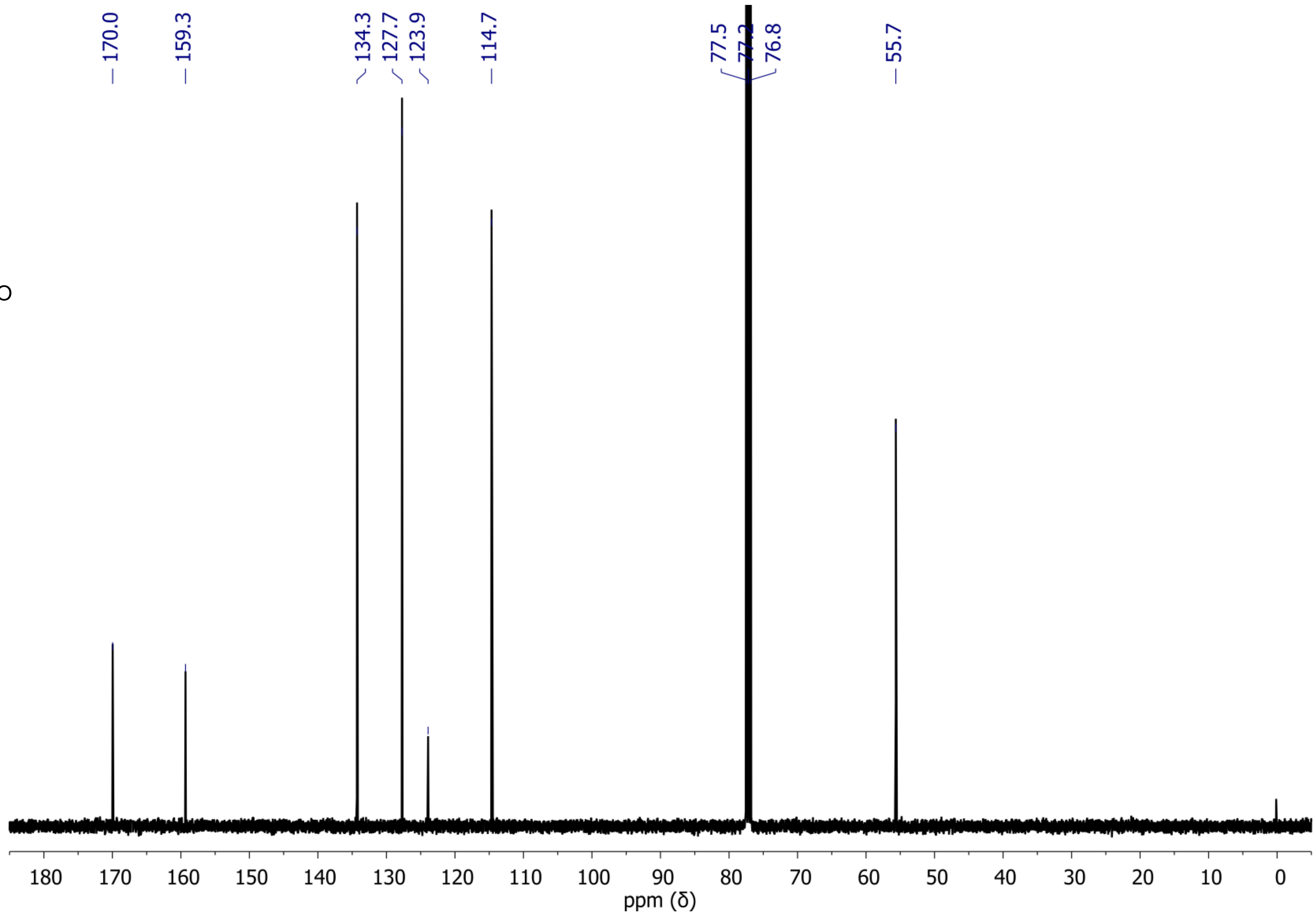

Figure S95. ^{1}H NMR Spectrum (400 MHz, $CDCl_3$) for *N*-(4-(dimethylamino)phenyl)maleimide (**7l**)

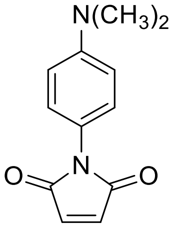

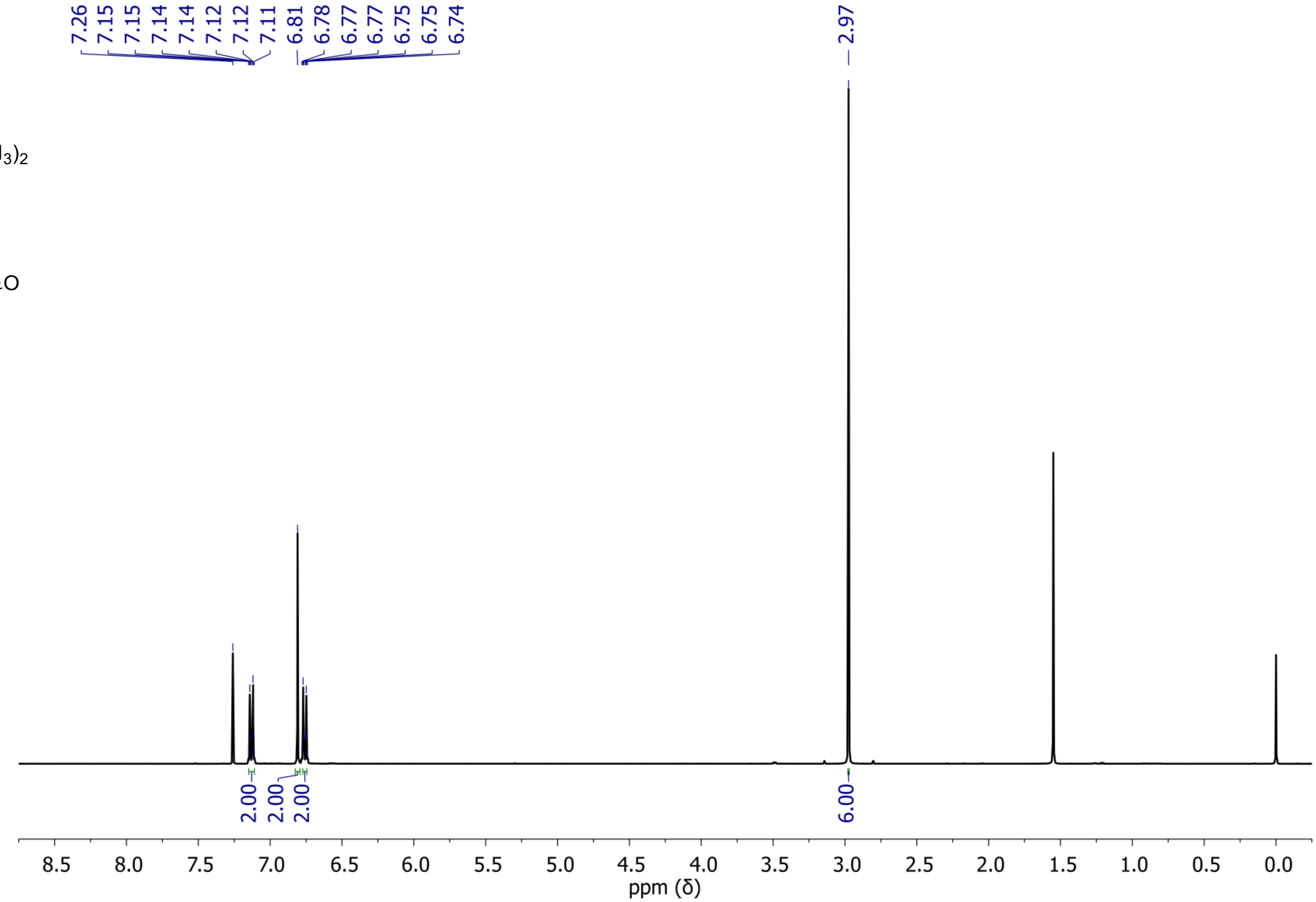

Figure S96. ^{13}C NMR Spectrum (100 MHz, $CDCl_3$) for *N*-(4-(dimethylamino)phenyl)maleimide (**7l**)

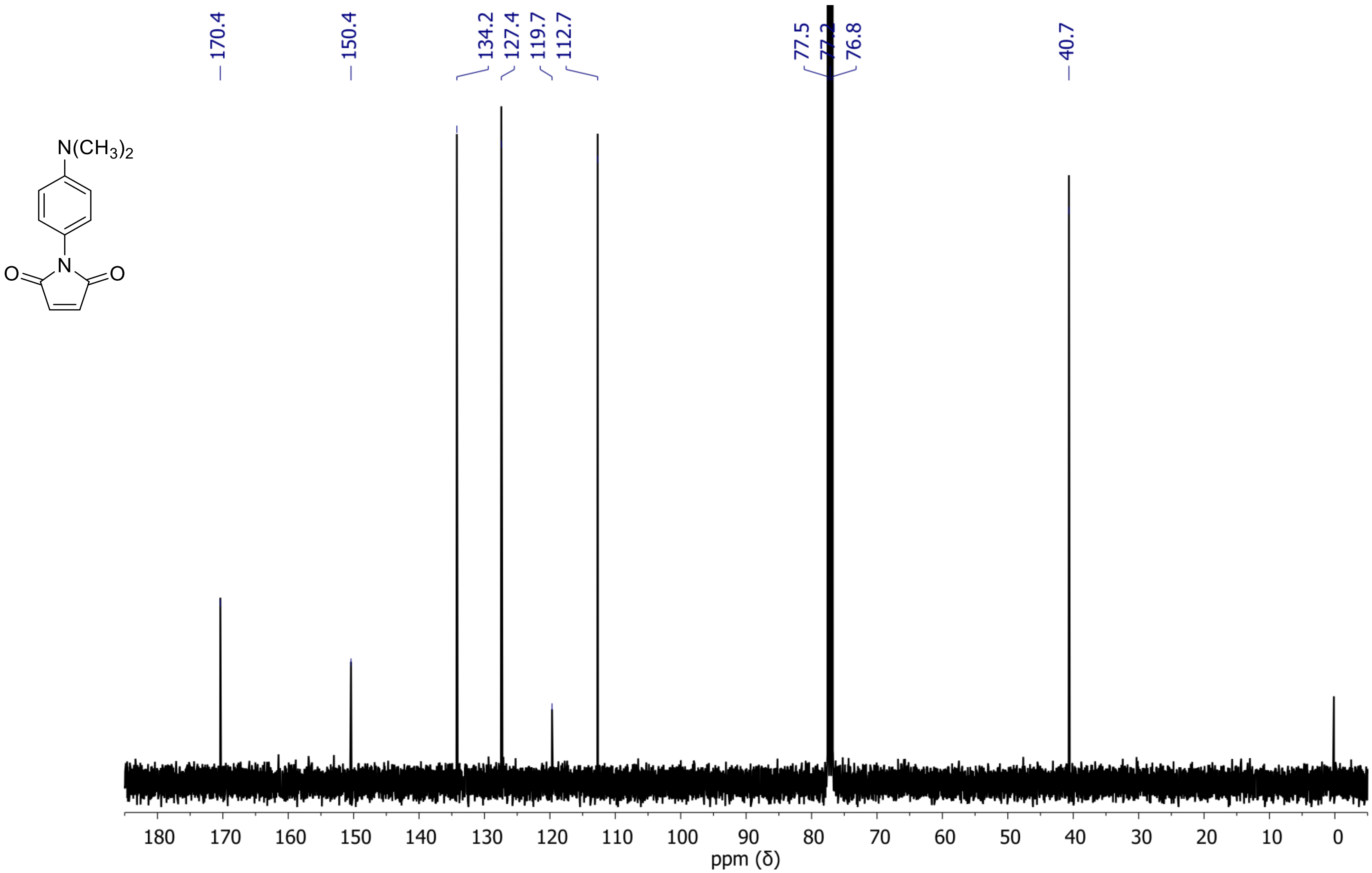

Figure S97. ^{1}H NMR Spectrum (400 MHz, $CDCl_3$) for *N*-(naphthalene-1-yl)maleimide (**7m**)

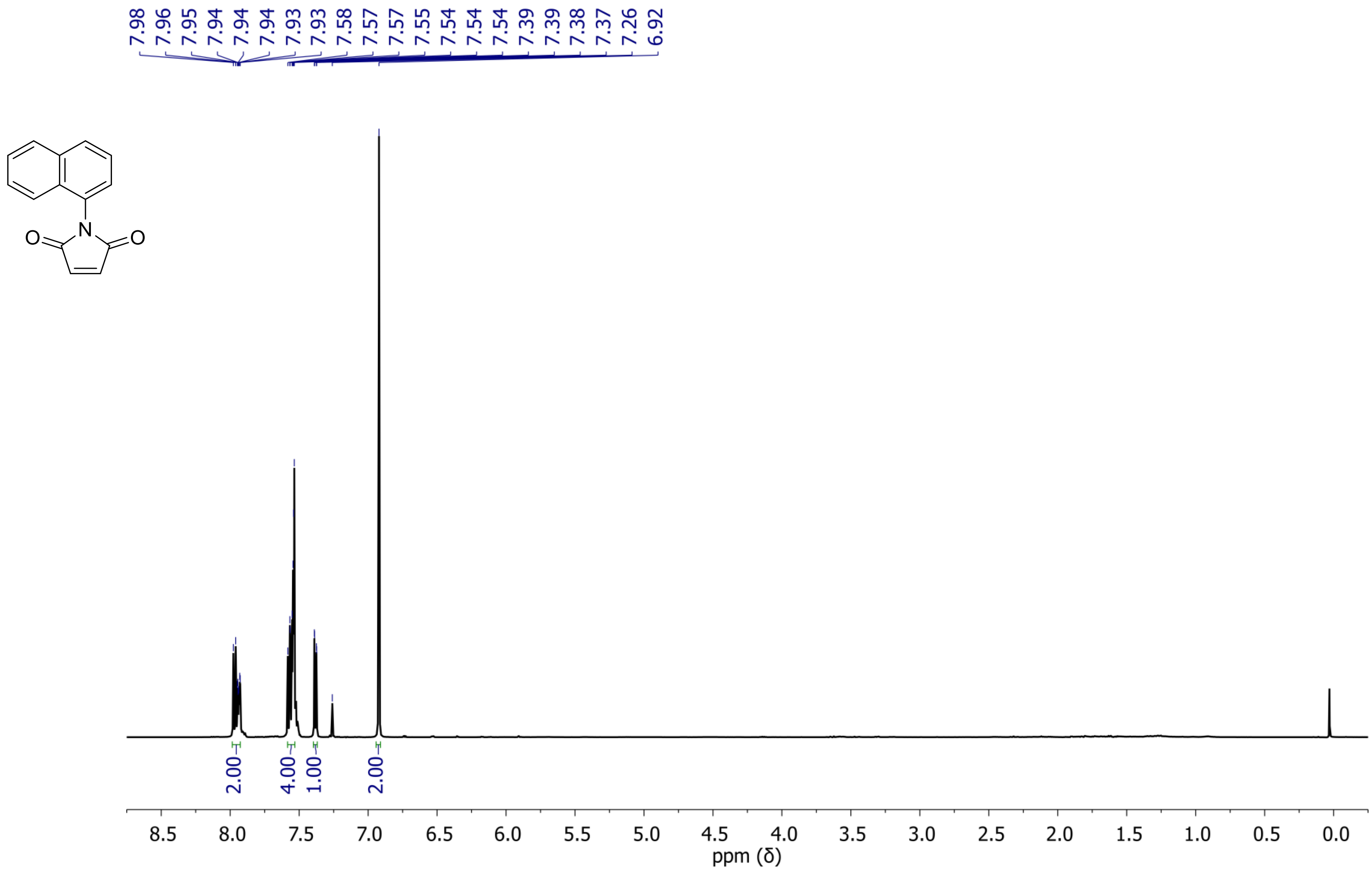

Figure S98. ^{13}C NMR Spectrum (100 MHz, $CDCl_3$) for *N*-(naphthalene-1-yl)maleimide (**7m**)

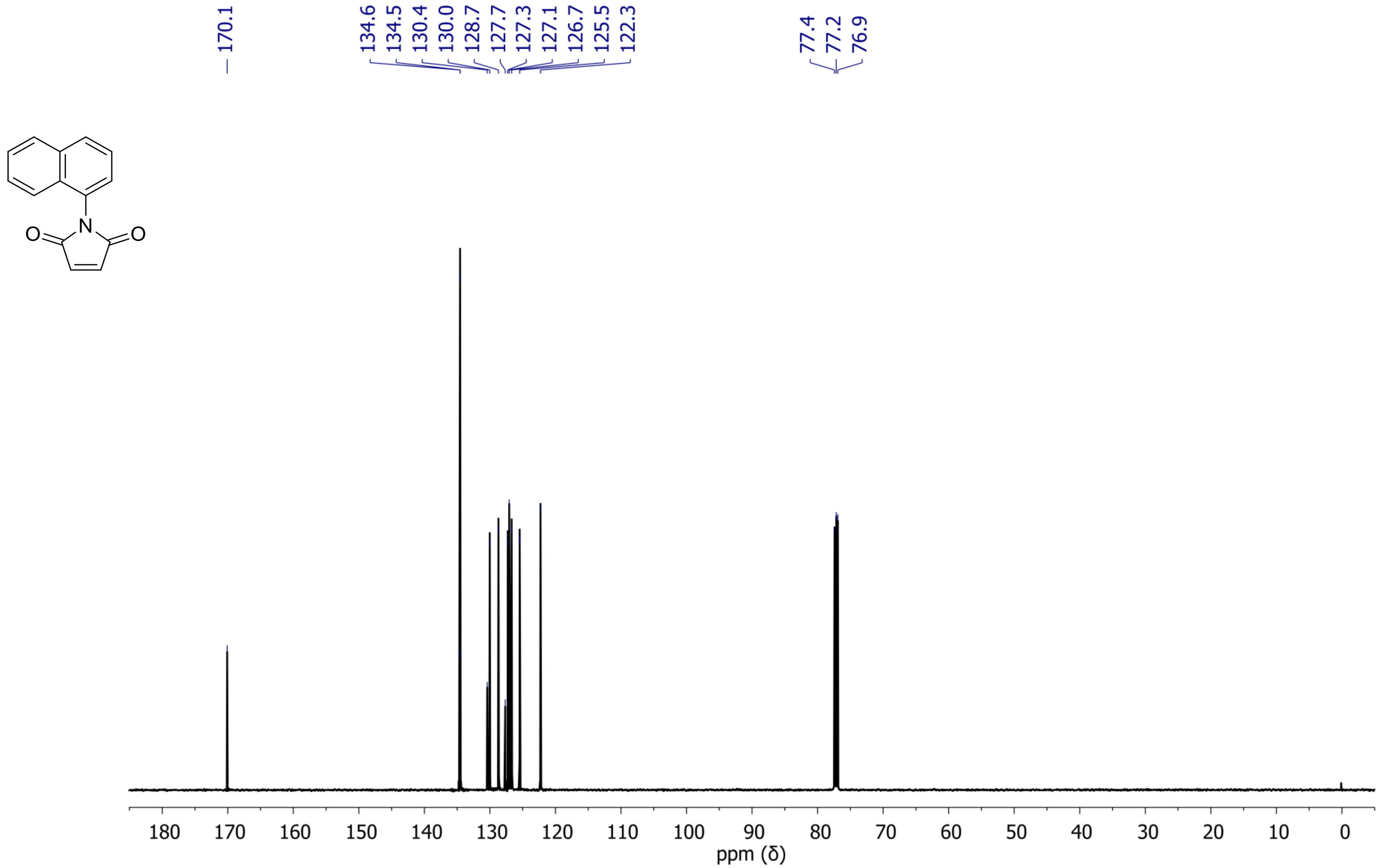

Figure S99. ^{1}H NMR Spectrum (400 MHz, $CDCl_3$) for *N*-benzylmaleimide (**7n**)

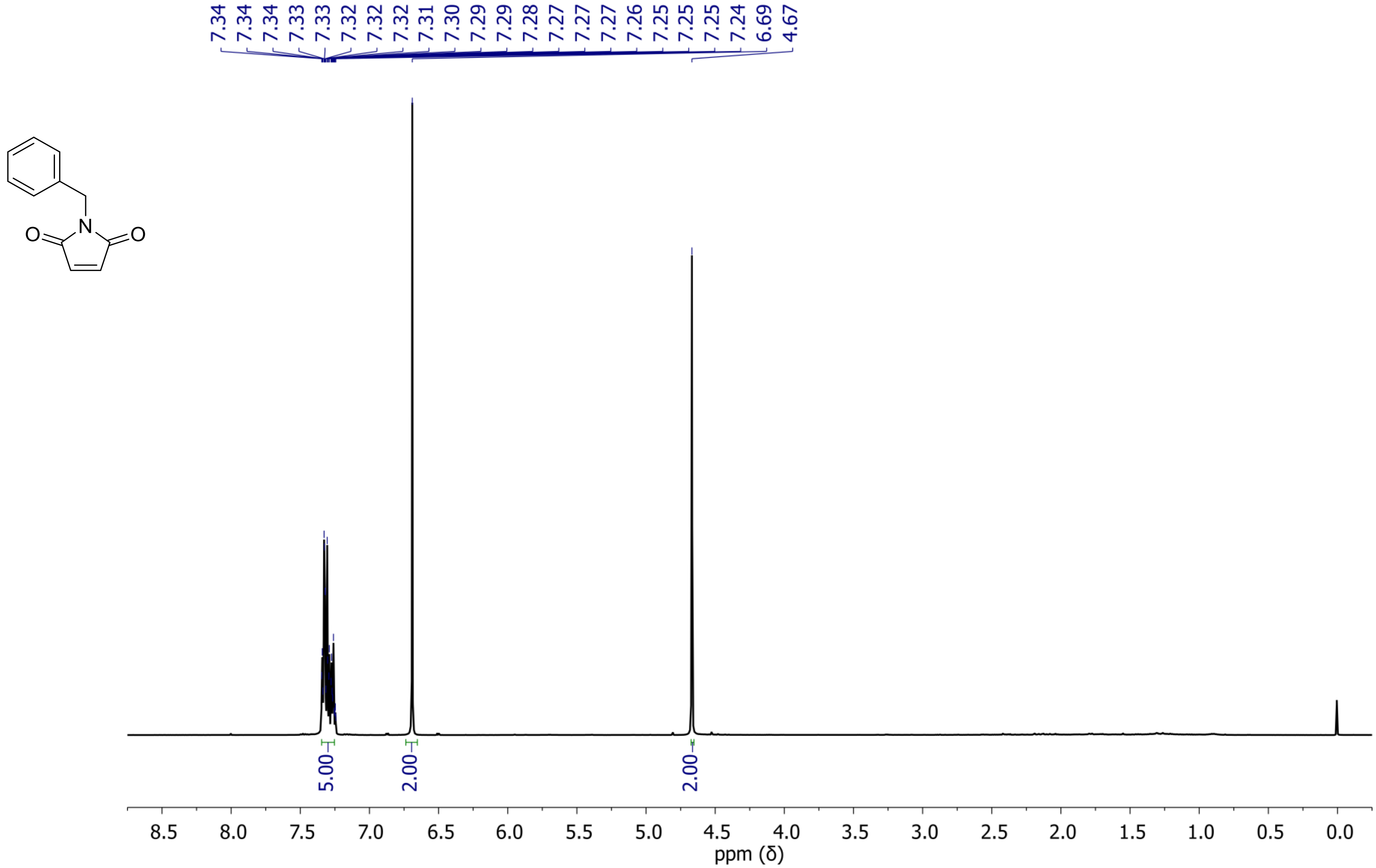

Figure S100. ^{13}C NMR Spectrum (100 MHz, $CDCl_3$) for *N*-benzylmaleimide (**7n**)

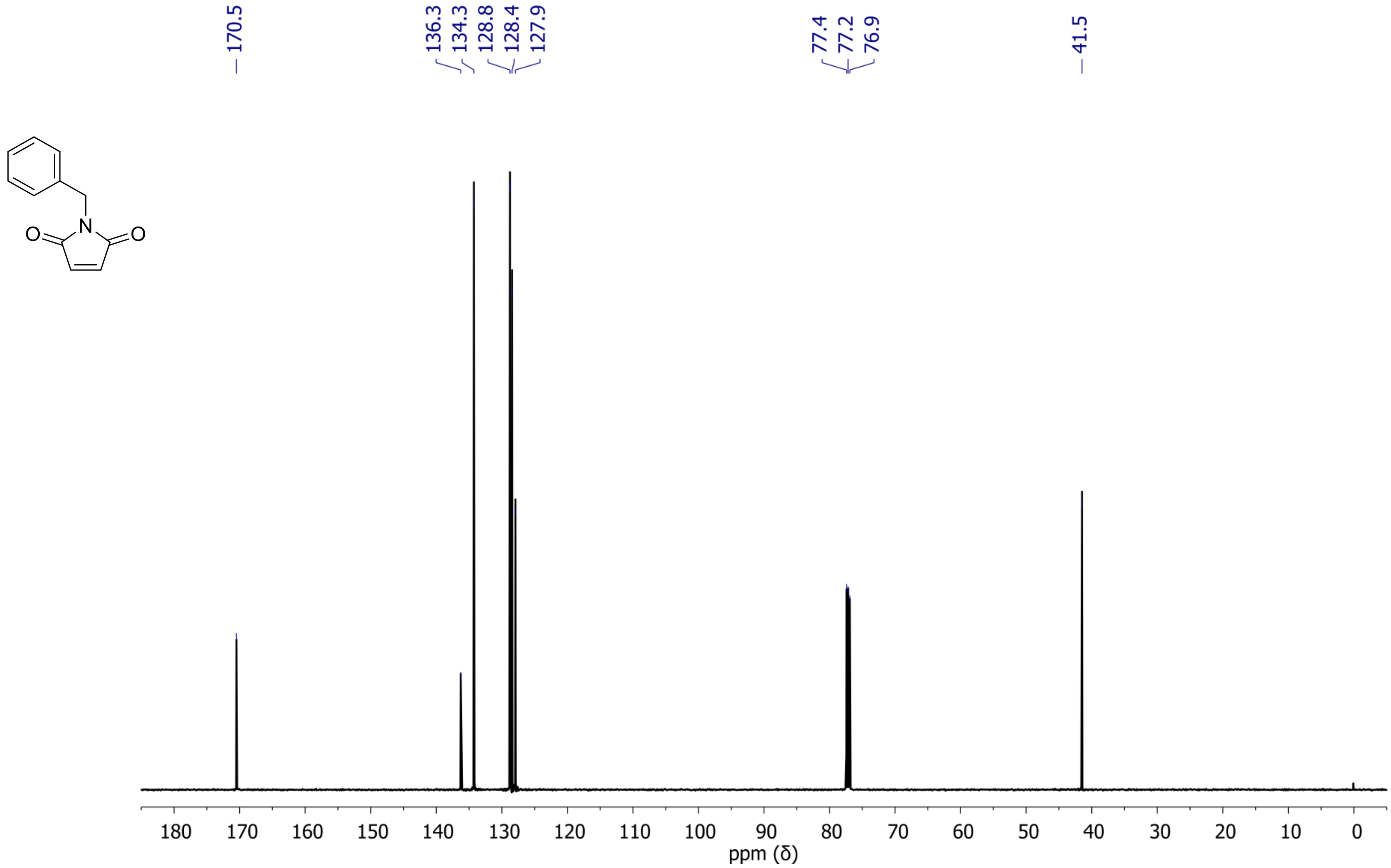

Figure S101. ^{1}H NMR Spectrum (400 MHz, $CDCl_3$) for *N*-(2-(1*H*-indol-3-yl)ethyl)maleimide (**7o**)

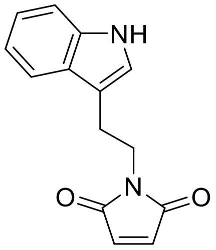

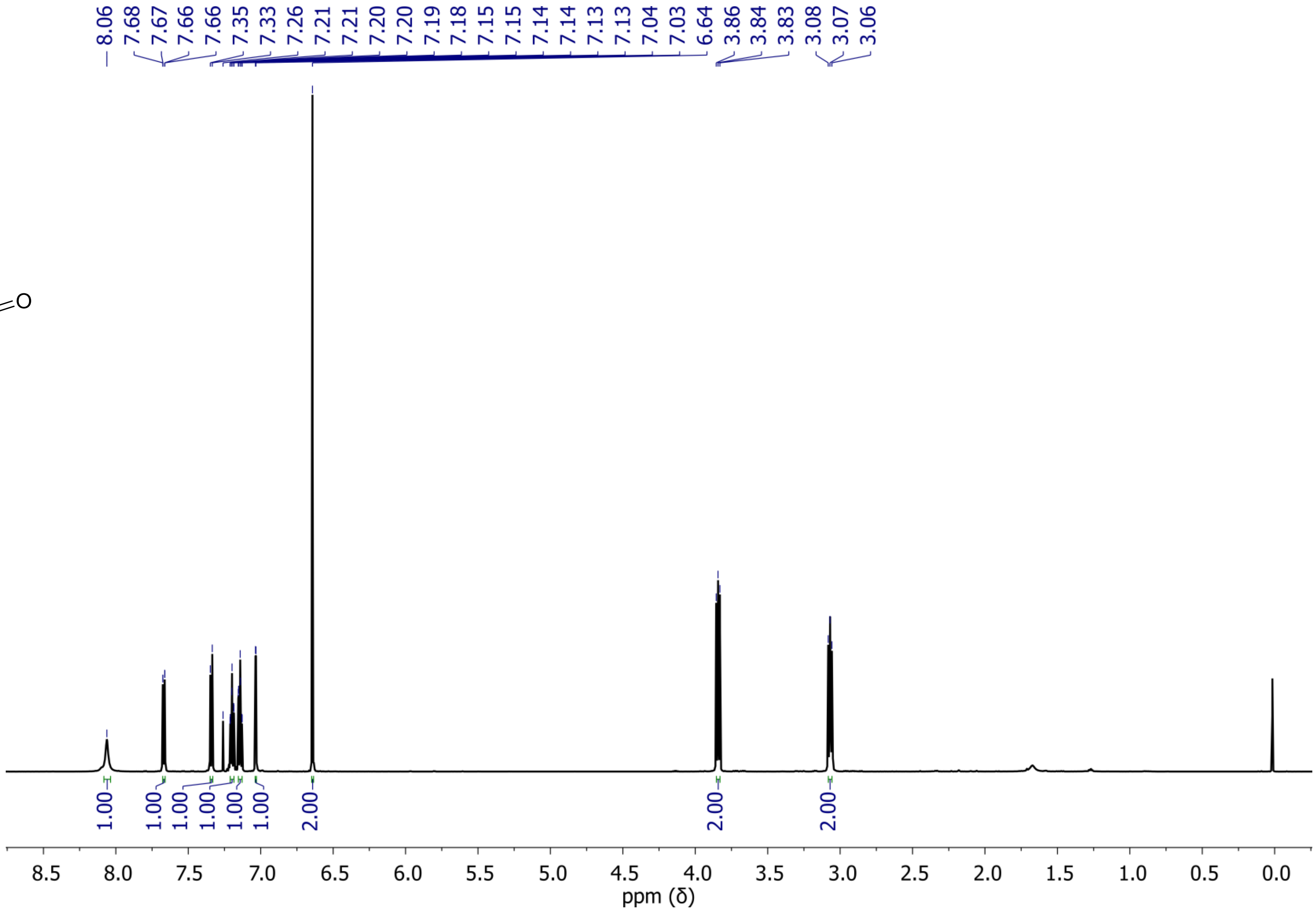

Figure S102. ^{13}C NMR Spectrum (100 MHz, $CDCl_3$) for *N*-(2-(1*H*-indol-3-yl)ethyl)maleimide (**7o**)

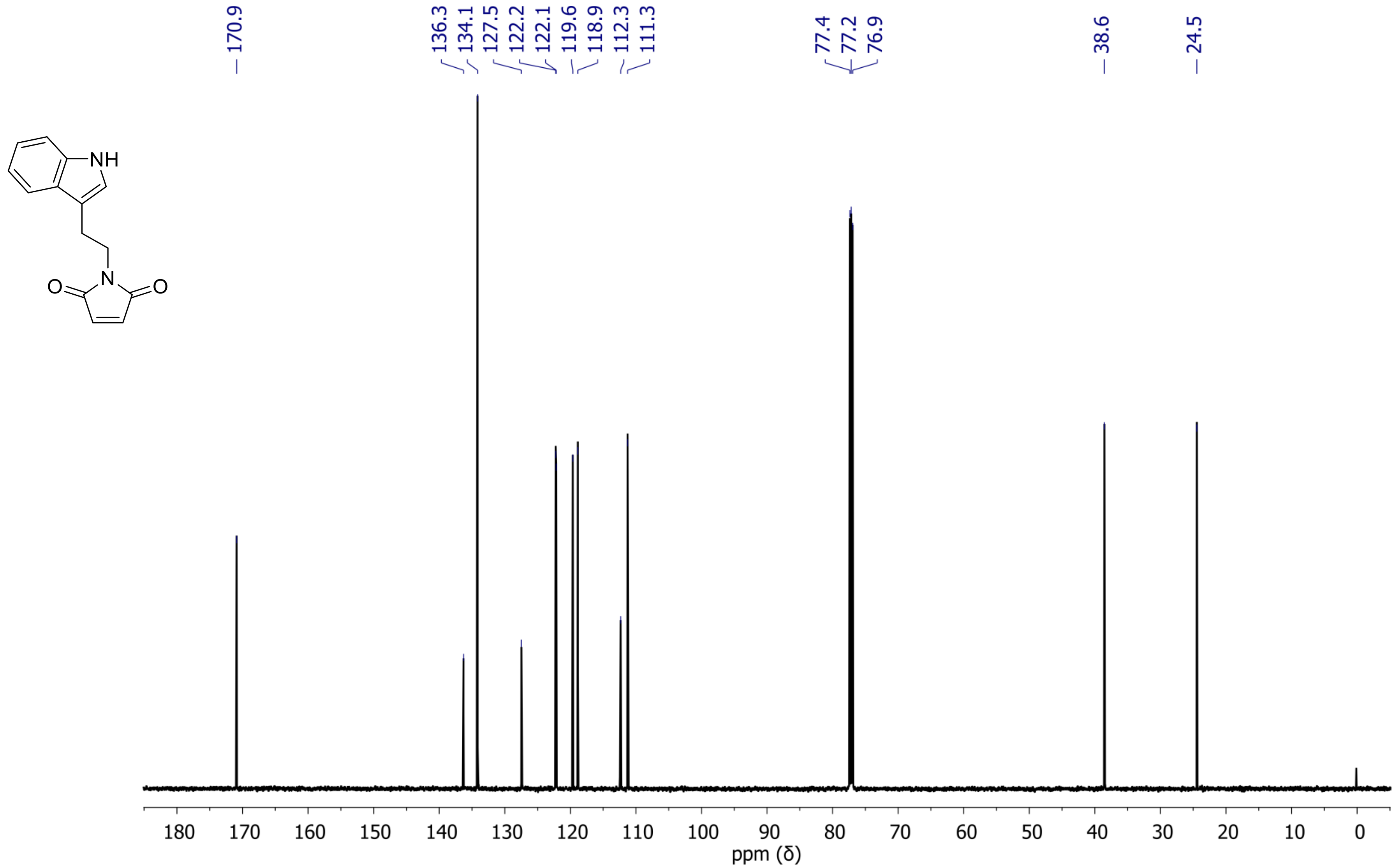

Figure S103. ^{1}H NMR Spectrum (400 MHz, $CDCl_3$) for *N*-(1-adamantyl)maleimide (**7p**)

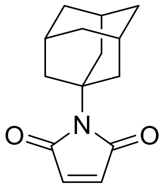

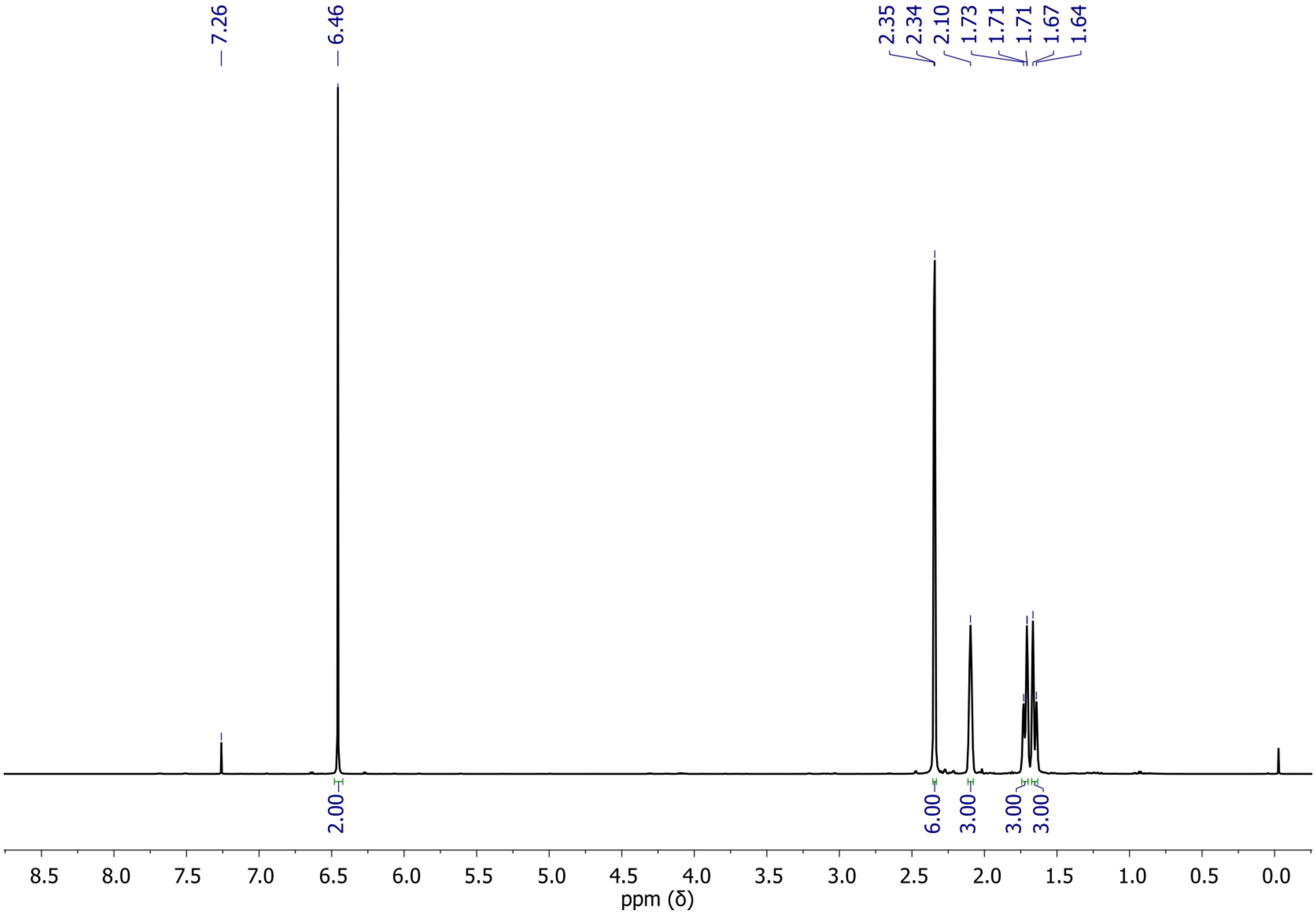

Figure S104. ^{13}C NMR Spectrum (100 MHz, $CDCl_3$) for *N*-(1-adamantyl)maleimide (**7p**)

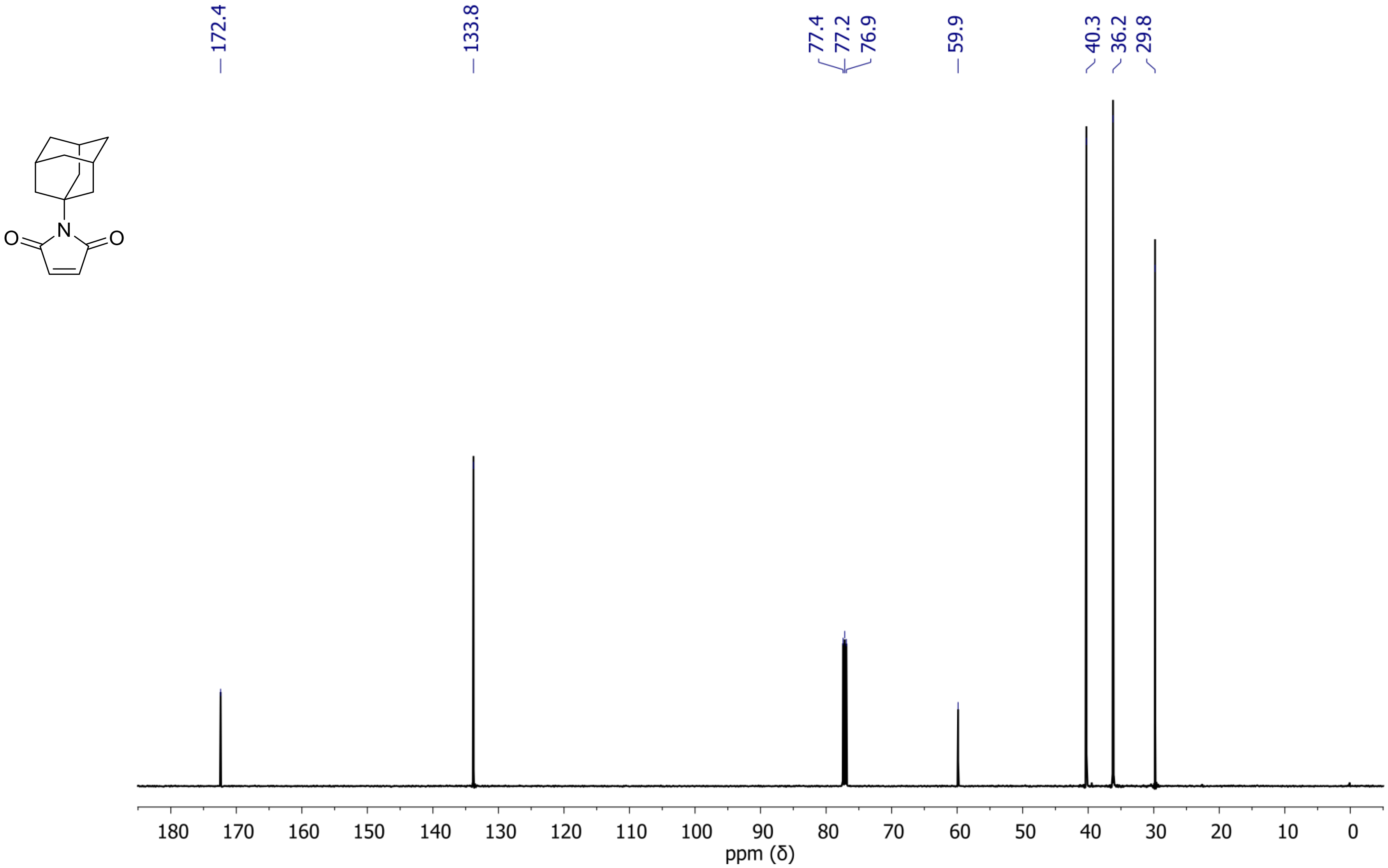

References

[1] R. R. A. Bolt, S. E. Raby-Buck, K. Ingram, J. A. Leitch, D. L. Browne, *Angew. Chem. Int. Ed.* **2022**, *61*, e202210508.

[2] K. W. Omari, L. Dodot, F. M. Kerton, *ChemSusChem* **2012**, *5*, 1767.

[3] F.A. Kucherov, K. I. Galkin, E. G. Gordeev, V. P. Ananikov, *Green Chem.*, **2017**, *19*, 4858.

[4] H. Chang, G. W. Huber, J. A. Dumesic, *ChemSusChem.*, **2020**, *13*, 5213.

[5] R. C. Cioc, T. J. Smak, M. Crockatt, J. C. van der Waal, P. C. A. Bruijnincx, *Green Chem.*, **2021**, *23*, 5503.

[6] J. G. Pereira, J. M. J. M. Ravasco, J. R. Vale, F. Queda, R. F. A. Gomes, *Green Chem.*, **2022**, *24*, 7131.

[7] R. F. A. Gomes, B. M. F. Gonçalves, K. H. S. Andrade, B. B. Sousa, N. Maulide, G. J. L. Bernardes, C. A. M. Afonso, *Angew. Chem. Int. Ed.*, **2023**, *62*, e202304449.

[8] M. Sortino, F. Garibotto, V. Cechinel-Filho, M. Gupta, R. Enriz, S. Zacchino, *Bioorg. Med. Chem.*, **2011**, *19*, 2823.

[9] K. Eloh, M. Demurtas, M. G. Mura, A. Deplano, V. Onnis, N. Sasanelli, A. Maxia, P. Caboni, *J. Agric. Food Chem.*, **2016**, *64*, 4876.

[10] R. Kumar, S. Chaudhary, R. Kumar, P. Upadhyay, D. Sahal, U. Sharma, *J. Org. Chem.*, **2018**, *83*, 11552.

[11] GaussView, Version 6; Dennington, Roy; Keith, Todd A.; Millam, John M. Semichem Inc., Shawnee Mission, KS, **2016**.

[12] P. Pracht, F. Bohle, S. Grimme, *Phys. Chem. Chem. Phys.*, **2020**, *22*, 7169.

[13] C. Bannwarth, E. Caldeweyher, S. Ehlert, A. Hansen, P. Pracht, Jakob S., S. Spicher, S. Grimme, *WIREs Comput. Mol. Sci.*, **2021**, *11*, e1493.

[14] Gaussian 16, Revision C.01; M. J. Frisch, G. W. Trucks, H. B. Schlegel, G. E. Scuseria, M. A. Robb, J. R. Cheeseman, G. Scalmani, V. Barone, G. A. Petersson, H. Nakatsuji, X. Li, M. Caricato, A. V. Marenich, J. Bloino, B. G. Janesko, R. Gomperts, B. Mennucci, H. P. Hratchian, J. V. Ortiz, A. F. Izmaylov, J. L. Sonnenberg, D. Williams-Young, F. Ding, F. Lipparini, F. Egidi, J. Goings, B. Peng, A. Petrone, T. Henderson, D. Ranasinghe, V. G. Zakrzewski, J. Gao, N. Rega, G. Zheng, W. Liang, M. Hada, M. Ehara, K. Toyota, R. Fukuda, J. Hasegawa, M. Ishida, T. Nakajima, Y. Honda, O. Kitao, H. Nakai, T. Vreven, K. Throssell, J. A. Montgomery Jr., J. E. Peralta, F. Ogliaro, M. J. Bearpark, J. J. Heyd, E. N. Brothers, K. N. Kudin, V. N. Staroverov, T. A. Keith, R. Kobayashi, J. Normand, K. Raghavachari, A. P. Rendell, J. C. Burant, S. S. Iyengar, J. Tomasi, M. Cossi, J. M. Millam, M. Klene, C. Adamo, R. Cammi, J. W. Ochterski, R. L. Martin, K. Morokuma, O. Farkas, J. B. Foresman, D. J. Fox, Gaussian, Inc., Wallingford, CT, **2016**.

[15] A. D. Becke, *J. Chem. Phys.*, **1993**, *98*, 5648.

[16] J. P. Perdew, K. Burke, M. Ernzerhof, *Phys. Rev. Lett.*, **1996**, *77*, 3865.

[17] (a) S. Grimme, J. Antony, S. Ehrlich, H. Krieg, *J. Chem. Phys.*, **2010**, *132*, 154104; (b) S. Grimme, S. Ehrlich, L. Goerigk, *J. Comput. Chem.*, **2011**, *32*, 1456.

[18] A. V. Marenich, C. J. Cramer and D. G. Truhlar, *J. Phys. Chem. B*, **2009**, *113*, 6378.
